# Deconvolution of haematological cancer methylation patterns reveals a predominantly non-disease related proliferation signal and uncovers true disease associated methylation changes

**DOI:** 10.1038/s41416-025-03239-3

**Published:** 2025-10-31

**Authors:** H. Lalchungnunga, Hande Atasoy, Edward C. Schwalbe, Chris M. Bacon, Gordon Strathdee

**Affiliations:** 1 Newcastle University Centre for Cancer, Biosciences Institute, Newcastle University, Newcastle, UK; 2 Center for Cancer Research, National Cancer Institute Bethesda, Bethesda, MD USA; 3 Necmettin Erbakan University, Selçuklu/Konya, Turkey; 4 Department of Applied Sciences, Northumbria University, Newcastle, UK; 5 Newcastle University Centre for Cancer, Translational and Clinical Research Institute, Newcastle University, Newcastle, UK

**Keywords:** Cancer genomics, Haematological cancer, Epigenomics

## Abstract

**Background:**

Cancers are associated with extensive reorganisation of epigenetic patterns, making identification of DNA methylation changes responsible for driving cancer development challenging. Here, we present a novel approach, integrative methylation mapping, which overcomes this, enabling identification of functionally relevant methylation-regulated genes in cancer.

**Methods:**

Comparison of genome-wide DNA methylation across multiple B-lymphocyte derived malignant/normal samples (total *n *= 995), enabled delineation of changes related to normal or cancer cell functions. Chromatin structure profiling (SeSAMe) analysis delineated different properties characterising the different categories of methylation change and lentiviral based re-expression enabled functional assessment of identified candidate genes.

**Results:**

This analysis determined that only 2–3% of DNA methylation changes in B-cell cancers are disease driven, with the overwhelming majority driven by normal processes, predominantly proliferation. Methylation changes associated with specific cancer or normal cell processes exhibited unique patterns of sequence context, chromatin structure and associated transcription factors. Furthermore, the low level of true disease-specific changes simplifies identification of functionally relevant methylation changes, illustrated here by identification and functional confirmation of *SLC22A15* as a tumour suppressor in acute lymphoblastic leukaemia.

**Conclusions:**

This approach leads to a clearer understanding of the role of altered DNA methylation in haematological cancer, facilitates identification of cancer-relevant DNA methylation targeted genes and novel therapeutic targets.

## Background

Development of haematological cancers is a multistep process involving accumulation of genetic and epigenetic changes, including dramatic genome-wide alterations in the pattern of DNA methylation [1]. This includes global reductions in 5-methylcytosine across the cancer genome versus normal cells, in association with the acquisition of methylation (hypermethylation) at CpG island sequences that typically have low methylation [2]. Hypermethylation of CpG islands is known to be important in cancer development and several well established tumour suppressor genes (TSG), such as *BRCA1* and *MLH1*, are primarily inactivated by promoter hypermethylation [3]. However, array and sequencing based approaches that allow genome-wide assessment have identified that thousands of gene-associated CpG islands can become aberrantly methylated in a single cancer, although likely only a small number of these have a direct functional role [1]. In addition, while for genetic changes, a high mutation frequency can be taken as a proxy for functional relevance, many of the identified methylation changes are present in essentially all cases of a particular cancer type and so a high frequency of occurrence of specific methylation changes does not imply a likely functional role in cancer development [4]. Thus, there is no clear mechanism for identifying the small number of methylation changes that are potentially important in driving cancer development from the overwhelming majority of passenger changes.

As an initial step to overcome this limitation, we developed a novel bioinformatic approach that utilises the complexity of genome-wide DNA methylation patterns to identify synthetic lethal-like genes (referred to as subtype specific vulnerability genes) that are specific for individual molecular subtypes within a certain cancer type, initially in acute lymphoblastic leukaemia (ALL) and medulloblastoma [5]. We also demonstrated a very strong overlap between altered methylation seen in ALL cells [6] with altered methylation seen in other B-cell malignancies and in long-lived normal B-cells (memory B-cells), relative to normal B-cell progenitors. Indeed, using a set of over 7000 CpG sites originally identified as altered in ALL (relative to B-cell progenitors), this analysis found that the methylation changes in ALL were highly predictive of nearly identical mirroring methylation changes in healthy memory B cells. Furthermore, the extent of methylation change in normal memory B-cells was essentially indistinguishable from more indolent B-cell malignancies such as chronic lymphocytic leukaemia (CLL) [5].

This demonstrates that many of the methylation changes seen in B-cell malignancies are not disease-specific, are independent of the differentiation status of the cancer cells, and are also present in specific populations of normal B-cells, which are long-lived and highly proliferative [7], like cancer cells. In addition, a number of other studies of B-cell malignancies have found similar results, with normal memory B-cells showing methylation changes that are highly overlapping with cancer derived from B-cells [8–10]. These results suggest that proliferation, independent of cellular transformation, may be the primary driver of altered methylation.

The similarity in disrupted methylation across all malignancies [4] suggests that the driving forces underlying altered DNA methylation in different cancer types are likely to be highly similar. Most tissues lack populations of normal cells that, like memory B-cells, are long-lived and proliferative, that could be used for comparison to cancer cells. However, similarities observed between ageing-related methylation and cancer-related methylation in multiple tissue types [11] and similarities to altered methylation in cells reaching replicative senescence [12] is consistent with the hypothesis that proliferation is the primary driver of methylation changes.

These observations suggest that by screening out methylation changes that also occur in normal cells following proliferation, true disease-associated methylation changes could be identified. Taking advantage of the naturally occurring long-lived/highly proliferative memory B-cells, this study aims to “map” the derivation of all methylation changes occurring in B-cell malignancies and to classify them into four specific types: 1) Proliferation-driven methylation changes, 2) Differentiation-driven methylation changes, 3) True disease-specific changes (i.e. changes occurring in malignant B-cells but absent from normal memory B-cells) and 4) cancer-absent methylation changes (i.e. changes occurring in memory B-cells but absent from malignant B-cells, suggesting direct selection against these methylation changes in cancer cells). We hypothesised that*, while methylation changes shared between cancer and normal cells may have functional relevance*, the final two categories will include only a small fraction of the total methylation changes and that these methylation changes will have a much greater likelihood of being functionally relevant in disease development.

## Methods

### Data used for bioinformatic analysis

All datasets are from publicly available sources. Methylation datasets were obtained from the Gene Expression Omnibus or European Genome-Phenome Archive:, for B-cell malignancies: ALL GSE49031 and GSE69229 (*n *= 517) [6, 13], CLL EGAD00010000254 (*n *= 187) [14], mantle cell lymphoma (MCL) EGAD00010001012 (*n *= 86) [15], diffuse large B-cell lymphoma (DLBCL) GSE37362 and TCGA-DLBC (n-79) [16, 17], primary central nervous system lymphoma (PCNSL) GSE92676 (*n *= 95) [18]. Normal B-cell populations: B-cell progenitor cells GSE45459 (*n *= 22) [19], Naïve B-cells, memory non-switched cells (MEM_NCS) and memory switched cells (MEM_CS) EGAD00010000254 (*n *= 9) [14]. All methylation data was processed as described [13].

Transcriptomic data sets used: Progenitor cell GSE45460 (*n *= 31) [19], Naïve B-cell, MEM_NCS, MEM_CS GSE24759 (*n *= 20) [20], ALL (GSE13159) (*n *= 326) [21], CLL EGAD00010000252 (*n *= 143) [22], MCL GSE93291 (*n *= 122) [23], DLBC GSE11318 (*n *= 203) [24], PCNSL GSE34771 (*n *= 34) [25].

### Bioinformatic and statistical analysis

**Fig. 1:**
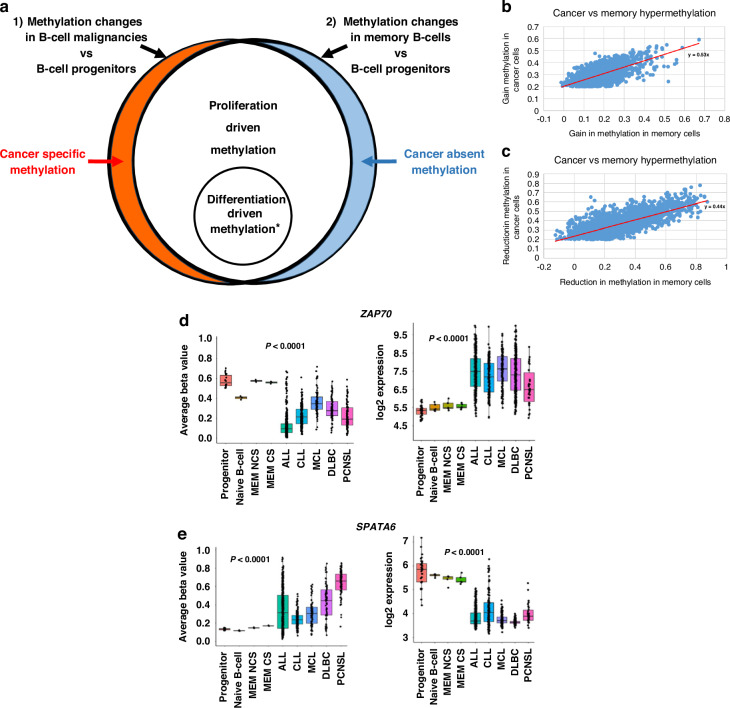
Identification of altered DNA methylation associated specifically with cancer development. **a** Outline of approach. Initially methylation differences are identified separately from two comparison 1) All B-cell malignancies (ALL, CLL, PCNSL, MCL, DLBCL) vs B-cell progenitors and 2) Memory B-cells versus B-cell progenitors. DMRs that are identified in all B-cell malignancies and also in memory B-cells (in comparison to B-cell progenitors) are regarded as “proliferation driven). *Differentiation driven methylation changes are also present in both sets, but only shared with mature B-cell malignancies (CLL, PCNSL, MCL, DLBCL) and specifically absent from ALL. Cancer-specific methylation changes represent a small minority of the total changes and include both “cancer-specific methylation” (present in all B-cell malignancies but absent in memory B-cells) and” cancer absent methylation” (present in memory B-cells but absent in all B-cell malignancies). **b**, **c** Methylation differences (DMRs) were identified by comparing all B-cell malignancies combined vs B-cell progenitors. The level of methylation changes in the B-cell malignancies in DMRs that were hypermethylated (**b**) or hypomethylated (**c**) were then compared to methylation differences at the same DMRs in the comparison of normal memory B-cells vs B-cell progenitors. Overall, changes in the DMRs identified in B-cell malignancies were found to be largely replicated in normal memory B-cells (Pearson correlation coefficient *r* = 0.94). **d**, **e** Examples of loci exhibiting B-cell malignancy specific methylation and expression patterns. Methylation (left-hand graph) and expression (right-hand graph) patterns for the three loci identified with DMRs located in their proximal promoter regions and exhibiting a negative correlation with gene expression. **a** ZAP70 and **b** SPATA6. Methylation and expression is shown for normal cell types (B-cell progenitors (progenitor) and class-switched memory B-cells (MEM CS)) and for the five B-call malignancies used in this study.

Bioinformatic analyses were undertaken using R v3.4.0 (https://www.r-project.org/foundation). Differentially methylated regions (DMR), identified using DMRcate [26], were selected based on the average beta-value difference across the full DMR exceeding 0.2 (or 0.1, when stated), the minimum number of CpG sites to define a DMR was two and significance cut-off was *p *< 0.0001. For initial integrative methylation mapping, DMR data sets were generated by comparing 1) B-cell malignancies (all five B-cell malignancies combined) vs B-cell progenitors and 2) Normal memory B-cells vs B-cell progenitors. The generated DMR datasets were then compared to allow characterisation of the identified DMRs using the criteria illustrated in Fig. 1a, with detailed criteria in supplementary methods.

Identification of disease specific dependency genes and disease specific tumour suppressor genes was performed using our previously developed bioinformatic approach [5] with the five different B-cell malignancies used as the “subtypes” for the analysis.

The SeSAMe package [27] was used to identify enriched genomic features within the filtered DMR sets. For each comparison, SeSAMe was used to perform Fisher’s Exact tests for enrichment of defined genomic feature sets, comparing the local genomic features defined by CpGs within a set of DMRs against genomic features associated with all probes within the Illumina Human Methylation 450k array; two-sided testing was performed throughout. The genomic feature sets were CpG island context (i.e. location with respect to proximal CpG islands), chromHMM (chromatin state), HMconsensus (histone modification), and TFBSconsensus (transcription factor binding sites (TFBS). Enrichments were visualised using SeSAMe.

Statistical assessments of differences in cell growth and apoptosis were carried out using t-tests, assuming equal variances.

### Quantitative RT-PCR

qPCR reactions were carried outed using Platinum SYBR Green qPCR SuperMix-UDG with ROX (Invitrogen, UK) following the manufacturer’s protocol. Total volume per reaction was 10 μl. This included 5 μl Syber Green Master mix, 2 μl CDNA, 0.8 μl primers mix (Eurofins Genomics, Luxembourg) at 300 ng/μl and 2.2 μl water. Each sample was performed in triplicate in a 384- well plate ((Thermo Fisher Scientific)) and sealed with MicroAmp optical adhesive film (Applied Biosystem). A water control, and no RT controls were included. The samples were run on a QuantStudio 7 Flex Real-Time PCR system and analysed using QuantStudio Real-Time PCR software v1.2 (both Applied Biosystems). *GAPDH* was used as reference.

### Apoptosis assays

Apoptosis was assessed by two methods. Firstly, the APC Annexin V (APC-labelled) and PI staining kit (BioLegend Cat No: 640932) was performed according to the manufacturer’s instructions, staining 100,000 cells for each cell line/condition. Flow cytometry was performed using the FACS Fortessa X-20. Apoptosis was assessed at multiple time points post-transduction, as indicated. FCS Express-7 software was used to analyse the results. Further confirmation of apoptosis was performed using the Caspase-Glo 3/7 Assay (Promega UK, Cat No:8091). Caspase 3/7 activity was measured at days 3-5 post-transduction in 96-well plates, according to the manufacturer protocols. Blank wells, with only reagent and media without cells, were used as blank controls and untreated (parental) and mock-treated cells were used as negative controls. All experiments were performed in triplicate. Luminescent readings were taken using the FLOUstar Omega plate reader (BMG LABTECH).

### Assessment of cell growth

Staining with eBioscience™ Cell Proliferation Dye eFluor™ 450 (Invitrogen) was performed to asses cell proliferation. At day3 post-transduction, 250,000 cells were washed twice with pre-warmed PBS, re-suspended in 500 μl PBS and then stained with cell proliferation dye according to the manufacturer’s instructions. Samples were analysed at multiple subsequent time points (as indicated in text) until day 17 post-transduction, after which fluorescent levels dropped too low to see detectable differences after additional rounds of cell division. Data was analysed using FCS Express 7 software.

### Lentiviral transduction

The pSINE-SIEW vector (gift from Dr Paul Sinclair) was used for lentiviral transductions. This vector allows for high efficiency expression of cloned sequences, in addition to eGFP (expressed from the same transcript, but as a separate protein via an internal ribosome entry site). The THEM4 donor clone was purchased from GeneCopoeia (GC-11844), TTC12, MAP9 and SLC22A15 donor clones were purchased from as Genescript. Subcloning into pSIN-SIEW-GTW plasmid was performed using gateway technology (Gateway™ LR Clonase™ II Enzyme Mix, Invitrogen) as per manufacturer’s protocol. Generation of lentiviral particles was performed by co-transfection of the appropriate lentiviral expression vectors, and the pMD2.G and pCMVΔR8.91 packaging vectors, into the 293 T packing cell line, using the EndoFectin Lenti transfection reagent (GeneCopoeia).

Transduction with lentiviral particles was performed using 2.5 ×105 target cells/well in a 12-well plate in 1 ml fresh RPMI1640 media, containing 5% calf foetal serum. 500 µl lentiviral-containing media was added to each well in a total volume of 1.5 ml. This was incubated at 37 °C and 5% CO2 for 16–20 h. Untreated (parental) and mock-treated cells were used as controls. After incubation, at post-transduction day 1, cells were washed twice with PBS and 1.5 ml fresh RPMI 1640 media added. This was incubated in the standard growth conditions and transduced cells were analysed by flow cytometry at multiple time points post-transduction, as indicated in the text.

### Western blotting

Proteins were extracted using sonication after treating with SDS-lysis buffer. Pierce BCA protein Assay Kit (Thermo Fisher Scientific) was used to estimate protein concentration. 15–30 µg of protein was loaded onto gels (Mini-protein precast genes, Bio-Rad). After electrophoresis, proteins were transferred onto Polyvinyl difluoride transfer membranes (Immuno- Blot PDVF Membrane, Biorad, UK). Membranes were blocked in 5% dried skimmed milk with 0.1% Tween-20 (TBST) for 1 hr at room temperature, after which they were probed with primary antibodies overnight at 4 °C. Primary antibodies used: anit-THEM4 (1:1000, Novus Bio (NBP2-94437)), anti-SLC22A15 (1:1000, Proteintech (20626-1-AP)) and anti-B-actin (1:10000, Abcam (ab49900)). Following incubation with the corresponding horseradish peroxidase-conjugated secondary antibodies, immunoreactive bands were visualized by enhanced chemiluminescence detection (Pierce ECL Western Blotting Substrate, Thermo Fisher Scientific Cat No: 32106) and ChemiDocTM MP imaging System (Bio-Rad).

## Results

### Methylation changes in B-cell malignancies are dominated by a proliferation signature

To map the derivation of methylation differences in B-cell malignancies, we identified differentially methylated regions (DMRs) identified between multiple types of B-cell malignancies and normal B-cell populations. This included malignancies arrested at the progenitor (ALL) and at mature stages of B-cell differentiation (CLL, DLBCL, MCL and PCNSL) (Fig. 1a) and normal healthy B-cells at an early (B-cell progenitors) and late differentiation stage (memory B-cells). B-cell progenitors are used as the “baseline” as, in addition to being normal/non-cancerous, they will have a relatively limited proliferation history (the total number of historical rounds of proliferation will be low). Inclusion of the memory B-cell population allowed identification of methylation changes that occur following proliferation of non-transformed B-cells. Thus, methylation differences shared in the comparison of all B-cell malignancies vs B-cell progenitors and memory B-cells vs B-cell progenitors must result from processes independent of transformation (as they are present in normal memory B-cells) and independent of differentiation status (as they are present in ALL) (Fig. 1a). This analysis also allows identification of methylation changes specific for differentiation, which will be absent in cancer/normal cells derived from undifferentiated populations but present in differentiated cancer/normal cells (Fig. 1a). The specific criteria used for identification of all DMR groups is detailed in Supplementary Fig. 1. Exclusion of DMRs associated with normal cell processes then enables the identification of two categories of DNA methylation changes specifically associated with the development of B-cell malignancies (cancer-specific and cancer-absent, Fig. 1a).

Initially, the analysis was performed to examine methylation changes shared across all B-cell malignancies. As shown in Fig. 1b, c, the overall altered methylation landscape of B-cell malignancies was highly similar to altered methylation seen in normal memory B-cells (r = 0.94, *p *< 0.0001), with less than 1% of DMRs (53 in total) exhibiting changes in the opposite direction in normal memory cells compared with the malignancies (dots below 0 on X axis in Fig. 1b, c). The pattern was dominated by a proliferation signal (87.9% of DMRs, Table 1). A further 9% of DMRs were found to map to the differentiation category and only 3% (174/5692) of DMRs mapped to the two categories in which methylation differences are specifically associated with malignancy (Table 1).

**Table 1:** Derivation of DMRs in B-cell malignancies.

DMR	Total DMRs^a^		Fraction of total DMRs (%)	
Group	0.2 cut-off	0.1 cut-off	0.2 cut-off	0.1 cut-off
Cancer specific	156	224	2.7	1.7
Cancer absent	18	56	0.3	0.4
Differentiation	514	4091	9.0	31.5
Proliferation	5004	8628	87.9	66.4

^a^Total DMRs from a comparison of B-cell progenitors vs all B-cell malignancies combined.

The analysis performed above focuses on DMRs with relatively large methylation changes (minimum beta-value difference of 0.2, approximately equivalent to a difference of 20% methylation). To examine the possibility that the low level of cancer-specific changes was related to a focus on large differences in methylation, we repeated the full analysis reducing the minimum beta-value difference to 0.1. As expected, this resulted in a large increase in the total number of DMRs (Table 1). The total number of potential cancer-specific DMRs increased (from 174 to 280). However, this represented a fall in the fraction of DMRs in the two cancer-specific categories (from 3% to 2.1%). Methylation changes associated with normal cell processes still dominate the pattern, with a higher fraction of differentiation related changes (Table 1). These results demonstrate that, in B-cell derived cancers, the number of methylation changes that are specifically associated with disease development is far smaller than previously believed and represents a very small fraction of the differences identified by comparing cancer cells to matched normal cells. Furthermore, matching differentiation status would only remove a modest fraction of the non-disease related methylation differences (Table 1).

### Characterisation of proliferation, differentiation and cancer-specific DMRs

Proliferation associated DMRs exhibited significantly higher numbers of CpG sites (average 7.3 CpGs/DMR vs 3.5–4.4 for other groups, *p *< 0.0001, Table 2) and were more likely to be proximal to transcriptional start sites (TSS) (34% versus 19–28% for other groups, *p *< 0.0001, Table 2). Proliferation associated DMRs exhibited similar levels of hyper and hypomethylation (53.4% of DMRs hypermethylated), while the differentiation associated DMRs were heavily skewed towards hypomethylated DMRs, with only 5.1% hypermethylated (*p *< 0.0001 vs proliferation DMRs). Cancer-specific DMRs were intermediate with 19.9% DMRs hypermethylated (*p *< 0.0001 vs both). Cancer absent DMRs were predominantly hypermethylated (88.9%, *P *< 0.01 vs any other group), although the absolute number of DMRs (18) was very small.

**Table 2:** Characteristics of DMR groups.

DMR group	CpG sites^a^		DMR size	
	Average CpG sites	Range	Average (bp)	Range (bp)
Proliferation	7.29	(2–110)	1307	4–24170
Differentiation	3.51	(2–29)	597	5–4534
Cancer specific	4.35	(2–18)	756	9–3060
Cancer absent	4.44	(2–13)	630	17–2018

	Hypermethylation vs hypomethylation		
	Total DMRs hypermethylated	Total DMRs Hypomethylated	fraction hypermethylated (%)
Proliferation	2673	2330	53.4
Differentiation	26	488	5.1
Cancer specific	31	125	19.9
Cancer absent	16	2	88.9

	Distance to TSS^b^		
	<1000 bp	>1000 bp	Fraction < 1000 bp (%)
Proliferation	1723	3280	34.4
Differentiation	112	402	21.8
Cancer specific	29	127	18.6
Cancer absent	5	13	27.8

^a^Average and range of CpG sites included in DMRs. This represents the number of CpG sites on the methylation array that are in the DMR and not the total number of CpG sites in the genomic region (as the methylation status of intervening CpG sites not represented on the array is unknown, they are not included in the above figures).

^b^DMRs are stratified by distance from TSS, either <1000 bp or >1000 bp. DMRs that lie near (<1000 bp) a TSS would likely influence gene expression when heavily methylated. DMRs more distal to TSS ( > 1000 bp) would have a less clear impact on expression of the nearest gene.

### Identification of ZAP70 as a functionally important target across all B-cell malignancies

A key aim of integrative methylation mapping was to facilitate the identification of functionally relevant methylation changes. To this end, transcriptome datasets from the cancer and normal cell populations were investigated to identify cancer specific/absent changes (using the 0.2 beta-value cut-off) exhibiting significant negative correlations with expression from the nearest gene. Of the 174 cancer specific/absent DMRs identified, 10 exhibited significant correlations with differential expression (Supplementary Table 1). However, these loci exhibited features suggestive that this correlation was not necessarily indicative of a direct role for differential methylation in regulating gene expression; the loci did not show an excess of negative correlations, 6/10 were in regions of low CpG density (only 2–3 CpG sites in the DMR) and 5/10 were highly distal ( > 40Kb) from the nearest TSS, with only 2/10 mapping to the proximal promoter region of a gene (<1 kb from TSS, *SPATA6*, *ZAP70*) (Fig. 1d, e). *ZAP70* was the clearest candidate for a pan B-cell malignancy driver, and the only one of the two candidates with a beta-value difference >0.2 (versus normal progenitors and memory cells) in all B-cell malignancies (Fig. 1d, Supplementary Table 1). Consistent with this, the *ZAP70* gene has been clearly implicated in the development of chronic lymphocytic leukaemia [28]. However, the consistent differences in methylation and expression across all B-cell malignancies suggest that analysis of its wider role across many B-cell derived malignancies would be merited.

### Integrative methylation mapping applied to individual cancers

It is possible that most key functional changes in DNA methylation may be specific for individual B-cell cancers. Thus, the analysis was repeated examining each individual B-cell malignancies (Supplementary Fig. 1E). Disease specific DMRs were defined as DMRs present in the specific disease (with a beta-value difference >0.2) but absent in any of the other groups (other B-cell malignancies and memory B-cells). “Absence of change” was defined as <0.1 beta-value difference or a change in the opposite direction. As can be seen from Table 3, this analysis also identifies a comparatively low fraction of methylation changes as being specific for individual disease, ranging from 0.1% to 5.4% across the five malignancies. Notably, three of the five malignancies exhibit only very small numbers of disease specific DMRs (6–17 DMRs or 0.1–0.15% of total DMRs for CLL, MCL and DLBCL (Table 3)), indicating a near absence of disease specific methylation changes. The results for ALL and PCNSL suggest that disease specific methylation changes may be more relevant in these malignancies with 240 (4.8% of total DMRs) in ALL and 1104 (5.4% of total DMRs) in PCNSL. We have previously observed higher absolute levels of methylation change in PCNSL at loci that also acquire methylation changes in other B-cell malignancies (Schwalbe et al. 2021). Thus, the high level of DMRs in PCNSL may primarily reflect this greater absolute size of methylation change, as opposed to true specificity. Consistent with this, use of a more stringent definition for disease specificity (defining “absence of change” in the other four cancer types as >4x lower than the change in the specific disease) would retain only 28% of the “specific” DMRs in PCNSL (reducing the fraction of specific DMRs to 1.5% of total DMRs). Applying the same criteria to ALL retains 78% of the DMRs, (reducing the fraction of specific DMRs to 3.4% of total DMRs). As for the all B-cell malignancy analysis, this approach can also identify disease absent methylation changes, but these were rare in all B-cell malignancies (only between 4 and 11 disease absent DMRs were identified). For ALL, this approach cannot identify disease absent DMRs, as their methylation patterns would be identical to normal differentiation related DMRs.

**Table 3:** Disease specific DMRs.

	Disease related (0.2 cut-off)^a^				
Disease	Total DMRs	All disease related	Fraction of total DMRs^b^	Disease specific	Disease absent
ALL	4988	240	4.80%	240	NA^c^
CLL	6326	6	0.10%	2	4
MCL	6659	10	0.15%	4	6
DLBCL	11701	17	0.15%	7	10
PCNSL	20583	1104	5.40%	1093	11

Disease	Disease related (0.1 cut-off)^d^			DMR characteristics (0.1 cut-off)		
	All DMRs	All disease related	Fraction of total DMRs	average CpG sites	Fraction <1000 bp to TSS	Fraction hypermethylated
ALL	14208	463	3.26	7.1	38.0	69.4
CLL	18544	48	0.26	6.4	25.0	22.9
MCL	21709	61	0.28	7.3	24.6	26.2
DLBCL	27335	106	0.39	5.8	24.5	20.8
PCNSL	34819	1612	4.63	6.4	33.3	3.4

^a^DMRs (relative to B-cell progenitors) using a minimum beta value cut-off of 0.2.

^b^Fraction of the total DMRs that are disease related (including disease specific and disease absent).

^c^Disease absent DMRs cannot be identified in ALL as their methylation patterns would be identical to normal differentiation related DMRs.

^d^DMRs (relative to B-cell progenitors) using a minimum beta value cut-off of 0.1.

As with the analysis of all B-cell malignancies combined, using a beta-value difference from 0.1 (as opposed to the original 0.2) increases the total number of DMRs identified, but does not increase the fraction of those DMRs which are identified as disease specific (Table 3). Thus, analysis at the individual disease level also determined that only a small fraction of DMRs identified in the individual diseases are disease specific.

### Regions targeted in disease specific methylation show similarities to proliferation driven methylated regions

Disease specific DMRs in ALL showed a clearly higher fraction of hypermethylated regions compared with any of the other B-cell malignancies (*p *< 0.0001 for all comparisons, Table 3) and also the highest fraction of DMRs located proximal to gene promoters (although differences from other diseases were not statistically significant, Table 3). In contrast, PCNSL exhibited a strong link with hypomethylation, with 96.6% of all PCNSL-specific DMRs being hypomethylated (*p *< 0.0001 versus all other individual B-cell malignancies). For all five B-cell malignancies the disease specific DMRs exhibited relatively high CpG densities (average of 5.8–7.3 CpG/DMR), in a similar range to those seen for proliferation derived DMRs in the original analysis (average of 7.3 CpG/DMR) (Table 3).

**Fig. 2:**
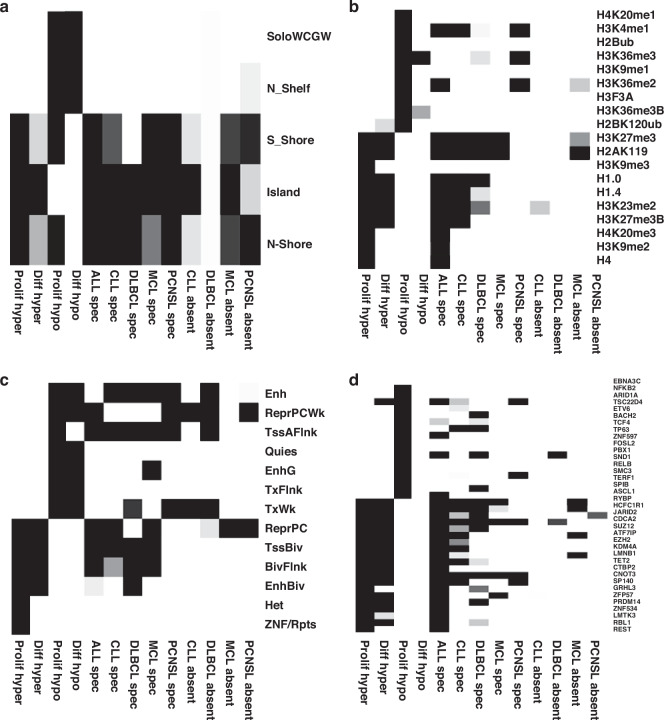
SeSAMe analysis of DMRs associated with normal and cancer-specific methylation changes. SeSAMe analysis was carried out to compare the characteristics of DMR regions associated with normal and cancer-specific processes. For all four domains up to 20 associations are shown for each group (with fewer shown if there were less than 20 associations with significant ( < 0.05) FDR values). Darker shading indicates an increasing strength of association and white/no shading that no significant association was identified. Associations are shown for (**a**) sequence context, **b** histone modifications, **c** chromatin states, **d** Transcription factor binding sites. While the chromatin states (**c**) exhibit a mixed pattern, for the other three domains there is a pronounced overlap between the cancer-specific associations in the B-cell malignancies and the normal methylation changes associated with hypermethylated regions.

To begin investigating the potential molecular basis of the identified DMR groups, the SeSAMe package [27] was used to identify specific DNA/chromatin structural elements associated with the different DMR groups. The results of this analysis are outlined in Fig. 2 (full details are in supplementary Figs. 2–5). This approach assesses the different DMR groups across four domains: Sequence context, chromatin state, histone modifications and transcription factor binding sites. For this analysis, the larger DMR groups from the pan-b-cell cancer analysis (proliferation and differentiation groups) were separated into hypermethylated (in B-cell malignancies/memory B-cells vs B-cell progenitors) and hypomethylated (in B-cell malignancies/memory B-cells vs B-cell progenitors) DMRs. Consistent with previous reports [29, 30], both proliferation and differentiation related hypermethylated DMRs are associated with CpG island and shore sequences, with polycomb complex regulated and bivalent promoter regions, and with regions linked to H3K9 and H3K27 trimethylation. In contrast, hypomethylated DMRs were linked to CpG sparse regions, regions associated with quiescence and enhancer regions and with H3K36 trimethylation. Notably, the disease specific DMRs show patterns similar to the proliferation/differentiation hypermethylated pattern in terms of three of these domains (Fig. 2). This is true for ALL, in which the disease specific DMRs are predominantly hypermethylated, but is also true for CLL, MCL and DLBCL in which the DMRs are predominantly hypomethylated. Of note, in terms of histone modifications, all these groups of disease specific DMRs are specifically associated with regions linked to H3K27 trimethylation but not H3K9 trimethylation. The same pattern is also seen for differentiation-associated hypermethylated DMRs, while proliferation DMRs are uniquely, and strongly, linked to H3K9 trimethylation. Disease specific DMRs identified in PCNSL, unlike the other diseases, exhibited patterns related to proliferation/differentiation hypomethylated region, consistent with the extreme association within this group to hypomethylated DMRs.

The analysis of transcription factor binding sites may allow identification of factors that could play active roles in promoting methylation changes in the different DMR groups. Consistent with the association of most disease specific DMRs with the hypermethylated regions driven by normal processes, multiple transcription factor binding sites were found to be shared across all these groups, including a core set of five TFBS (CDCA2, CNOT3, HCFC1R1, RYBP, SP140) (Fig. 2). Despite this similarity, associations with core PRC2 proteins (EZH2, SUZ12, JARID2) were amongst the strongest in the proliferation/differentiation DMR groups but absent or only very weakly associated in the disease specific DMR groups. TET2 binding, a factor strongly linked to myeloid malignancies, was found to associate specifically with proliferation DMRs and ALL specific DMRs (supplementary Fig. 5), suggesting a potential role for TET2 in ALL specific methylation changes. Furthermore, most of the DMR groups are associated with a set of unique TF binding sites that may have important roles in modulating methylation changes in that group (supplementary Fig. 5).

### Candidate gene identification in individual diseases

Genome-wide expression data for each of the five malignancies was assessed to identify genes linked to DMRs that showed reciprocal specific differences in gene expression. This analysis identified a total of 15 candidate genes across four of the five diseases (no candidates were identified for DLBCL). Analysis of ALL, which had the greatest fraction of disease specific methylation changes, also resulted in the identification of the largest number of candidate genes (Supplementary Table 2).

### Similarity of candidates identified through adaption of an alternative bioinformatic approach

The relatively small number of candidate genes identified in B-cell malignancies above could suggest that the integrative methylation mapping approach lacks sensitivity. To investigate this possibility, we used an alternative approach for identification of functionally relevant targets for aberrant methylation and then assessed the extent of overlap in the candidate genes identified. This second method is based on our previously reported bioinformatic approach [5], which can be used to identify cancer subtype specific synthetic lethal and tumour suppressor genes. The very high degree of overlap in DNA methylation changes between the different B-cell malignancies allows a similar approach to be used, utilising the individual B-cell malignancies as the “subtypes” in the analysis. Utilisation of this approach identified five tumour suppressor candidates and eight synthetic lethal-like genes (referred to as disease specific dependency genes) across the five malignancies. As shown in Supplementary Table 2, these exhibit a highly significant overlap with the candidates identified through integrative methylation mapping (10/13 candidates identified by this approach (“Subtype Analysis”) were also identified through integrative methylation mapping). Methylation and expression patterns of the identified candidates are detailed in Supplementary Fig. 6. While each approach identifies some additional unique candidates, the number of candidates identified remains low, consistent with the low frequency of true disease-specific methylation changes.

### Functional assessment of novel tumour suppressor candidates

Amongst the candidates that were identified by both approaches was a set of four candidate tumour suppressor gene candidates identified in ALL (*THEM4, MAP9, SLC22A15, TTC12*). To allow assessment of function independent of specific ALL genetic subtype, functional assessment was carried out in a panel of cell lines that included representatives of multiple different ALL cytogenetic subtypes. Re-expression of candidates was achieved using transduction with a lentiviral expression construct which also co-expresses eGFP from the same transcript to allow transduced cells to be tracked by flow cytometry. Correct cloning and lack of genetic disruption of the candidates in the expression construct was confirmed by sequencing. One of the four candidates (*MAP9*) failed to exhibit any detectable expression in transduced populations and so its functional impact could not be determined.

**Fig. 3:**
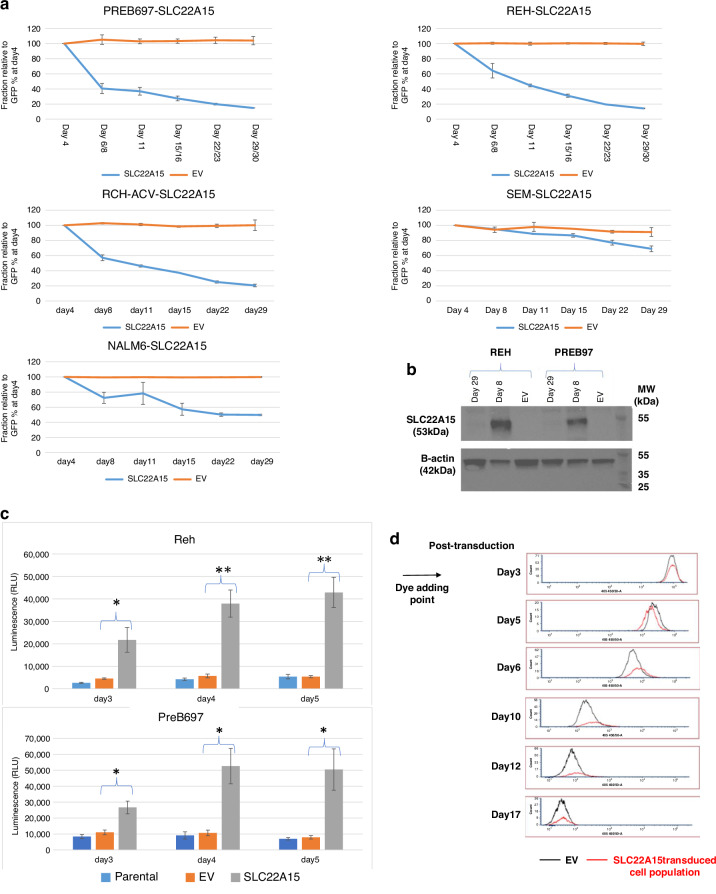
Selection against SLC22A15 expression across cell lines derived from multiple ALL genetic subtypes. **a** Following transduction of ALL cell lines with a lentiviral vector expression SLC22A15 the SLC22A15 expressing cells were lost from the population in all cell lines. By contrast, a control empty vector (expressing only eGFP) remained stable throughout the period. **b** SLC22A15 protein expression was assessed in PreB697 and Reh cells at day 8 and 29 post-transduction and in control empty vector transduced cells. Re-expression of SLC22A15 is clearly detectable at day 8, but is lost by day 29. **c** Caspase activation was assessed post-transduction in the Reh and PreB697 cells. While transduction with the control vector (EV) had no detectable impact on caspase activation, a pronounced activation of caspase 3/7 was initially detectable at day 3 post-transduction with SLC22A15 expressing vector, increasing to a maximal level by day 4/5 in both cell lines. **d** Proliferation of SLC22A15 or control EV transduced cells was assessed following staining with VPD450 (results are shown for the Reh cell line, although essentially identical results were also found in PreB697 cells). While a slight delay in proliferation appears to be present at days6-12, there is no evidence for a population of arrested cells following SLC22A15 re-expression.

Of the other three candidates, clear expression from the polycistronic transcript was detected four days post transduction (Fig. 3, Supplementary Fig. 7). As a control, a corresponding “empty vector” control transduction (expressing only eGFP) was also carried out. For one of the three candidates, *TTC12*, expression levels (assessed by fraction of eGFP positive cells) remained essentially unchanged throughout the time course (22 days) in all cell lines, suggesting that *TTC12* is either not a tumour suppressor gene or that its function is not effectively modelled in cell lines. *THEM4* expression was rapidly lost in the PreB697 cell line (t(1;19) genetic subtype) but remained relatively constant in all other cell lines, including a second t(1;19) derived cell line (RCH-ACV). In contrast, following re-expression of *SLC22A15*, loss of eGFP/SLC22A15 positive cells was seen in all five ALL cell lines assessed (Fig. 3). This was associated with a corresponding loss of SLC22A15 protein expression (Fig. 3b). Furthermore, analysis of caspase activation (in Reh and PreB697 lines) demonstrated that re-expression of SLC22A15 was associated with rapid induction of caspases 3/7 (Fig. 3c) in the absence of alteration in the underlying rate of proliferation (Fig. 3d), suggesting that re-expression of SLC22A15 resulted in induction of cell death and thus functions to inhibit ALL cell survival in all ALL cytogenetic subgroups tested.

## Discussion

Following the development of genome-wide methods for DNA methylation analysis it has been widely reported that haematological and other cancer types exhibit extensive alterations in DNA methylation compared with equivalent normal cells [1]. However, based on the results presented here only a very small proportion of these methylation changes can be regarded as truly disease-associated. Instead, the predominant drivers of alterations in DNA methylation in cancer cells are normal cell processes, specifically proliferation and, to a lesser extent, differentiation. Thus, normal Memory B cells showed highly overlapping patterns of altered DNA methylation with B-cell malignancies. Most of these changes were shared with all B-cell malignancies (proliferation-driven), whilst a smaller subset were only shared with other B-cell malignancies derived from differentiated B-cells (differentiation-driven). Although, this analysis was restricted to cancers derived from the B-lymphocyte lineage, the strong similarity in overall patterns of altered DNA methylation seen in all cancers [2] strongly suggests that the driving forces behind altered DNA methylation in cancer will be broadly similar.

An important aspect of the analysis performed here is that it is based on comparison of both cancer and normal cell populations to a “base state”, in this case B-cell progenitors. This allowed us to identify related changes in DNA methylation even when absolute size of the changes were larger in some cancer types. These differences in absolute size of methylation change are likely a product of some B-cell cancers being more proliferative and thus have greater absolute change at proliferation-driven DMRs. Consistent with this, more indolent cancers (CLL and MCL) exhibited methylation changes at similar levels to memory B-cells, while more proliferative cancers, ALL and PCNSL, exhibited consistently larger methylation changes at almost all shared DMRs.

The key aim of this approach is to identify DNA methylation changes that are truly specific for B-cell malignancies and thus enable identification of alterations in DNA methylation that are important in driving disease development or progression. Initially, we used this approach to look at DNA methylation changes occurring across all B-cell malignancies, to investigate the possibility of key drivers of transformation applicable to all malignant B-cell disease. Combined with corresponding analysis of gene expression this identified a single gene, *ZAP70*, as implicated across all B-cell cancers. ZAP70 has been extensively investigated for its role in CLL [28]. However, its role in other B-cell malignancies has received less attention [31] and the results presented here suggest that functional studies in all B-cell malignancies may be warranted. Expanding the analysis to identify DNA methylation events specific for individual B-cell malignancies allowed the identification of a larger number of candidate genes, including the identification of *SLC22A15* as a novel tumour suppressor gene in ALL. SLC22A15 encodes a solute carrier which has recently been de-orphaned and shown to have a specificity for zwitterions such as carnosine, ergothioneine and betaine [32], Little is known about its normal physiological roles, although a recent study has suggested a key role in controlling carnosine availability in the brain [33] and several studies have identified carnosine as a metabolite that shows anti-tumour activity against multiple different cancers [34–36]. Although SLC22A15 has not previously been implicated in haematological malignancies, studies have suggested a potential role in colorectal, hepatocellular and pancreatic cancer [37–39]. Thus the results presented here suggest that treatments aimed at modulating carnosine levels may be of value for treatment of ALL. This could be of particular interest as carnosine has also been found to protect against chemotherapy induced cardiotoxicity and neurotoxicity [40]. However, as SLC22A15 can be involved in both the uptake and efflux of substrates and as it is thought to have multiple substrates, further work will be required to identify the impact of SLC22A15 in the uptake and efflux of these substrates from ALL cells.

The results presented here suggest at least three separate driving forces (proliferation, differentiation, disease development) underlie the DNA methylation changes seen in B-cell malignancies and likely other cancers. Analysis of the chromatin/genomic features of these different groups found multiple differences in the associated transcription factor binding sites, locations relative to transcribed regions and associated chromatin structures, further emphasising the differences between the DMR groups. This may provide a starting point to investigate differences in the underlying mechanisms responsible for these different groups of methylation change. Such studies could enable the identification of mechanisms that are predominantly or exclusively involved in the disease associated DNA methylation changes and thus potentially allow for strategies aimed at specifically reversing or preventing changes directly involved in cancer development without having more widespread effects on normal cellular epigenetic mechanisms.

All the analysis presented here is based on data derived from Illumina beadchip methylation arrays following bisulphite conversion of DNA. This data has two key limitations. Firstly, bisulphite modification does not differentiate between 5’methyl cytosine and 5’hydoxymethylcytosine [41]. Thus any differences between in the relative accumulation of these two different modifications between normal and malignant cells cannot be assessed. Furthermore, the CpG sites represented on these arrays represent only a small fraction of the total number of CpG sites and lack full representation of some genomic regions, such as enhancers. This is especially true for the 450 K array [42] from which all data used in this study was derived. Thus the fraction of methylation changes that are disease specific in these regions remains to be fully elucidated and may vary from the rates found in this study.

Overall, the results presented here contribute to a much clearer understanding of the basis of the extensive alterations in DNA methylation patterns that characterise haematological cancer development and demonstrate that the overwhelming majority of DNA methylation changes that occur in cancer cells are the consequence of normal cellular functions and not specifically related to disease development. This deconvolution of the methylation patterns, identifying only a comparatively small number of methylation changes that are truly disease specific, opens up new opportunities for utilising true disease associated methylation changes to identify novel cancer drivers and therapeutic targets, as illustrated here by the identification of *SLC22A15* as a novel tumour suppressor candidate in ALL.

## Supplementary information

Supplementary methods

Supplementary Figures

Supplementary Tables

## Data Availability

All datasets used in this study are from publicly available sources.
