# Supplementary material for: Deconvolution of haematological cancer methylation patterns reveals a predominantly non-disease related proliferation signal and uncovers true disease associated methylation changes
Source: Br J Cancer. 2025 Oct 31;134(1):108–18. doi: 10.1038/s41416-025-03239-3 (PMC12764965; doi:10.1038/s41416-025-03239-3)
Supplement: Supplementary file 1 — Supplementary methods [file 41416_2025_3239_MOESM1_ESM.docx]

**Specific criteria used for identification of DMRs in bioinformatic analysis**

For cancer specific methylation changes, criteria used to identify changes specific for all transformed B-cell population were: 1) Methylation change in all five B-cell malignancies combined vs B-cell progenitors >0.2 beta value. 2) Methylation change seen in each individual B-cell malignancy vs B-cell progenitors >0.1 beta value. 3) Methylation change seen in Memory B-cells vs progenitors is <0.1 beta value and at least four times smaller than change in each individual B-cell malignancy.

Cancer absent methylation changes met the following criteria: 1) Methylation changes seen in B-cell memory greater than >0.2 beta value as compared to B-cell progenitor. 2) Methylation change seen in B-cell memory cell is four times greater than methylation in all five B-cell malignancies combined. 3) Methylation change seen in all individual B-cell malignancies is <0.1 beta value.

For proliferation related methylation changes, multiple criteria were defined to ensure DNA methylation changes are due to B-cell proliferation: 1) Methylation changes in total proliferated B-cells (B-cell malignancies and B-cell memory cells) are greater than >0.2 beta value compared to B-cell progenitor. 2) Methylation change seen in all individual B-cell malignancies as well as in B-cell memory B-cells compared to B-cell progenitors is >0.1 beta value.

For methylation changes due to B-cell differentiation: 1) Methylation changes in differentiated B-cells (i.e., B-cell malignancies (with the exception of ALL) and B-cell memory cells) vs B-cell progenitors >0.2 beta value. 2) Methylation change seen in an individual differentiated B-cells (B-cell malignancies and B-cell memory cells), with the exception of ALL, vs B-cell progenitors >0.1 beta value. 3) Methylation change in ALL vs B-cell progenitors <0.1 beta value.

For individual disease specific methylation changes, criteria used to identify changes were: 1) Methylation change in the specific disease vs B-cell progenitors >0.2 beta value. 2) Methylation change seen in all other B-cell malignancies and normal memory B-cells vs B-cell progenitors <0.1 beta value. To increase stringency, a third criteria 3) Methylation change in the specific disease vs B-cell progenitors at least four times greater than methylation change vs B-cell progenitors for any of the other diseases or memory B-cells, was also included where indicated.

**RNA extraction and cDNA synthesis**

Total RNA was extracted using a GeneJET RNA Purification Kit (Thermo Fisher Scientific, Cat No: K0731) according to the manufacturer’s protocol. The extracted RNA was stored at -20oC (for use in the short term) or at -80oC if long-term storage was required.

The purified RNA was quantified using the Nanodrop ND-1000 spectrophotometer (Nanodrop, Delaware, USA). About 2μg of RNA was used for cDNA synthesis using the High-Capacity cDNA Reverse Transcription Kit (Applied Biosystems, UK Cat No: 4368814) according to the manufacturer’s protocol. Samples with no reverse transcriptase (RT) were also included in the reaction to act as a control in subsequent PCR reactions.

**Cell culture**

All leukemia and lymphoma cell lines were cultured in RPMI 1640 media with L-glutamine and sodium bicarbonate (Sigma-Aldrich, UK). 293T cells were cultured in Dulbecco’s Modified Eagle’s Medium with 4500mg/l glucose, L-glutamine supplemented with foetal calf serum (Gibco, UK). Mycoplasma infection of cell lines was regularly checked for by using Mycoalert® Detection Kit (Lonza, Basel, Switzerland). Cells grown in an incubator at 37⁰C and 5% CO2. Cell lines have been recently authenticated (by Northgene, UK) by STR analysis.
