# Supplementary material for: Deconvolution of haematological cancer methylation patterns reveals a predominantly non-disease related proliferation signal and uncovers true disease associated methylation changes
Source: Br J Cancer. 2025 Oct 31;134(1):108–18. doi: 10.1038/s41416-025-03239-3 (PMC12764965; doi:10.1038/s41416-025-03239-3)
Supplement: Supplementary file 2 — Supplementary Figures [file 41416_2025_3239_MOESM2_ESM.pdf]

Supplementary Figure 1

A

Flow diagram for identification of Cancer specific DMRs

1) Identification of B-cell malignancy DMRs: Beta value difference >0.2 in all B-cell malignancies vs B-cell progenitors comparison →

2) Specificity for B-cell malignancies: Beta value difference[1] <0.1 in Memory B-cell vs B-cell progenitors comparison →

3) DMR present in all B-cell malignancies: Beta value difference >0.1 in each individual B-cell malignancy vs B-cell progenitors comparison →

4) DMR specific in all B-cell malignancies[2]: Beta value difference >four times larger in each individual B-cell malignancy than in memory B-cells

[1]Beta value differences must also be in the same direction to be defined as present in multiple populations (i.e. cancer specific DMRs need to be hypermethylated in all B-cell cancers or hypomethylated in all B-cell cancers). A lack of a difference or a difference in the opposite direction are similarly regarded as not a shared difference.
[2]This added step ensures specificity and allows exclusion of minor differences in methylation change that coincidentally lie on either side of the cut-off (e.g. a DMR with a 0.09 beta value change in memory B-cells and a 0.11 change in CLL would be excluded) and allows the analysis to account for the absolute size of the methylation change in the B-cell cancers.

B

Flow diagram for identification of proliferation DMRs

1) Identification of B-cell DMRs: Beta value difference >0.2 in all proliferated B-cell populations[1] vs B-cell progenitors comparison →

2) DMR present in all B-cell malignancies: Beta value difference >0.1 in all B-cell malignancies vs B-cell progenitors comparison →

3) DMR present in proliferated normal B-cells: Beta value difference >0.1 in all memory B-cells vs B-cell progenitors comparison

[1]Proliferated populations include all B-cell malignancies and normal memory B-cells.

C

Flow diagram for identification of differentiation DMRs

1) Identification of differentiated B-cell DMRs: Beta value difference >0.2 in all differentiated B-cell populations[1] vs B-cell progenitors comparison →

2) DMR present in all differentiated B-cell malignancies: Beta value difference >0.1 in all differentiated B-cell malignancies vs B-cell progenitors comparison →

3) DMR present in memory B-cells: Beta value difference >0.1 in all memory B-cells vs B-cell progenitors comparison →

4) DMR absent in ALL: Beta value difference <0.1 in ALL vs B-cell progenitors comparison

[1]Differentiated populations include B-cell malignancies (except ALL) and normal memory B-cells.

D

Flow diagram for identification of Cancer absent DMRs

1) Identification of Memory B-cell DMRs: Beta value difference >0.2 in memory B-cell vs B-cell progenitors comparison →

2) Specificity for memory B-cells: Beta value difference >four times larger in memory B-cells than in all B-cell malignancies →

3) DMR absent in all B-cell malignancies: Beta value difference <0.1 in each individual B-cell malignancy vs B-cell progenitors comparison

E

Flow diagram for identification of disease specific DMRs

1) Identification of Disease DMRs: Beta value difference >0.2 in specific disease vs B-cell progenitors comparison →

2) DMR absent in all other B-cell populations: Beta value difference <0.1 in other B-cell malignancies/ memory B-cells vs B-cell progenitors comparisons →

3) Increased stringency of disease specificity: Beta value difference >four times larger in specific disease than in all other B-cell populations

Supplementary figure 1. Flow diagrams outlining the criteria used for identification of DMRs associated with the different DMR groups

Supplementary Figure 2

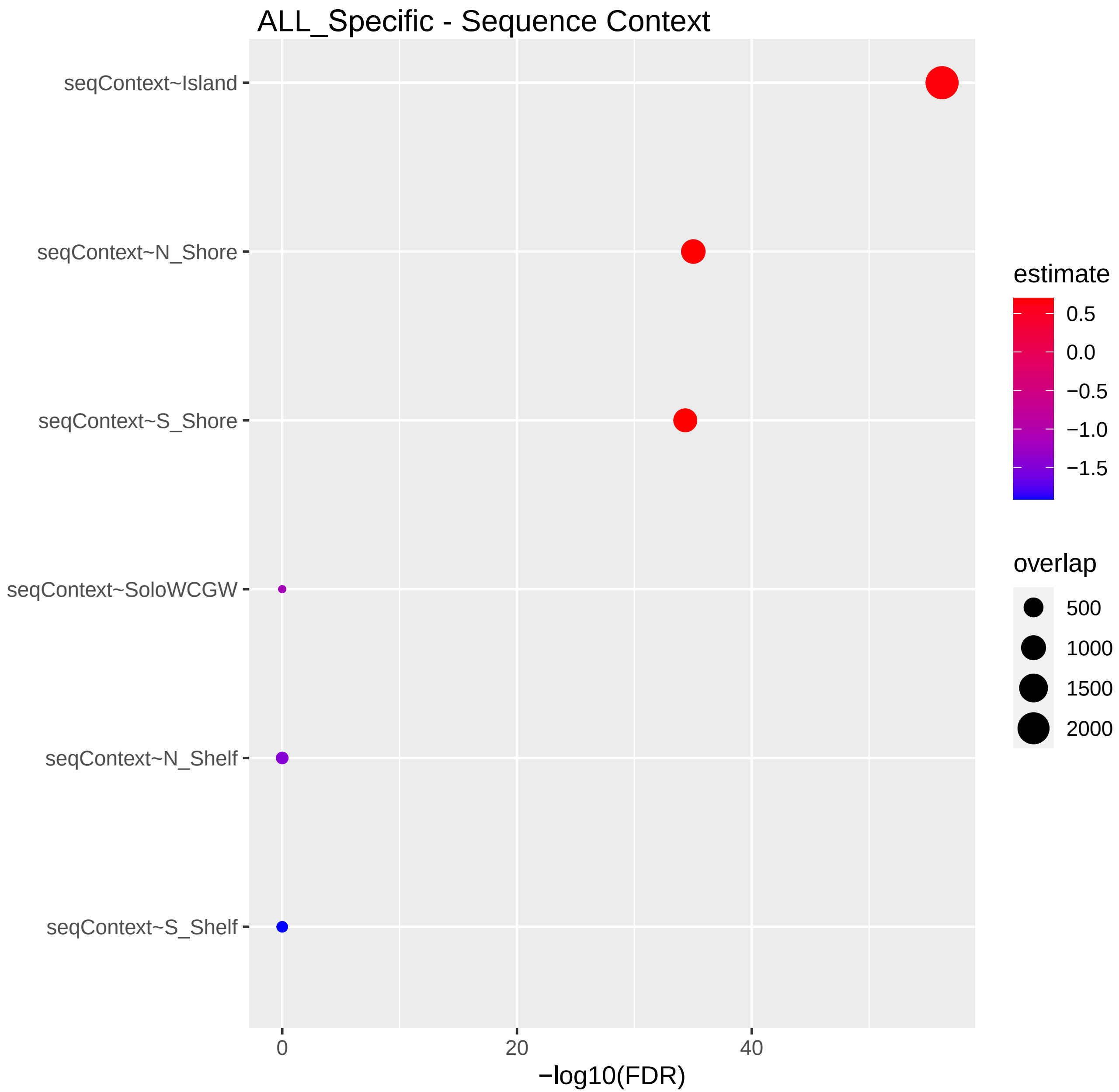

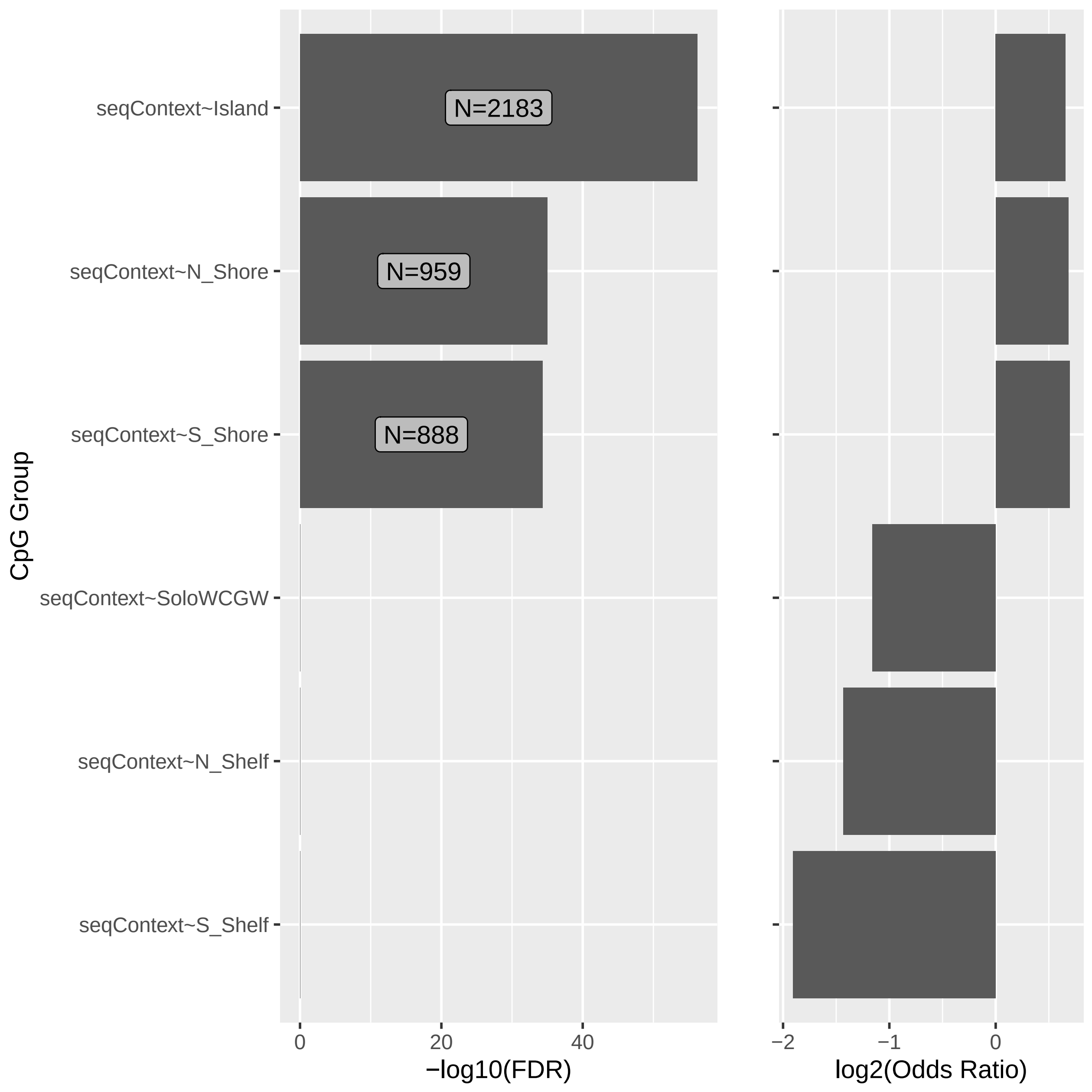

seqContext~Island
seqContext~N_Shore
seqContext~S_Shore
seqContext~SoloWCGW
seqContext~N_Shelf
seqContext~S_Shelf
CpG Group
N=2183
N=959
N=888
0
20
40
−log10(FDR)
−2
−1
0
log2(Odds Ratio)

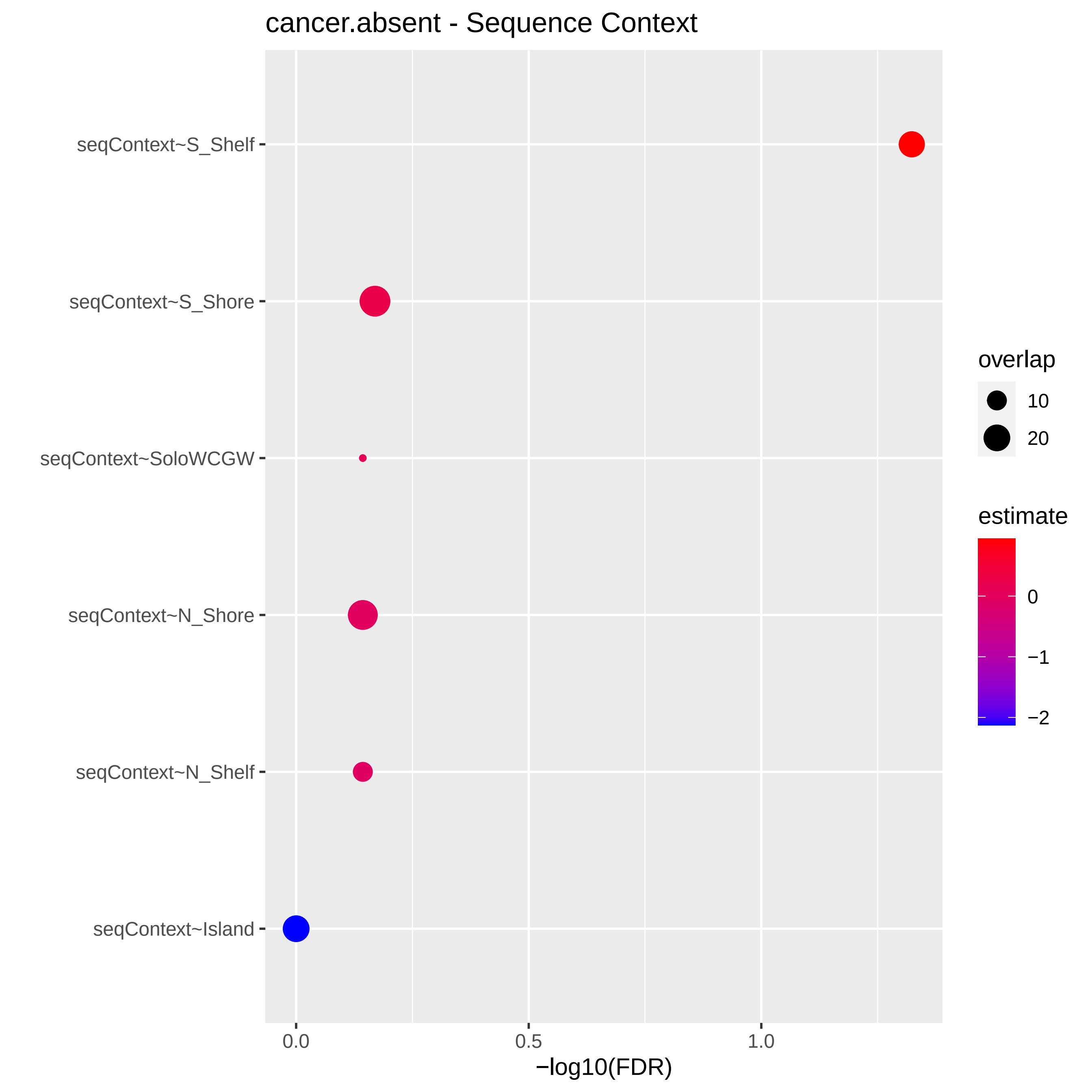
cancer.absent - Sequence Context
seqContext~S_Shelf
seqContext~S_Shore
seqContext~SoloWCGW
seqContext~N_Shore
seqContext~N_Shelf
seqContext~Island
0.0
0.5
1.0
−log10(FDR)
overlap
10
20
estimate
0
−1
−2

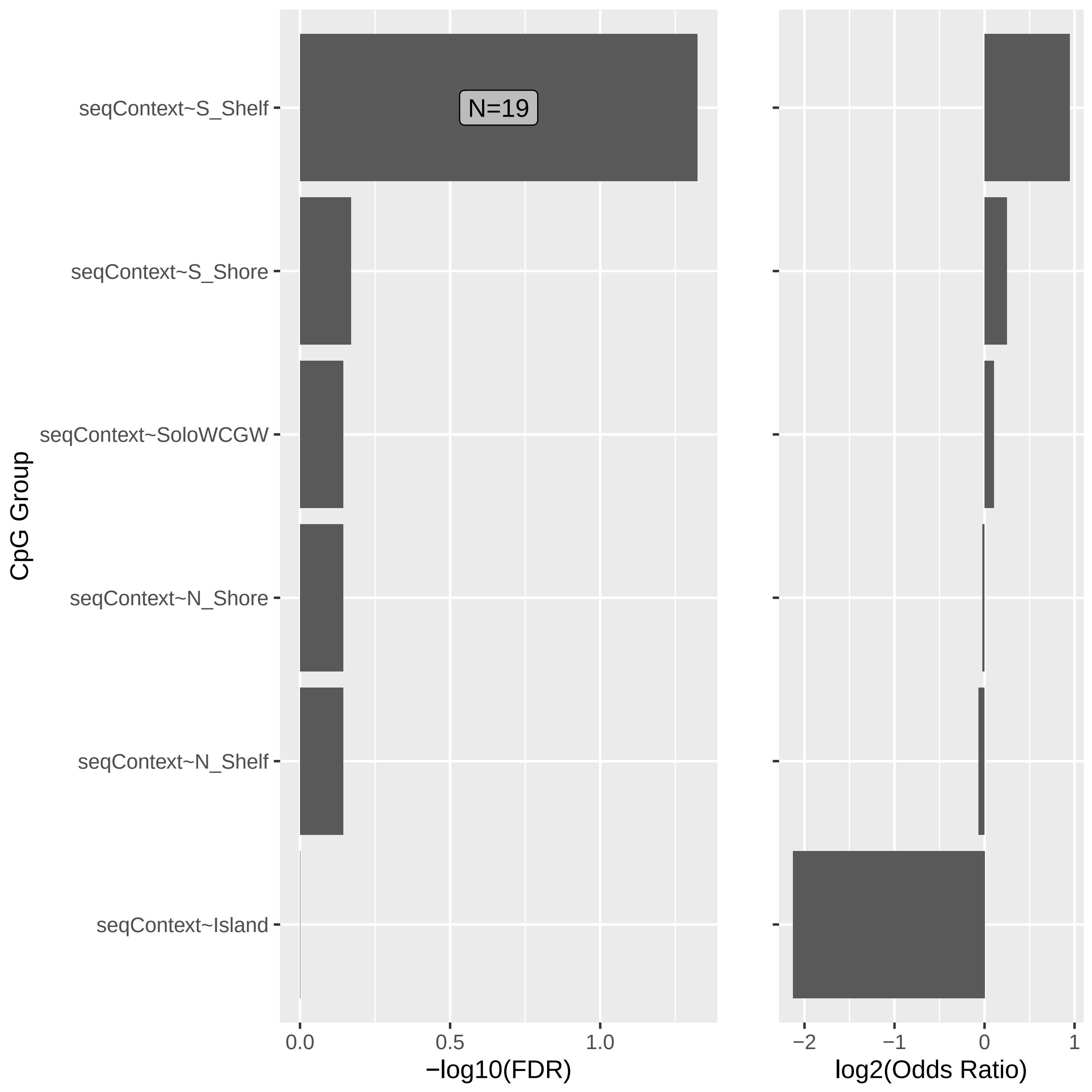

CpG Group
seqContext~S_Shelf
seqContext~S_Shore
seqContext~SoloWCGW
seqContext~N_Shore
seqContext~N_Shelf
seqContext~Island
N=19
0.0
0.5
1.0
−log10(FDR)
−2
−1
0
1
log2(Odds Ratio)

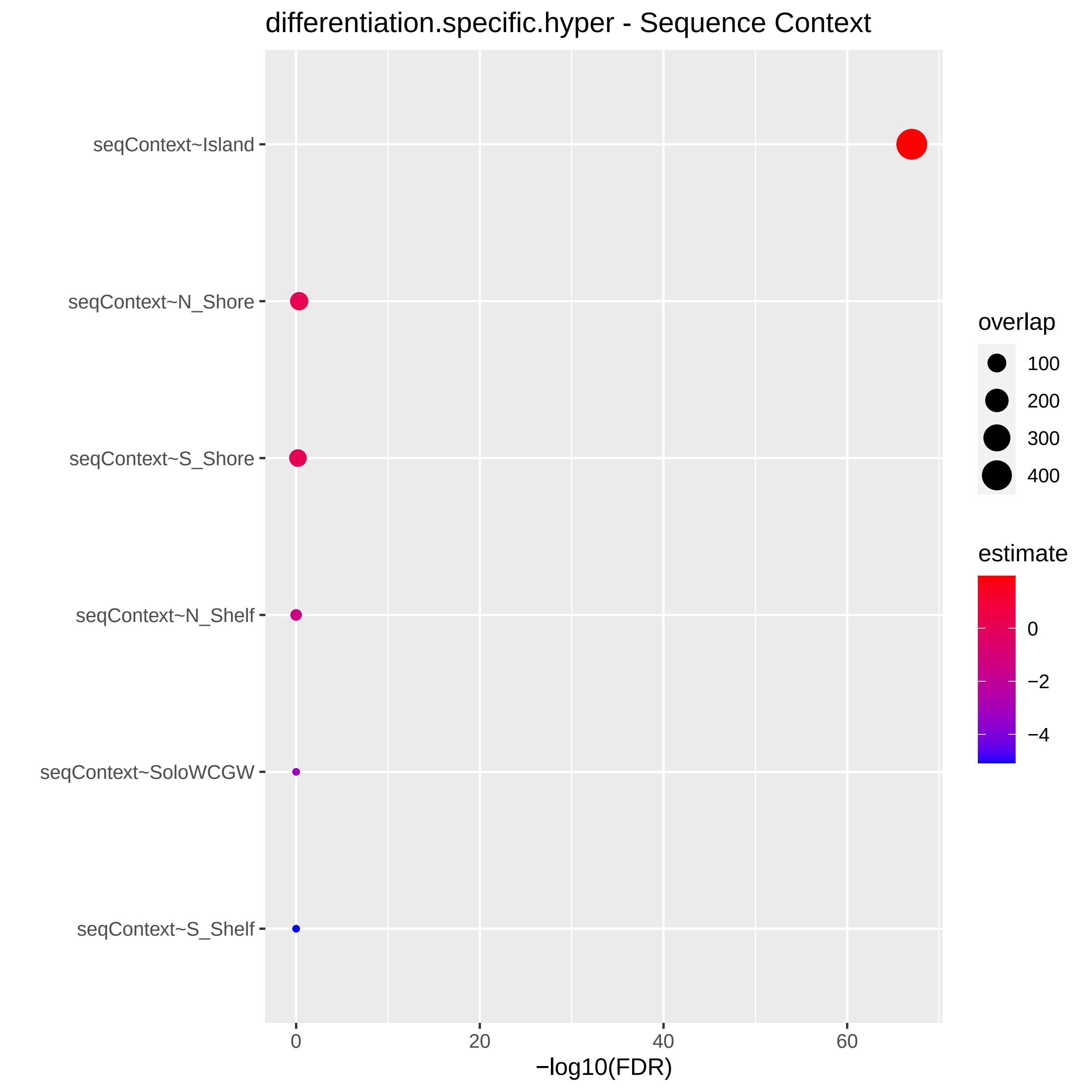

differentiation.specific.hyper - Sequence Context
seqContext~Island
seqContext~N_Shore
seqContext~S_Shore
seqContext~N_Shelf
seqContext~SoloWCGW
seqContext~S_Shelf
0
20
40
60
−log10(FDR)
overlap
100
200
300
400
estimate
0
−2
−4

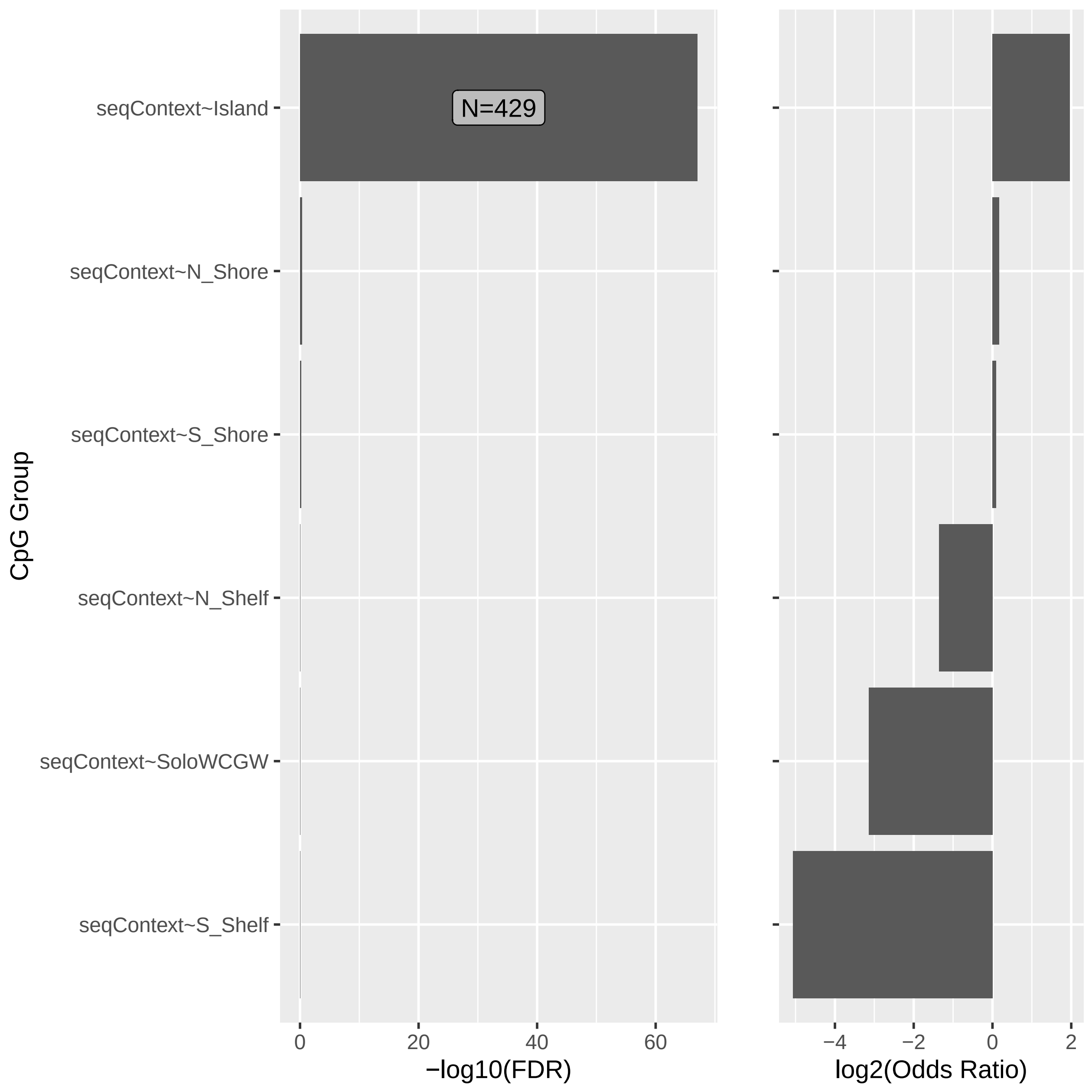

N=429
seqContext~Island
seqContext~N_Shore
seqContext~S_Shore
seqContext~N_Shelf
seqContext~SoloWCGW
seqContext~S_Shelf
CpG Group
0
20
40
60
−log10(FDR)
−4
−2
0
2
log2(Odds Ratio)

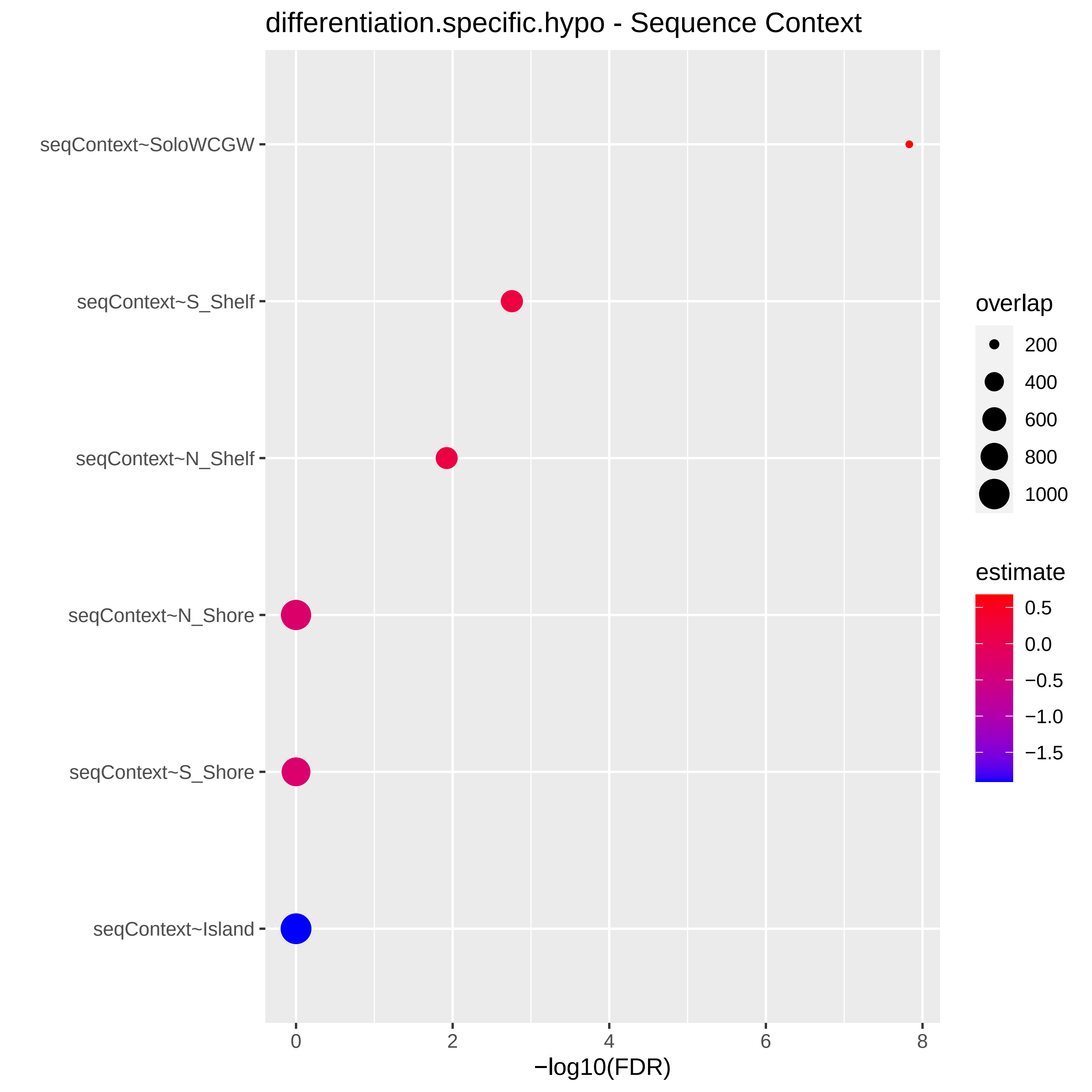
differentiation.specific.hypo - Sequence Context
seqContext~SoloWCGW
seqContext~S_Shelf
seqContext~N_Shelf
seqContext~N_Shore
seqContext~S_Shore
seqContext~Island
0
2
4
6
8
−log10(FDR)
overlap
200
400
600
800
1000
estimate
0.5
0.0
−0.5
−1.0
−1.5

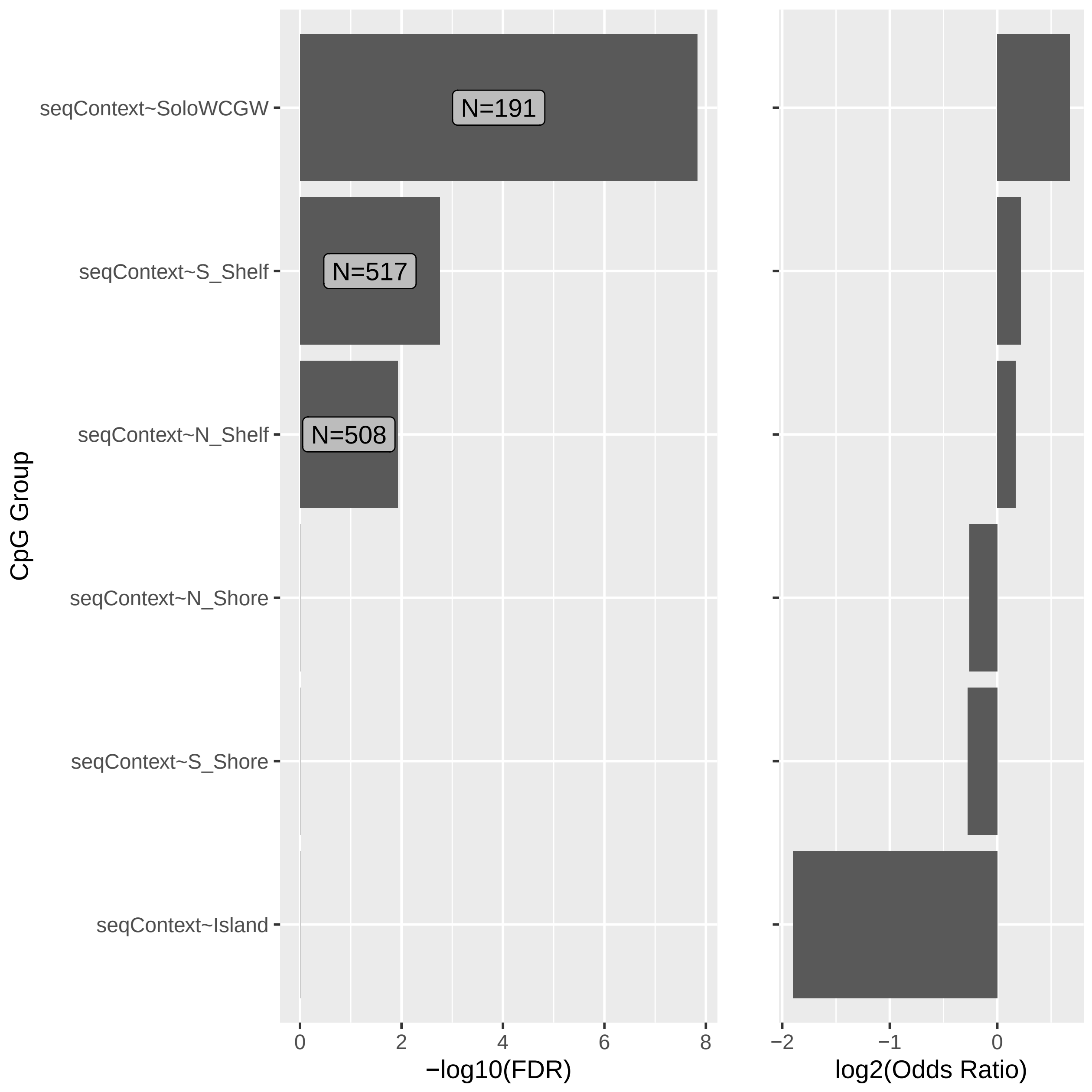

N=191
N=517
N=508
seqContext~SoloWCGW
seqContext~S_Shelf
seqContext~N_Shelf
seqContext~N_Shore
seqContext~S_Shore
seqContext~Island
CpG Group
0
2
4
6
8
−log10(FDR)
−2
−1
0
log2(Odds Ratio)

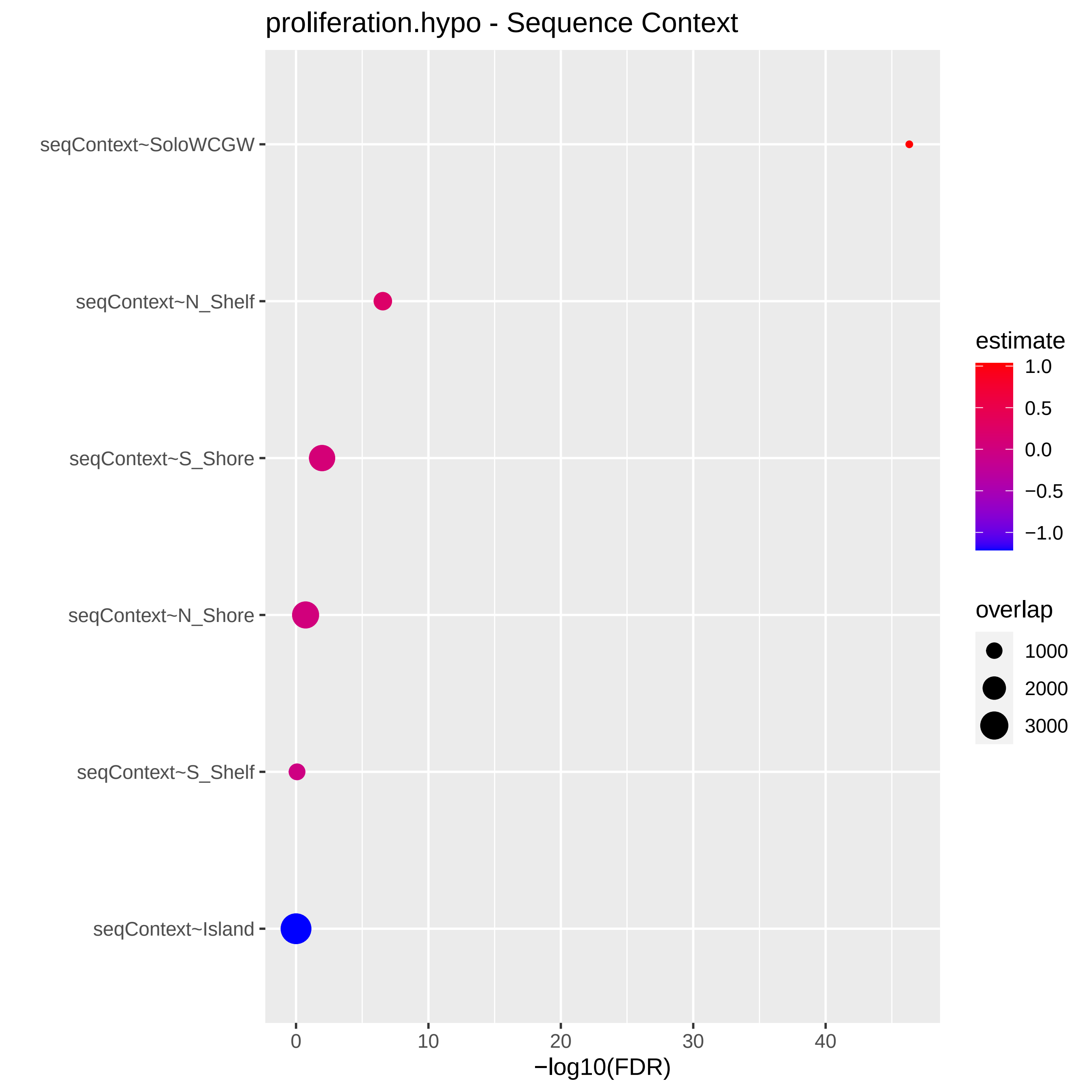

proliferation.hypo - Sequence Context
seqContext~SoloWCGW
seqContext~N_Shelf
seqContext~S_Shore
seqContext~N_Shore
seqContext~S_Shelf
seqContext~Island
0
10
20
30
40
−log10(FDR)
estimate
1.0
0.5
0.0
−0.5
−1.0
overlap
1000
2000
3000

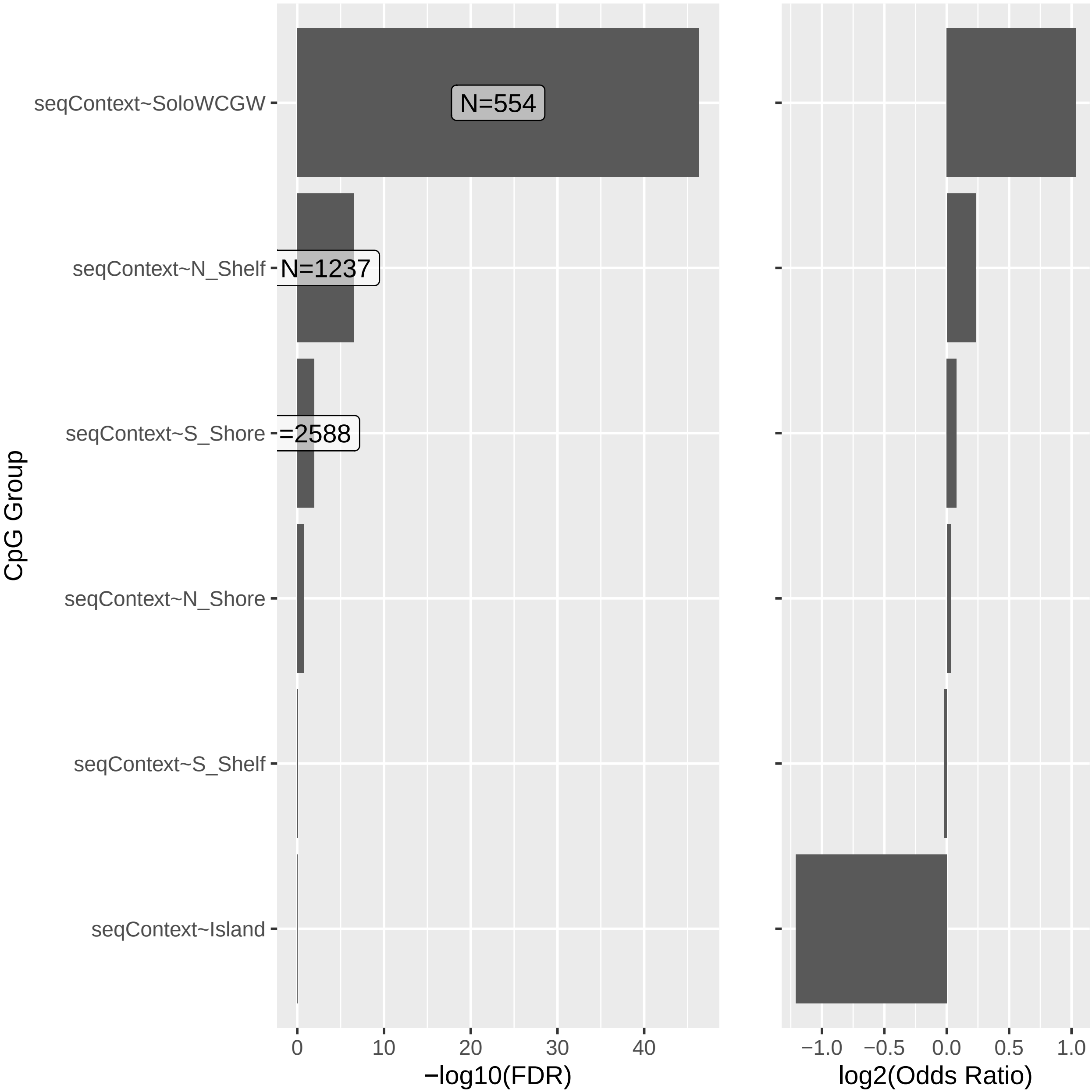
CpG Group
seqContext~SoloWCGW
seqContext~N_Shelf
seqContext~S_Shore
seqContext~N_Shore
seqContext~S_Shelf
seqContext~Island
N=554
N=1237
=2588
0
10
20
30
40
−log10(FDR)
−1.0
−0.5
0.0
0.5
1.0
log2(Odds Ratio)

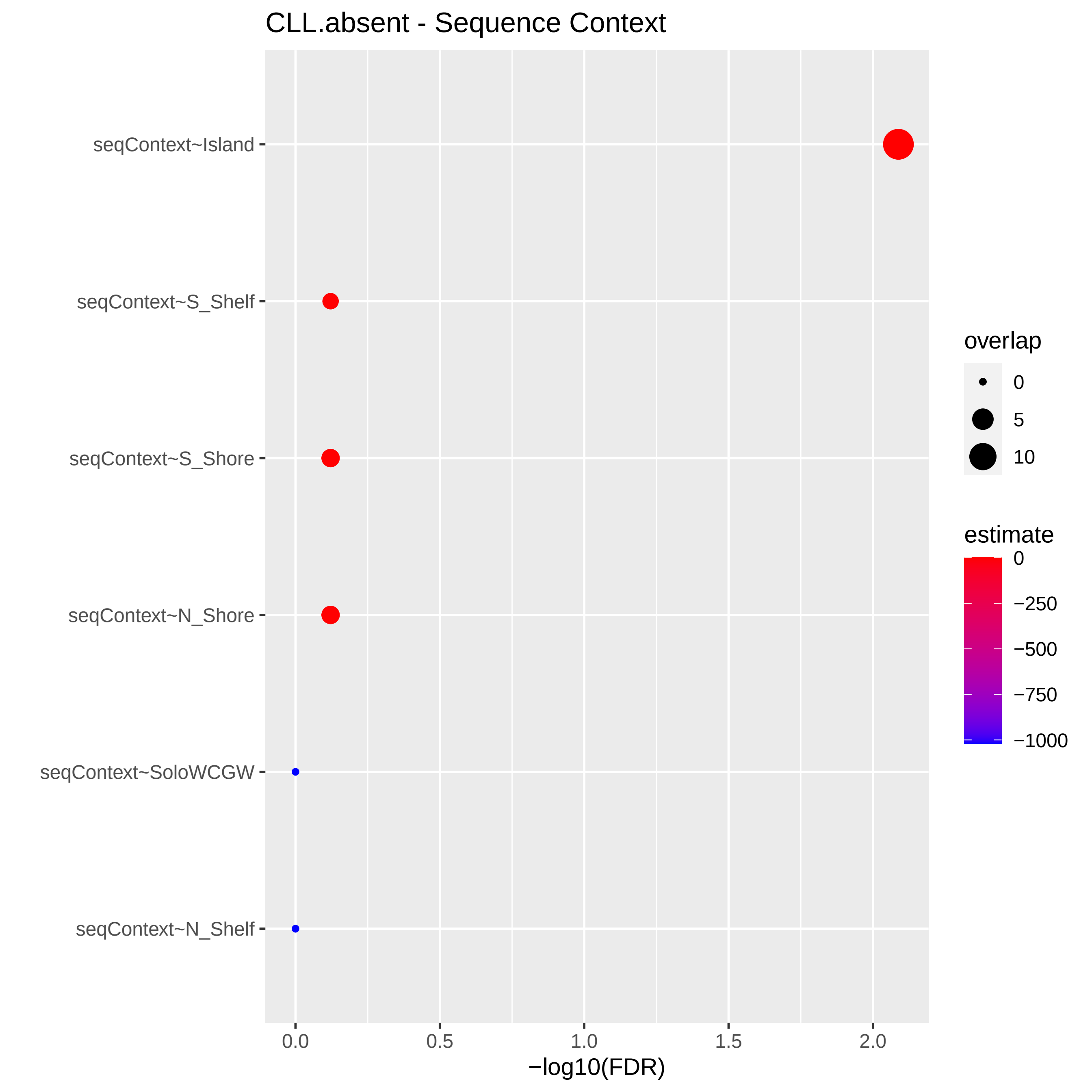

CLL.absent - Sequence Context
seqContext~Island
seqContext~S_Shelf
seqContext~S_Shore
seqContext~N_Shore
seqContext~SoloWCGW
seqContext~N_Shelf
0.0
0.5
1.0
1.5
2.0
−log10(FDR)
overlap
0
5
10
estimate
0
−250
−500
−750
−1000

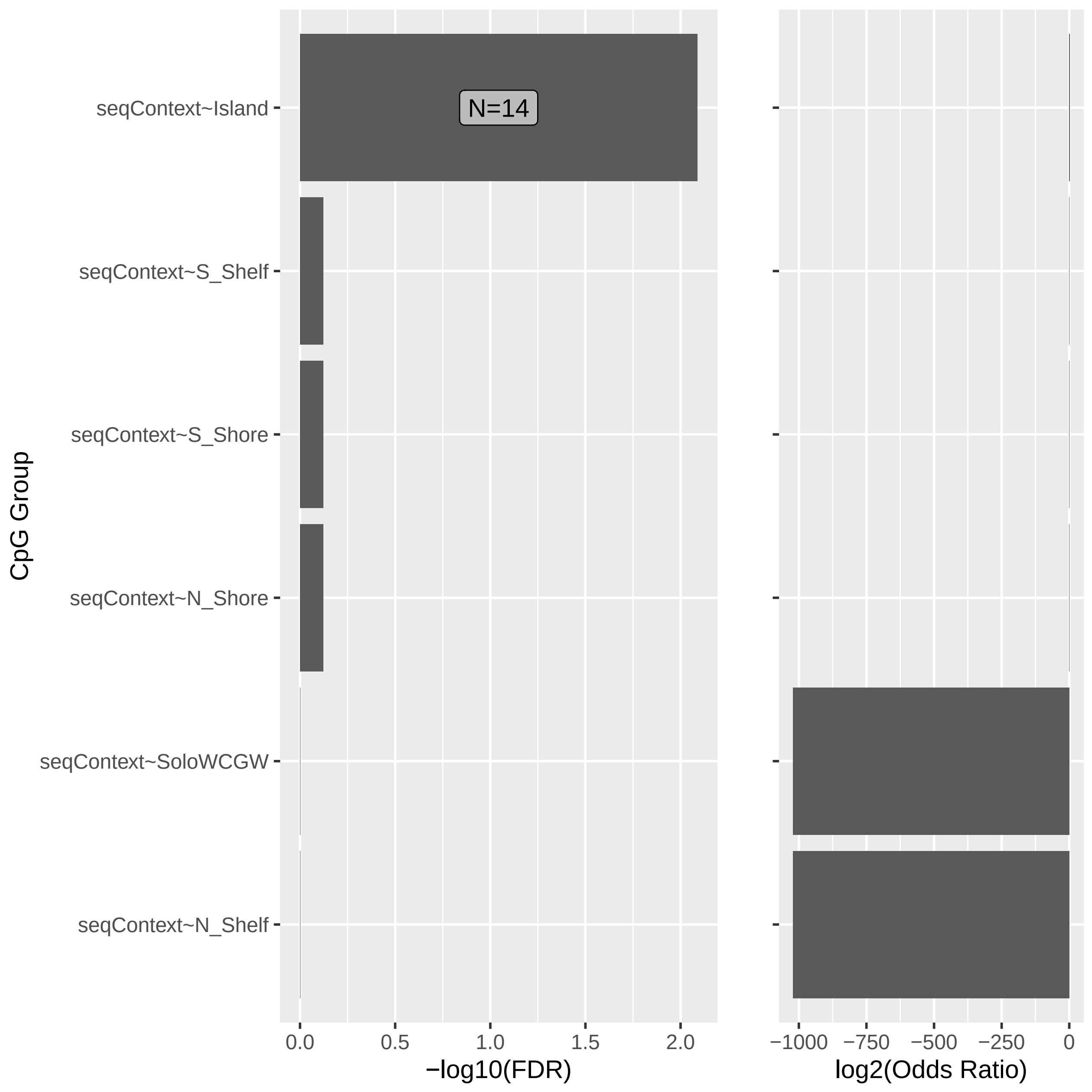

N=14
seqContext~Island
seqContext~S_Shelf
seqContext~S_Shore
seqContext~N_Shore
seqContext~SoloWCGW
seqContext~N_Shelf
CpG Group
0.0
0.5
1.0
1.5
2.0
−log10(FDR)
−1000
−750
−500
−250
0
log2(Odds Ratio)

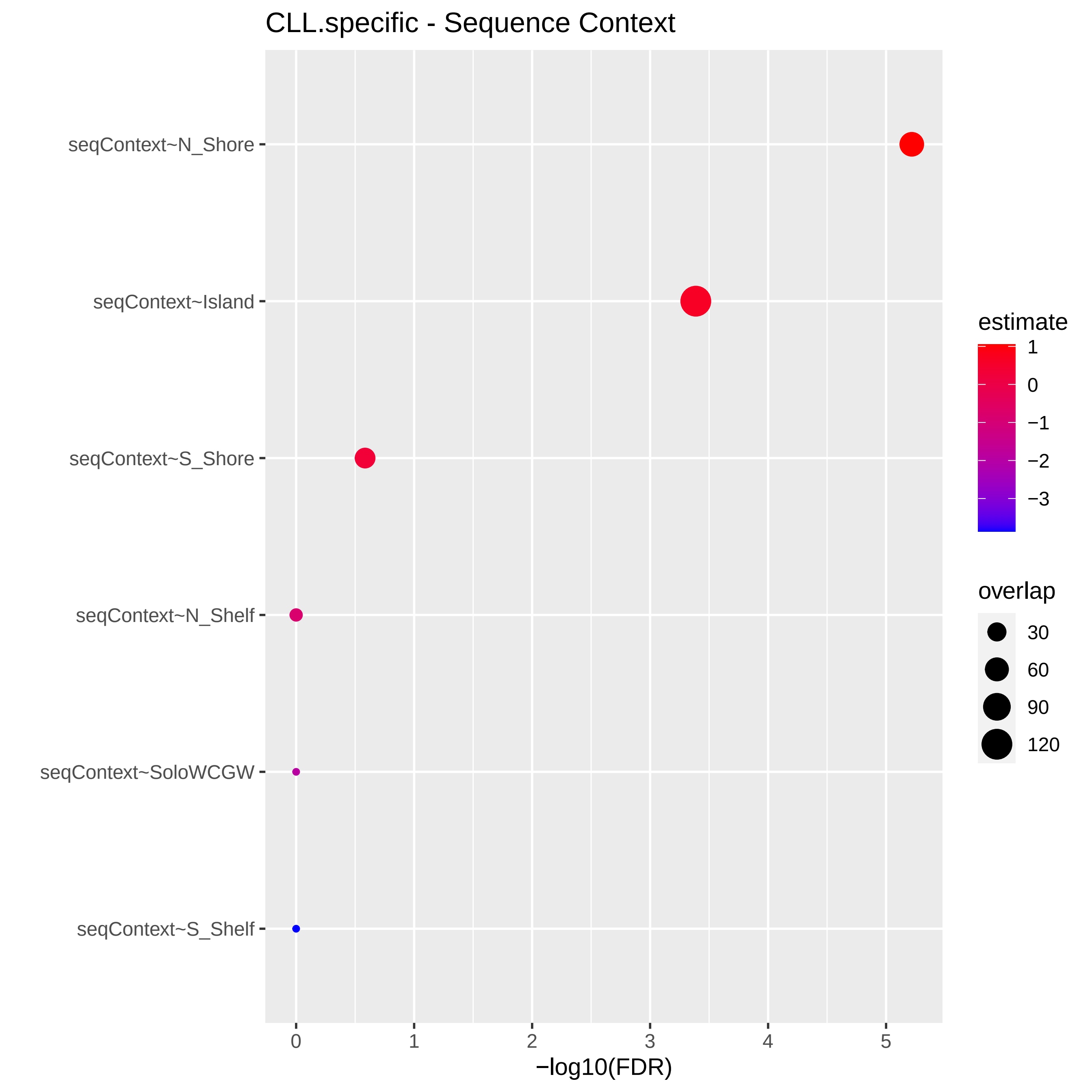

CLL.specific - Sequence Context
seqContext~N_Shore
seqContext~Island
seqContext~S_Shore
seqContext~N_Shelf
seqContext~SoloWCGW
seqContext~S_Shelf
0
1
2
3
4
5
−log10(FDR)
estimate
1
0
−1
−2
−3
overlap
30
60
90
120

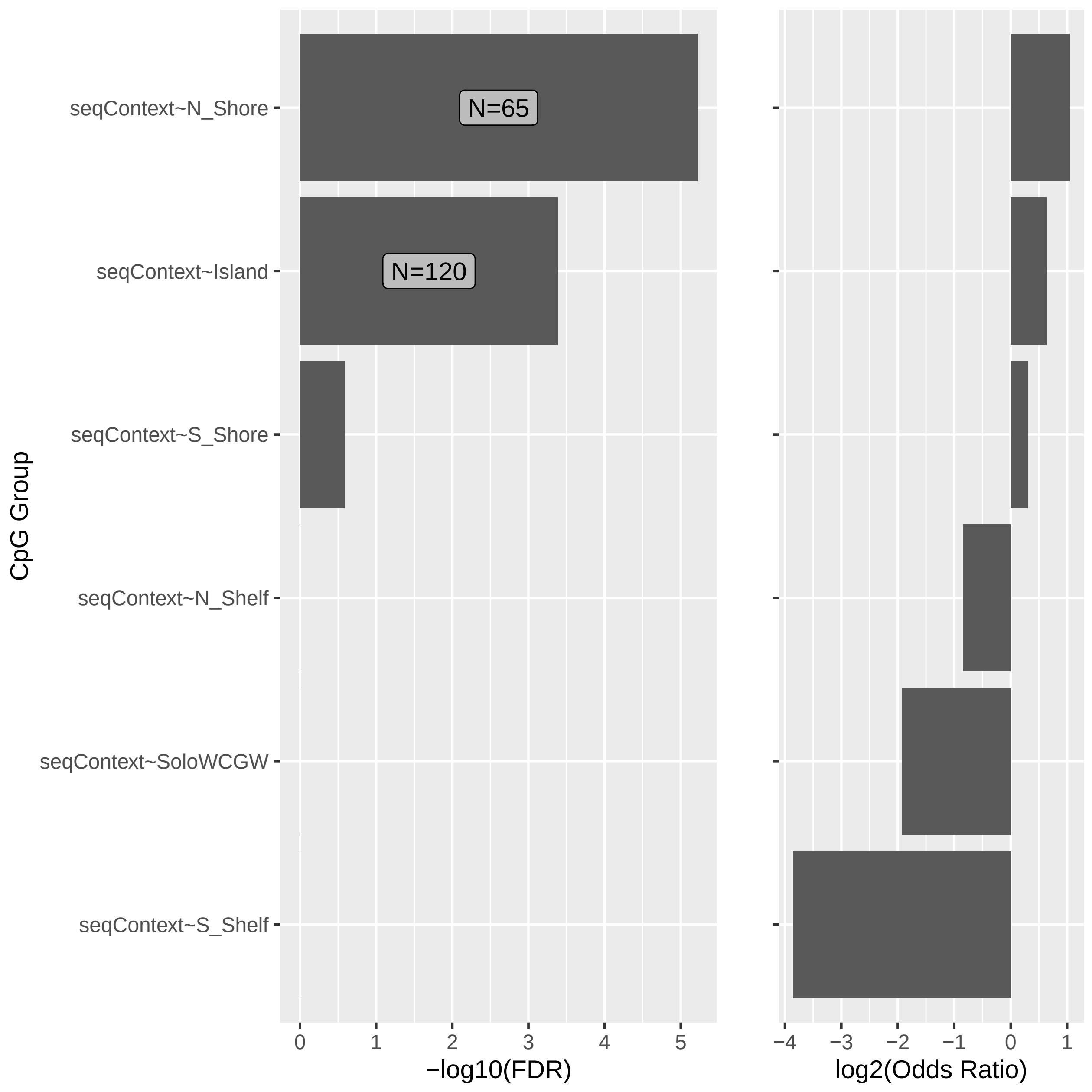

CpG Group
seqContext~N_Shore
seqContext~Island
seqContext~S_Shore
seqContext~N_Shelf
seqContext~SoloWCGW
seqContext~S_Shelf
N=65
N=120
0
1
2
3
4
5
−log10(FDR)
−4
−3
−2
−1
0
1
log2(Odds Ratio)

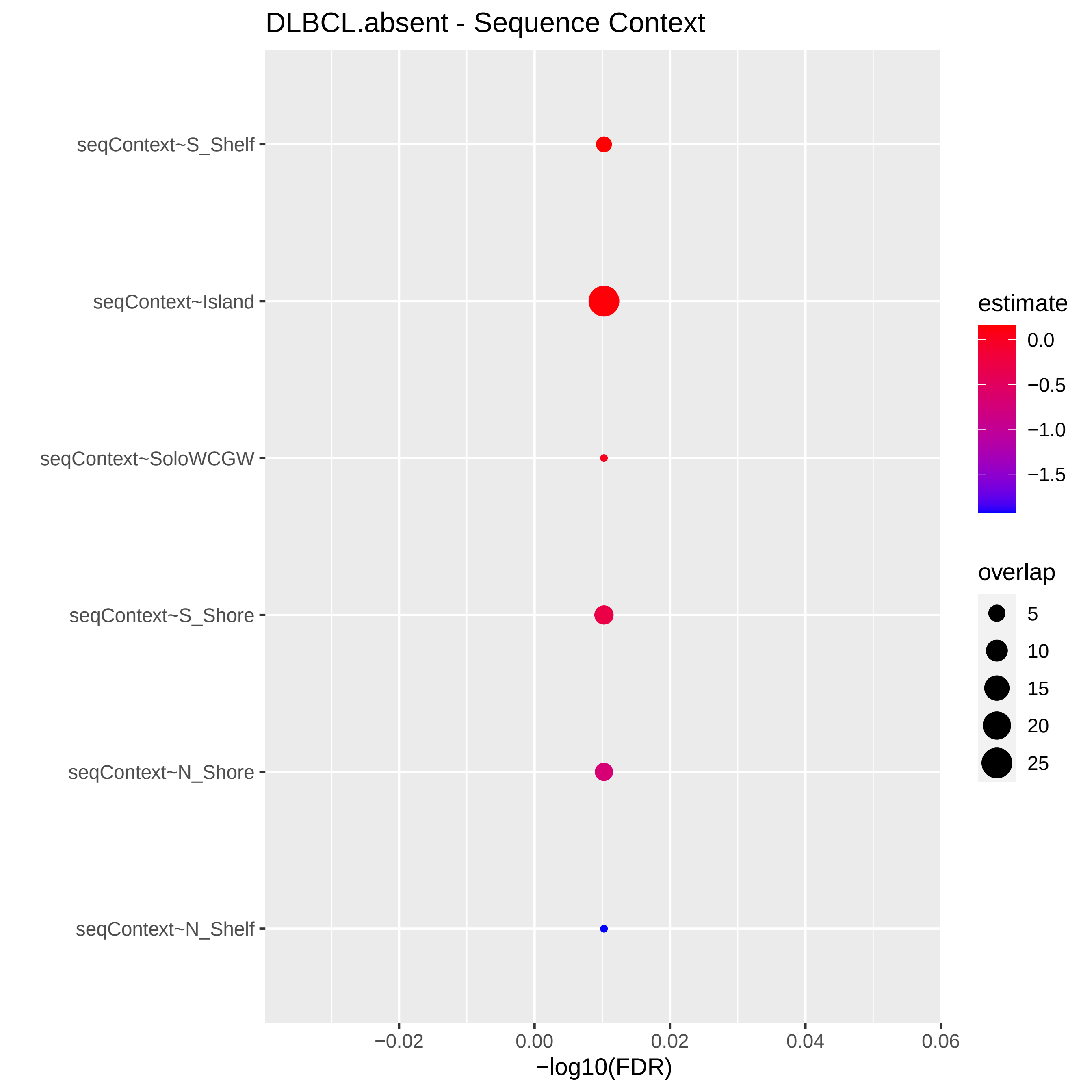

DLBCL.absent - Sequence Context
seqContext~S_Shelf
seqContext~Island
seqContext~SoloWCGW
seqContext~S_Shore
seqContext~N_Shore
seqContext~N_Shelf
−0.02
0.00
0.02
0.04
0.06
−log10(FDR)
estimate
0.0
−0.5
−1.0
−1.5
overlap
5
10
15
20
25

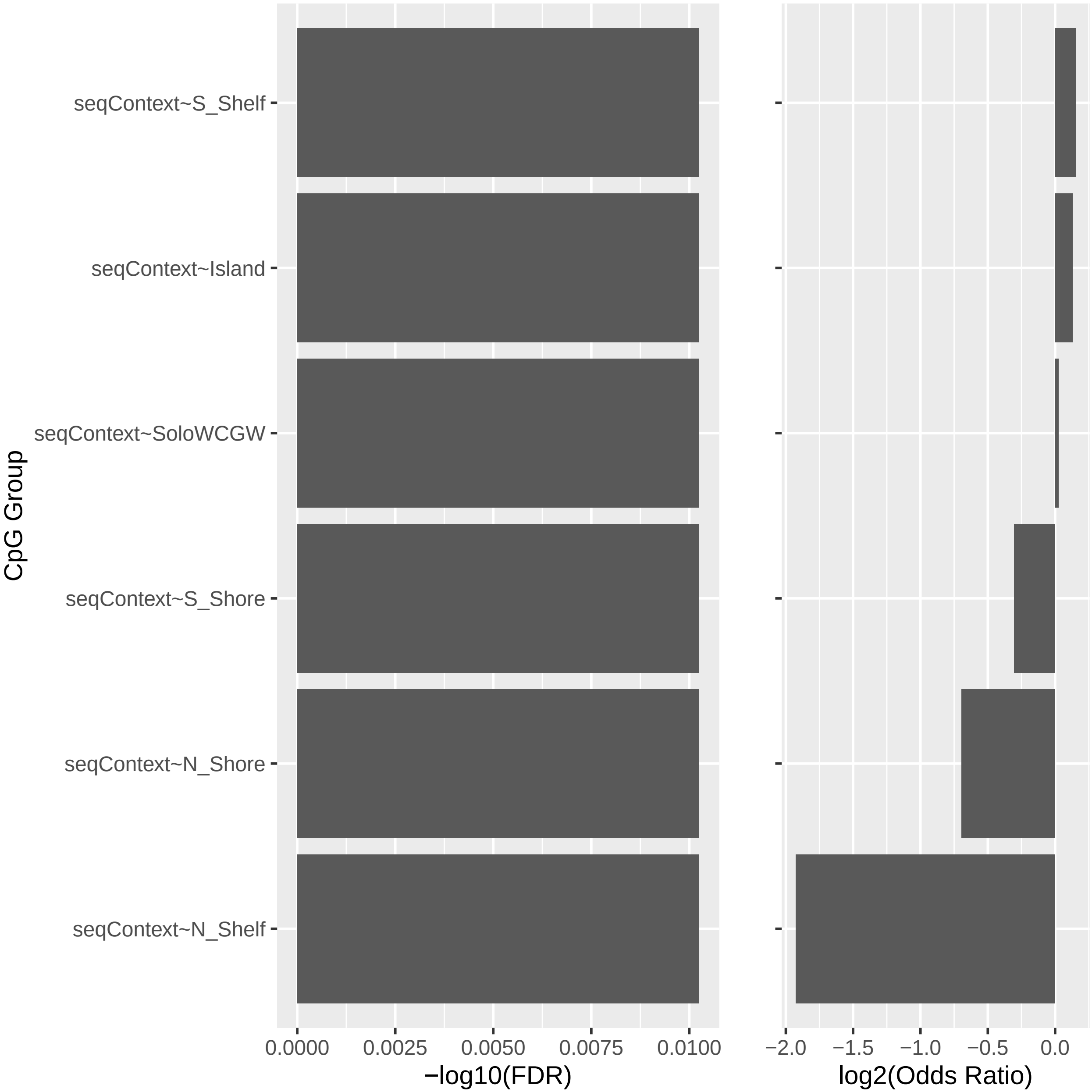

CpG Group
seqContext~S_Shelf
seqContext~Island
seqContext~SoloWCGW
seqContext~S_Shore
seqContext~N_Shore
seqContext~N_Shelf
0.0000
0.0025
0.0050
0.0075
0.0100
−log10(FDR)
−2.0
−1.5
−1.0
−0.5
0.0
log2(Odds Ratio)

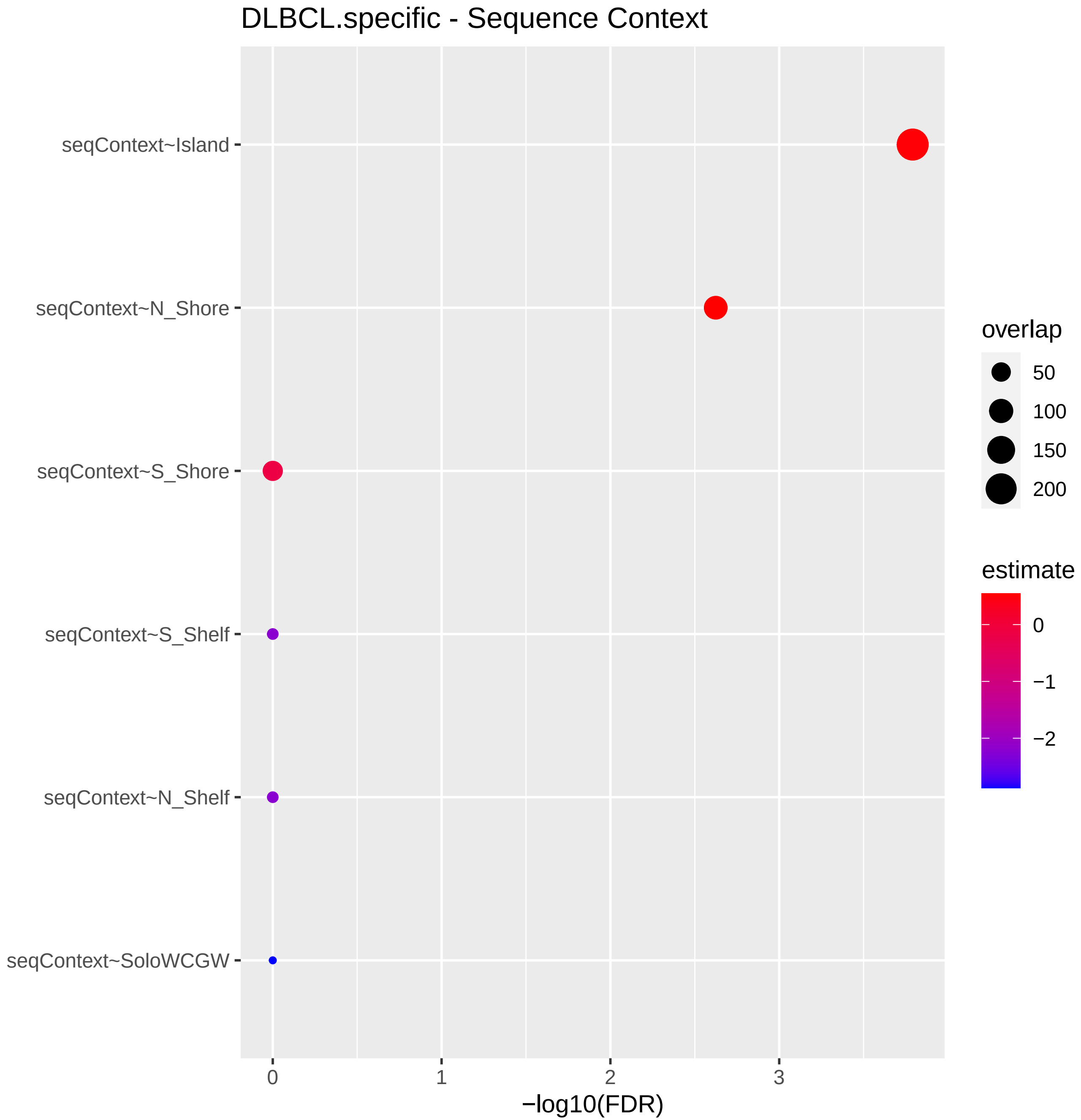

DLBCL.specific - Sequence Context
seqContext~Island
seqContext~N_Shore
seqContext~S_Shore
seqContext~S_Shelf
seqContext~N_Shelf
seqContext~SoloWCGW
0
1
2
3
−log10(FDR)
overlap
50
100
150
200
estimate
0
−1
−2

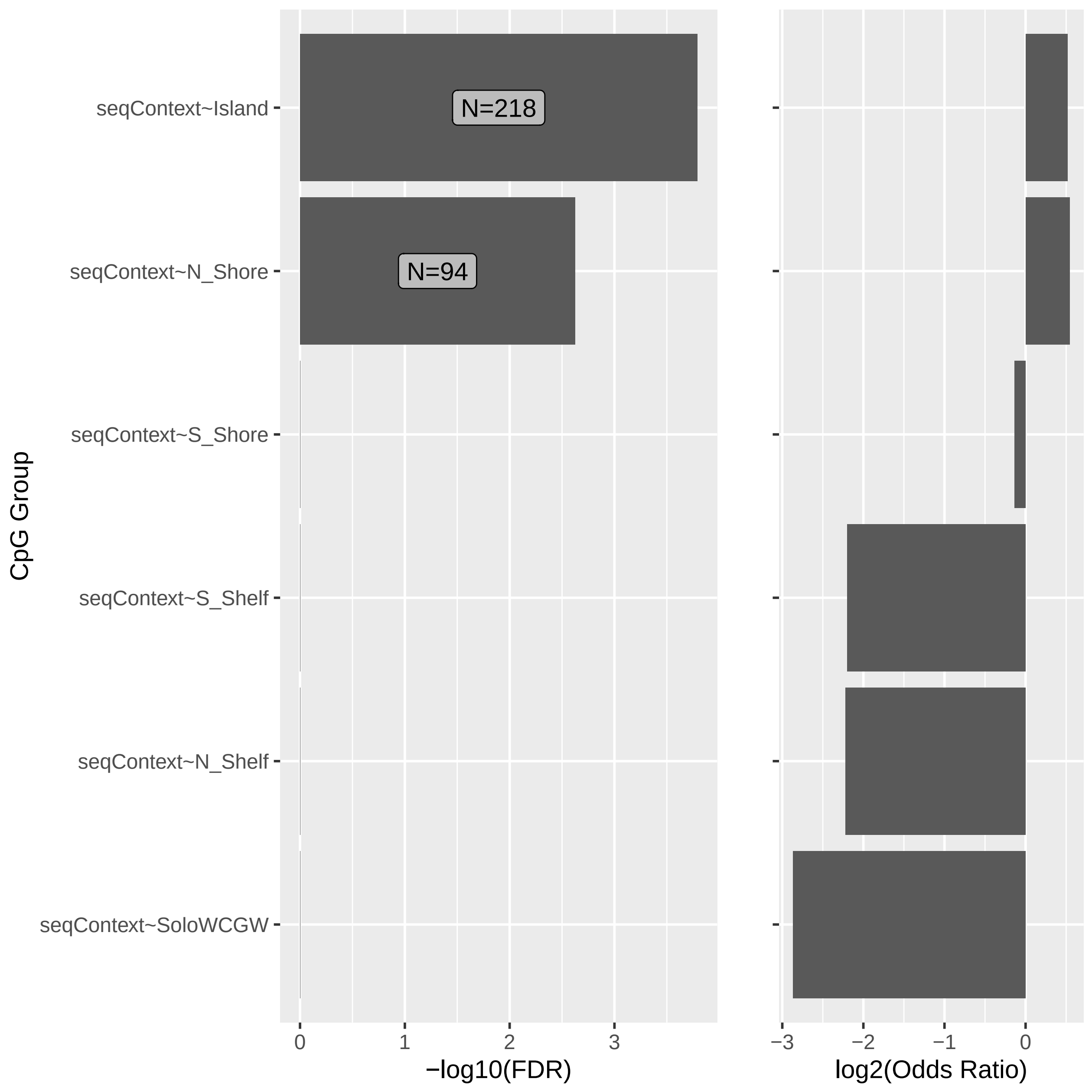

CpG Group
seqContext~Island
seqContext~N_Shore
seqContext~S_Shore
seqContext~S_Shelf
seqContext~N_Shelf
seqContext~SoloWCGW
N=218
N=94
0
1
2
3
−log10(FDR)
−3
−2
−1
0
log2(Odds Ratio)

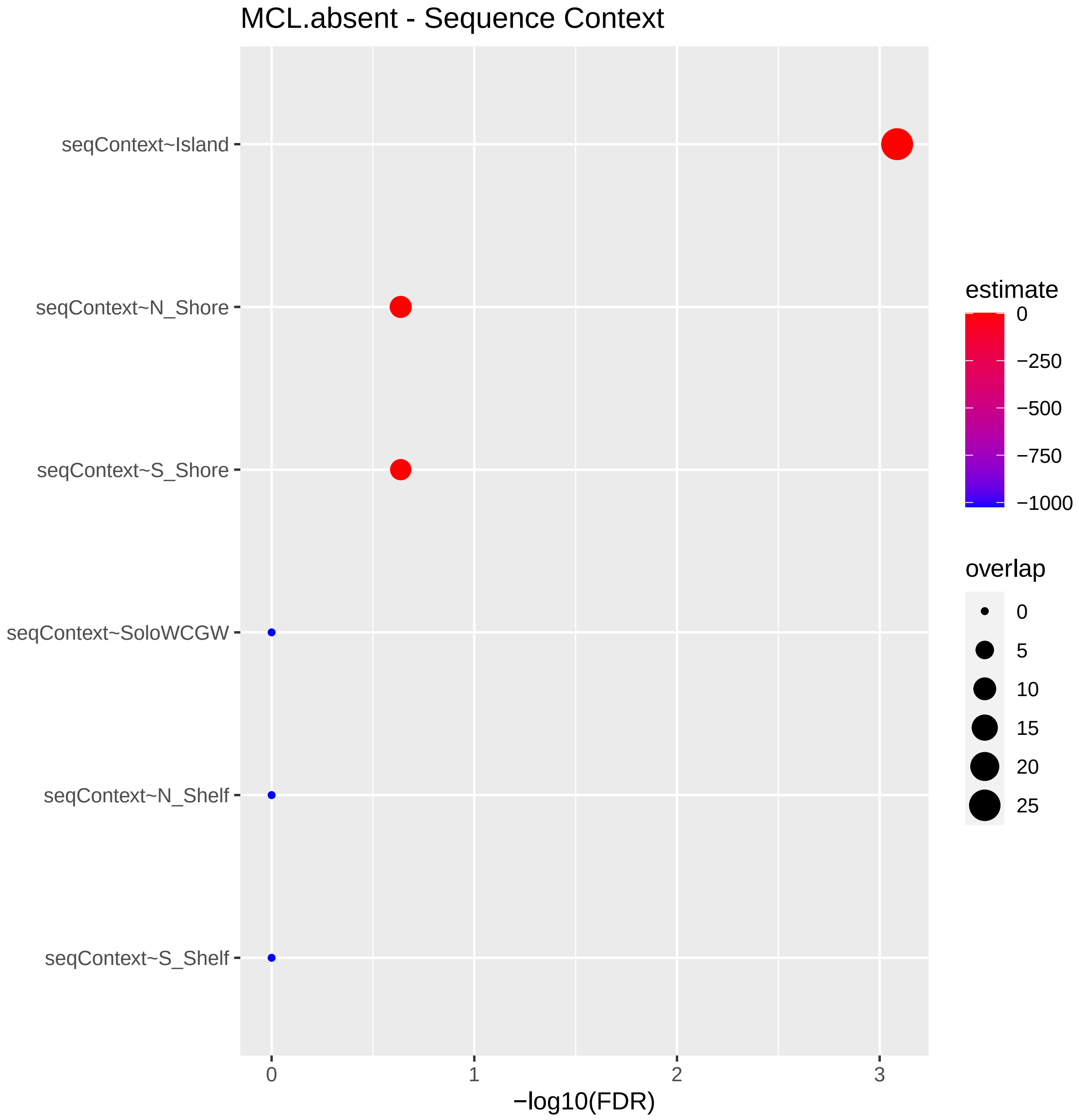

MCL.absent - Sequence Context
seqContext~Island
seqContext~N_Shore
seqContext~S_Shore
seqContext~SoloWCGW
seqContext~N_Shelf
seqContext~S_Shelf
0
1
2
3
−log10(FDR)
estimate
0
−250
−500
−750
−1000
overlap
0
5
10
15
20
25

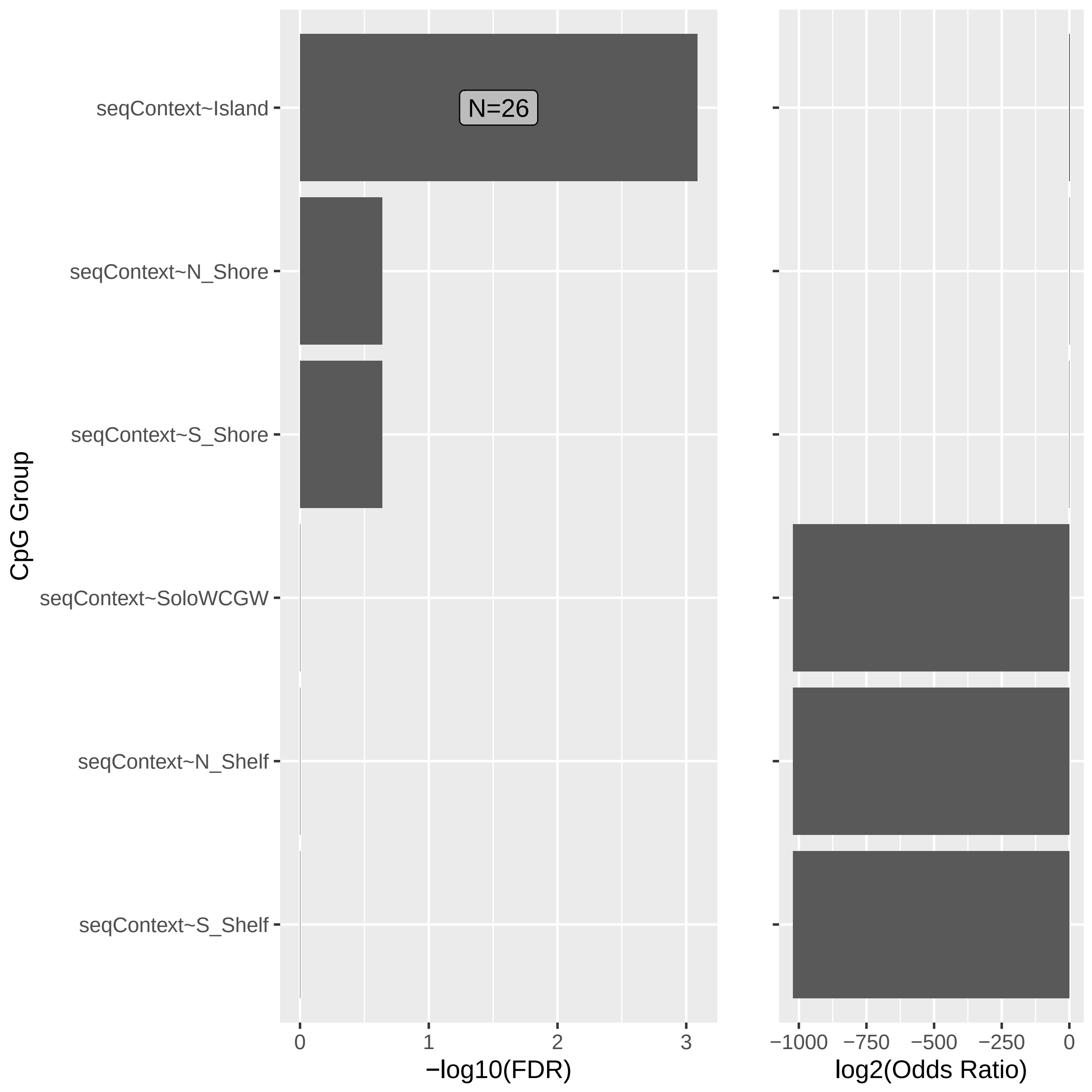

seqContext~Island
seqContext~N_Shore
seqContext~S_Shore
seqContext~SoloWCGW
seqContext~N_Shelf
seqContext~S_Shelf
CpG Group
N=26
0
1
2
3
−log10(FDR)
−1000
−750
−500
−250
0
log2(Odds Ratio)

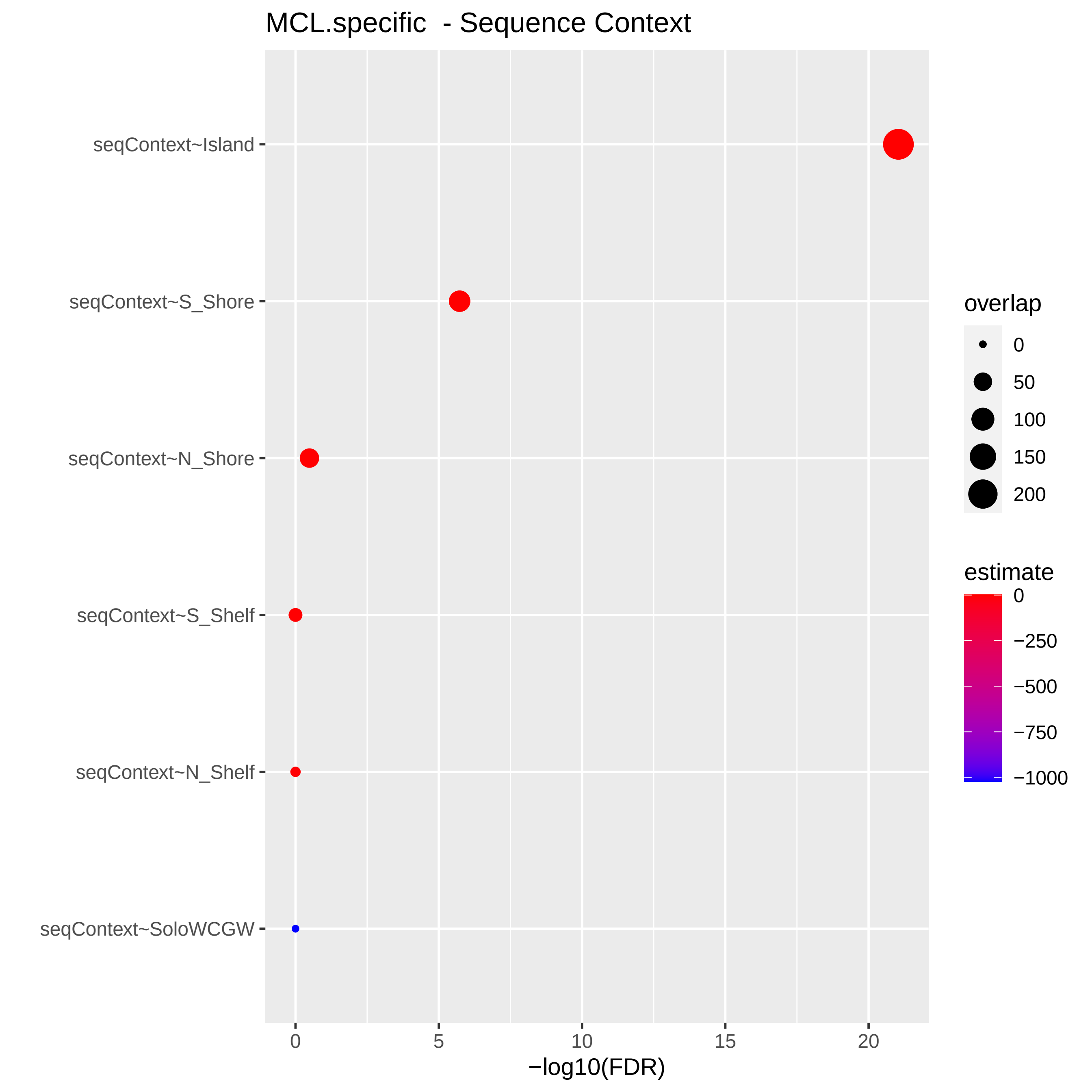
MCL.specific - Sequence Context
seqContext~Island
seqContext~S_Shore
seqContext~N_Shore
seqContext~S_Shelf
seqContext~N_Shelf
seqContext~SoloWCGW
0
5
10
15
20
−log10(FDR)
overlap
0
50
100
150
200
estimate
0
−250
−500
−750
−1000

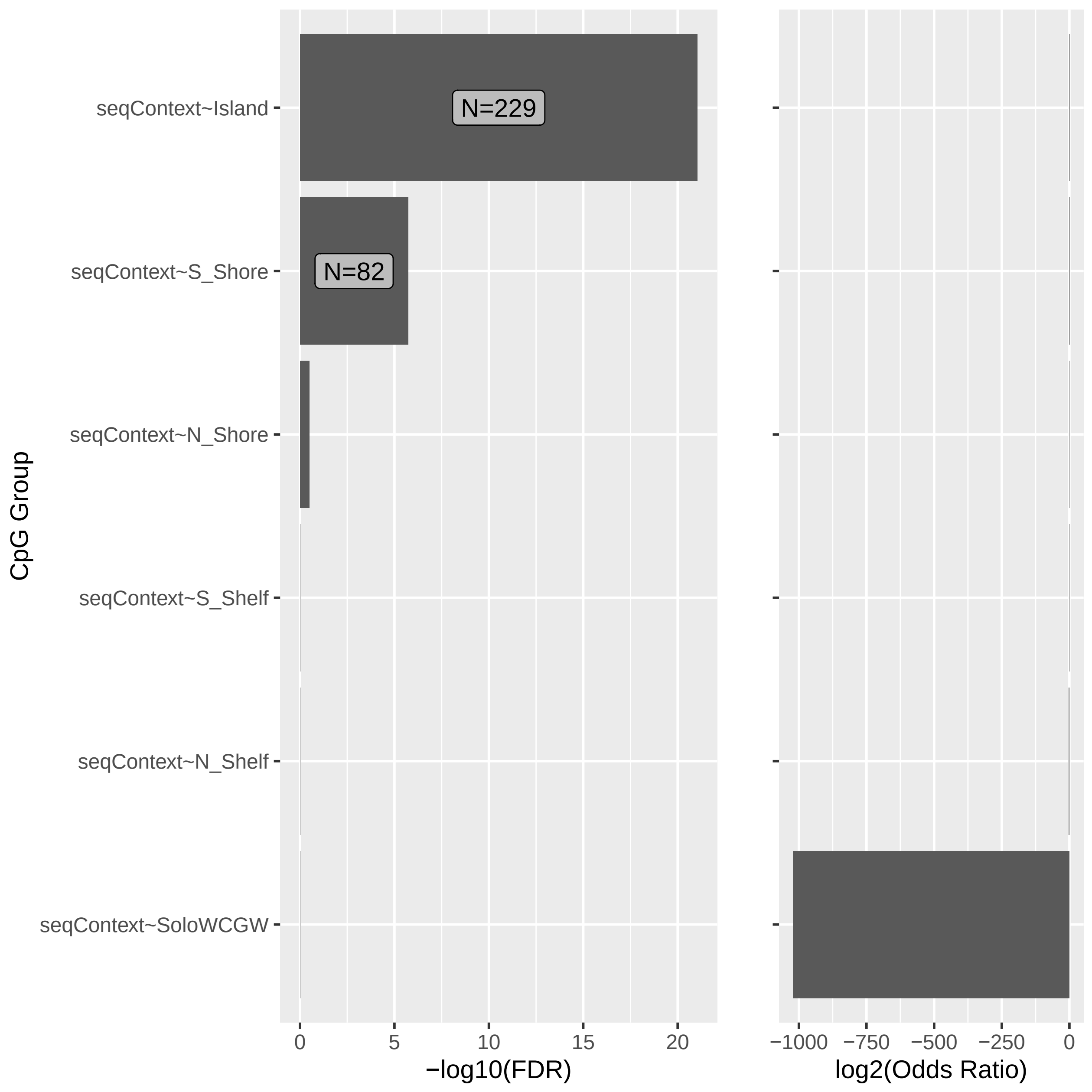

CpG Group
seqContext~Island
seqContext~S_Shore
seqContext~N_Shore
seqContext~S_Shelf
seqContext~N_Shelf
seqContext~SoloWCGW
N=229
N=82
0
5
10
15
20
−log10(FDR)
−1000
−750
−500
−250
0
log2(Odds Ratio)

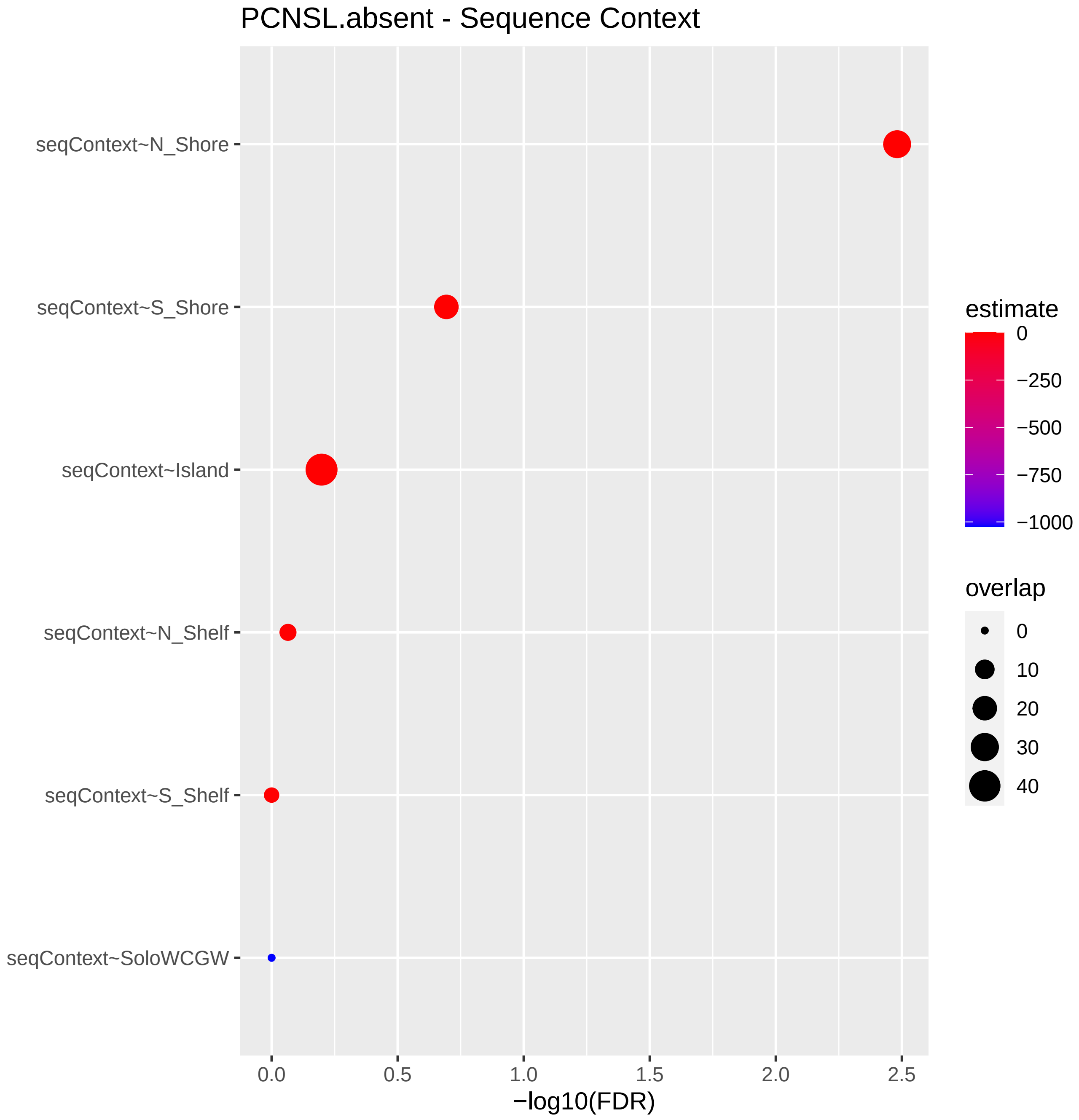
PCNSL.absent - Sequence Context
seqContext~N_Shore
seqContext~S_Shore
seqContext~Island
seqContext~N_Shelf
seqContext~S_Shelf
seqContext~SoloWCGW
0.0
0.5
1.0
1.5
2.0
2.5
−log10(FDR)
estimate
0
−250
−500
−750
−1000
overlap
0
10
20
30
40

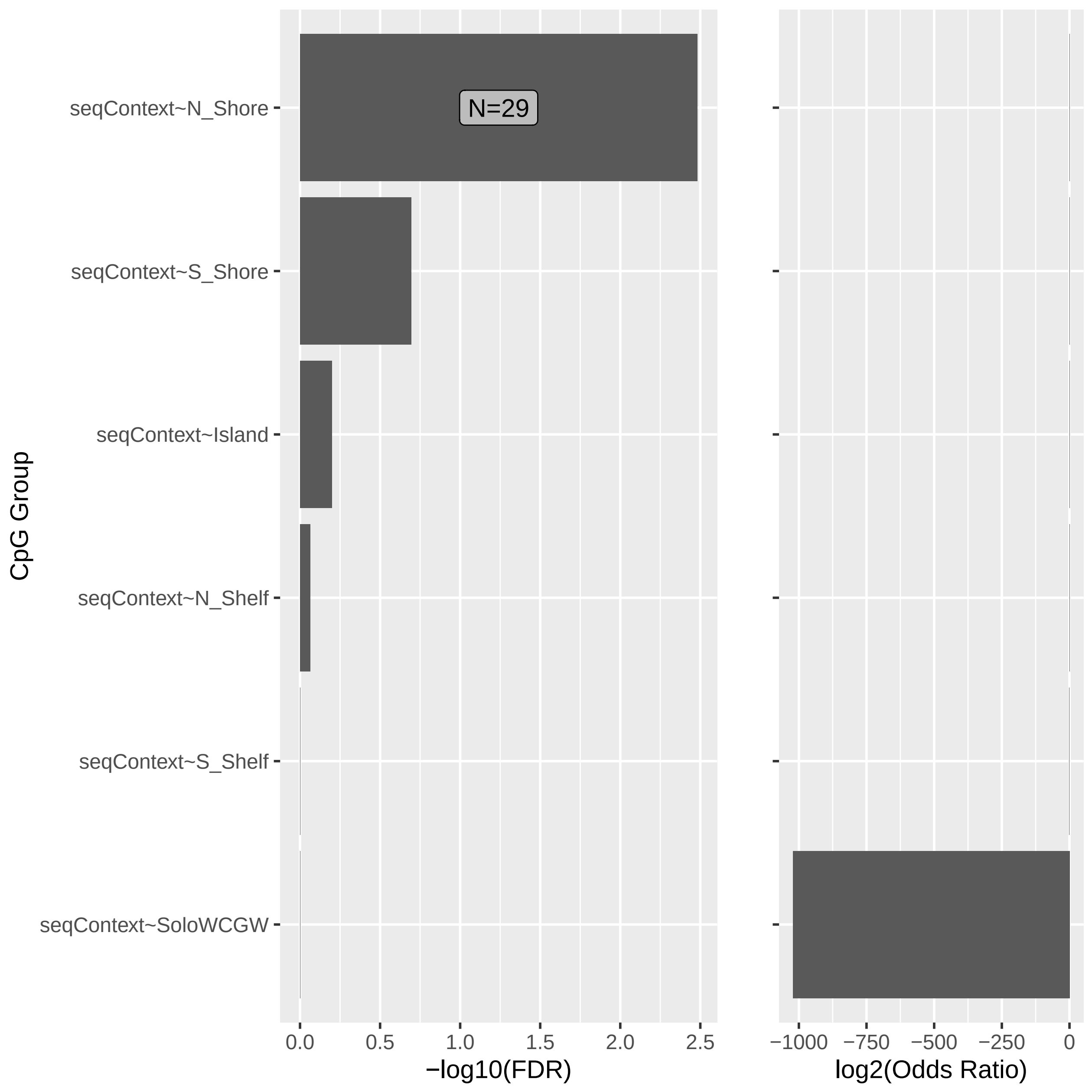

CpG Group
seqContext~N_Shore
seqContext~S_Shore
seqContext~Island
seqContext~N_Shelf
seqContext~S_Shelf
seqContext~SoloWCGW
N=29
0.0
0.5
1.0
1.5
2.0
2.5
−log10(FDR)
−1000
−750
−500
−250
0
log2(Odds Ratio)

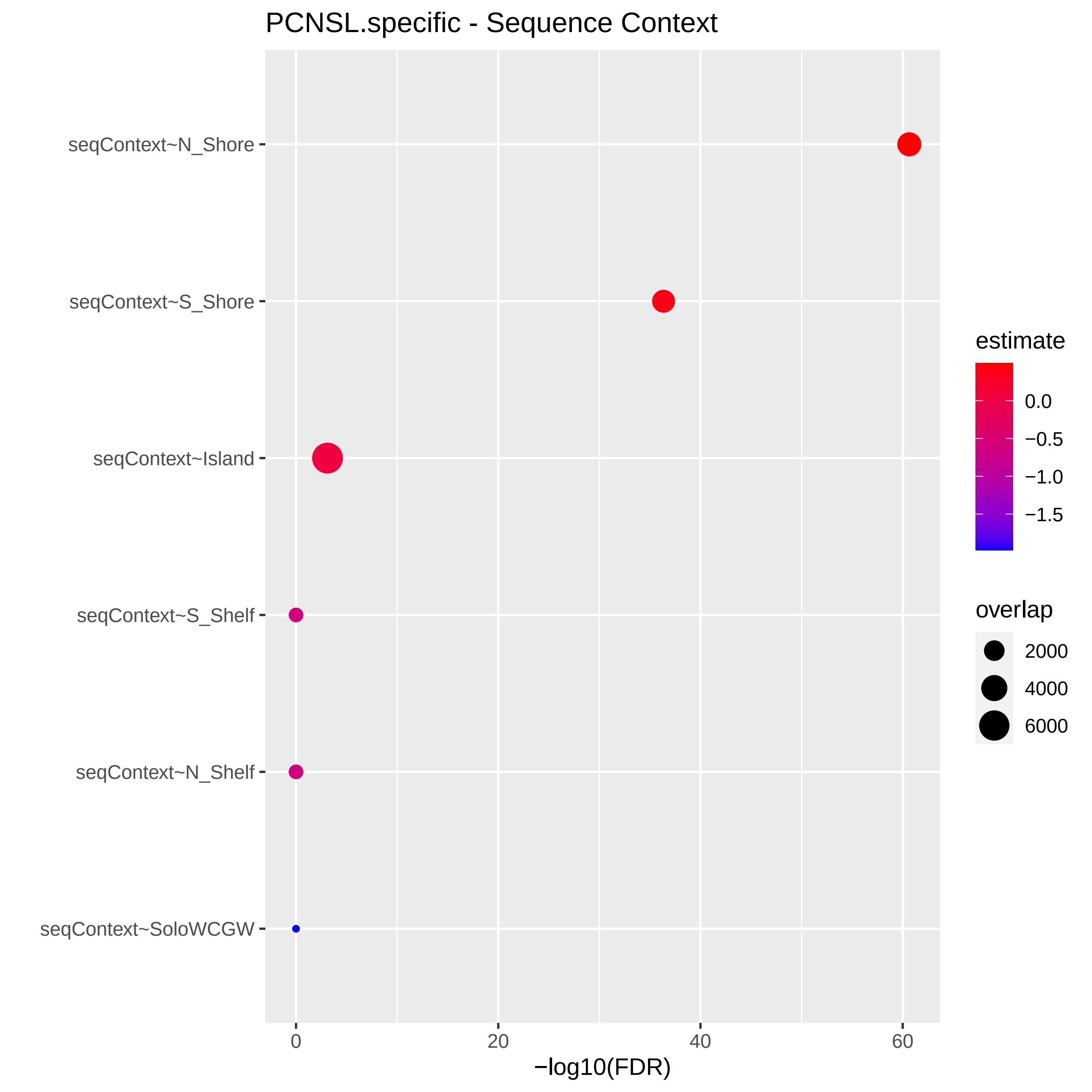
PCNSL.specific - Sequence Context
seqContext~N_Shore
seqContext~S_Shore
seqContext~Island
seqContext~S_Shelf
seqContext~N_Shelf
seqContext~SoloWCGW
0
20
40
60
−log10(FDR)
estimate
0.0
−0.5
−1.0
−1.5
overlap
2000
4000
6000

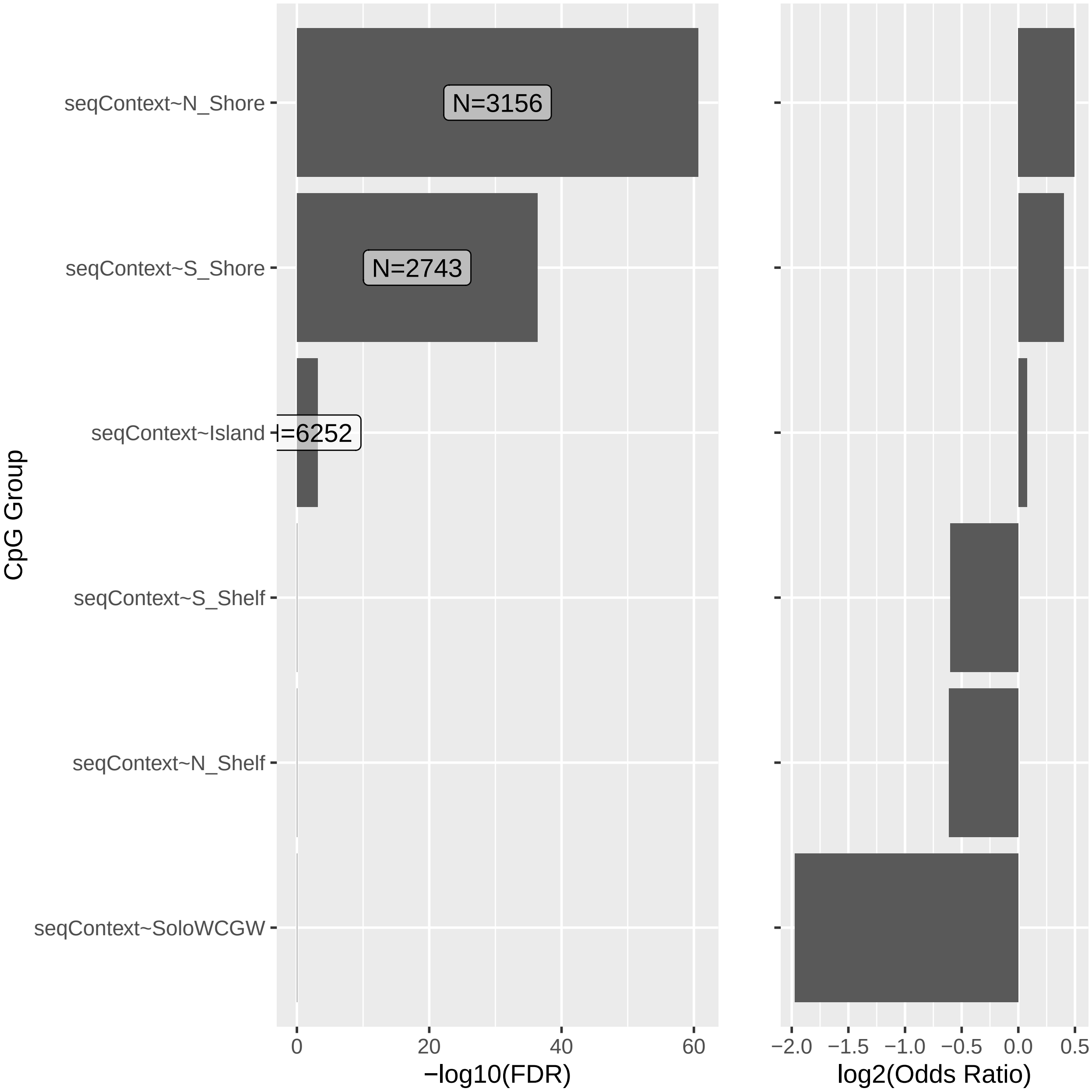

CpG Group
seqContext~N_Shore
seqContext~S_Shore
seqContext~Island
seqContext~S_Shelf
seqContext~N_Shelf
seqContext~SoloWCGW
N=3156
N=2743
=6252
0
20
40
60
−log10(FDR)
−2.0
−1.5
−1.0
−0.5
0.0
0.5
log2(Odds Ratio)

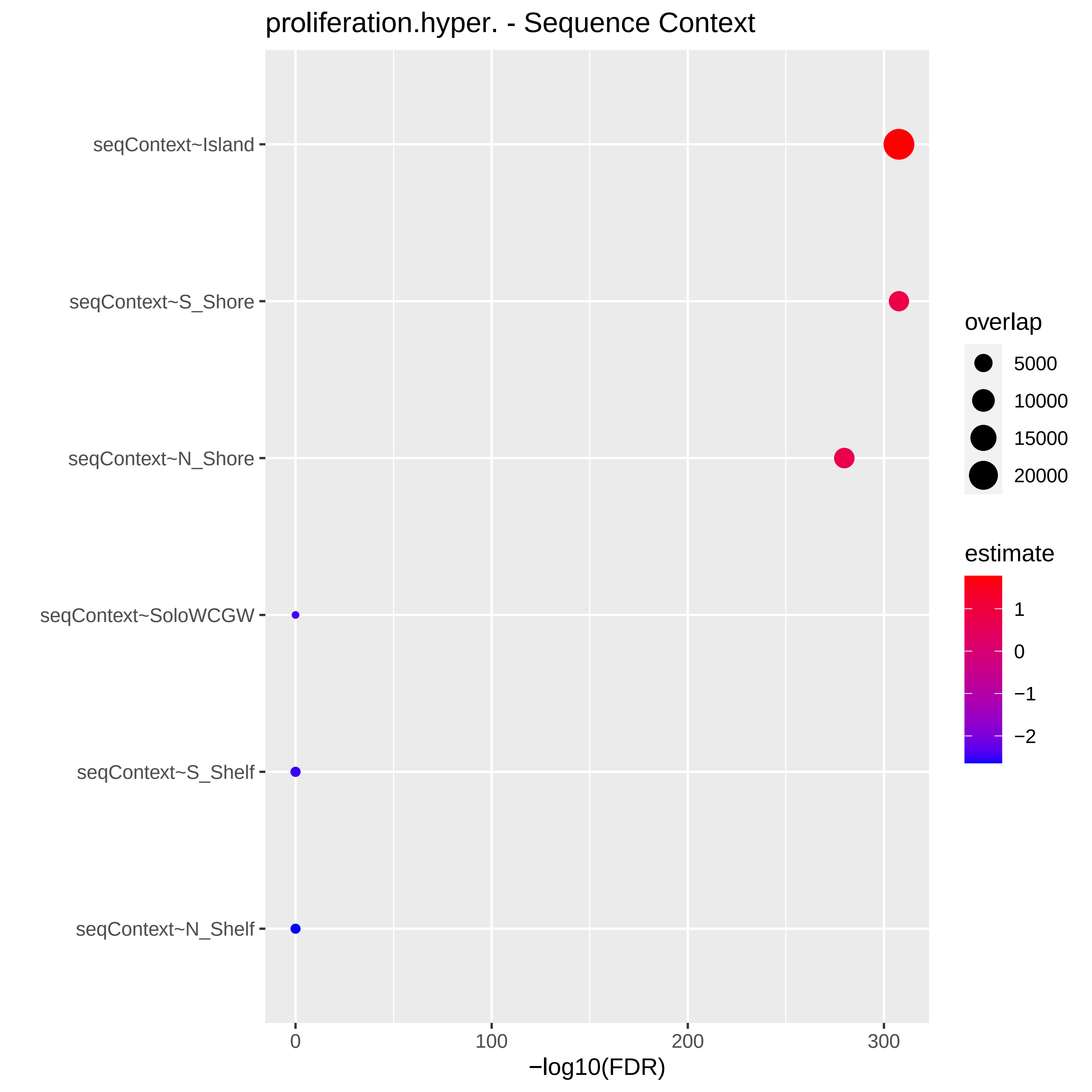
proliferation.hyper. - Sequence Context
seqContext~Island
seqContext~S_Shore
seqContext~N_Shore
seqContext~SoloWCGW
seqContext~S_Shelf
seqContext~N_Shelf
0
100
200
300
−log10(FDR)
overlap
5000
10000
15000
20000
estimate
1
0
−1
−2

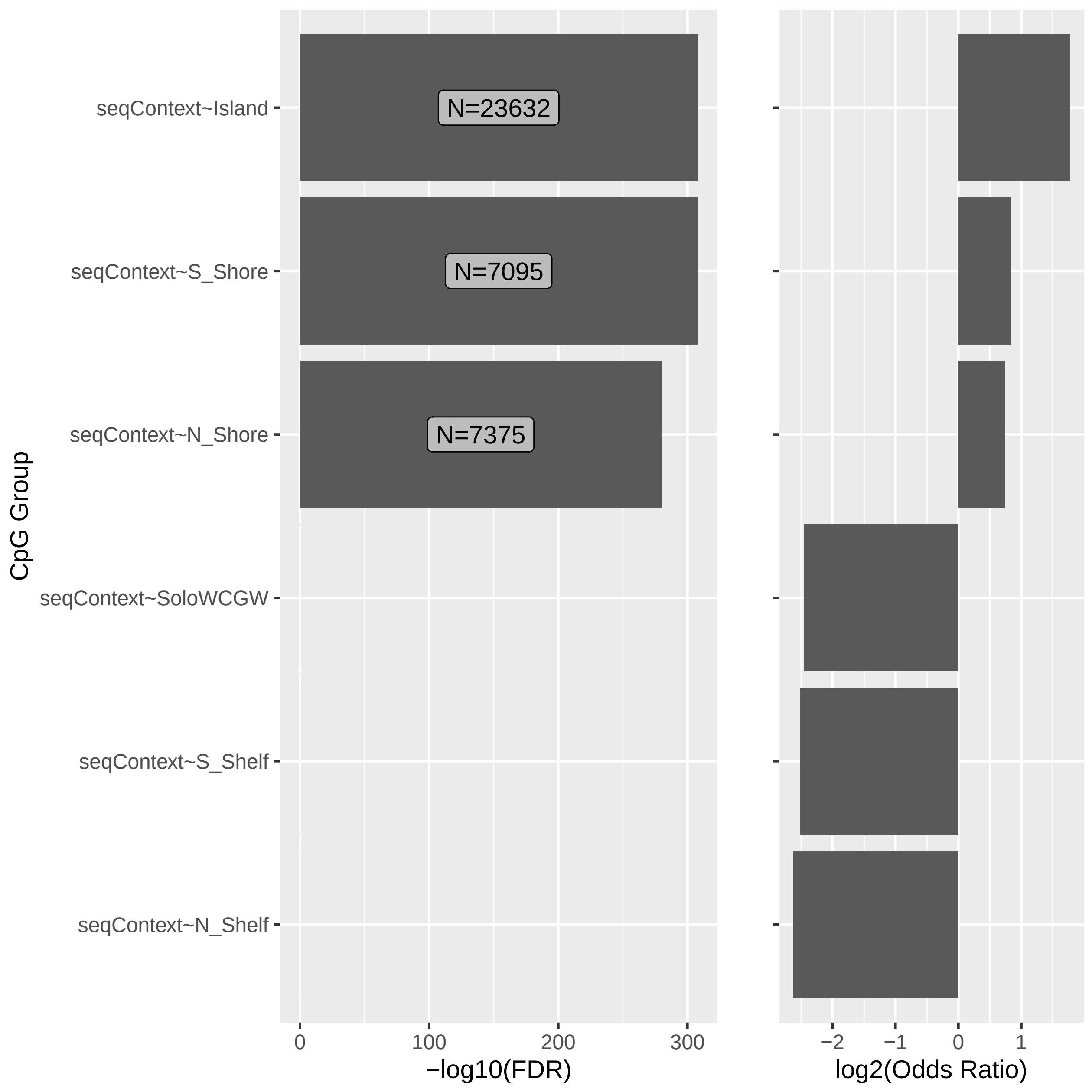

CpG Group
seqContext~Island
seqContext~S_Shore
seqContext~N_Shore
seqContext~SoloWCGW
seqContext~S_Shelf
seqContext~N_Shelf
N=23632
N=7095
N=7375
0
100
200
300
−log10(FDR)
−2
−1
0
1
log2(Odds Ratio)

Supplementary figure 2. Detailed results of SeSAMe analysis for sequence context for the different DMR groups, as indicated. For each DMR group the first graph shows the significance of the association with the specific sequence context adjusted for false discovery rate (−log10(FDR)). The second also includes details of the number of overlapping DMRs overlapping with the specific sequence context feature.

Supplementary Figure 3

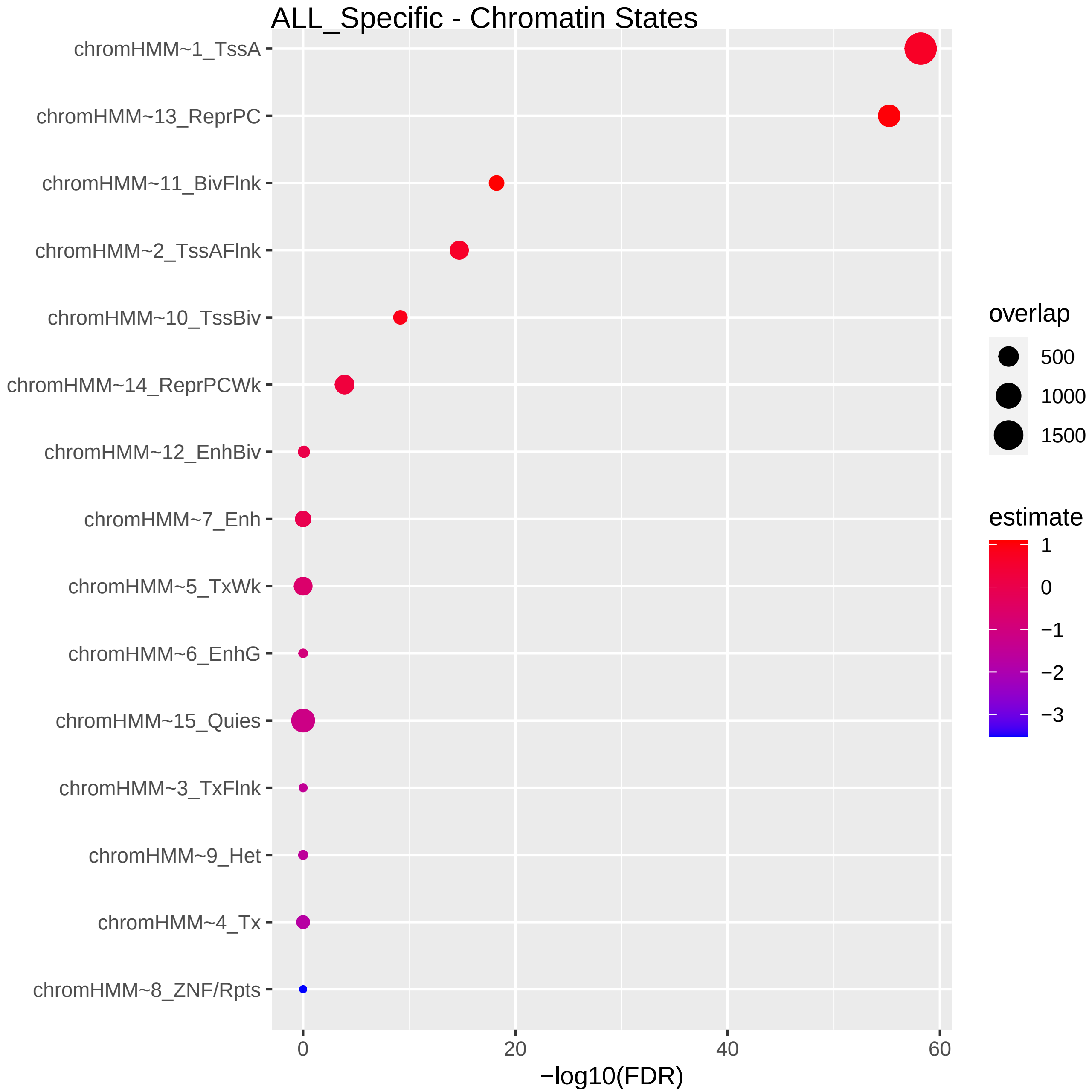

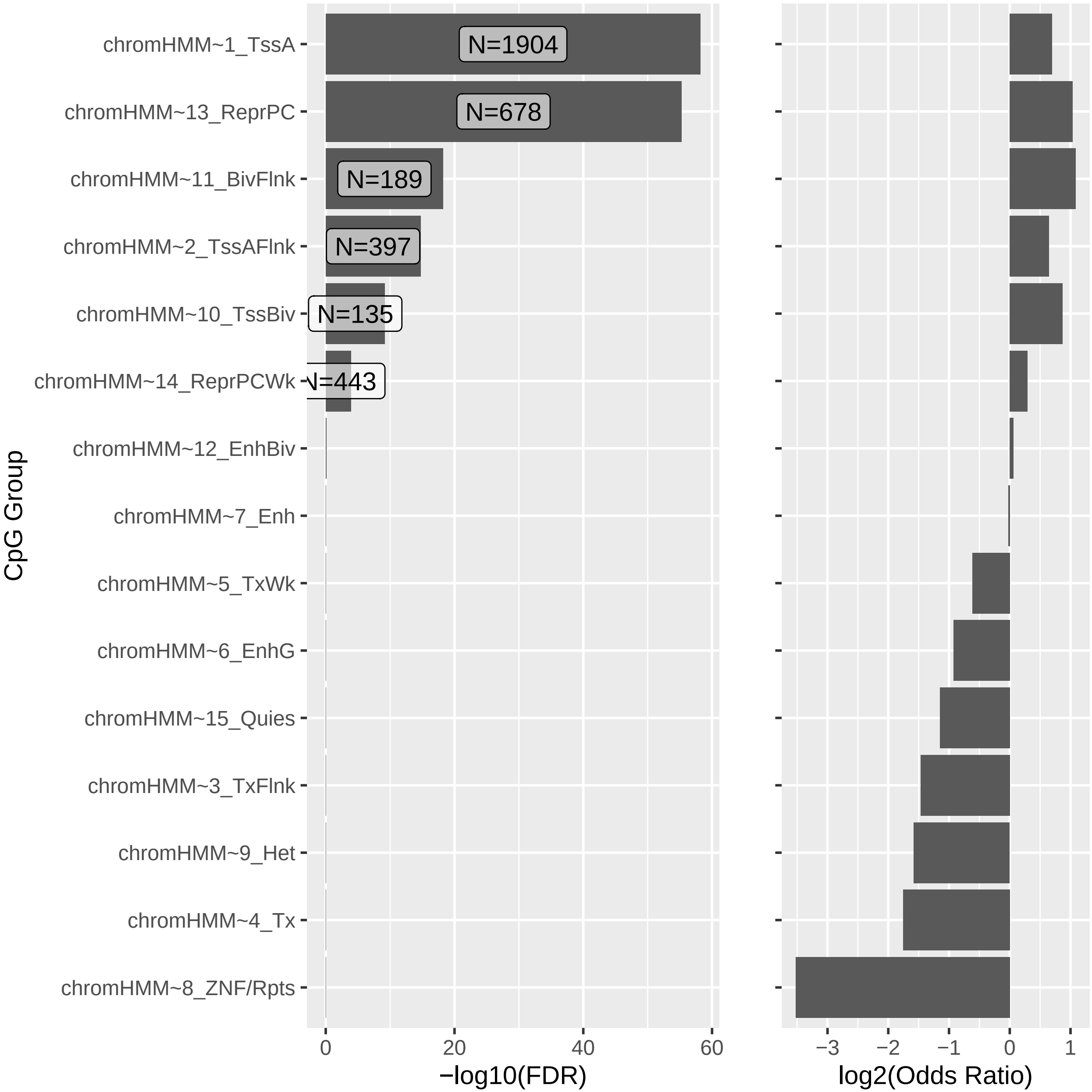
CpG Group
chromHMM~1_TssA
chromHMM~13_ReprPC
chromHMM~11_BivFlnk
chromHMM~2_TssAFlnk
chromHMM~10_TssBiv
chromHMM~14_ReprPCWk
chromHMM~12_EnhBiv
chromHMM~7_Enh
chromHMM~5_TxWk
chromHMM~6_EnhG
chromHMM~15_Quies
chromHMM~3_TxFlnk
chromHMM~9_Het
chromHMM~4_Tx
chromHMM~8_ZNF/Rpts
N=1904
N=678
N=189
N=397
N=135
N=443
0
20
40
60
−log10(FDR)
−3
−2
−1
0
1
log2(Odds Ratio)

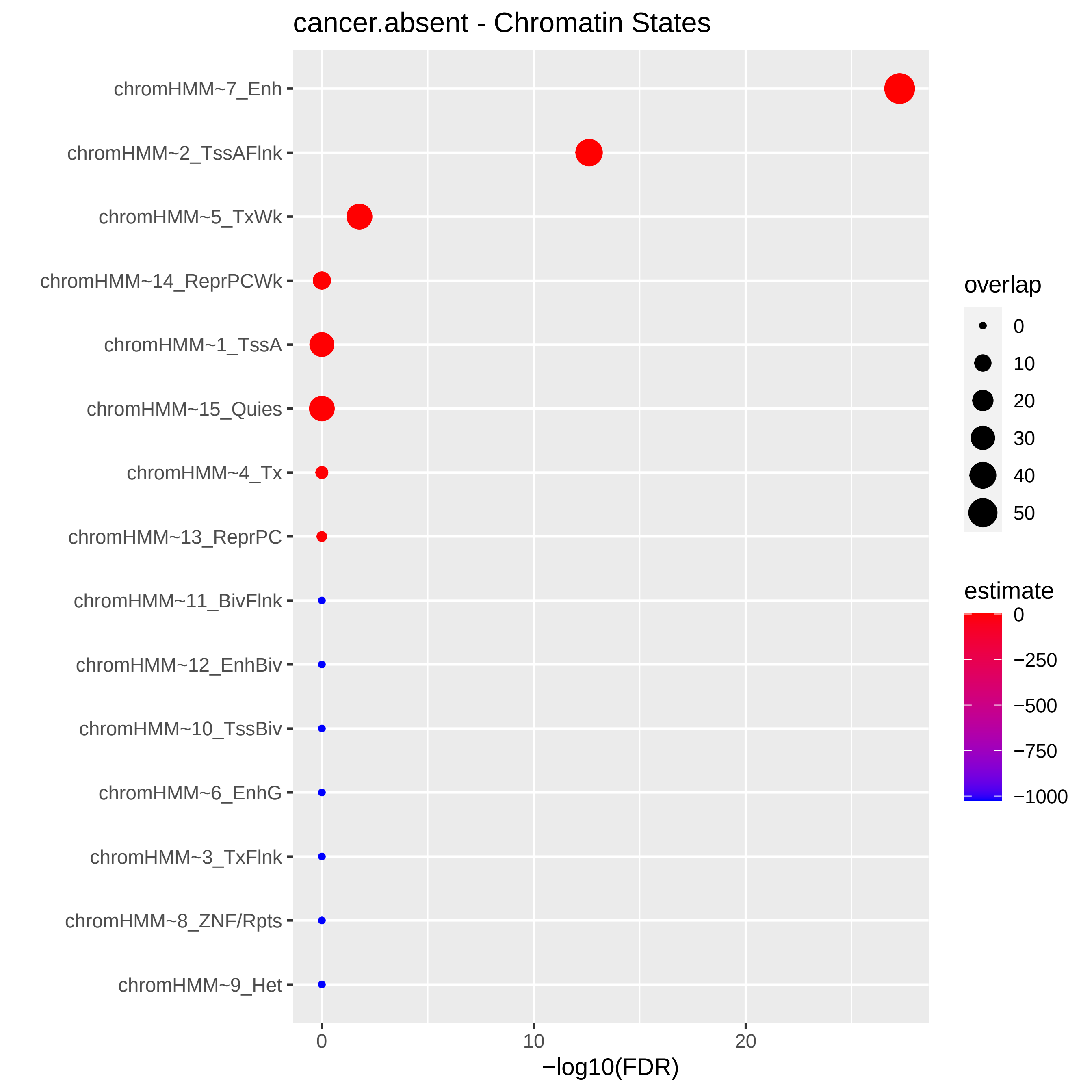
cancer.absent - Chromatin States
chromHMM~7_Enh
chromHMM~2_TssAFlnk
chromHMM~5_TxWk
chromHMM~14_ReprPCWk
chromHMM~1_TssA
chromHMM~15_Quies
chromHMM~4_Tx
chromHMM~13_ReprPC
chromHMM~11_BivFlnk
chromHMM~12_EnhBiv
chromHMM~10_TssBiv
chromHMM~6_EnhG
chromHMM~3_TxFlnk
chromHMM~8_ZNF/Rpts
chromHMM~9_Het
0
10
20
−log10(FDR)
overlap
0
10
20
30
40
50
estimate
0
−250
−500
−750
−1000

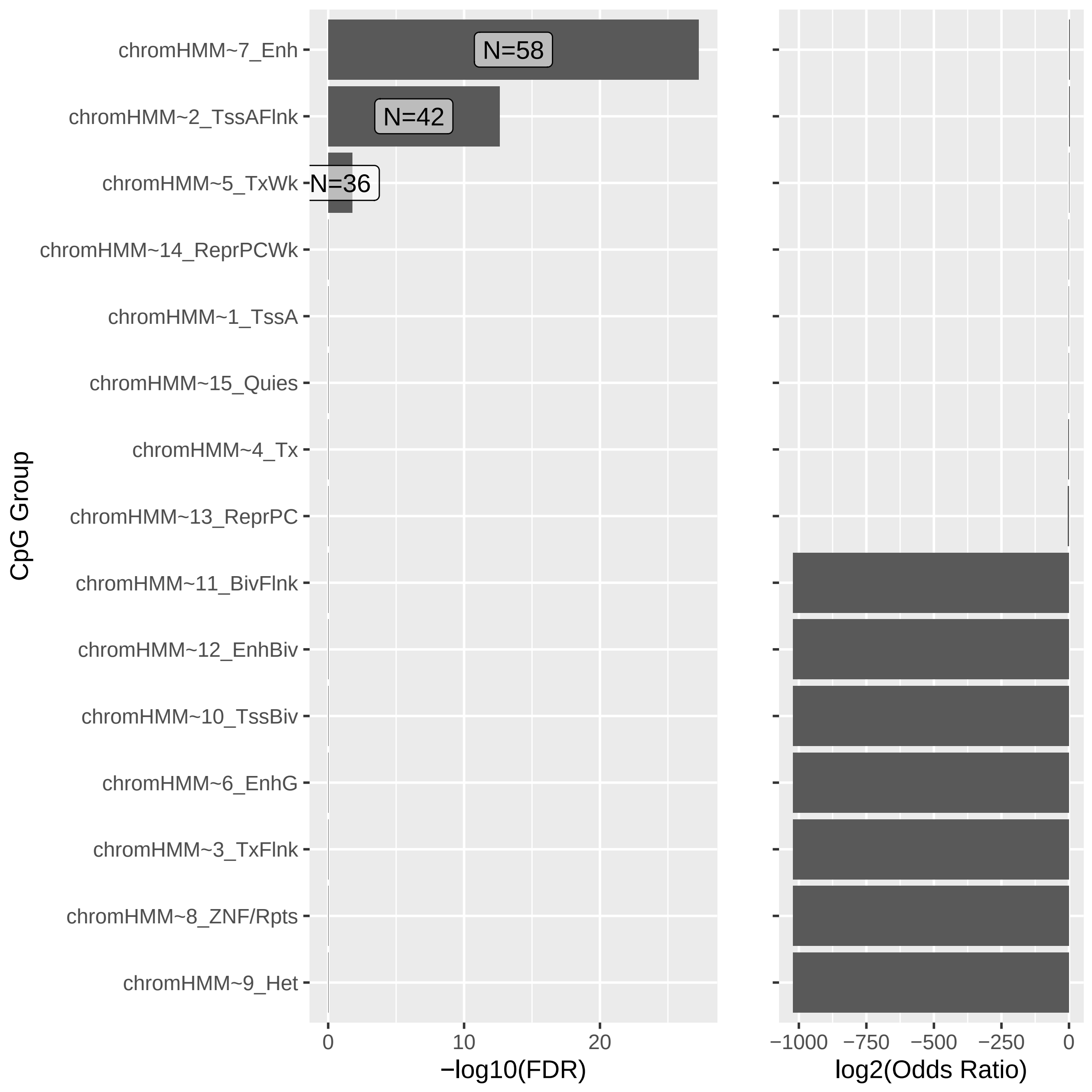

CpG Group
chromHMM~7_Enh
chromHMM~2_TssAFlnk
chromHMM~5_TxWk
chromHMM~14_ReprPCWk
chromHMM~1_TssA
chromHMM~15_Quies
chromHMM~4_Tx
chromHMM~13_ReprPC
chromHMM~11_BivFlnk
chromHMM~12_EnhBiv
chromHMM~10_TssBiv
chromHMM~6_EnhG
chromHMM~3_TxFlnk
chromHMM~8_ZNF/Rpts
chromHMM~9_Het
N=58
N=42
N=36
0
10
20
−log10(FDR)
−1000
−750
−500
−250
0
log2(Odds Ratio)

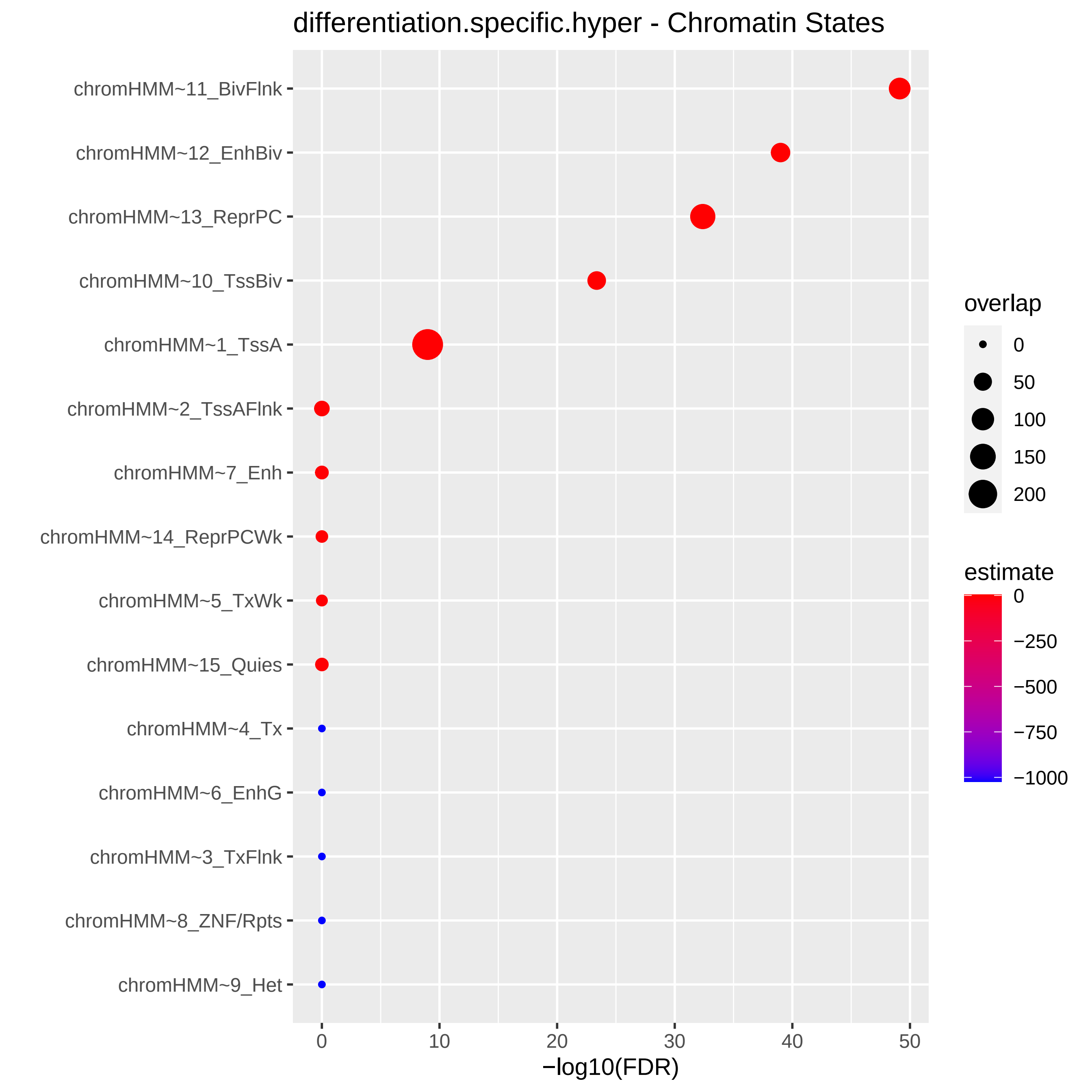

differentiation.specific.hyper - Chromatin States
chromHMM~11_BivFlnk
chromHMM~12_EnhBiv
chromHMM~13_ReprPC
chromHMM~10_TssBiv
chromHMM~1_TssA
chromHMM~2_TssAFlnk
chromHMM~7_Enh
chromHMM~14_ReprPCWk
chromHMM~5_TxWk
chromHMM~15_Quies
chromHMM~4_Tx
chromHMM~6_EnhG
chromHMM~3_TxFlnk
chromHMM~8_ZNF/Rpts
chromHMM~9_Het
0
10
20
30
40
50
−log10(FDR)
overlap
0
50
100
150
200
estimate
0
−250
−500
−750
−1000

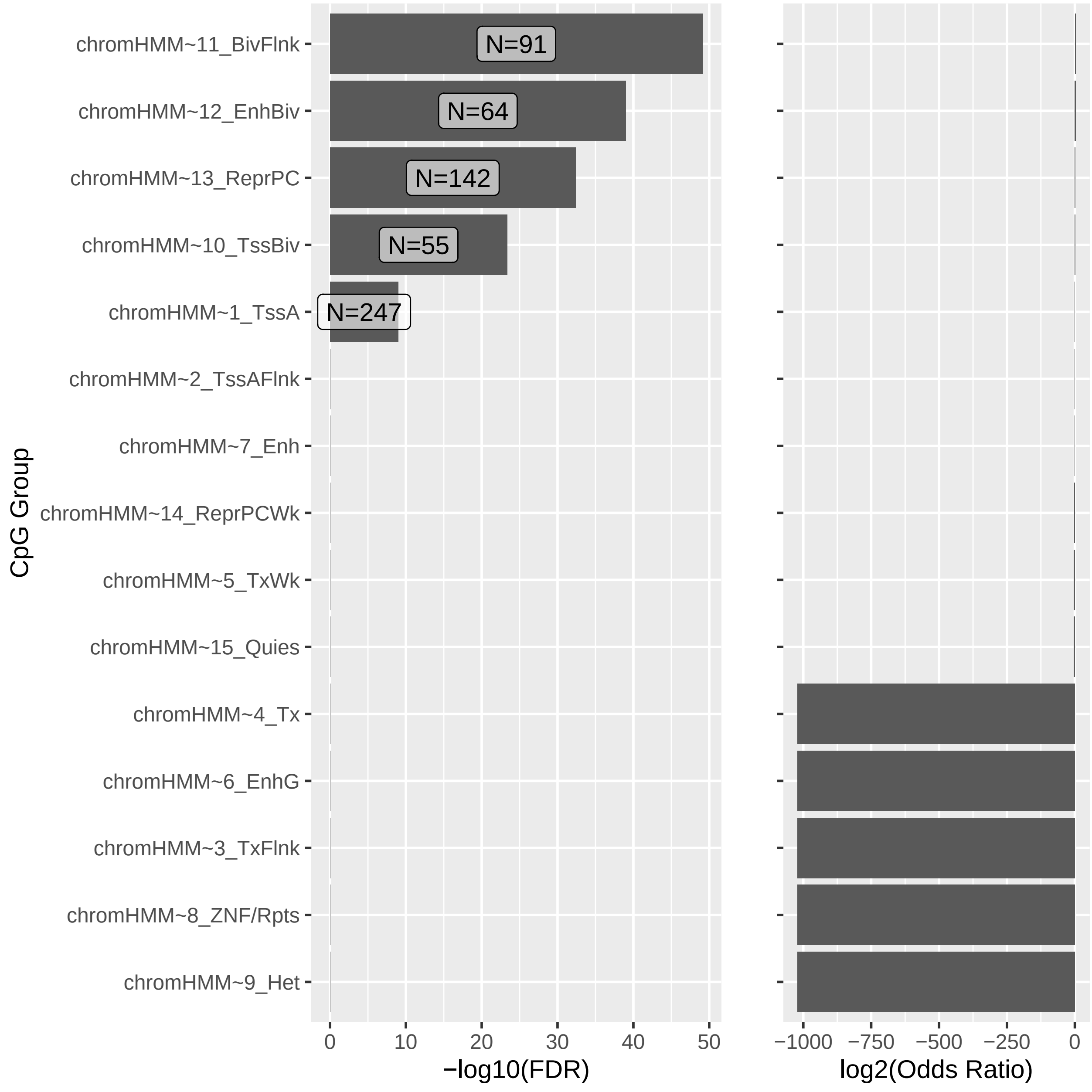

CpG Group
chromHMM~11_BivFlnk
chromHMM~12_EnhBiv
chromHMM~13_ReprPC
chromHMM~10_TssBiv
chromHMM~1_TssA
chromHMM~2_TssAFlnk
chromHMM~7_Enh
chromHMM~14_ReprPCWk
chromHMM~5_TxWk
chromHMM~15_Quies
chromHMM~4_Tx
chromHMM~6_EnhG
chromHMM~3_TxFlnk
chromHMM~8_ZNF/Rpts
chromHMM~9_Het
N=91
N=64
N=142
N=55
N=247
0
10
20
30
40
50
−log10(FDR)
−1000
−750
−500
−250
0
log2(Odds Ratio)

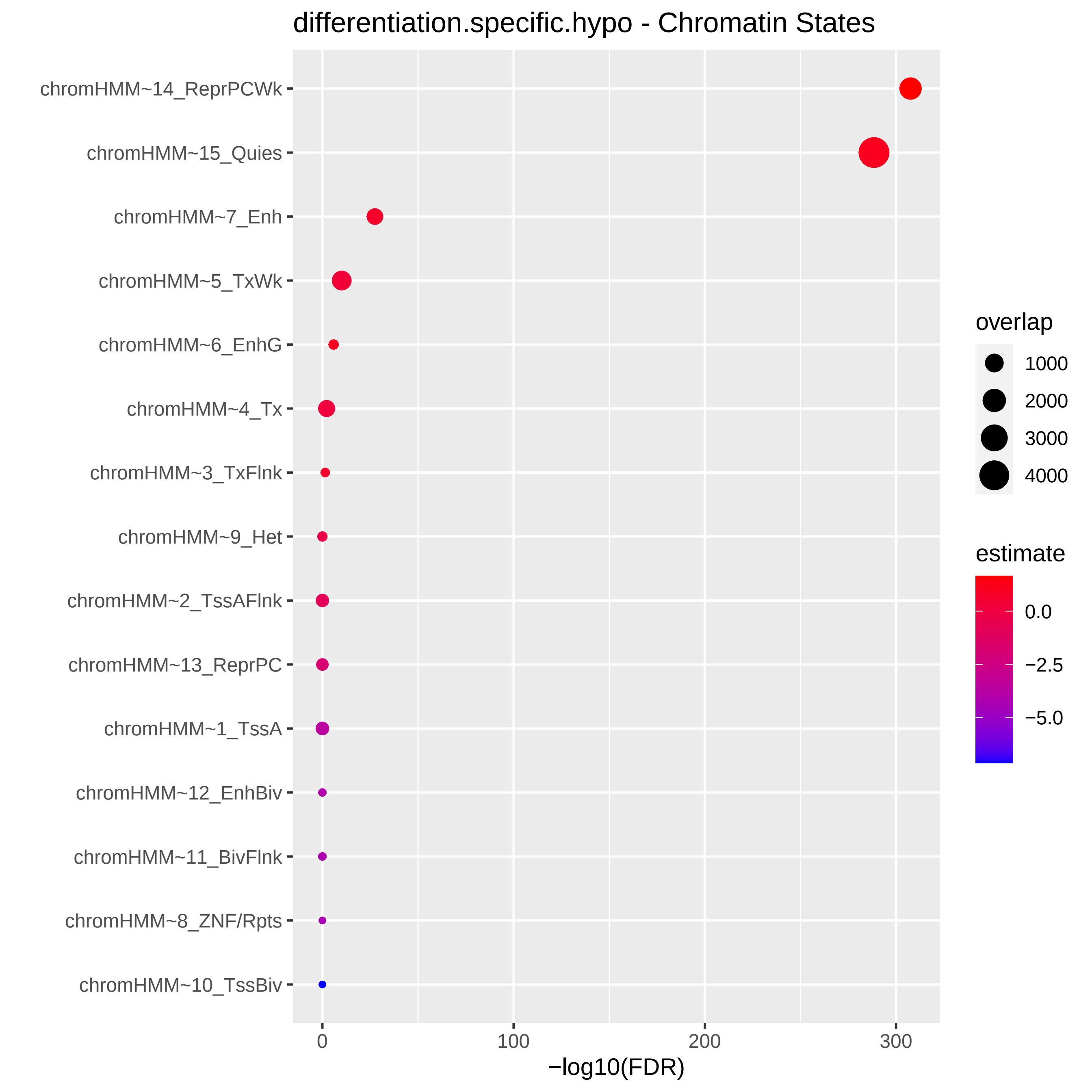
differentiation.specific.hypo - Chromatin States
chromHMM~14_ReprPCWk
chromHMM~15_Quies
chromHMM~7_Enh
chromHMM~5_TxWk
chromHMM~6_EnhG
chromHMM~4_Tx
chromHMM~3_TxFlnk
chromHMM~9_Het
chromHMM~2_TssAFlnk
chromHMM~13_ReprPC
chromHMM~1_TssA
chromHMM~12_EnhBiv
chromHMM~11_BivFlnk
chromHMM~8_ZNF/Rpts
chromHMM~10_TssBiv
0
100
200
300
−log10(FDR)
overlap
1000
2000
3000
4000
estimate
0.0
−2.5
−5.0

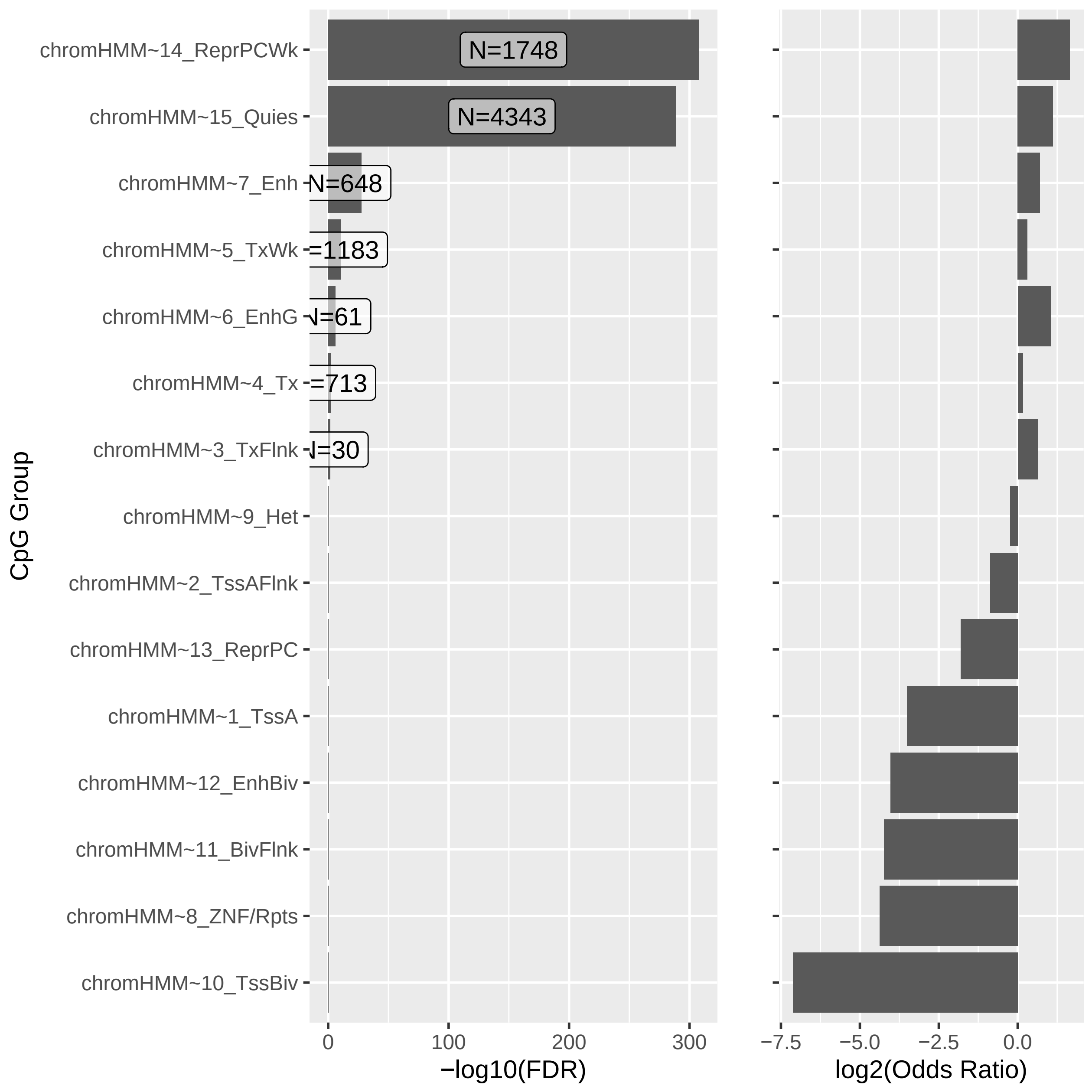

chromHMM~14_ReprPCWk
chromHMM~15_Quies
chromHMM~7_Enh
chromHMM~5_TxWk
chromHMM~6_EnhG
chromHMM~4_Tx
chromHMM~3_TxFlnk
chromHMM~9_Het
chromHMM~2_TssAFlnk
chromHMM~13_ReprPC
chromHMM~1_TssA
chromHMM~12_EnhBiv
chromHMM~11_BivFlnk
chromHMM~8_ZNF/Rpts
chromHMM~10_TssBiv
N=1748
N=4343
N=648
=1183
N=61
=713
N=30
CpG Group
0
100
200
300
−log10(FDR)
−7.5
−5.0
−2.5
0.0
log2(Odds Ratio)

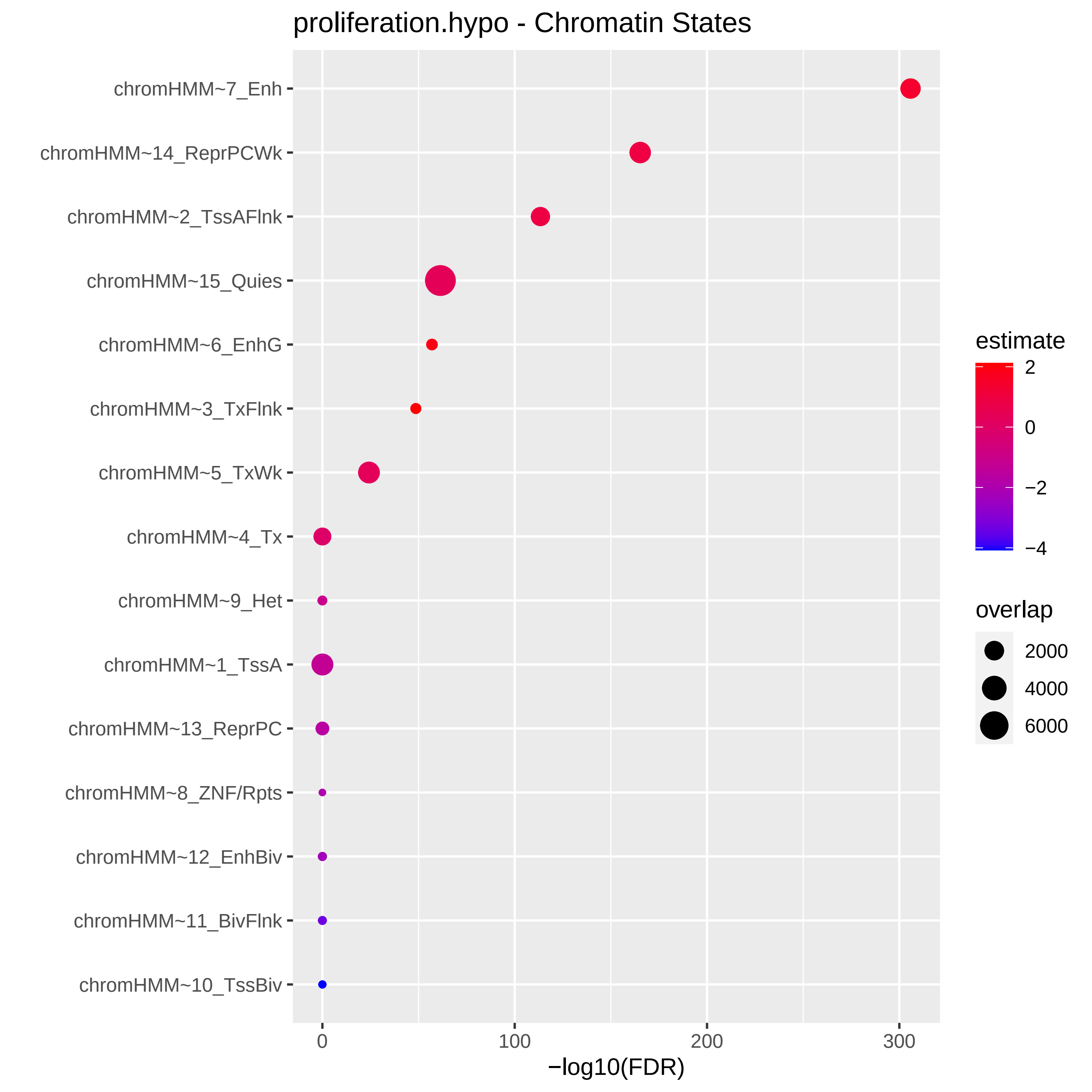
proliferation.hypo - Chromatin States
chromHMM~7_Enh
chromHMM~14_ReprPCWk
chromHMM~2_TssAFlnk
chromHMM~15_Quies
chromHMM~6_EnhG
chromHMM~3_TxFlnk
chromHMM~5_TxWk
chromHMM~4_Tx
chromHMM~9_Het
chromHMM~1_TssA
chromHMM~13_ReprPC
chromHMM~8_ZNF/Rpts
chromHMM~12_EnhBiv
chromHMM~11_BivFlnk
chromHMM~10_TssBiv
0
100
200
300
−log10(FDR)
estimate
2
0
−2
−4
overlap
2000
4000
6000

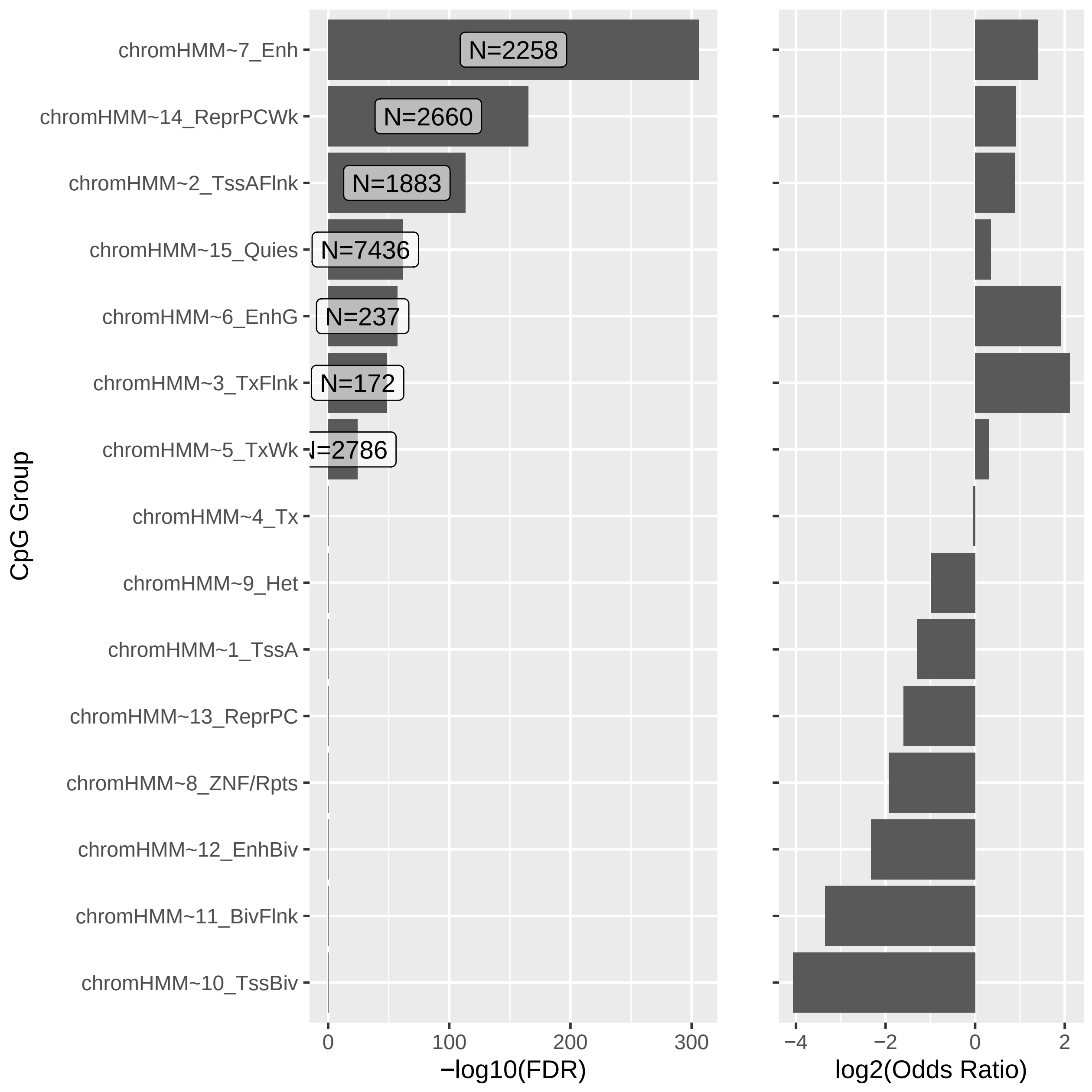
CpG Group
chromHMM~7_Enh
chromHMM~14_ReprPCWk
chromHMM~2_TssAFlnk
chromHMM~15_Quies
chromHMM~6_EnhG
chromHMM~3_TxFlnk
chromHMM~5_TxWk
chromHMM~4_Tx
chromHMM~9_Het
chromHMM~1_TssA
chromHMM~13_ReprPC
chromHMM~8_ZNF/Rpts
chromHMM~12_EnhBiv
chromHMM~11_BivFlnk
chromHMM~10_TssBiv
N=2258
N=2660
N=1883
N=7436
N=237
N=172
N=2786
0
100
200
300
−log10(FDR)
−4
−2
0
2
log2(Odds Ratio)

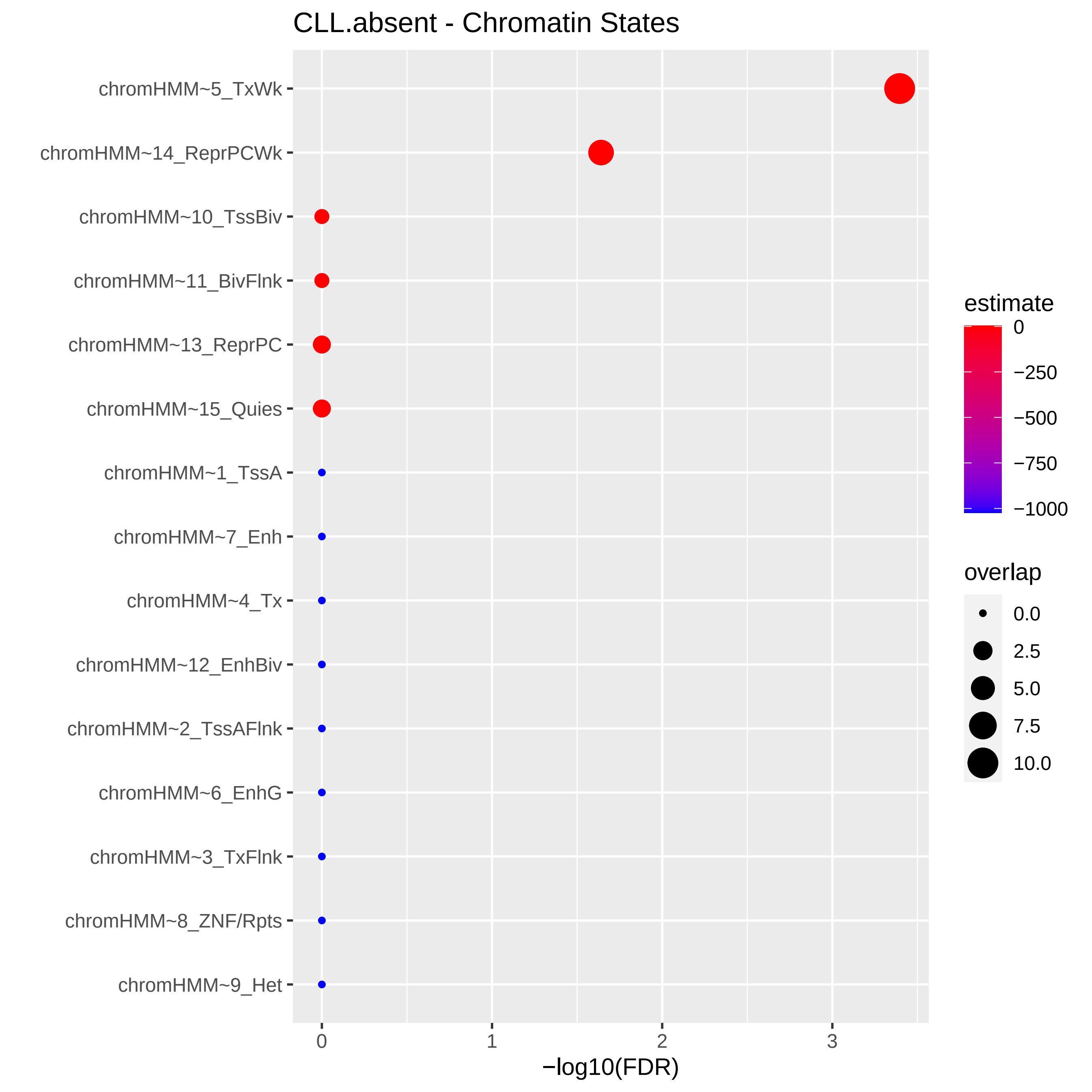
CLL.absent - Chromatin States
chromHMM~5_TxWk
chromHMM~14_ReprPCWk
chromHMM~10_TssBiv
chromHMM~11_BivFlnk
chromHMM~13_ReprPC
chromHMM~15_Quies
chromHMM~1_TssA
chromHMM~7_Enh
chromHMM~4_Tx
chromHMM~12_EnhBiv
chromHMM~2_TssAFlnk
chromHMM~6_EnhG
chromHMM~3_TxFlnk
chromHMM~8_ZNF/Rpts
chromHMM~9_Het
0
1
2
3
−log10(FDR)
estimate
0
−250
−500
−750
−1000
overlap
0.0
2.5
5.0
7.5
10.0

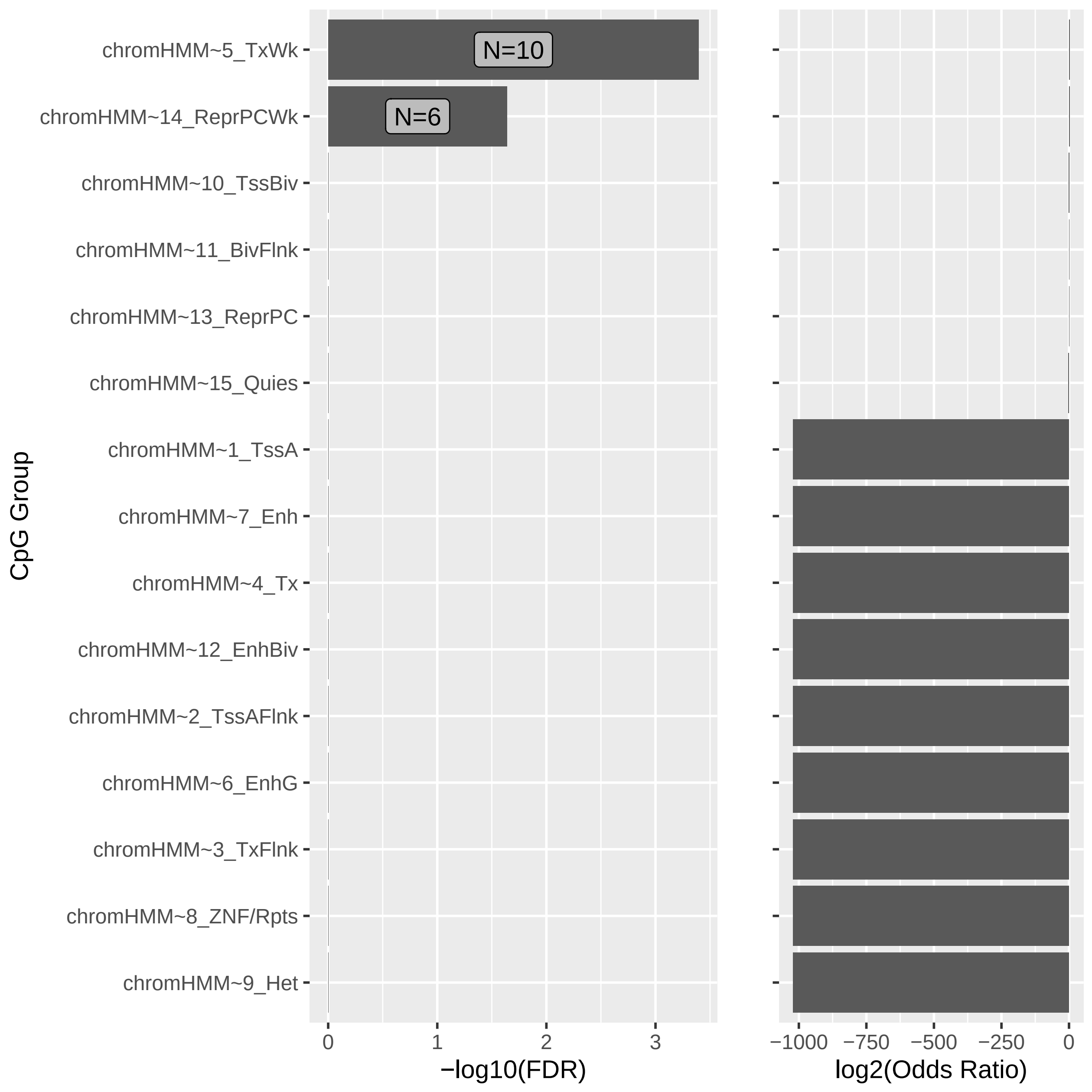
CpG Group
chromHMM~5_TxWk
chromHMM~14_ReprPCWk
chromHMM~10_TssBiv
chromHMM~11_BivFlnk
chromHMM~13_ReprPC
chromHMM~15_Quies
chromHMM~1_TssA
chromHMM~7_Enh
chromHMM~4_Tx
chromHMM~12_EnhBiv
chromHMM~2_TssAFlnk
chromHMM~6_EnhG
chromHMM~3_TxFlnk
chromHMM~8_ZNF/Rpts
chromHMM~9_Het
N=10
N=6
0
1
2
3
−log10(FDR)
−1000
−750
−500
−250
0
log2(Odds Ratio)

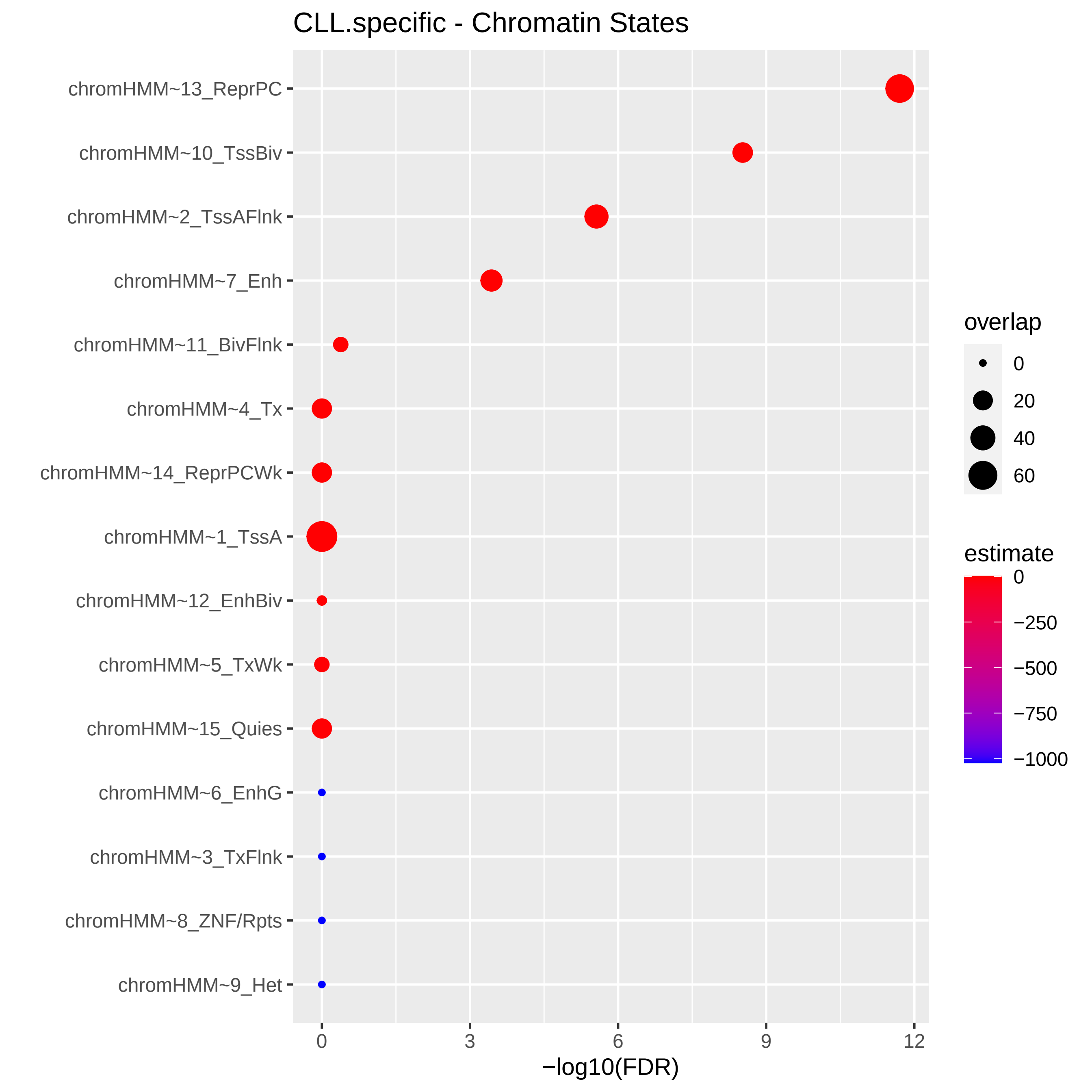

CLL.specific - Chromatin States
chromHMM~13_ReprPC
chromHMM~10_TssBiv
chromHMM~2_TssAFlnk
chromHMM~7_Enh
chromHMM~11_BivFlnk
chromHMM~4_Tx
chromHMM~14_ReprPCWk
chromHMM~1_TssA
chromHMM~12_EnhBiv
chromHMM~5_TxWk
chromHMM~15_Quies
chromHMM~6_EnhG
chromHMM~3_TxFlnk
chromHMM~8_ZNF/Rpts
chromHMM~9_Het
0
3
6
9
12
−log10(FDR)
overlap
0
20
40
60
estimate
0
−250
−500
−750
−1000

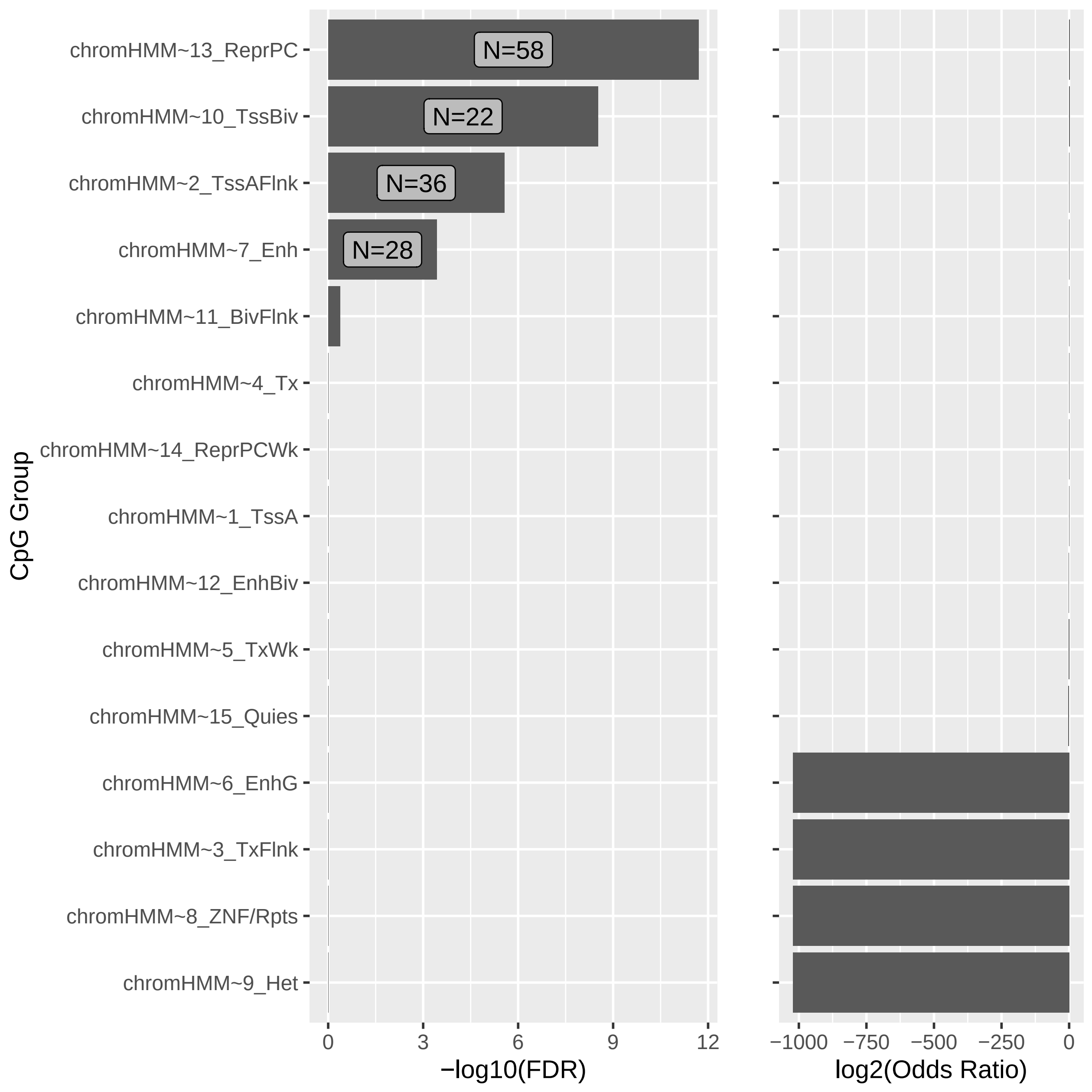
CpG Group
chromHMM~13_ReprPC
chromHMM~10_TssBiv
chromHMM~2_TssAFlnk
chromHMM~7_Enh
chromHMM~11_BivFlnk
chromHMM~4_Tx
chromHMM~14_ReprPCWk
chromHMM~1_TssA
chromHMM~12_EnhBiv
chromHMM~5_TxWk
chromHMM~15_Quies
chromHMM~6_EnhG
chromHMM~3_TxFlnk
chromHMM~8_ZNF/Rpts
chromHMM~9_Het
N=58
N=22
N=36
N=28
0
3
6
9
12
−log10(FDR)
−1000
−750
−500
−250
0
log2(Odds Ratio)

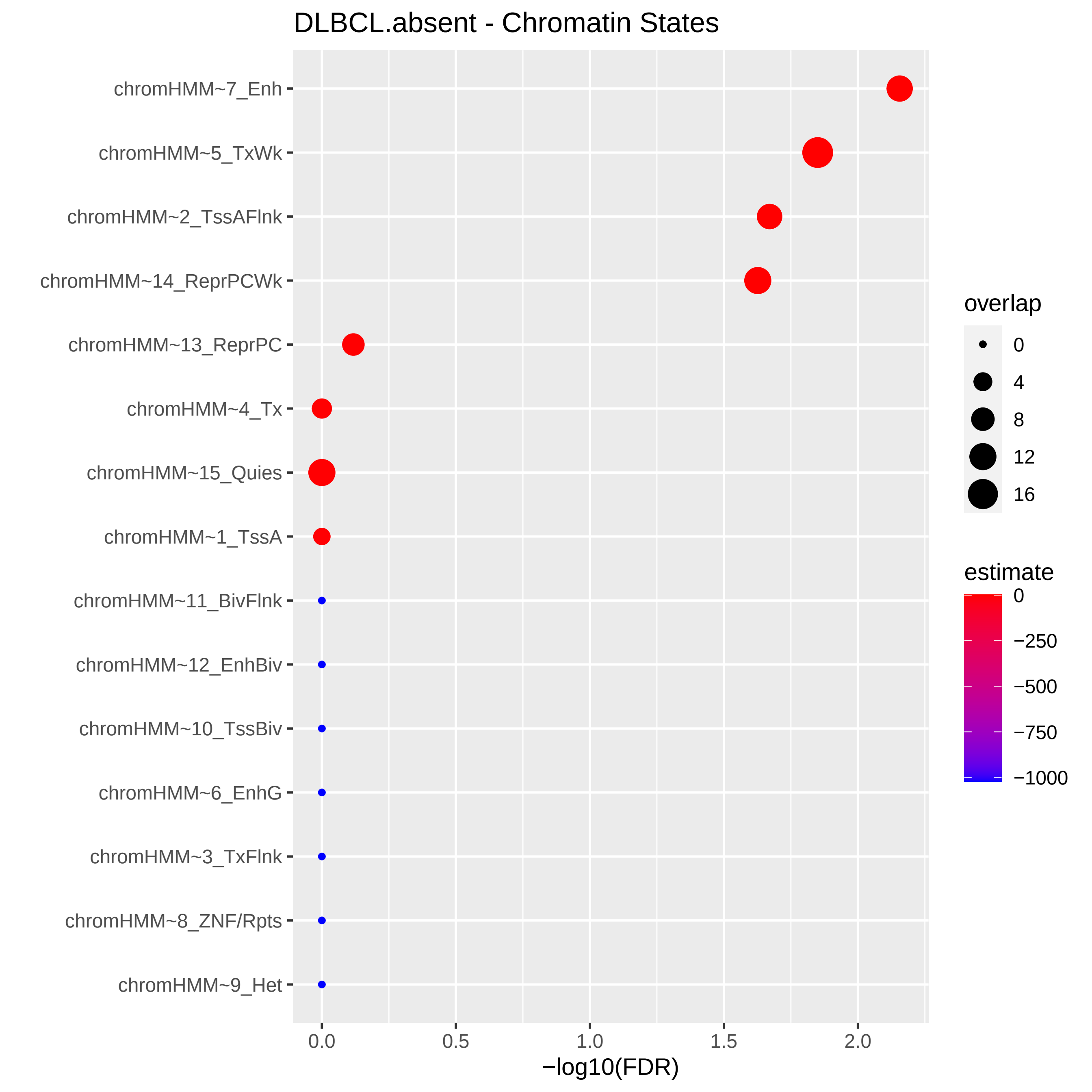

DLBCL.absent - Chromatin States
chromHMM~7_Enh
chromHMM~5_TxWk
chromHMM~2_TssAFlnk
chromHMM~14_ReprPCWk
chromHMM~13_ReprPC
chromHMM~4_Tx
chromHMM~15_Quies
chromHMM~1_TssA
chromHMM~11_BivFlnk
chromHMM~12_EnhBiv
chromHMM~10_TssBiv
chromHMM~6_EnhG
chromHMM~3_TxFlnk
chromHMM~8_ZNF/Rpts
chromHMM~9_Het
0.0
0.5
1.0
1.5
2.0
−log10(FDR)
overlap
0
4
8
12
16
estimate
0
−250
−500
−750
−1000

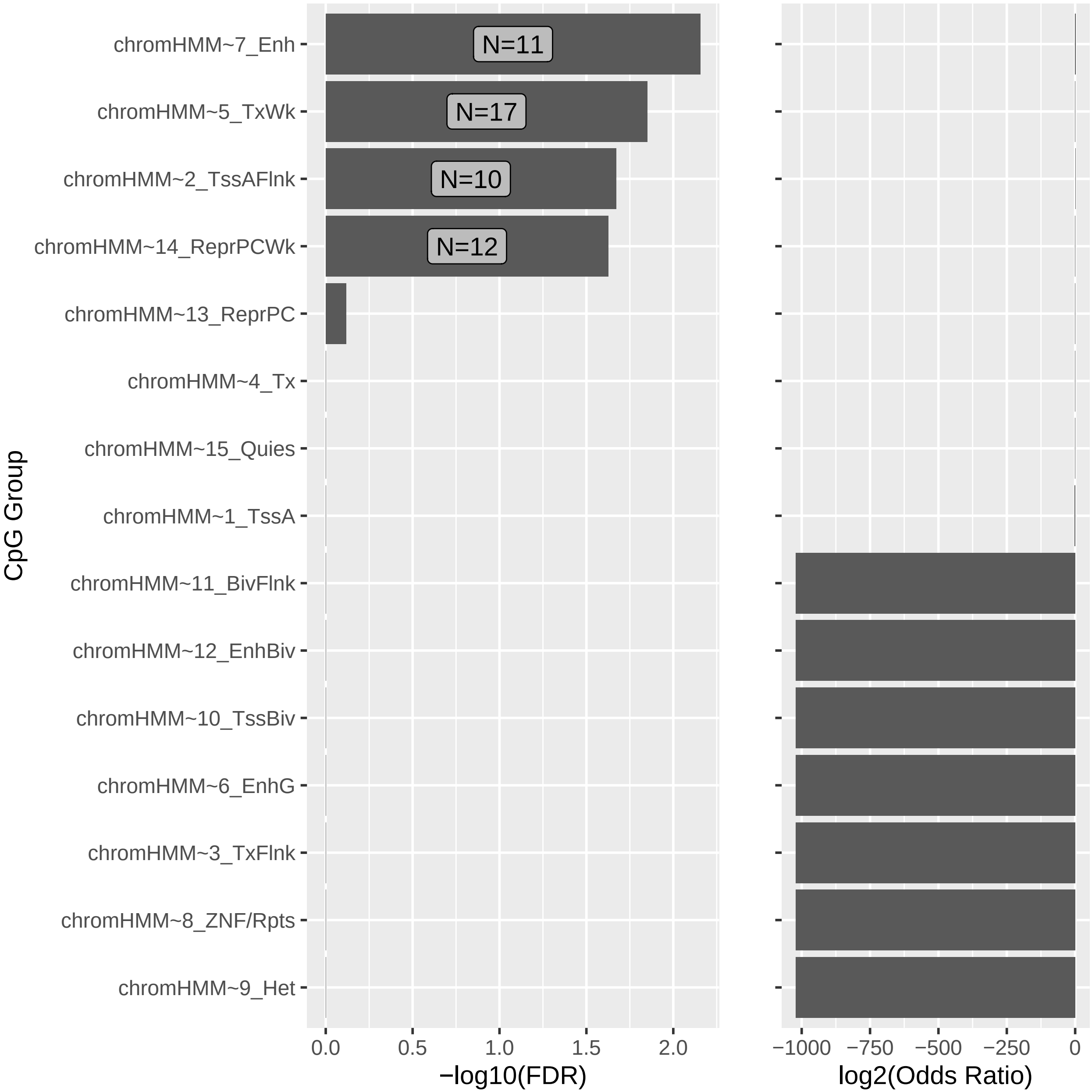

CpG Group
chromHMM~7_Enh
chromHMM~5_TxWk
chromHMM~2_TssAFlnk
chromHMM~14_ReprPCWk
chromHMM~13_ReprPC
chromHMM~4_Tx
chromHMM~15_Quies
chromHMM~1_TssA
chromHMM~11_BivFlnk
chromHMM~12_EnhBiv
chromHMM~10_TssBiv
chromHMM~6_EnhG
chromHMM~3_TxFlnk
chromHMM~8_ZNF/Rpts
chromHMM~9_Het
N=11
N=17
N=10
N=12
0.0
0.5
1.0
1.5
2.0
−log10(FDR)
−1000
−750
−500
−250
0
log2(Odds Ratio)

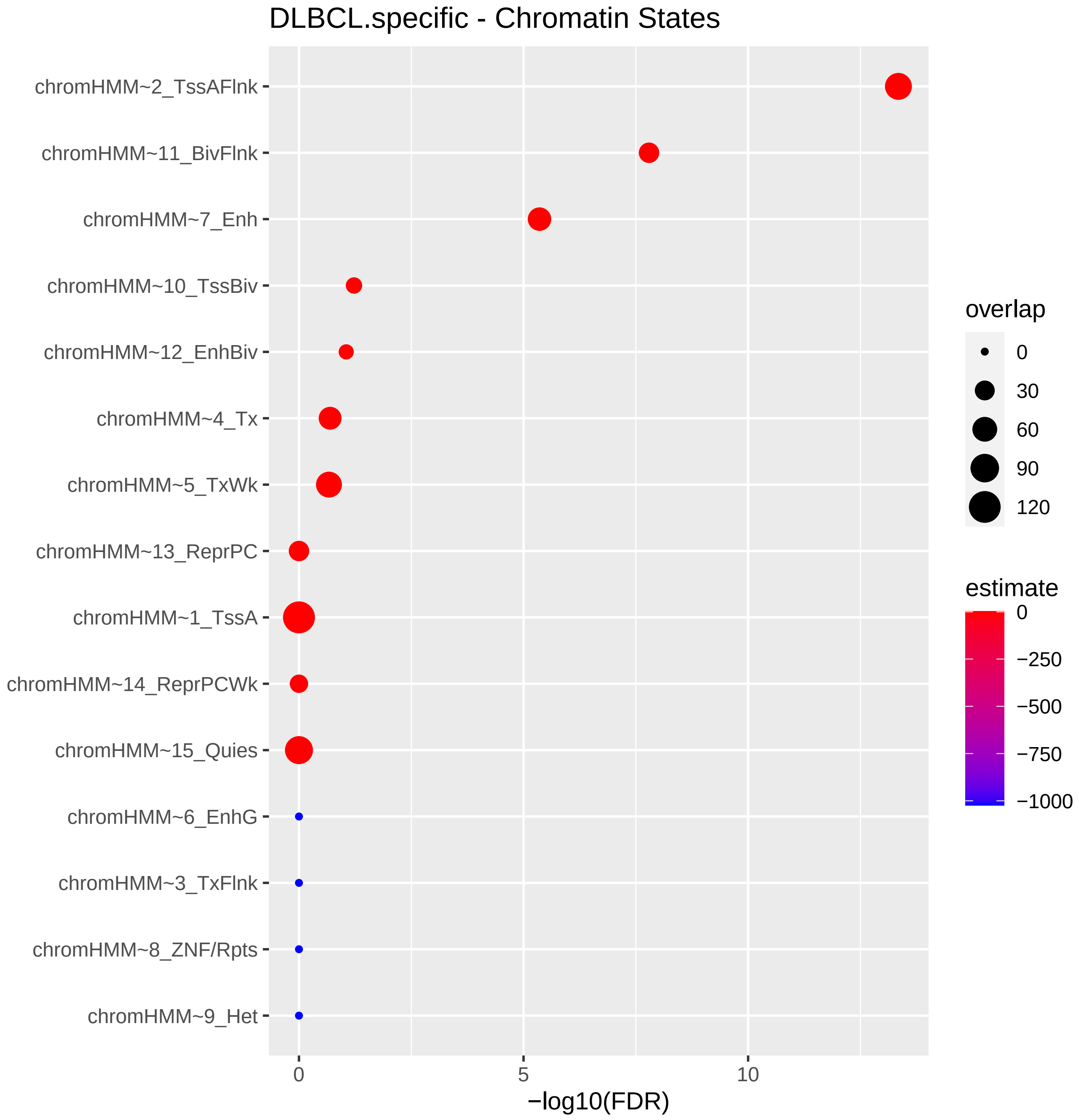

DLBCL.specific - Chromatin States
chromHMM~2_TssAFlnk
chromHMM~11_BivFlnk
chromHMM~7_Enh
chromHMM~10_TssBiv
chromHMM~12_EnhBiv
chromHMM~4_Tx
chromHMM~5_TxWk
chromHMM~13_ReprPC
chromHMM~1_TssA
chromHMM~14_ReprPCWk
chromHMM~15_Quies
chromHMM~6_EnhG
chromHMM~3_TxFlnk
chromHMM~8_ZNF/Rpts
chromHMM~9_Het
0
5
10
−log10(FDR)
overlap
0
30
60
90
120
estimate
0
−250
−500
−750
−1000

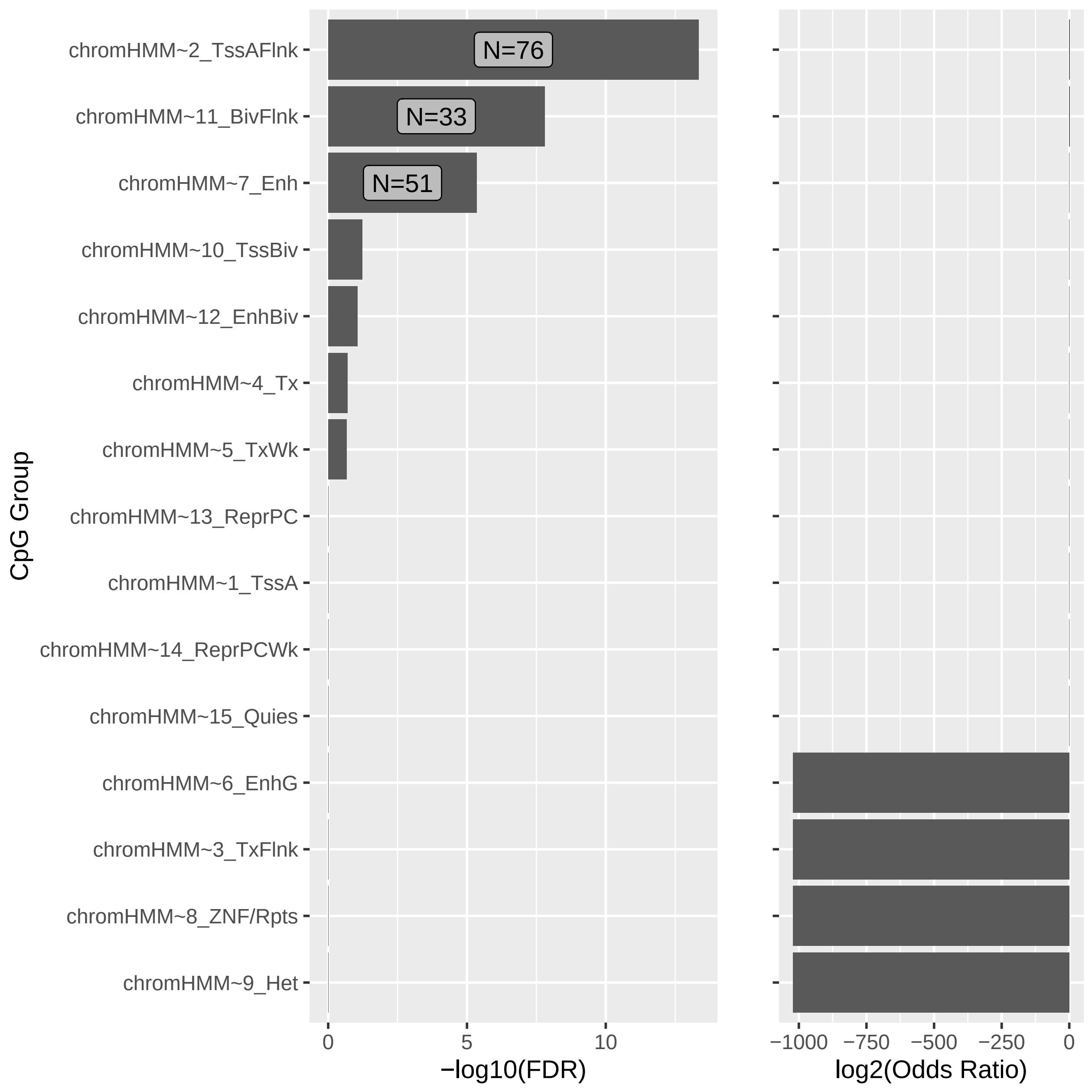

CpG Group
chromHMM~2_TssAFlnk
chromHMM~11_BivFlnk
chromHMM~7_Enh
chromHMM~10_TssBiv
chromHMM~12_EnhBiv
chromHMM~4_Tx
chromHMM~5_TxWk
chromHMM~13_ReprPC
chromHMM~1_TssA
chromHMM~14_ReprPCWk
chromHMM~15_Quies
chromHMM~6_EnhG
chromHMM~3_TxFlnk
chromHMM~8_ZNF/Rpts
chromHMM~9_Het
N=76
N=33
N=51
0
5
10
−log10(FDR)
−1000
−750
−500
−250
0
log2(Odds Ratio)

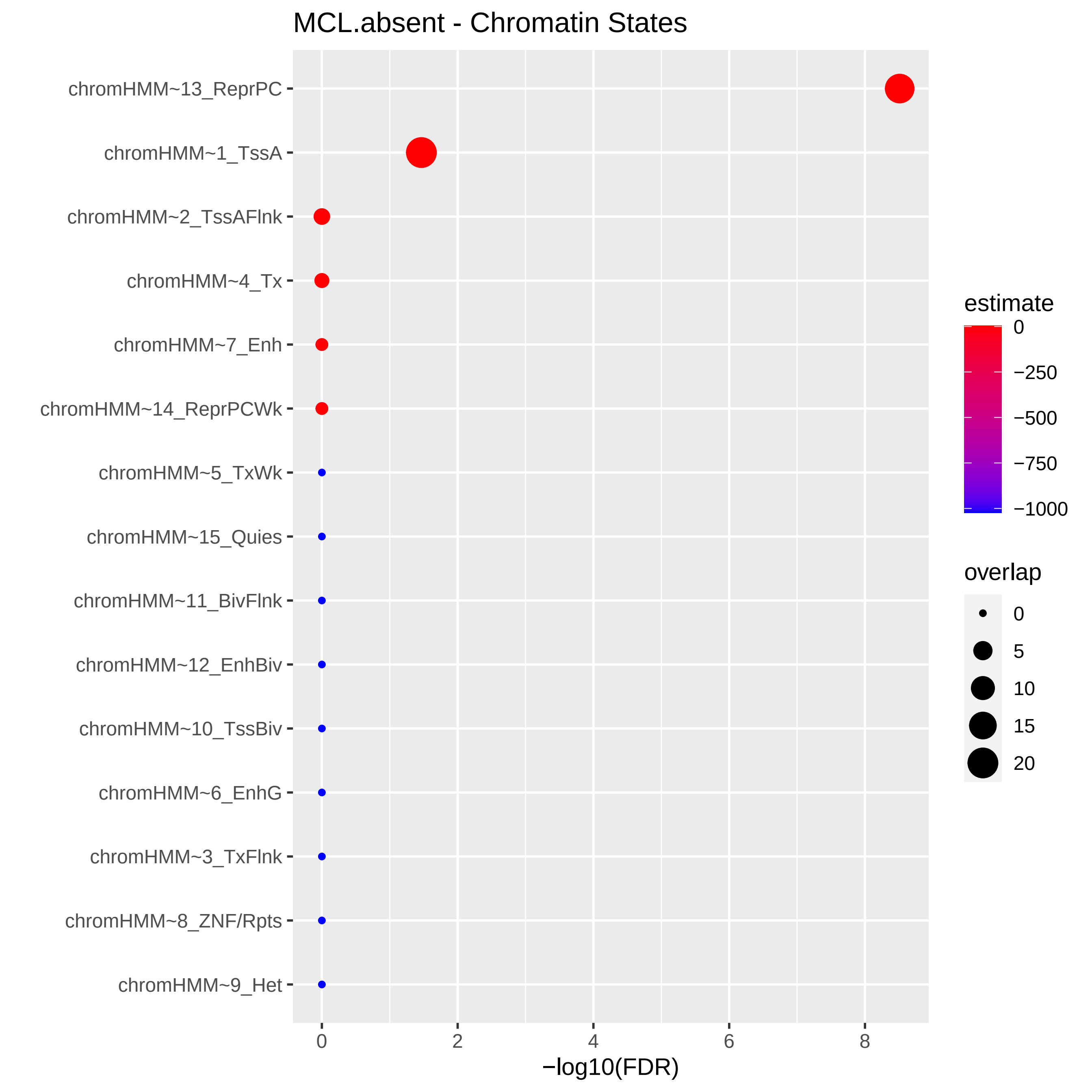

MCL.absent - Chromatin States
chromHMM~13_ReprPC
chromHMM~1_TssA
chromHMM~2_TssAFlnk
chromHMM~4_Tx
chromHMM~7_Enh
chromHMM~14_ReprPCWk
chromHMM~5_TxWk
chromHMM~15_Quies
chromHMM~11_BivFlnk
chromHMM~12_EnhBiv
chromHMM~10_TssBiv
chromHMM~6_EnhG
chromHMM~3_TxFlnk
chromHMM~8_ZNF/Rpts
chromHMM~9_Het
0
2
4
6
8
−log10(FDR)
estimate
0
−250
−500
−750
−1000
overlap
0
5
10
15
20

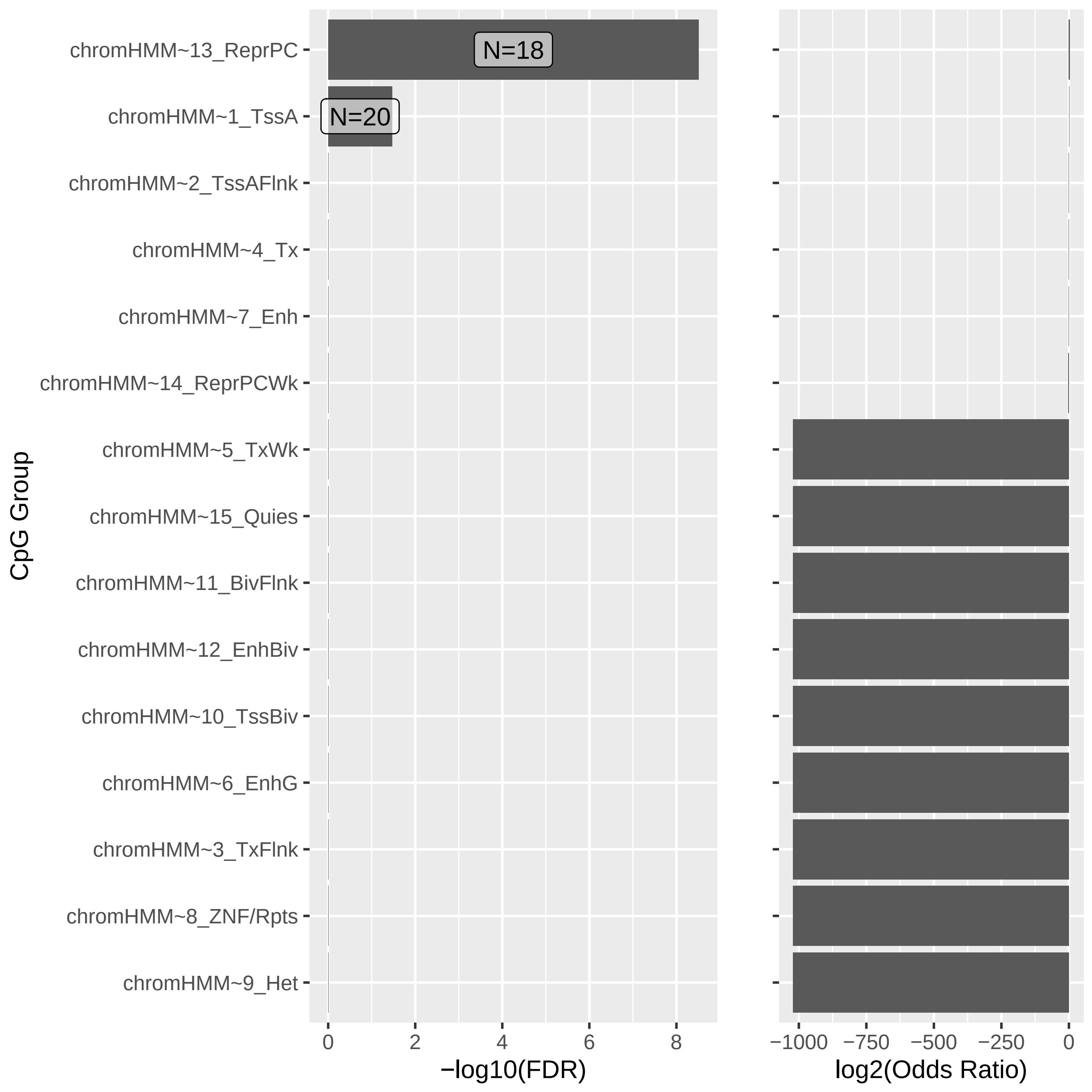
CpG Group
chromHMM~13_ReprPC
chromHMM~1_TssA
chromHMM~2_TssAFlnk
chromHMM~4_Tx
chromHMM~7_Enh
chromHMM~14_ReprPCWk
chromHMM~5_TxWk
chromHMM~15_Quies
chromHMM~11_BivFlnk
chromHMM~12_EnhBiv
chromHMM~10_TssBiv
chromHMM~6_EnhG
chromHMM~3_TxFlnk
chromHMM~8_ZNF/Rpts
chromHMM~9_Het
N=18
N=20
0
2
4
6
8
−log10(FDR)
−1000
−750
−500
−250
0
log2(Odds Ratio)

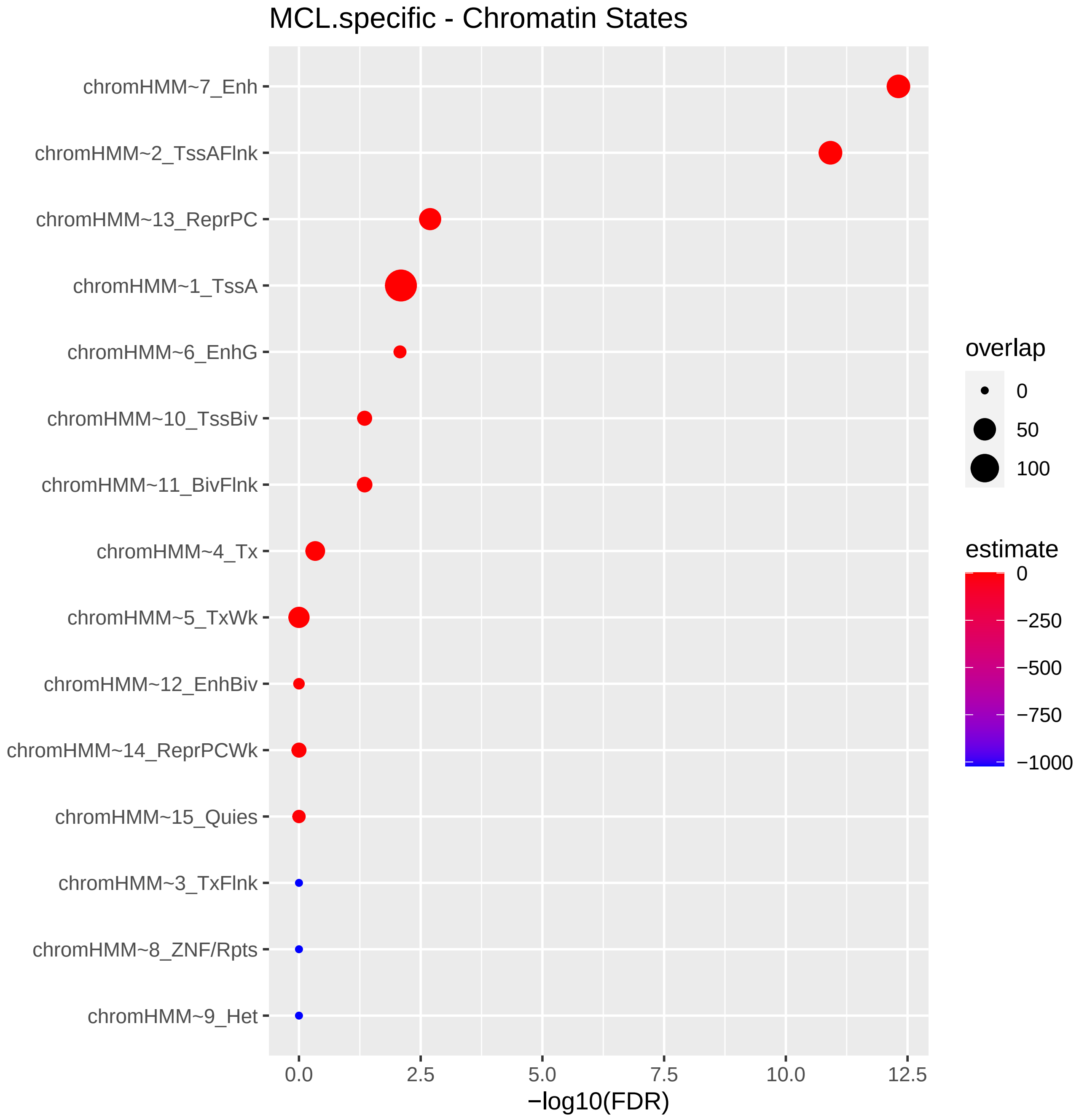

MCL.specific - Chromatin States
chromHMM~7_Enh
chromHMM~2_TssAFlnk
chromHMM~13_ReprPC
chromHMM~1_TssA
chromHMM~6_EnhG
chromHMM~10_TssBiv
chromHMM~11_BivFlnk
chromHMM~4_Tx
chromHMM~5_TxWk
chromHMM~12_EnhBiv
chromHMM~14_ReprPCWk
chromHMM~15_Quies
chromHMM~3_TxFlnk
chromHMM~8_ZNF/Rpts
chromHMM~9_Het
0.0
2.5
5.0
7.5
10.0
12.5
−log10(FDR)
overlap
0
50
100
estimate
0
−250
−500
−750
−1000

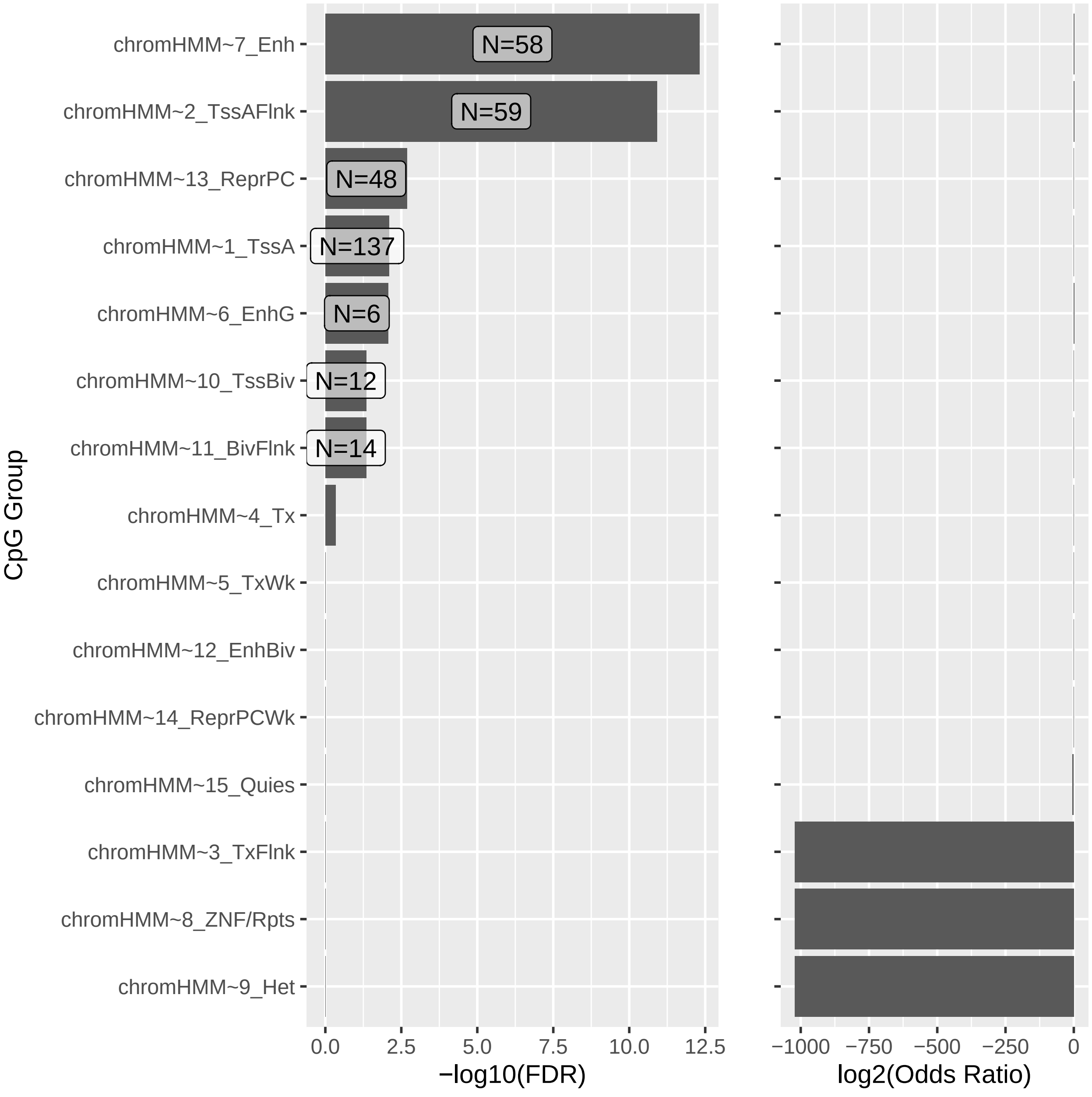

CpG Group
chromHMM~7_Enh
chromHMM~2_TssAFlnk
chromHMM~13_ReprPC
chromHMM~1_TssA
chromHMM~6_EnhG
chromHMM~10_TssBiv
chromHMM~11_BivFlnk
chromHMM~4_Tx
chromHMM~5_TxWk
chromHMM~12_EnhBiv
chromHMM~14_ReprPCWk
chromHMM~15_Quies
chromHMM~3_TxFlnk
chromHMM~8_ZNF/Rpts
chromHMM~9_Het
N=58
N=59
N=48
N=137
N=6
N=12
N=14
0.0
2.5
5.0
7.5
10.0
12.5
−log10(FDR)
−1000
−750
−500
−250
0
log2(Odds Ratio)

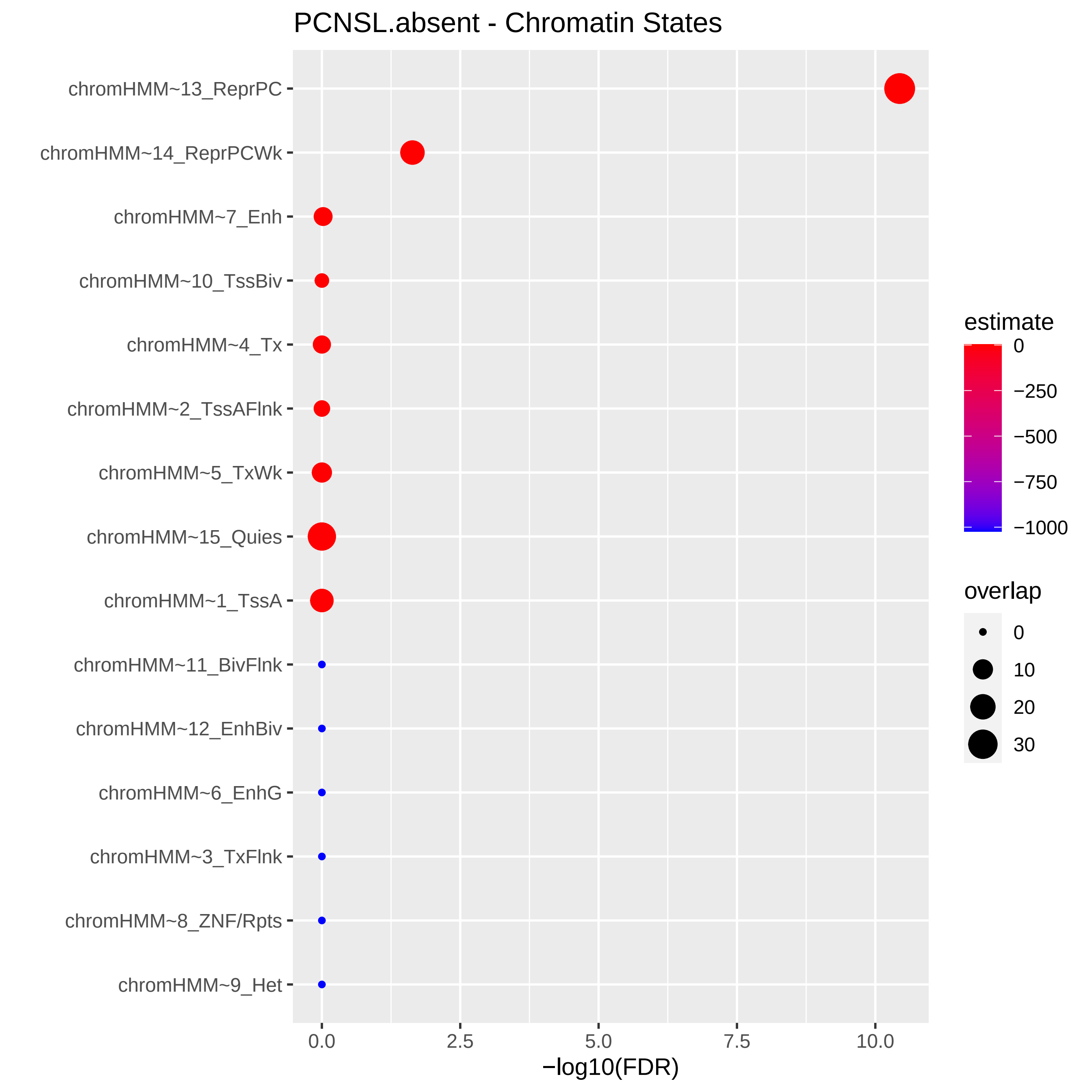

PCNSL.absent - Chromatin States
chromHMM~13_ReprPC
chromHMM~14_ReprPCWk
chromHMM~7_Enh
chromHMM~10_TssBiv
chromHMM~4_Tx
chromHMM~2_TssAFlnk
chromHMM~5_TxWk
chromHMM~15_Quies
chromHMM~1_TssA
chromHMM~11_BivFlnk
chromHMM~12_EnhBiv
chromHMM~6_EnhG
chromHMM~3_TxFlnk
chromHMM~8_ZNF/Rpts
chromHMM~9_Het
0.0
2.5
5.0
7.5
10.0
−log10(FDR)
estimate
0
−250
−500
−750
−1000
overlap
0
10
20
30

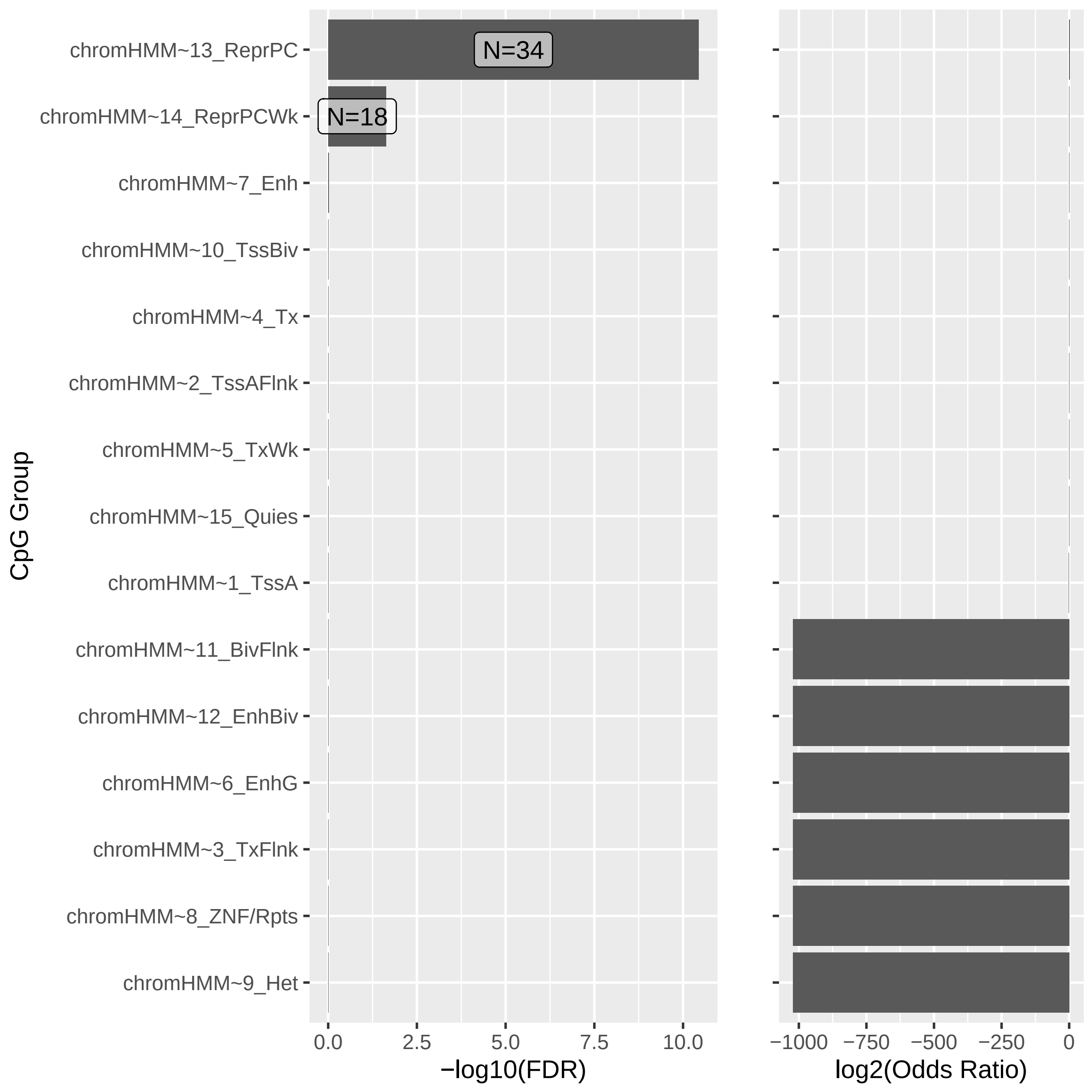

CpG Group
chromHMM~13_ReprPC
chromHMM~14_ReprPCWk
chromHMM~7_Enh
chromHMM~10_TssBiv
chromHMM~4_Tx
chromHMM~2_TssAFlnk
chromHMM~5_TxWk
chromHMM~15_Quies
chromHMM~1_TssA
chromHMM~11_BivFlnk
chromHMM~12_EnhBiv
chromHMM~6_EnhG
chromHMM~3_TxFlnk
chromHMM~8_ZNF/Rpts
chromHMM~9_Het
N=34
N=18
0.0
2.5
5.0
7.5
10.0
−log10(FDR)
−1000
−750
−500
−250
0
log2(Odds Ratio)

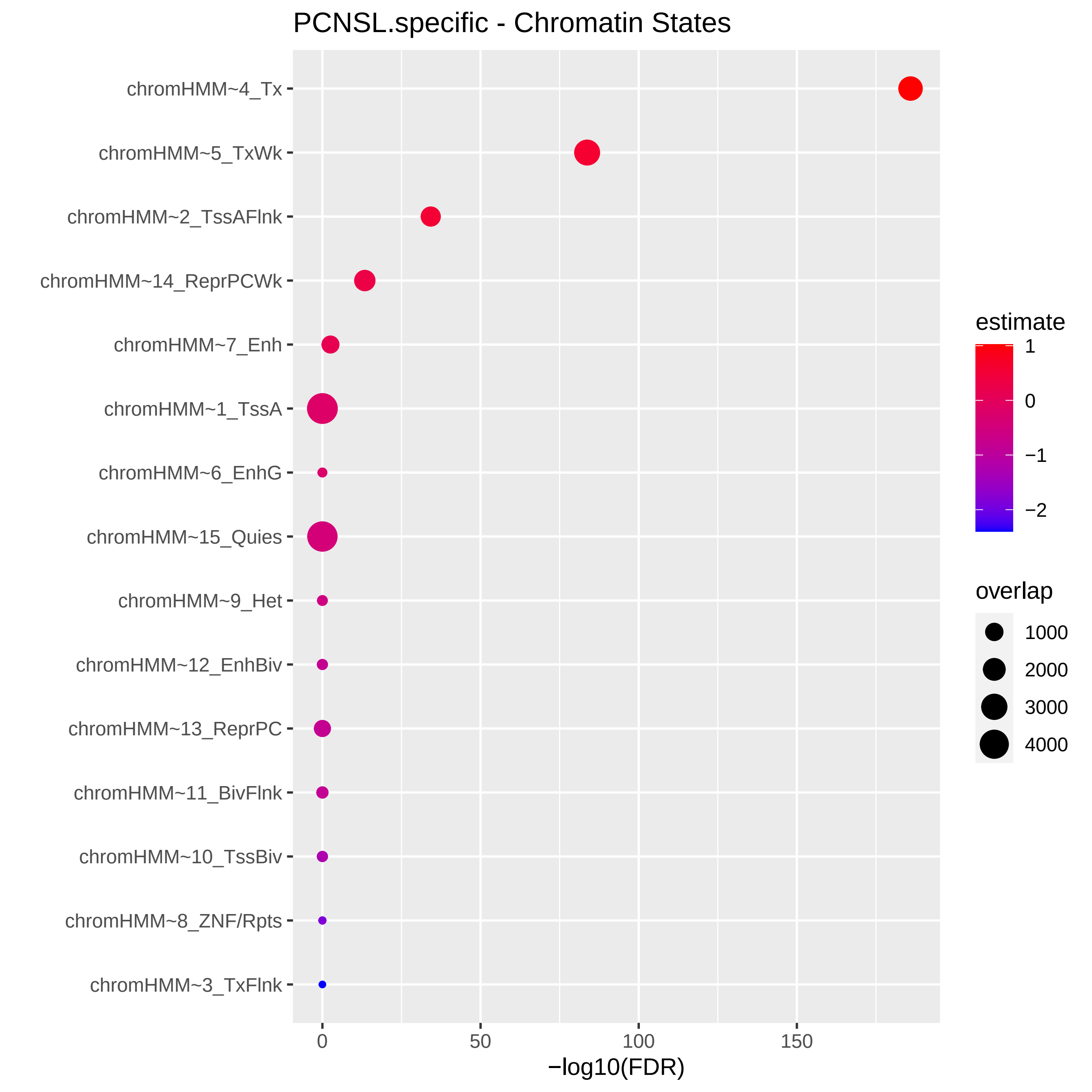
PCNSL.specific - Chromatin States
chromHMM~4_Tx
chromHMM~5_TxWk
chromHMM~2_TssAFlnk
chromHMM~14_ReprPCWk
chromHMM~7_Enh
chromHMM~1_TssA
chromHMM~6_EnhG
chromHMM~15_Quies
chromHMM~9_Het
chromHMM~12_EnhBiv
chromHMM~13_ReprPC
chromHMM~11_BivFlnk
chromHMM~10_TssBiv
chromHMM~8_ZNF/Rpts
chromHMM~3_TxFlnk
0
50
100
150
−log10(FDR)
estimate
1
0
−1
−2
overlap
1000
2000
3000
4000

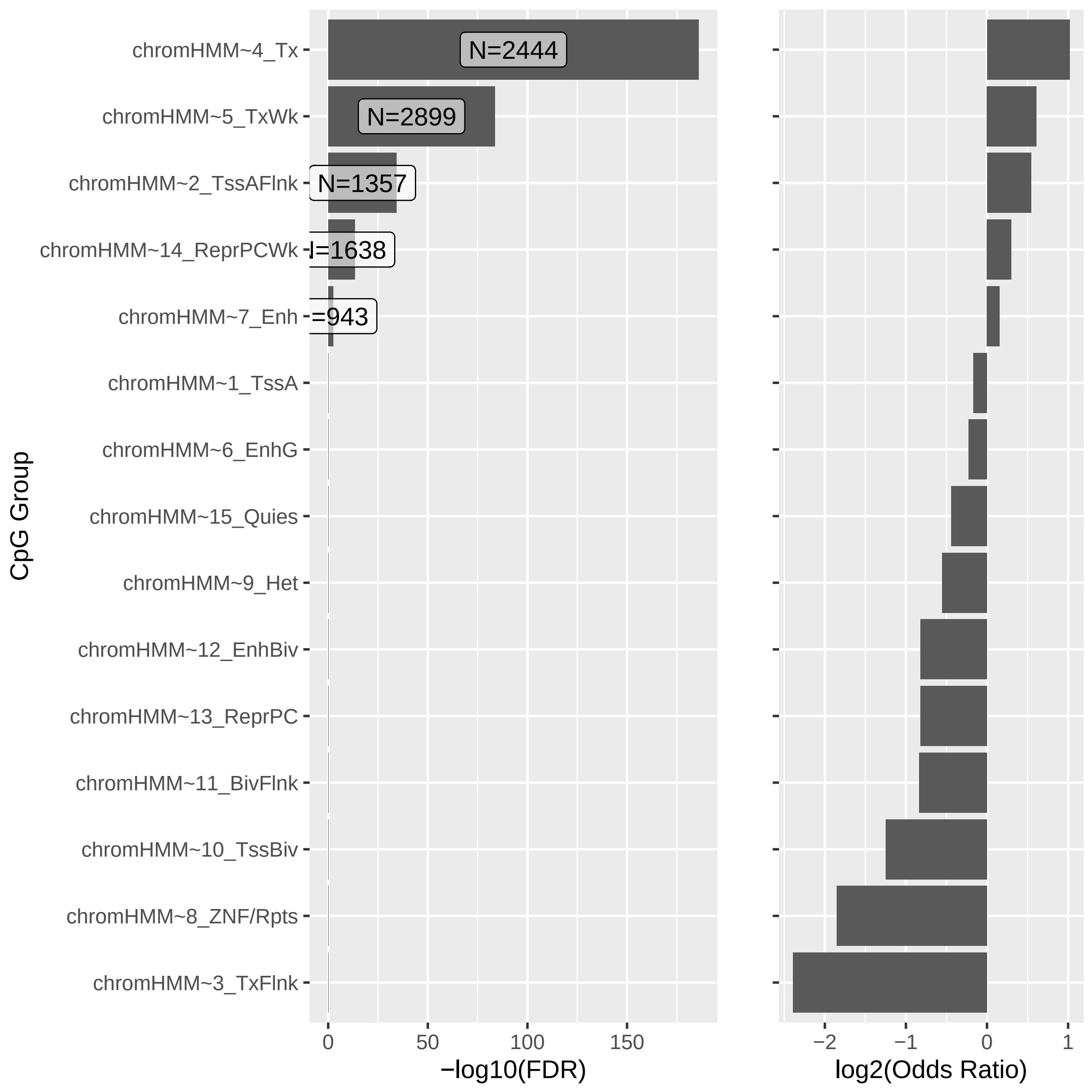

CpG Group
chromHMM~4_Tx
chromHMM~5_TxWk
chromHMM~2_TssAFlnk
chromHMM~14_ReprPCWk
chromHMM~7_Enh
chromHMM~1_TssA
chromHMM~6_EnhG
chromHMM~15_Quies
chromHMM~9_Het
chromHMM~12_EnhBiv
chromHMM~13_ReprPC
chromHMM~11_BivFlnk
chromHMM~10_TssBiv
chromHMM~8_ZNF/Rpts
chromHMM~3_TxFlnk
N=2444
N=2899
N=1357
=1638
=943
0
50
100
150
−log10(FDR)
−2
−1
0
1
log2(Odds Ratio)

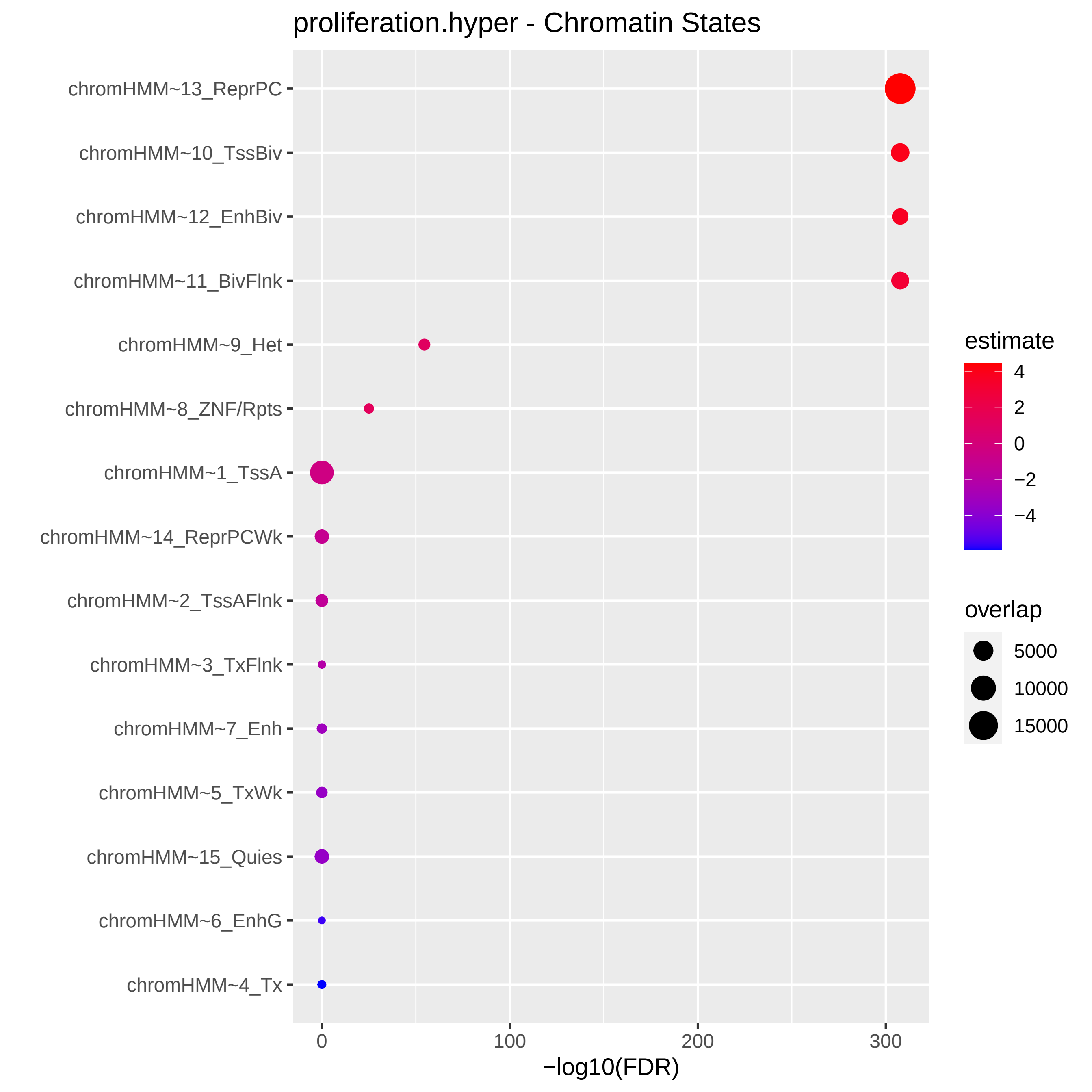
proliferation.hyper - Chromatin States
chromHMM~13_ReprPC
chromHMM~10_TssBiv
chromHMM~12_EnhBiv
chromHMM~11_BivFlnk
chromHMM~9_Het
chromHMM~8_ZNF/Rpts
chromHMM~1_TssA
chromHMM~14_ReprPCWk
chromHMM~2_TssAFlnk
chromHMM~3_TxFlnk
chromHMM~7_Enh
chromHMM~5_TxWk
chromHMM~15_Quies
chromHMM~6_EnhG
chromHMM~4_Tx
0
100
200
300
−log10(FDR)
estimate
4
2
0
−2
−4
overlap
5000
10000
15000

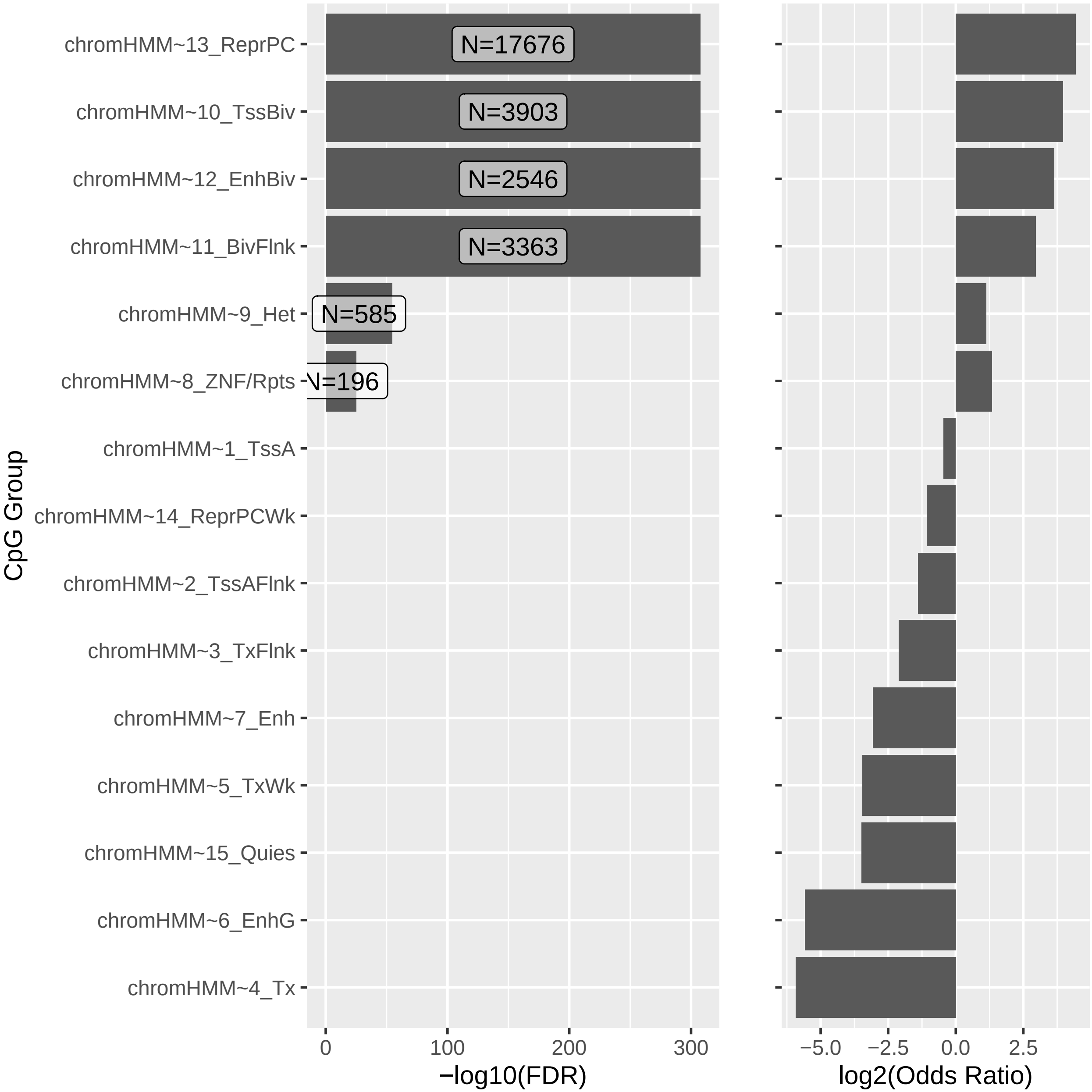

CpG Group
chromHMM~13_ReprPC
chromHMM~10_TssBiv
chromHMM~12_EnhBiv
chromHMM~11_BivFlnk
chromHMM~9_Het
chromHMM~8_ZNF/Rpts
chromHMM~1_TssA
chromHMM~14_ReprPCWk
chromHMM~2_TssAFlnk
chromHMM~3_TxFlnk
chromHMM~7_Enh
chromHMM~5_TxWk
chromHMM~15_Quies
chromHMM~6_EnhG
chromHMM~4_Tx
N=17676
N=3903
N=2546
N=3363
N=585
N=196
0
100
200
300
−log10(FDR)
−5.0
−2.5
0.0
2.5
log2(Odds Ratio)

Supplementary figure 3. Detailed results of SeSAMe analysis for chromatin states for the different DMR groups, as indicated. For each DMR group the first graph shows the significance of the association with the specific chromatin state adjusted for false discovery rate (−log10(FDR)). The second also includes details of the number of overlapping DMRs overlapping with the specific chromatin state feature.

Supplementary Figure 4

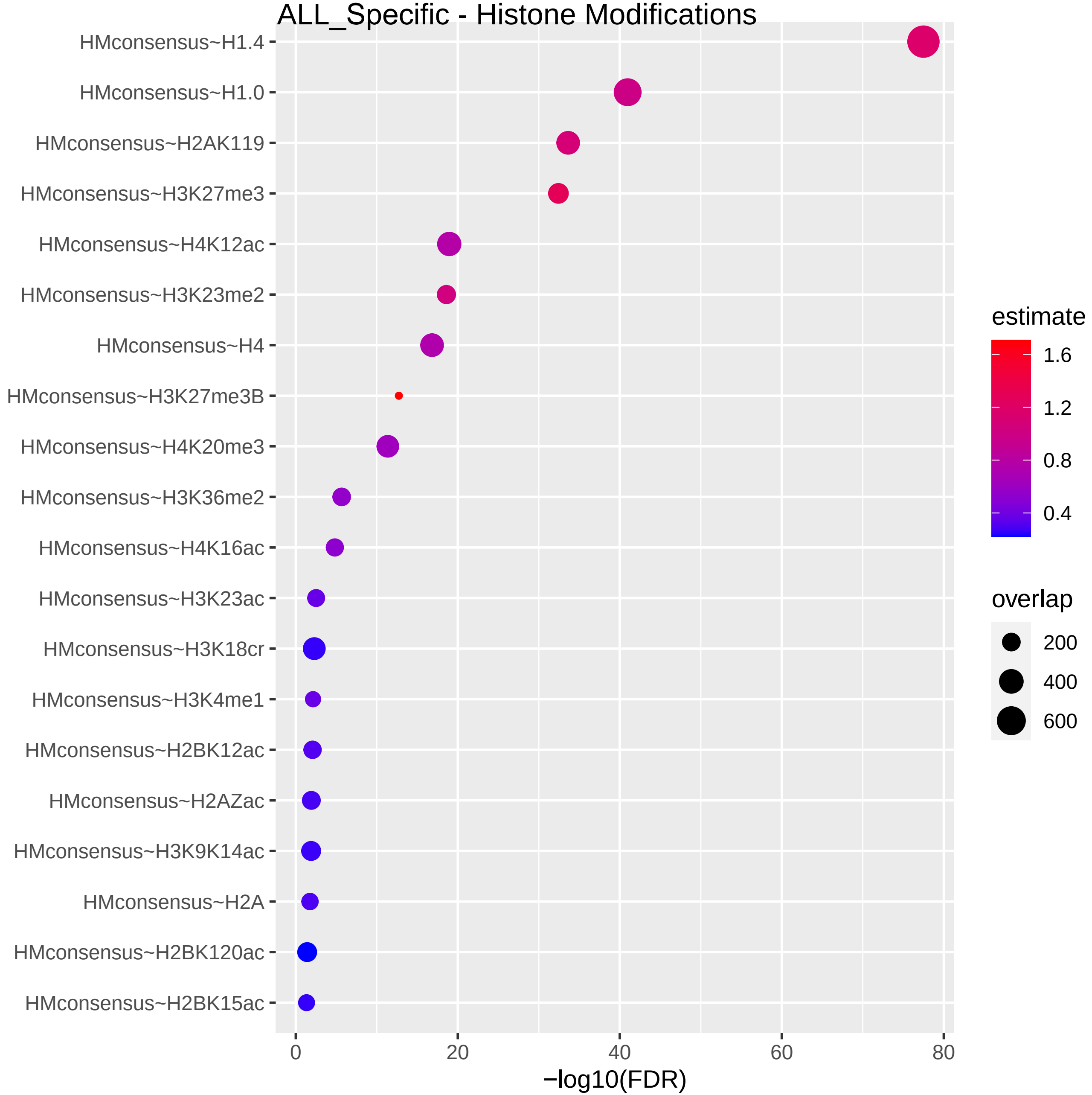

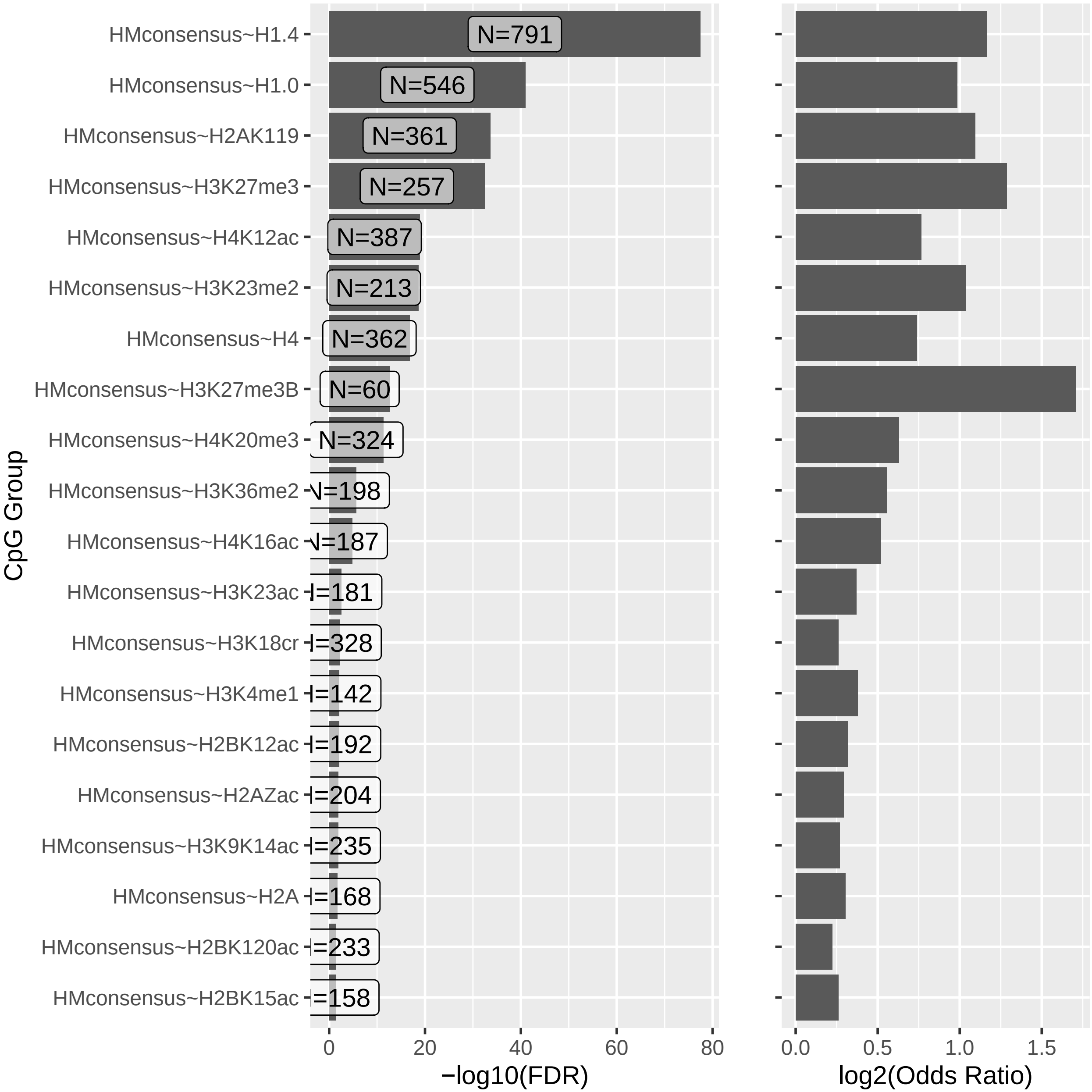

CpG Group
HMconsensus~H1.4
HMconsensus~H1.0
HMconsensus~H2AK119
HMconsensus~H3K27me3
HMconsensus~H4K12ac
HMconsensus~H3K23me2
HMconsensus~H4
HMconsensus~H3K27me3B
HMconsensus~H4K20me3
HMconsensus~H3K36me2
HMconsensus~H4K16ac
HMconsensus~H3K23ac
HMconsensus~H3K18cr
HMconsensus~H3K4me1
HMconsensus~H2BK12ac
HMconsensus~H2AZac
HMconsensus~H3K9K14ac
HMconsensus~H2A
HMconsensus~H2BK120ac
HMconsensus~H2BK15ac
N=791
N=546
N=361
N=257
N=387
N=213
N=362
N=60
N=324
0
20
40
60
80
−log10(FDR)
0.0
0.5
1.0
1.5
log2(Odds Ratio)

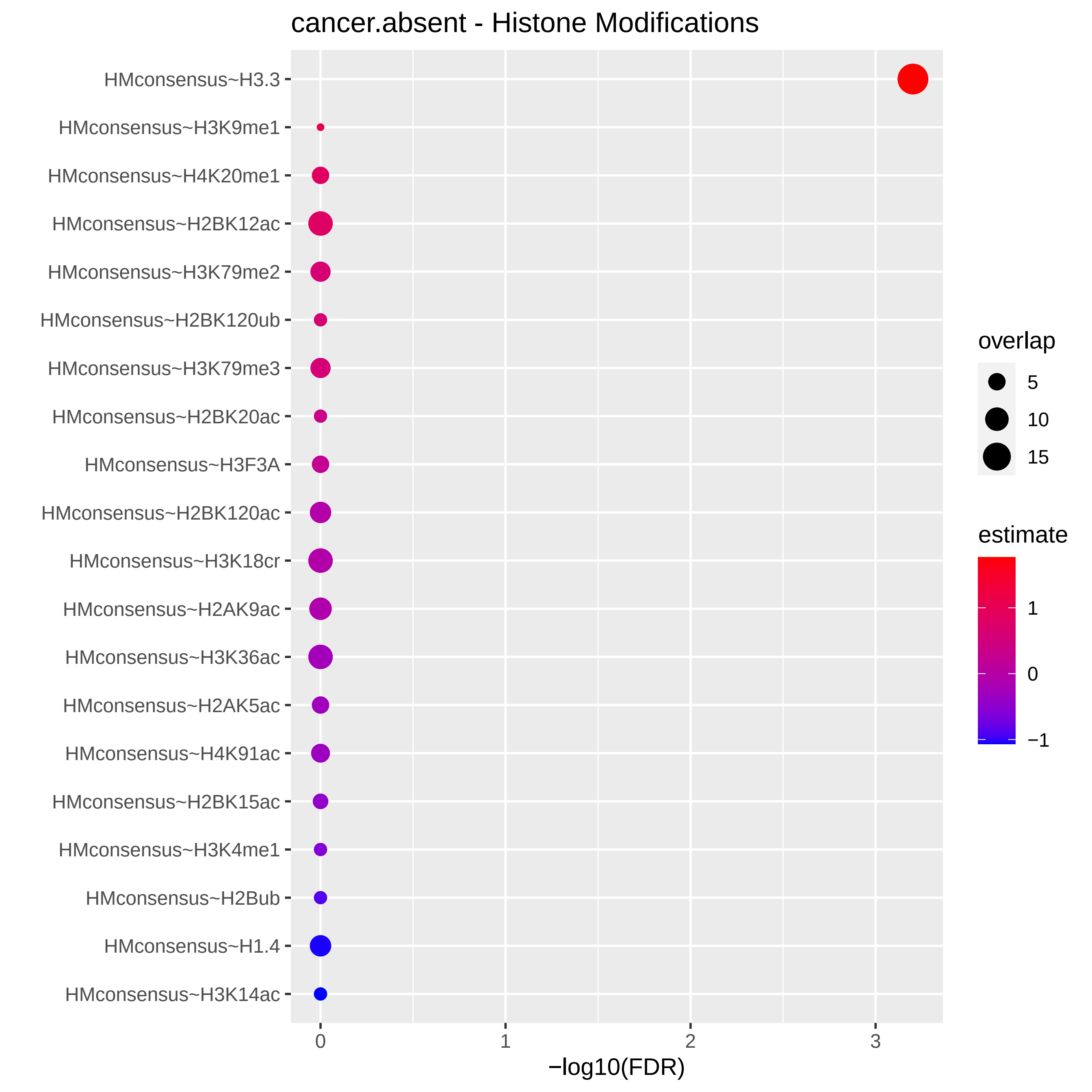

cancer.absent - Histone Modifications
HMconsensus~H3.3
HMconsensus~H3K9me1
HMconsensus~H4K20me1
HMconsensus~H2BK12ac
HMconsensus~H3K79me2
HMconsensus~H2BK120ub
HMconsensus~H3K79me3
HMconsensus~H2BK20ac
HMconsensus~H3F3A
HMconsensus~H2BK120ac
HMconsensus~H3K18cr
HMconsensus~H2AK9ac
HMconsensus~H3K36ac
HMconsensus~H2AK5ac
HMconsensus~H4K91ac
HMconsensus~H2BK15ac
HMconsensus~H3K4me1
HMconsensus~H2Bub
HMconsensus~H1.4
HMconsensus~H3K14ac
0
1
2
3
−log10(FDR)
overlap
5
10
15
estimate
1
0
−1

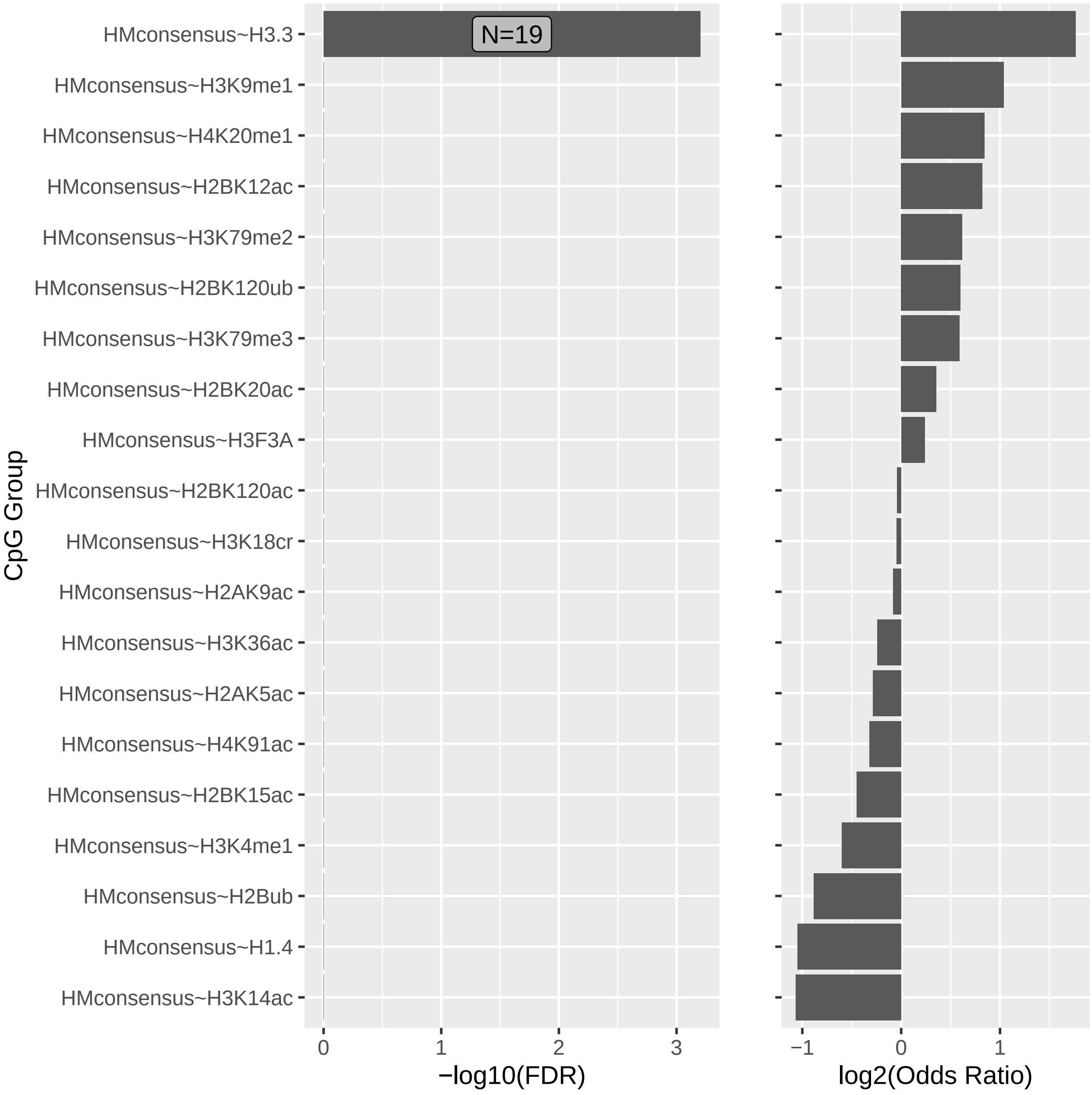
CpG Group
HMconsensus~H3.3
HMconsensus~H3K9me1
HMconsensus~H4K20me1
HMconsensus~H2BK12ac
HMconsensus~H3K79me2
HMconsensus~H2BK120ub
HMconsensus~H3K79me3
HMconsensus~H2BK20ac
HMconsensus~H3F3A
HMconsensus~H2BK120ac
HMconsensus~H3K18cr
HMconsensus~H2AK9ac
HMconsensus~H3K36ac
HMconsensus~H2AK5ac
HMconsensus~H4K91ac
HMconsensus~H2BK15ac
HMconsensus~H3K4me1
HMconsensus~H2Bub
HMconsensus~H1.4
HMconsensus~H3K14ac
N=19
0
1
2
3
−log10(FDR)
−1
0
1
log2(Odds Ratio)

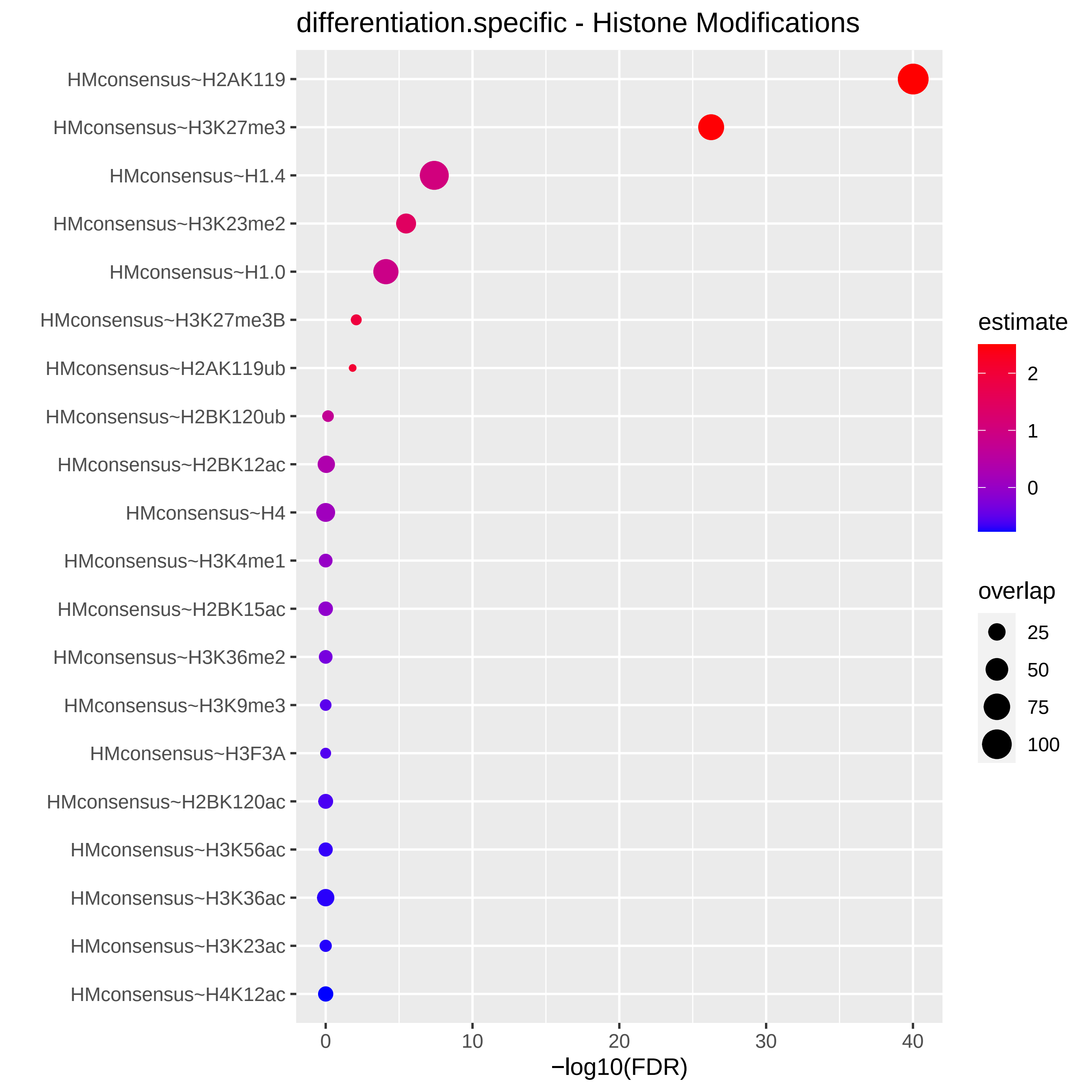

differentiation.specific - Histone Modifications
HMconsensus~H2AK119
HMconsensus~H3K27me3
HMconsensus~H1.4
HMconsensus~H3K23me2
HMconsensus~H1.0
HMconsensus~H3K27me3B
HMconsensus~H2AK119ub
HMconsensus~H2BK120ub
HMconsensus~H2BK12ac
HMconsensus~H4
HMconsensus~H3K4me1
HMconsensus~H2BK15ac
HMconsensus~H3K36me2
HMconsensus~H3K9me3
HMconsensus~H3F3A
HMconsensus~H2BK120ac
HMconsensus~H3K56ac
HMconsensus~H3K36ac
HMconsensus~H3K23ac
HMconsensus~H4K12ac
0
10
20
30
40
−log10(FDR)
estimate
2
1
0
overlap
25
50
75
100

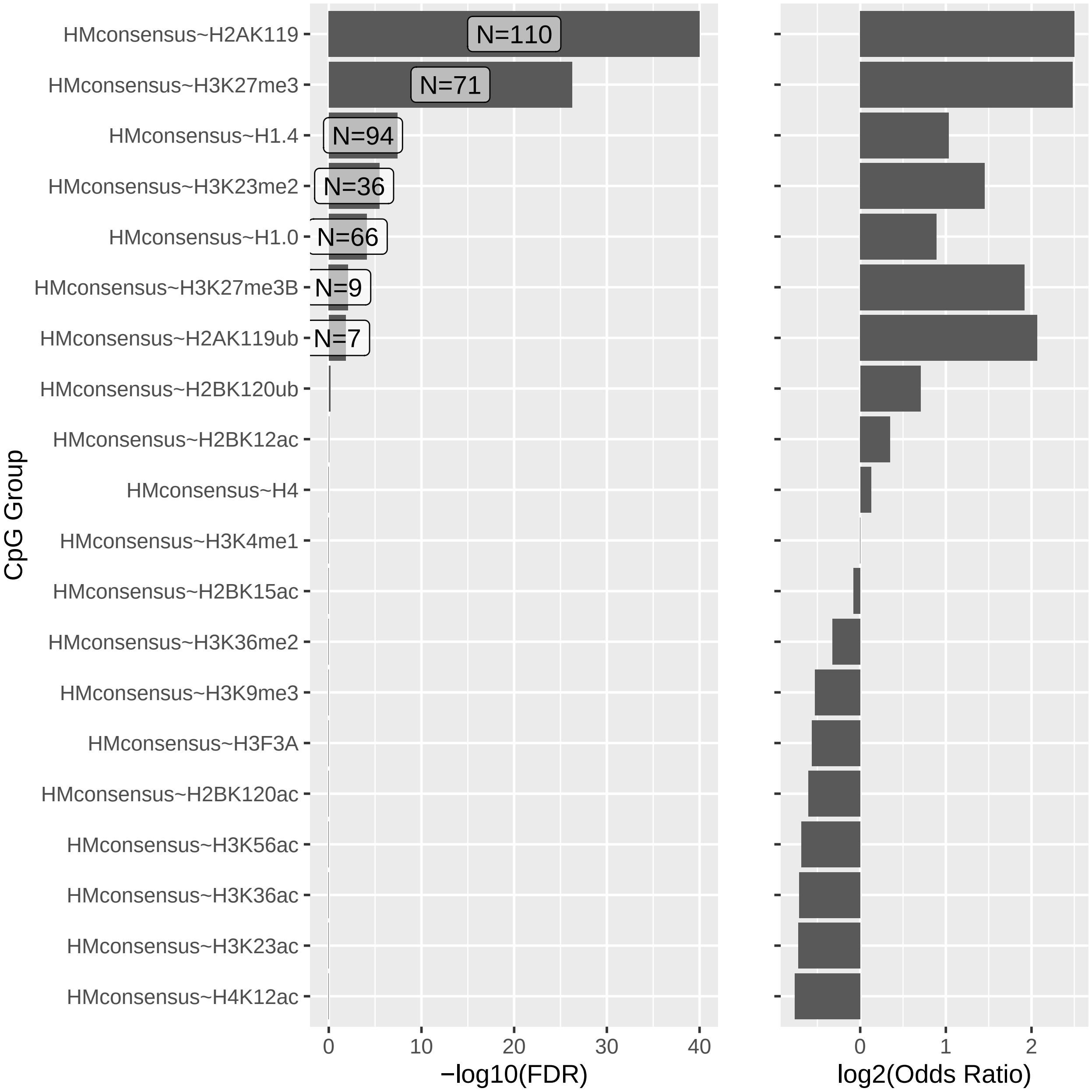

CpG Group
HMconsensus~H2AK119
HMconsensus~H3K27me3
HMconsensus~H1.4
HMconsensus~H3K23me2
HMconsensus~H1.0
HMconsensus~H3K27me3B
HMconsensus~H2AK119ub
HMconsensus~H2BK120ub
HMconsensus~H2BK12ac
HMconsensus~H4
HMconsensus~H3K4me1
HMconsensus~H2BK15ac
HMconsensus~H3K36me2
HMconsensus~H3K9me3
HMconsensus~H3F3A
HMconsensus~H2BK120ac
HMconsensus~H3K56ac
HMconsensus~H3K36ac
HMconsensus~H3K23ac
HMconsensus~H4K12ac
N=110
N=71
N=94
N=36
N=66
N=9
N=7
0
10
20
30
40
−log10(FDR)
0
1
2
log2(Odds Ratio)

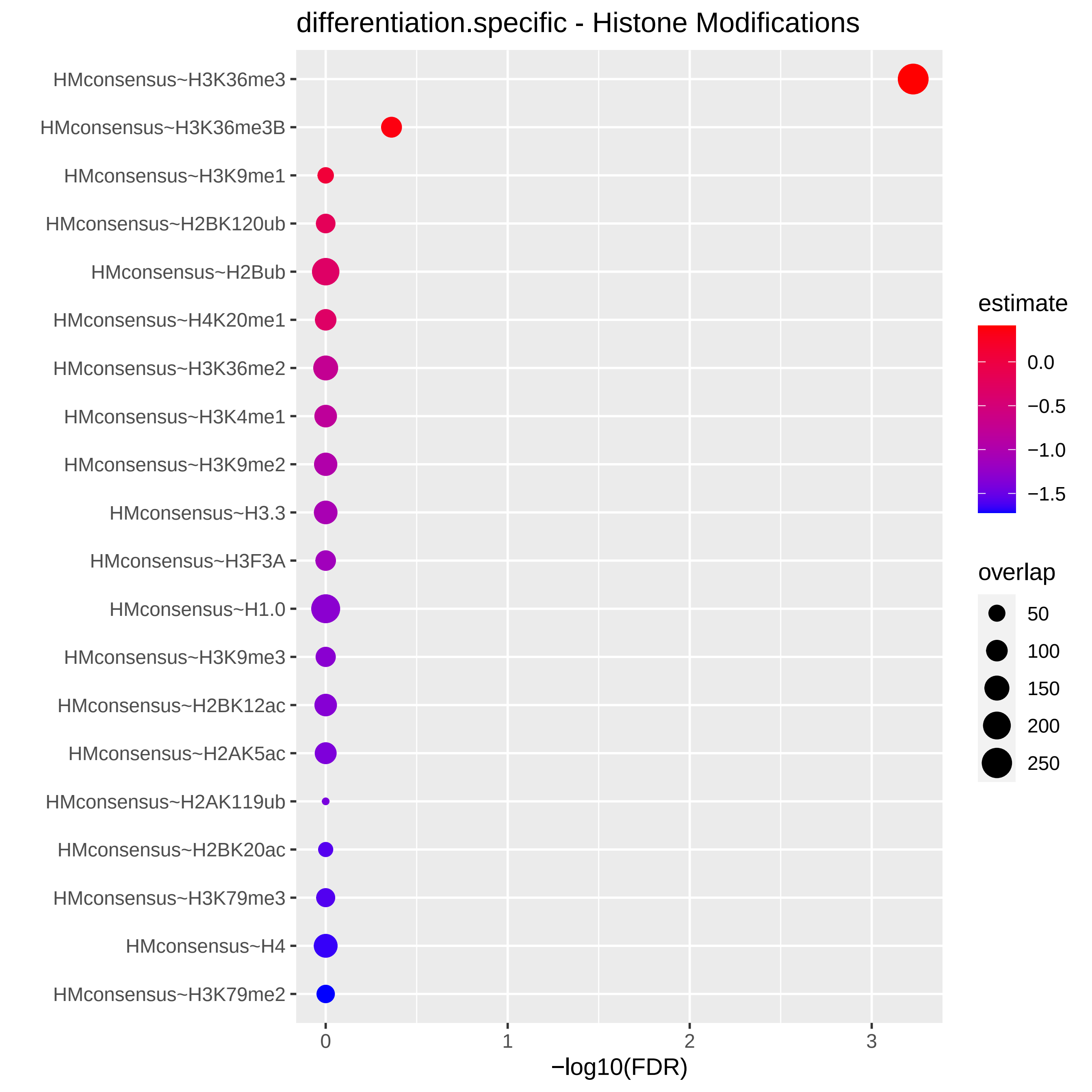
differentiation.specific - Histone Modifications
HMconsensus~H3K36me3
HMconsensus~H3K36me3B
HMconsensus~H3K9me1
HMconsensus~H2BK120ub
HMconsensus~H2Bub
HMconsensus~H4K20me1
HMconsensus~H3K36me2
HMconsensus~H3K4me1
HMconsensus~H3K9me2
HMconsensus~H3.3
HMconsensus~H3F3A
HMconsensus~H1.0
HMconsensus~H3K9me3
HMconsensus~H2BK12ac
HMconsensus~H2AK5ac
HMconsensus~H2AK119ub
HMconsensus~H2BK20ac
HMconsensus~H3K79me3
HMconsensus~H4
HMconsensus~H3K79me2
0
1
2
3
−log10(FDR)
estimate
0.0
−0.5
−1.0
−1.5
overlap
50
100
150
200
250

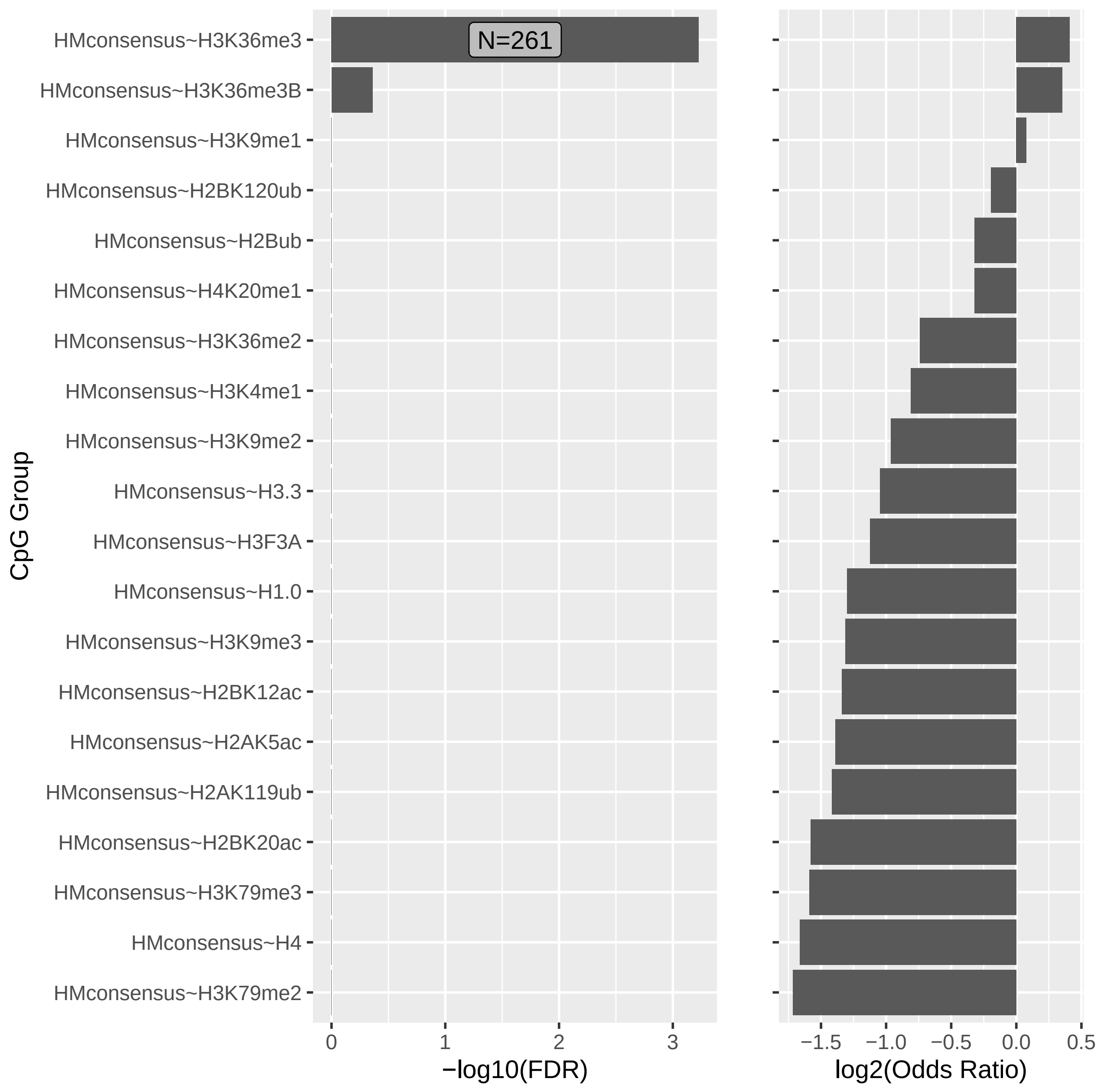

CpG Group
HMconsensus~H3K36me3
HMconsensus~H3K36me3B
HMconsensus~H3K9me1
HMconsensus~H2BK120ub
HMconsensus~H2Bub
HMconsensus~H4K20me1
HMconsensus~H3K36me2
HMconsensus~H3K4me1
HMconsensus~H3K9me2
HMconsensus~H3.3
HMconsensus~H3F3A
HMconsensus~H1.0
HMconsensus~H3K9me3
HMconsensus~H2BK12ac
HMconsensus~H2AK5ac
HMconsensus~H2AK119ub
HMconsensus~H2BK20ac
HMconsensus~H3K79me3
HMconsensus~H4
HMconsensus~H3K79me2
N=261
0
1
2
3
−log10(FDR)
−1.5
−1.0
−0.5
0.0
0.5
log2(Odds Ratio)

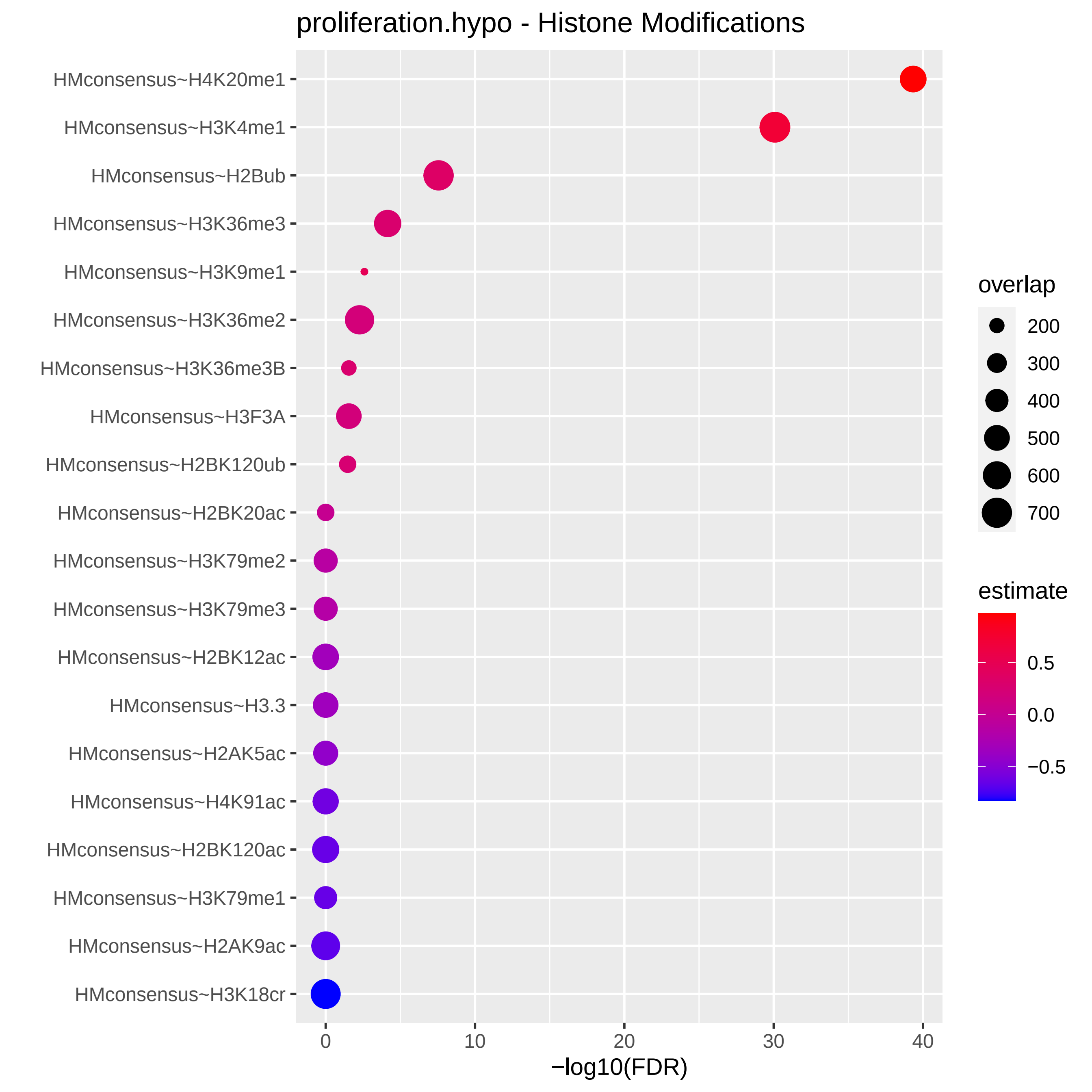

proliferation.hypo - Histone Modifications
HMconsensus~H4K20me1
HMconsensus~H3K4me1
HMconsensus~H2Bub
HMconsensus~H3K36me3
HMconsensus~H3K9me1
HMconsensus~H3K36me2
HMconsensus~H3K36me3B
HMconsensus~H3F3A
HMconsensus~H2BK120ub
HMconsensus~H2BK20ac
HMconsensus~H3K79me2
HMconsensus~H3K79me3
HMconsensus~H2BK12ac
HMconsensus~H3.3
HMconsensus~H2AK5ac
HMconsensus~H4K91ac
HMconsensus~H2BK120ac
HMconsensus~H3K79me1
HMconsensus~H2AK9ac
HMconsensus~H3K18cr
0
10
20
30
40
−log10(FDR)
overlap
200
300
400
500
600
700
estimate
0.5
0.0
−0.5

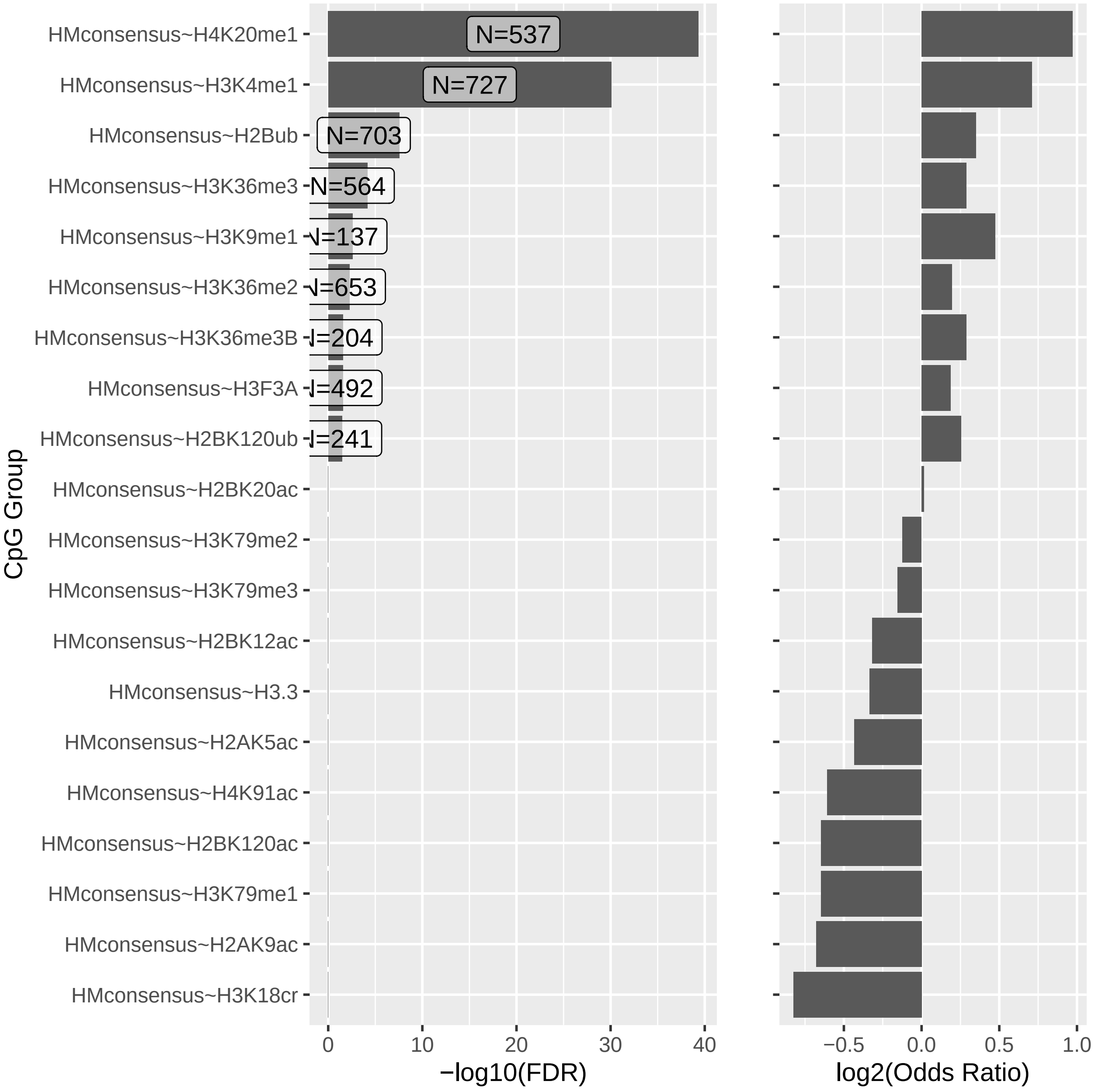

CpG Group
HMconsensus~H4K20me1
HMconsensus~H3K4me1
HMconsensus~H2Bub
HMconsensus~H3K36me3
HMconsensus~H3K9me1
HMconsensus~H3K36me2
HMconsensus~H3K36me3B
HMconsensus~H3F3A
HMconsensus~H2BK120ub
HMconsensus~H2BK20ac
HMconsensus~H3K79me2
HMconsensus~H3K79me3
HMconsensus~H2BK12ac
HMconsensus~H3.3
HMconsensus~H2AK5ac
HMconsensus~H4K91ac
HMconsensus~H2BK120ac
HMconsensus~H3K79me1
HMconsensus~H2AK9ac
HMconsensus~H3K18cr
N=537
N=727
N=703
N=564
N=137
N=653
N=204
N=492
N=241
0
10
20
30
40
−log10(FDR)
−0.5
0.0
0.5
1.0
log2(Odds Ratio)

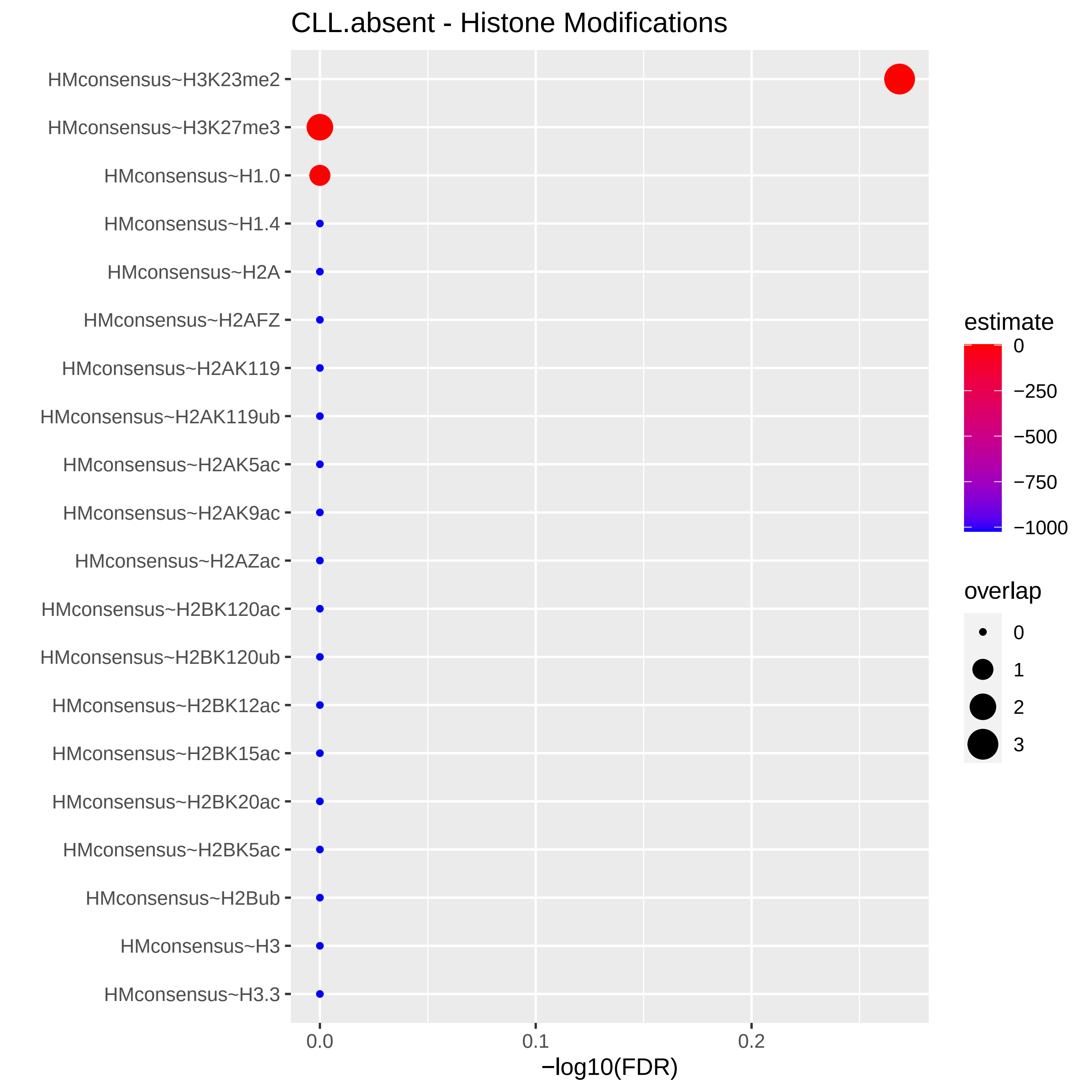
CLL.absent - Histone Modifications
HMconsensus~H3K23me2
HMconsensus~H3K27me3
HMconsensus~H1.0
HMconsensus~H1.4
HMconsensus~H2A
HMconsensus~H2AFZ
HMconsensus~H2AK119
HMconsensus~H2AK119ub
HMconsensus~H2AK5ac
HMconsensus~H2AK9ac
HMconsensus~H2AZac
HMconsensus~H2BK120ac
HMconsensus~H2BK120ub
HMconsensus~H2BK12ac
HMconsensus~H2BK15ac
HMconsensus~H2BK20ac
HMconsensus~H2BK5ac
HMconsensus~H2Bub
HMconsensus~H3
HMconsensus~H3.3
0.0
0.1
0.2
−log10(FDR)
estimate
0
−250
−500
−750
−1000
overlap
0
1
2
3

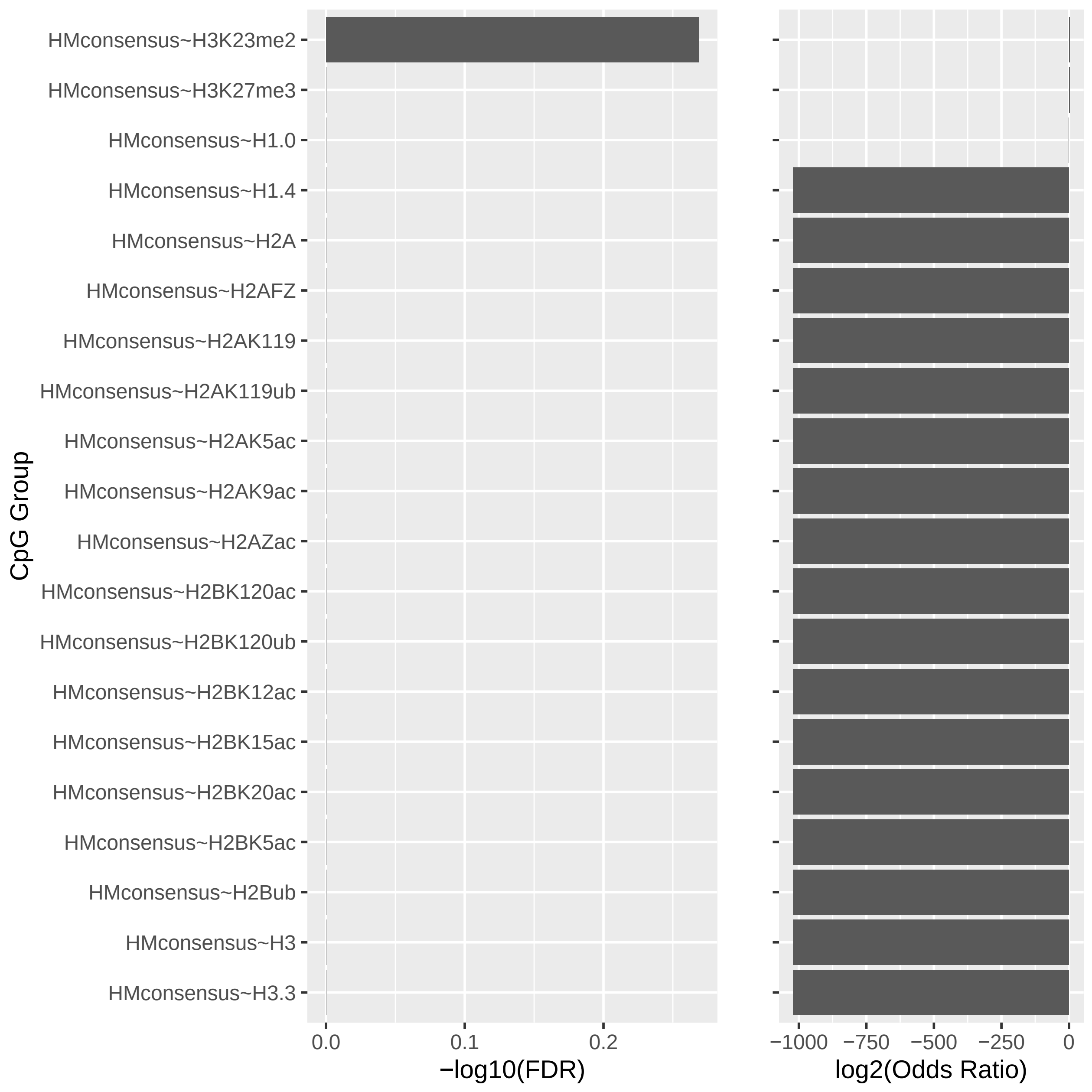

CpG Group
HMconsensus~H3K23me2
HMconsensus~H3K27me3
HMconsensus~H1.0
HMconsensus~H1.4
HMconsensus~H2A
HMconsensus~H2AFZ
HMconsensus~H2AK119
HMconsensus~H2AK119ub
HMconsensus~H2AK5ac
HMconsensus~H2AK9ac
HMconsensus~H2AZac
HMconsensus~H2BK120ac
HMconsensus~H2BK120ub
HMconsensus~H2BK12ac
HMconsensus~H2BK15ac
HMconsensus~H2BK20ac
HMconsensus~H2BK5ac
HMconsensus~H2Bub
HMconsensus~H3
HMconsensus~H3.3
0.0
0.1
0.2
−log10(FDR)
−1000
−750
−500
−250
0
log2(Odds Ratio)

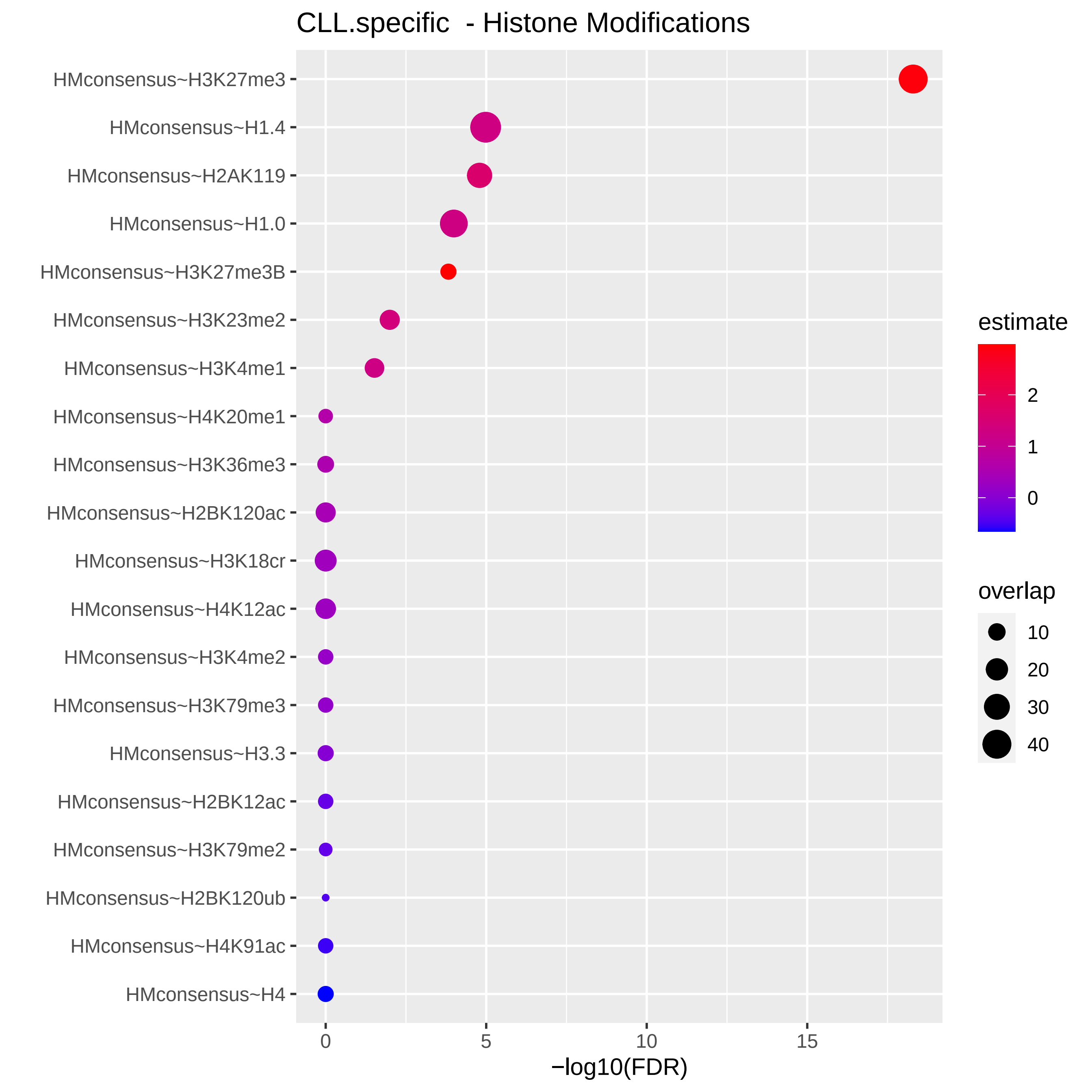

CLL.specific - Histone Modifications
HMconsensus~H3K27me3
HMconsensus~H1.4
HMconsensus~H2AK119
HMconsensus~H1.0
HMconsensus~H3K27me3B
HMconsensus~H3K23me2
HMconsensus~H3K4me1
HMconsensus~H4K20me1
HMconsensus~H3K36me3
HMconsensus~H2BK120ac
HMconsensus~H3K18cr
HMconsensus~H4K12ac
HMconsensus~H3K4me2
HMconsensus~H3K79me3
HMconsensus~H3.3
HMconsensus~H2BK12ac
HMconsensus~H3K79me2
HMconsensus~H2BK120ub
HMconsensus~H4K91ac
HMconsensus~H4
0
5
10
15
−log10(FDR)
estimate
2
1
0
overlap
10
20
30
40

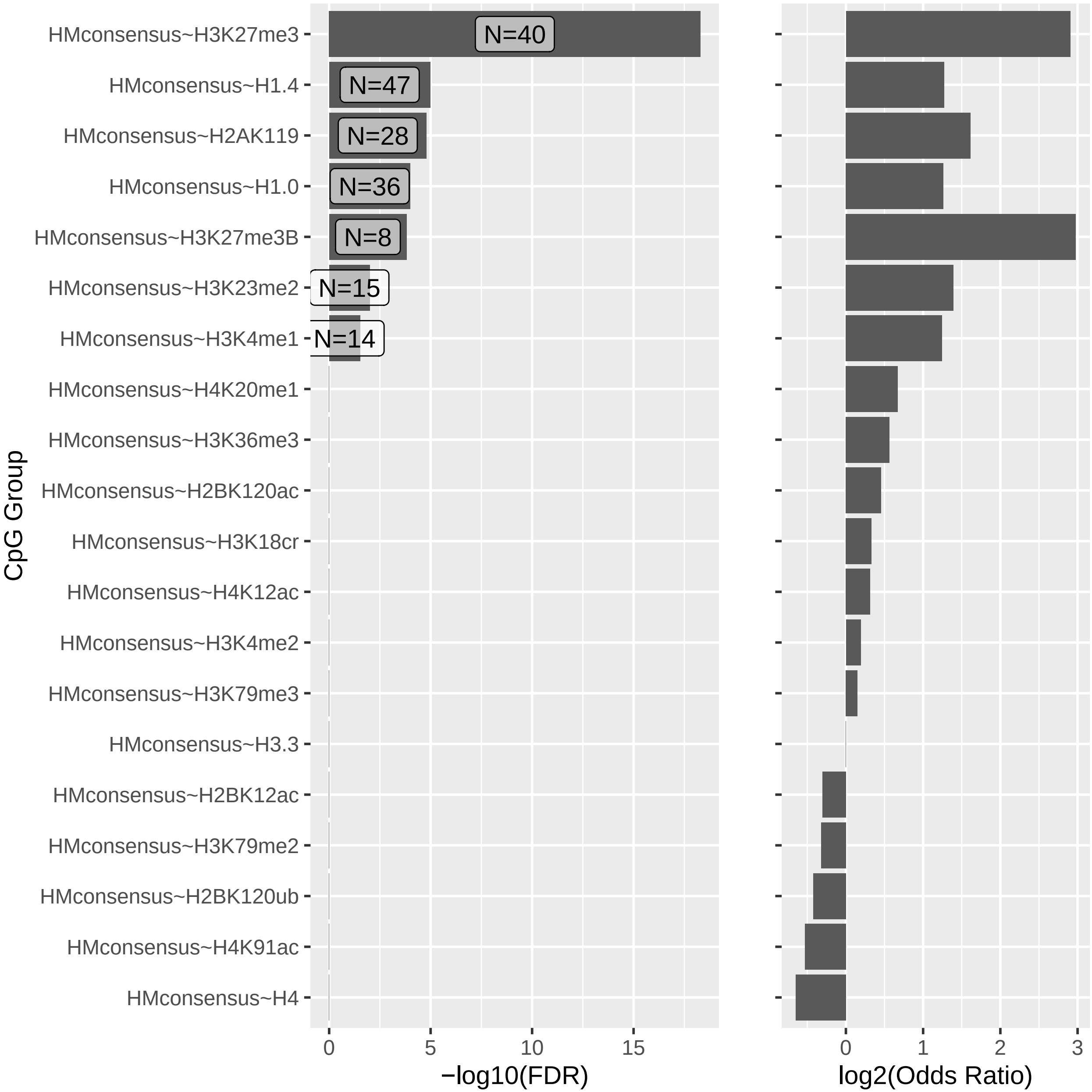
CpG Group
HMconsensus~H3K27me3
HMconsensus~H1.4
HMconsensus~H2AK119
HMconsensus~H1.0
HMconsensus~H3K27me3B
HMconsensus~H3K23me2
HMconsensus~H3K4me1
HMconsensus~H4K20me1
HMconsensus~H3K36me3
HMconsensus~H2BK120ac
HMconsensus~H3K18cr
HMconsensus~H4K12ac
HMconsensus~H3K4me2
HMconsensus~H3K79me3
HMconsensus~H3.3
HMconsensus~H2BK12ac
HMconsensus~H3K79me2
HMconsensus~H2BK120ub
HMconsensus~H4K91ac
HMconsensus~H4
N=40
N=47
N=28
N=36
N=8
N=15
N=14
0
5
10
15
−log10(FDR)
0
1
2
3
log2(Odds Ratio)

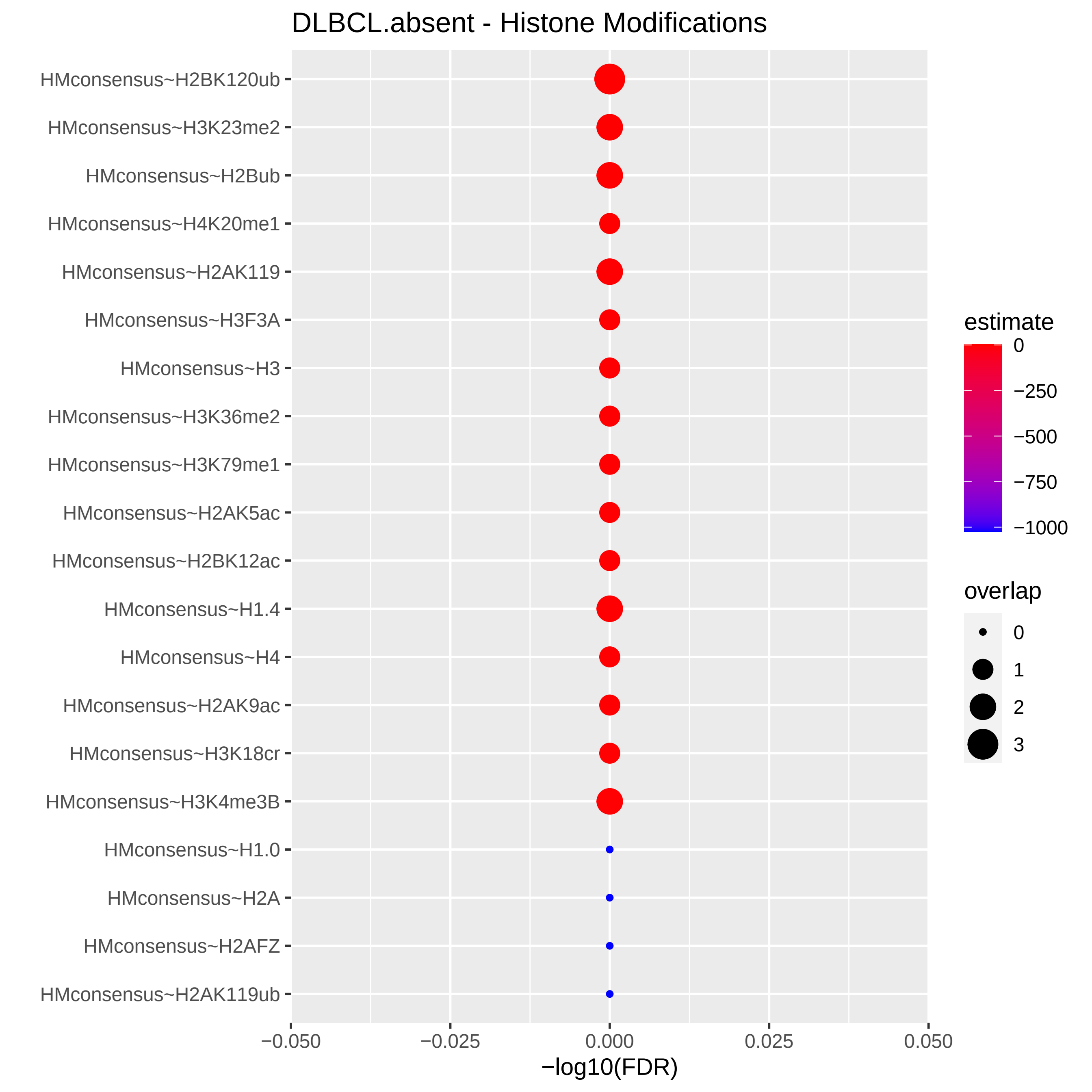
DLBCL.absent - Histone Modifications
HMconsensus~H2BK120ub
HMconsensus~H3K23me2
HMconsensus~H2Bub
HMconsensus~H4K20me1
HMconsensus~H2AK119
HMconsensus~H3F3A
HMconsensus~H3
HMconsensus~H3K36me2
HMconsensus~H3K79me1
HMconsensus~H2AK5ac
HMconsensus~H2BK12ac
HMconsensus~H1.4
HMconsensus~H4
HMconsensus~H2AK9ac
HMconsensus~H3K18cr
HMconsensus~H3K4me3B
HMconsensus~H1.0
HMconsensus~H2A
HMconsensus~H2AFZ
HMconsensus~H2AK119ub
−0.050
−0.025
0.000
0.025
0.050
−log10(FDR)
estimate
0
−250
−500
−750
−1000
overlap
0
1
2
3

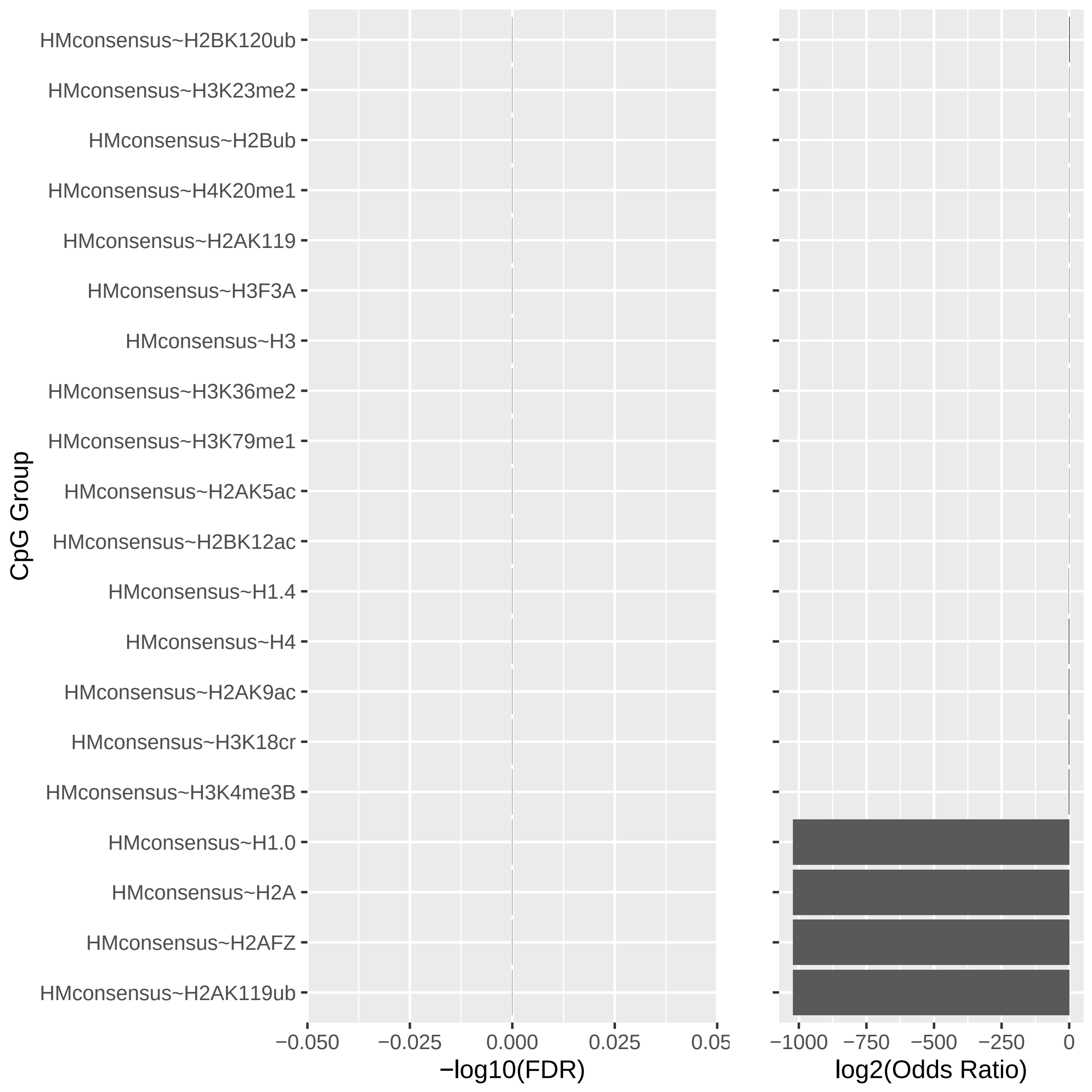

HMconsensus~H2BK120ub
HMconsensus~H3K23me2
HMconsensus~H2Bub
HMconsensus~H4K20me1
HMconsensus~H2AK119
HMconsensus~H3F3A
HMconsensus~H3
HMconsensus~H3K36me2
HMconsensus~H3K79me1
HMconsensus~H2AK5ac
HMconsensus~H2BK12ac
HMconsensus~H1.4
HMconsensus~H4
HMconsensus~H2AK9ac
HMconsensus~H3K18cr
HMconsensus~H3K4me3B
HMconsensus~H1.0
HMconsensus~H2A
HMconsensus~H2AFZ
HMconsensus~H2AK119ub
CpG Group
−0.050
−0.025
0.000
0.025
0.05
−log10(FDR)
−1000
−750
−500
−250
0
log2(Odds Ratio)

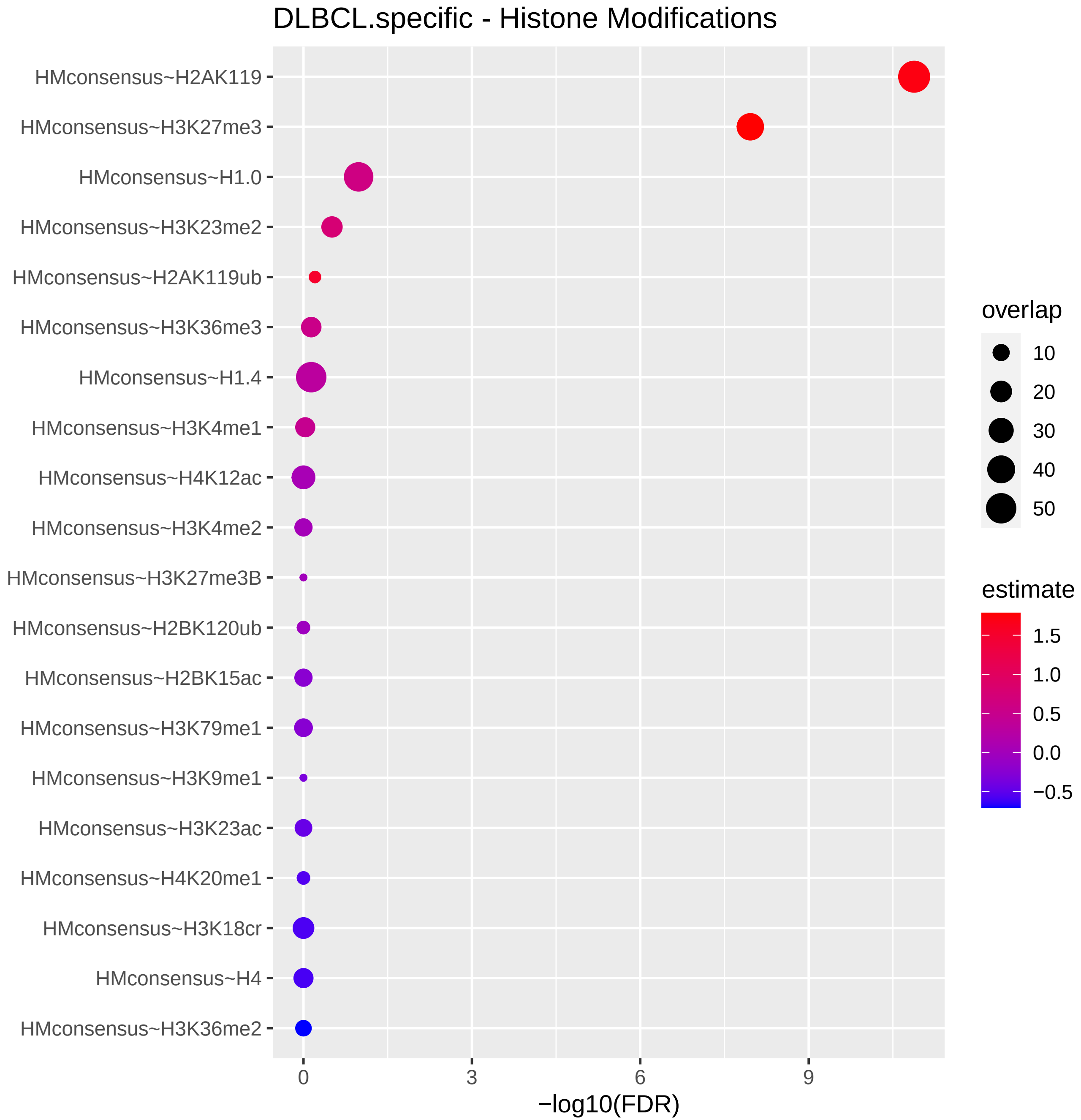

DLBCL.specific - Histone Modifications
HMconsensus~H2AK119
HMconsensus~H3K27me3
HMconsensus~H1.0
HMconsensus~H3K23me2
HMconsensus~H2AK119ub
HMconsensus~H3K36me3
HMconsensus~H1.4
HMconsensus~H3K4me1
HMconsensus~H4K12ac
HMconsensus~H3K4me2
HMconsensus~H3K27me3B
HMconsensus~H2BK120ub
HMconsensus~H2BK15ac
HMconsensus~H3K79me1
HMconsensus~H3K9me1
HMconsensus~H3K23ac
HMconsensus~H4K20me1
HMconsensus~H3K18cr
HMconsensus~H4
HMconsensus~H3K36me2
0
3
6
9
−log10(FDR)
overlap
10
20
30
40
50
estimate
1.5
1.0
0.5
0.0
−0.5

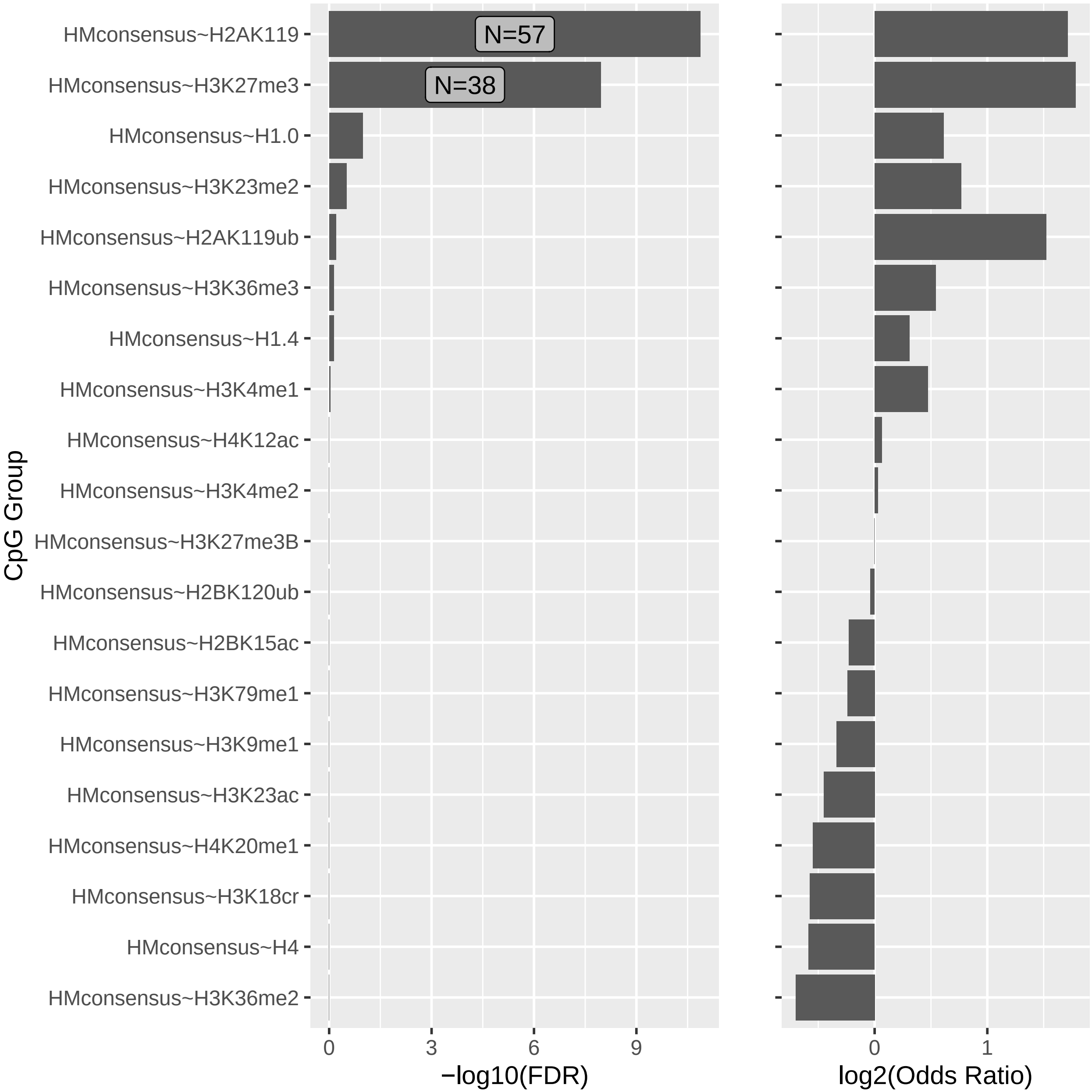

CpG Group
HMconsensus~H2AK119
HMconsensus~H3K27me3
HMconsensus~H1.0
HMconsensus~H3K23me2
HMconsensus~H2AK119ub
HMconsensus~H3K36me3
HMconsensus~H1.4
HMconsensus~H3K4me1
HMconsensus~H4K12ac
HMconsensus~H3K4me2
HMconsensus~H3K27me3B
HMconsensus~H2BK120ub
HMconsensus~H2BK15ac
HMconsensus~H3K79me1
HMconsensus~H3K9me1
HMconsensus~H3K23ac
HMconsensus~H4K20me1
HMconsensus~H3K18cr
HMconsensus~H4
HMconsensus~H3K36me2
N=57
N=38
0
3
6
9
−log10(FDR)
0
1
log2(Odds Ratio)

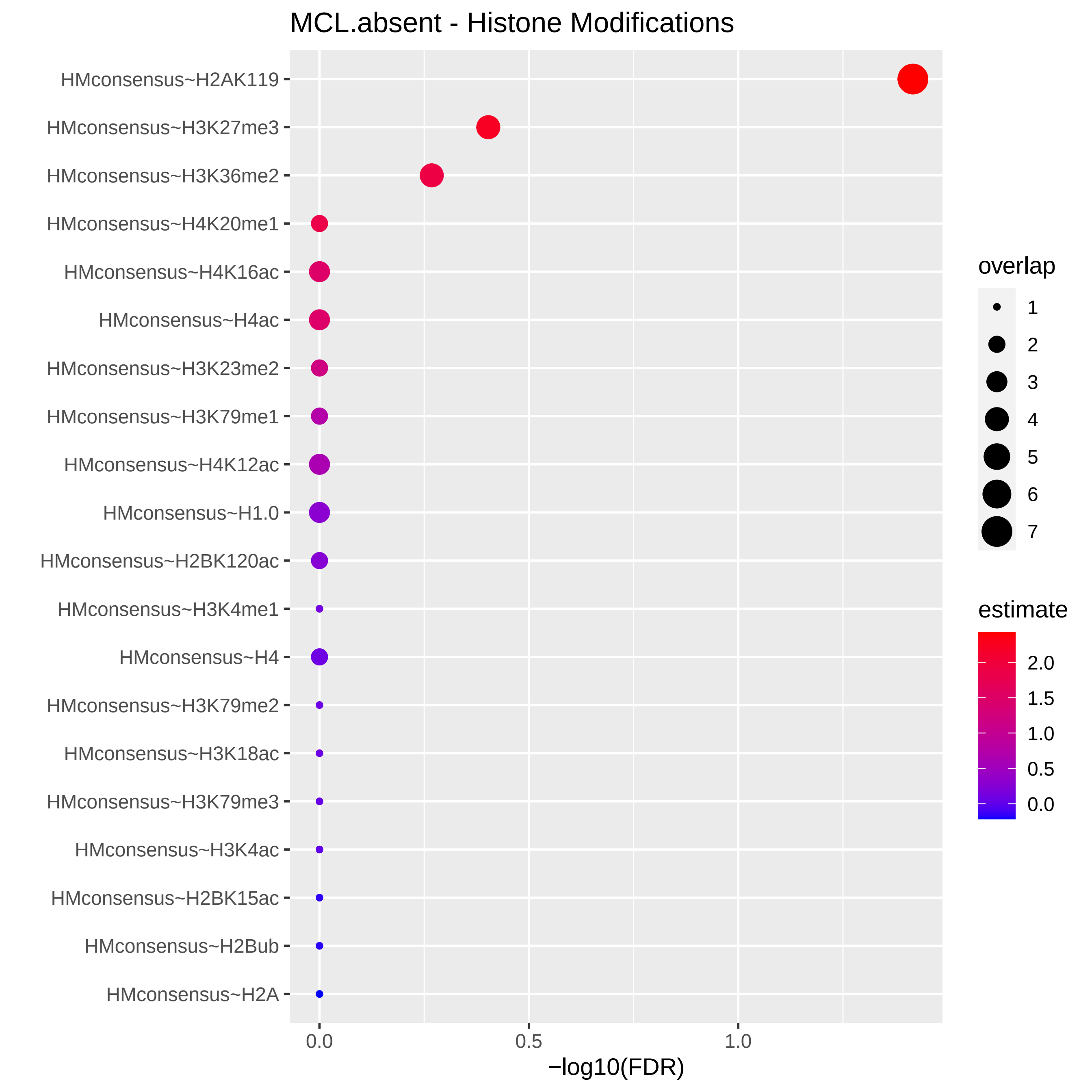
MCL.absent - Histone Modifications
HMconsensus~H2AK119
HMconsensus~H3K27me3
HMconsensus~H3K36me2
HMconsensus~H4K20me1
HMconsensus~H4K16ac
HMconsensus~H4ac
HMconsensus~H3K23me2
HMconsensus~H3K79me1
HMconsensus~H4K12ac
HMconsensus~H1.0
HMconsensus~H2BK120ac
HMconsensus~H3K4me1
HMconsensus~H4
HMconsensus~H3K79me2
HMconsensus~H3K18ac
HMconsensus~H3K79me3
HMconsensus~H3K4ac
HMconsensus~H2BK15ac
HMconsensus~H2Bub
HMconsensus~H2A
0.0
0.5
1.0
−log10(FDR)
overlap
1
2
3
4
5
6
7
estimate
2.0
1.5
1.0
0.5
0.0

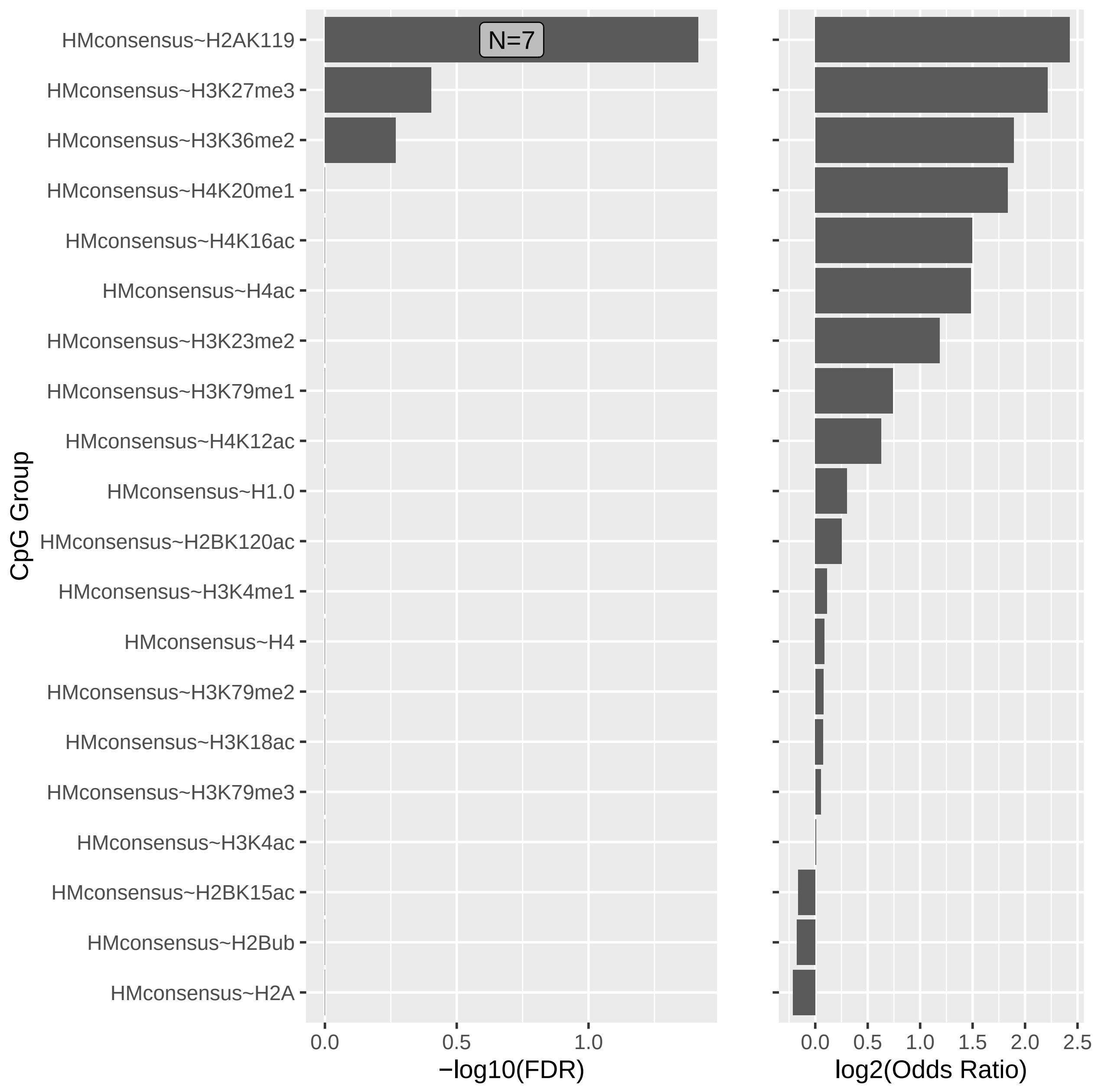

HMconsensus~H2AK119
HMconsensus~H3K27me3
HMconsensus~H3K36me2
HMconsensus~H4K20me1
HMconsensus~H4K16ac
HMconsensus~H4ac
HMconsensus~H3K23me2
HMconsensus~H3K79me1
HMconsensus~H4K12ac
HMconsensus~H1.0
HMconsensus~H2BK120ac
HMconsensus~H3K4me1
HMconsensus~H4
HMconsensus~H3K79me2
HMconsensus~H3K18ac
HMconsensus~H3K79me3
HMconsensus~H3K4ac
HMconsensus~H2BK15ac
HMconsensus~H2Bub
HMconsensus~H2A
CpG Group
N=7
0.0
0.5
1.0
−log10(FDR)
0.0
0.5
1.0
1.5
2.0
2.5
log2(Odds Ratio)

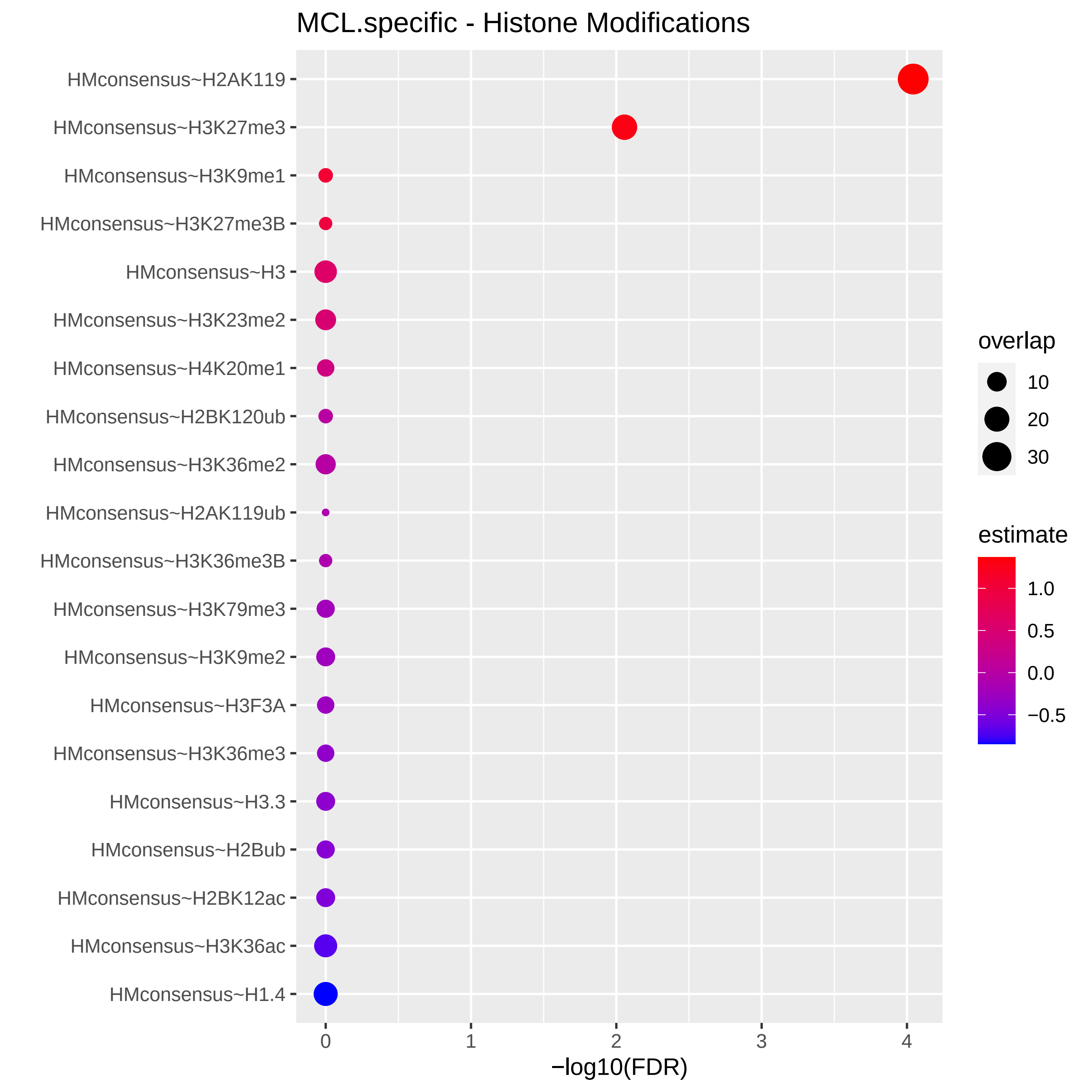

MCL.specific - Histone Modifications
HMconsensus~H2AK119
HMconsensus~H3K27me3
HMconsensus~H3K9me1
HMconsensus~H3K27me3B
HMconsensus~H3
HMconsensus~H3K23me2
HMconsensus~H4K20me1
HMconsensus~H2BK120ub
HMconsensus~H3K36me2
HMconsensus~H2AK119ub
HMconsensus~H3K36me3B
HMconsensus~H3K79me3
HMconsensus~H3K9me2
HMconsensus~H3F3A
HMconsensus~H3K36me3
HMconsensus~H3.3
HMconsensus~H2Bub
HMconsensus~H2BK12ac
HMconsensus~H3K36ac
HMconsensus~H1.4
0
1
2
3
4
−log10(FDR)
overlap
10
20
30
estimate
1.0
0.5
0.0
−0.5

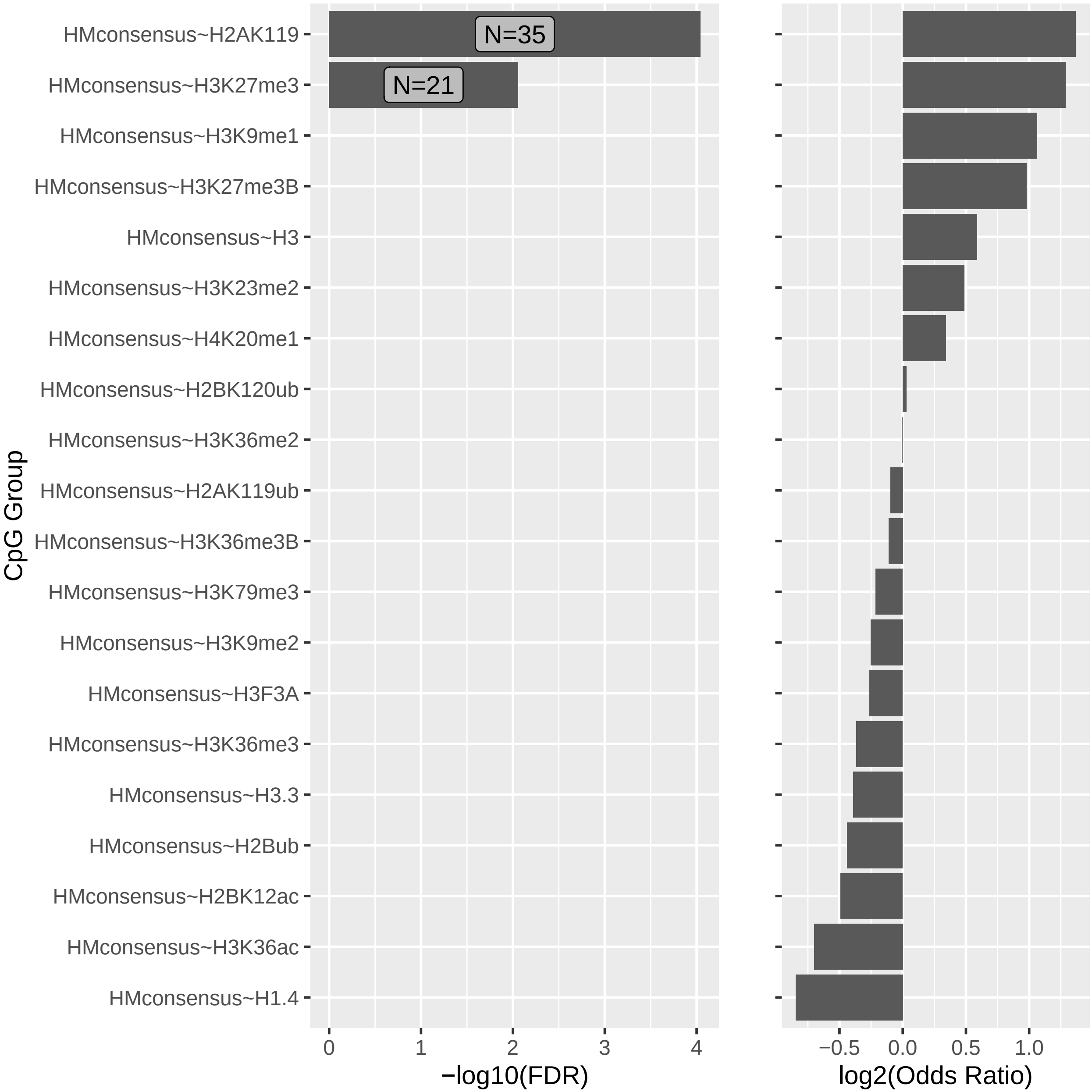

CpG Group
HMconsensus~H2AK119
HMconsensus~H3K27me3
HMconsensus~H3K9me1
HMconsensus~H3K27me3B
HMconsensus~H3
HMconsensus~H3K23me2
HMconsensus~H4K20me1
HMconsensus~H2BK120ub
HMconsensus~H3K36me2
HMconsensus~H2AK119ub
HMconsensus~H3K36me3B
HMconsensus~H3K79me3
HMconsensus~H3K9me2
HMconsensus~H3F3A
HMconsensus~H3K36me3
HMconsensus~H3.3
HMconsensus~H2Bub
HMconsensus~H2BK12ac
HMconsensus~H3K36ac
HMconsensus~H1.4
N=35
N=21
0
1
2
3
4
−log10(FDR)
−0.5
0.0
0.5
1.0
log2(Odds Ratio)

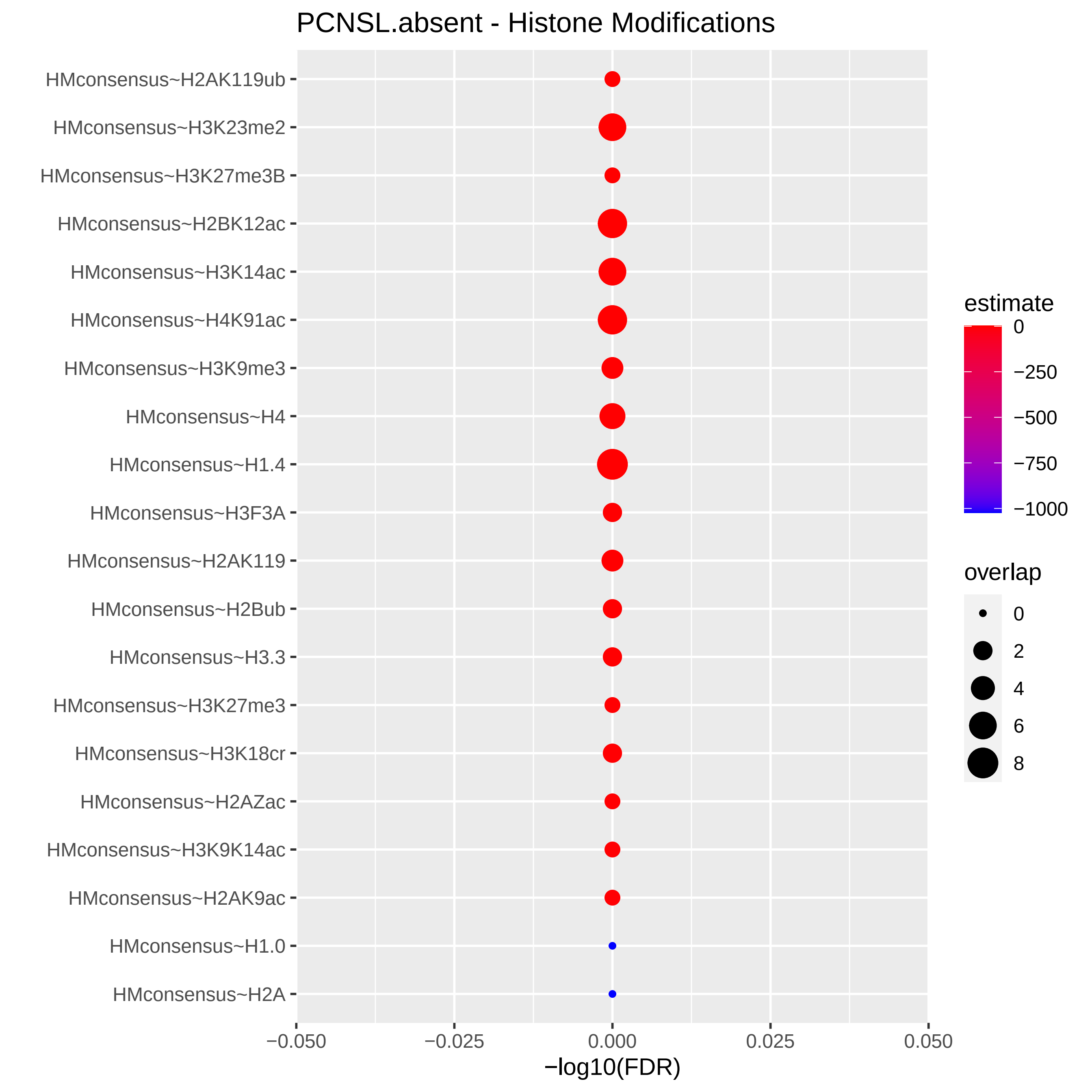

PCNSL.absent - Histone Modifications
HMconsensus~H2AK119ub
HMconsensus~H3K23me2
HMconsensus~H3K27me3B
HMconsensus~H2BK12ac
HMconsensus~H3K14ac
HMconsensus~H4K91ac
HMconsensus~H3K9me3
HMconsensus~H4
HMconsensus~H1.4
HMconsensus~H3F3A
HMconsensus~H2AK119
HMconsensus~H2Bub
HMconsensus~H3.3
HMconsensus~H3K27me3
HMconsensus~H3K18cr
HMconsensus~H2AZac
HMconsensus~H3K9K14ac
HMconsensus~H2AK9ac
HMconsensus~H1.0
HMconsensus~H2A
−0.050
−0.025
0.000
0.025
0.050
−log10(FDR)
estimate
0
−250
−500
−750
−1000
overlap
0
2
4
6
8

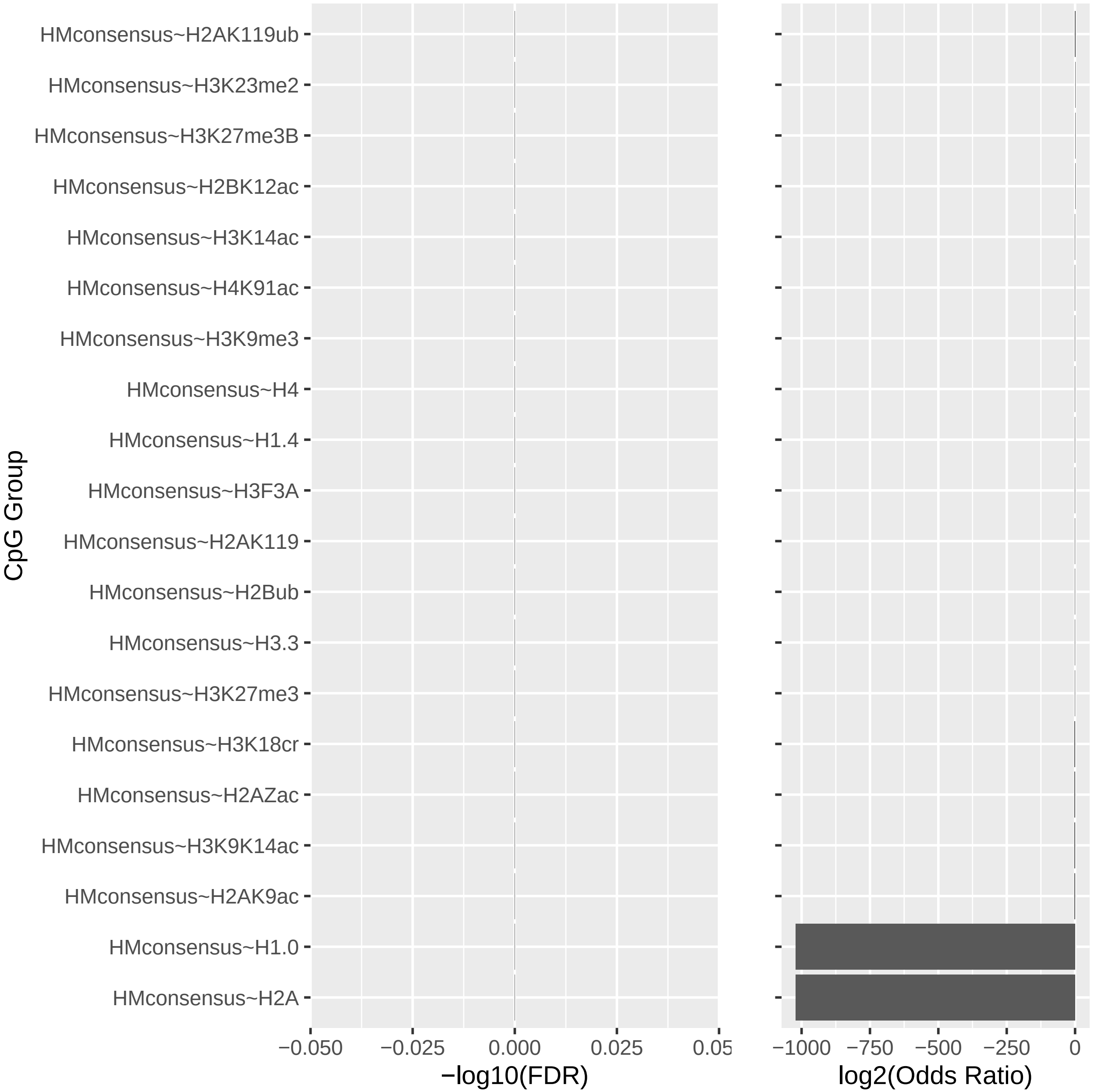
HMconsensus~H2AK119ub
HMconsensus~H3K23me2
HMconsensus~H3K27me3B
HMconsensus~H2BK12ac
HMconsensus~H3K14ac
HMconsensus~H4K91ac
HMconsensus~H3K9me3
HMconsensus~H4
HMconsensus~H1.4
HMconsensus~H3F3A
HMconsensus~H2AK119
HMconsensus~H2Bub
HMconsensus~H3.3
HMconsensus~H3K27me3
HMconsensus~H3K18cr
HMconsensus~H2AZac
HMconsensus~H3K9K14ac
HMconsensus~H2AK9ac
HMconsensus~H1.0
HMconsensus~H2A
CpG Group
−0.050
−0.025
0.000
0.025
0.05
−log10(FDR)
−1000
−750
−500
−250
0
log2(Odds Ratio)

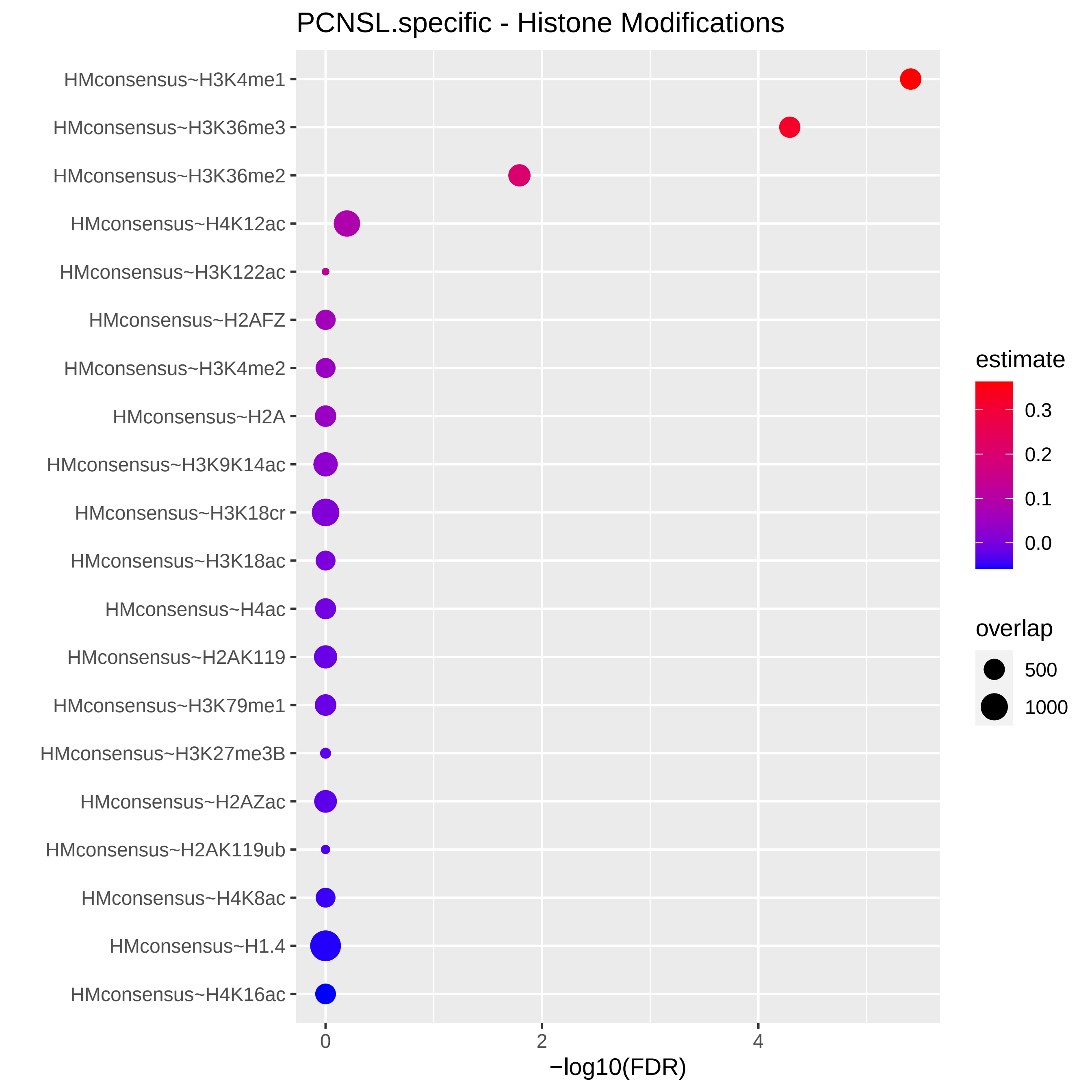

PCNSL.specific - Histone Modifications
HMconsensus~H3K4me1
HMconsensus~H3K36me3
HMconsensus~H3K36me2
HMconsensus~H4K12ac
HMconsensus~H3K122ac
HMconsensus~H2AFZ
HMconsensus~H3K4me2
HMconsensus~H2A
HMconsensus~H3K9K14ac
HMconsensus~H3K18cr
HMconsensus~H3K18ac
HMconsensus~H4ac
HMconsensus~H2AK119
HMconsensus~H3K79me1
HMconsensus~H3K27me3B
HMconsensus~H2AZac
HMconsensus~H2AK119ub
HMconsensus~H4K8ac
HMconsensus~H1.4
HMconsensus~H4K16ac
0
2
4
−log10(FDR)
estimate
0.3
0.2
0.1
0.0
overlap
500
1000

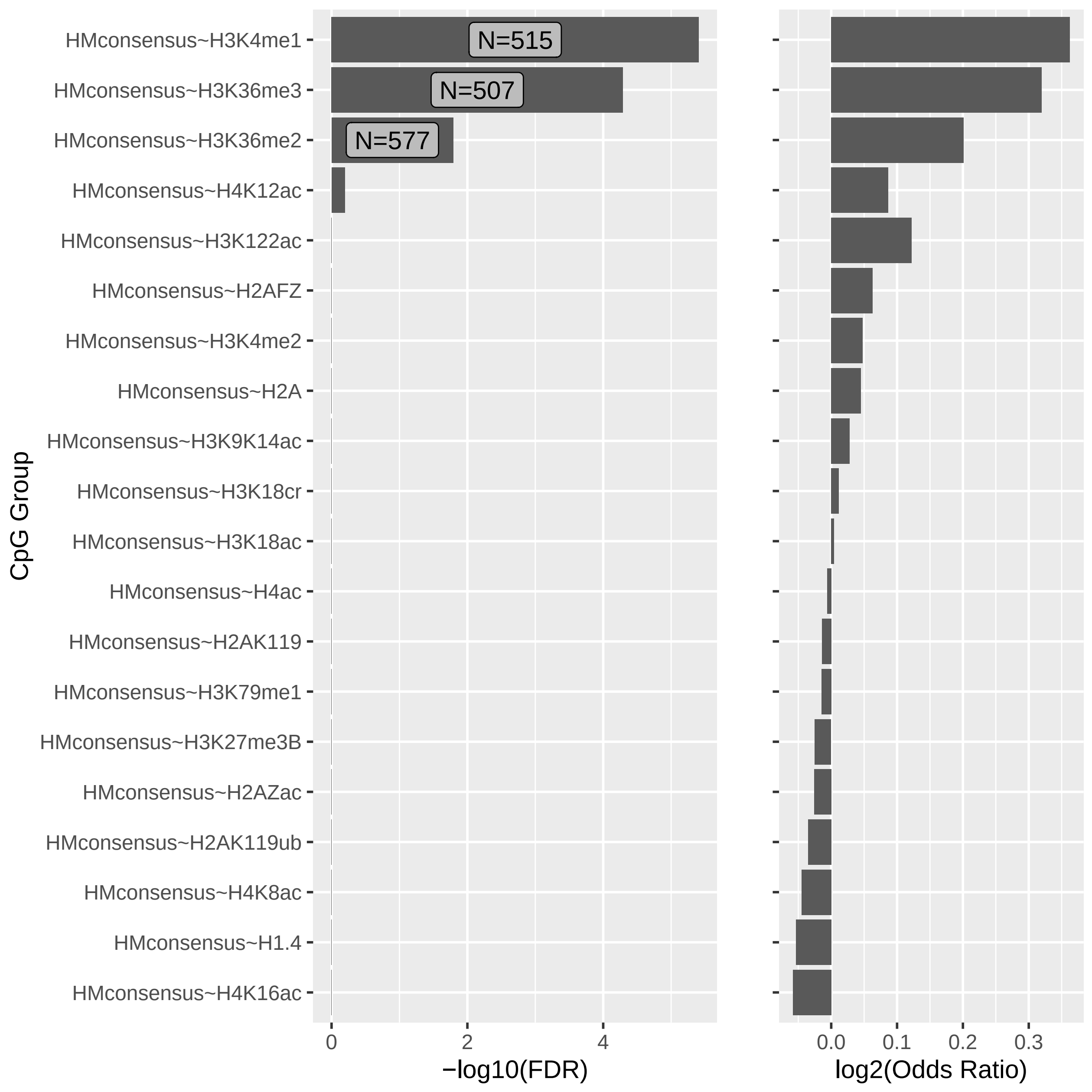
CpG Group
HMconsensus~H3K4me1
HMconsensus~H3K36me3
HMconsensus~H3K36me2
HMconsensus~H4K12ac
HMconsensus~H3K122ac
HMconsensus~H2AFZ
HMconsensus~H3K4me2
HMconsensus~H2A
HMconsensus~H3K9K14ac
HMconsensus~H3K18cr
HMconsensus~H3K18ac
HMconsensus~H4ac
HMconsensus~H2AK119
HMconsensus~H3K79me1
HMconsensus~H3K27me3B
HMconsensus~H2AZac
HMconsensus~H2AK119ub
HMconsensus~H4K8ac
HMconsensus~H1.4
HMconsensus~H4K16ac
N=515
N=507
N=577
0
2
4
−log10(FDR)
0.0
0.1
0.2
0.3
log2(Odds Ratio)

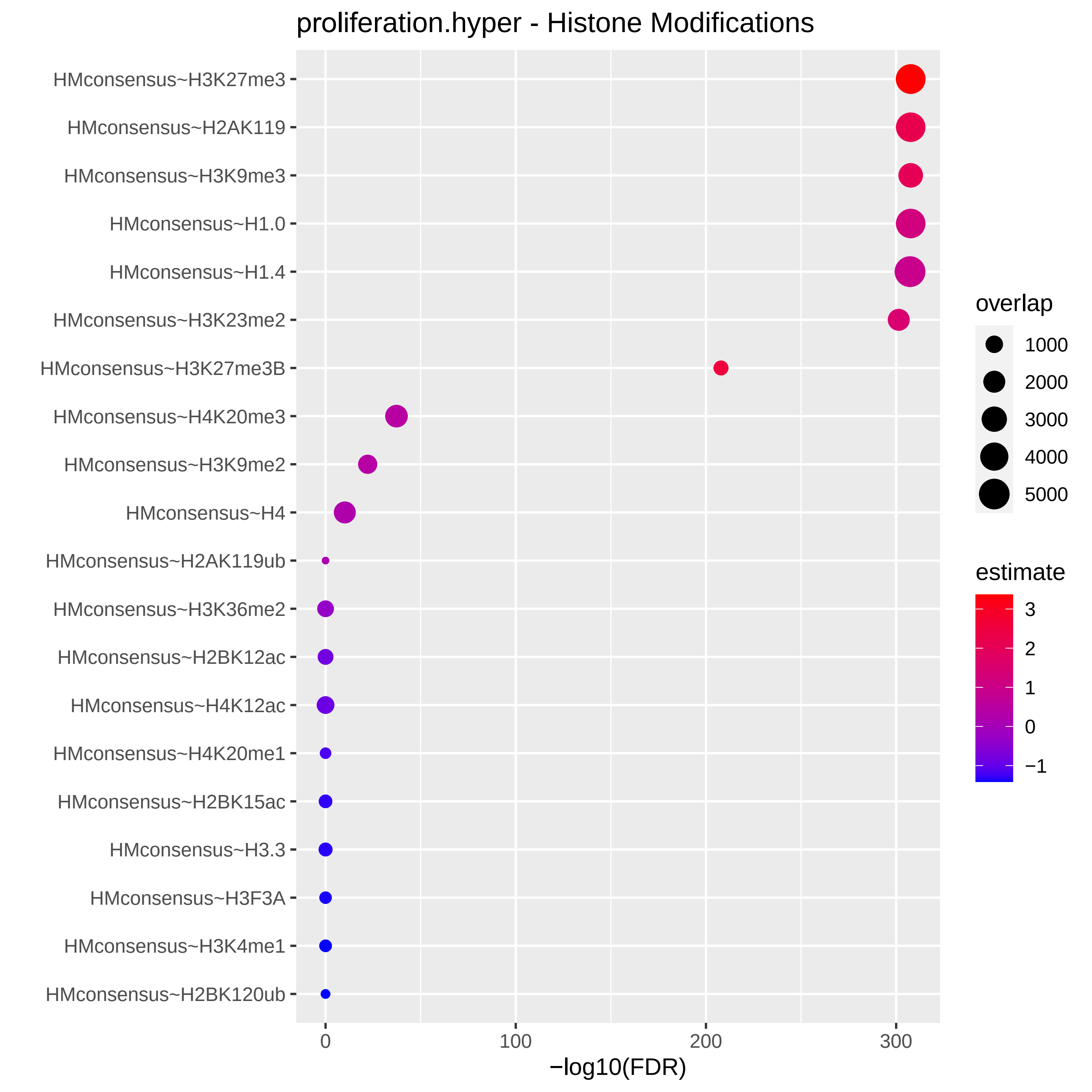

proliferation.hyper - Histone Modifications
HMconsensus~H3K27me3
HMconsensus~H2AK119
HMconsensus~H3K9me3
HMconsensus~H1.0
HMconsensus~H1.4
HMconsensus~H3K23me2
HMconsensus~H3K27me3B
HMconsensus~H4K20me3
HMconsensus~H3K9me2
HMconsensus~H4
HMconsensus~H2AK119ub
HMconsensus~H3K36me2
HMconsensus~H2BK12ac
HMconsensus~H4K12ac
HMconsensus~H4K20me1
HMconsensus~H2BK15ac
HMconsensus~H3.3
HMconsensus~H3F3A
HMconsensus~H3K4me1
HMconsensus~H2BK120ub
0
100
200
300
−log10(FDR)
overlap
1000
2000
3000
4000
5000
estimate
3
2
1
0
−1

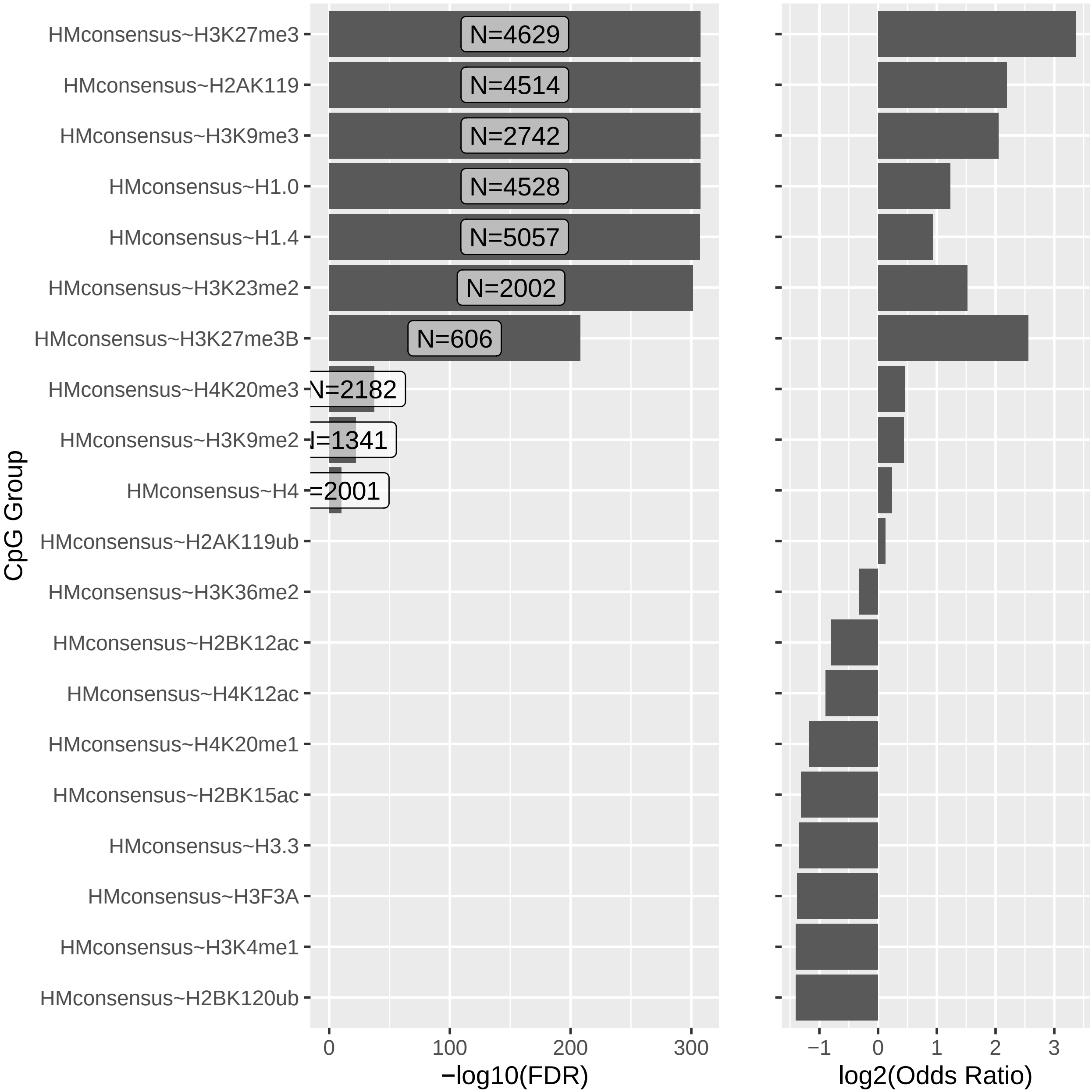

CpG Group
HMconsensus~H3K27me3
HMconsensus~H2AK119
HMconsensus~H3K9me3
HMconsensus~H1.0
HMconsensus~H1.4
HMconsensus~H3K23me2
HMconsensus~H3K27me3B
HMconsensus~H4K20me3
HMconsensus~H3K9me2
HMconsensus~H4
HMconsensus~H2AK119ub
HMconsensus~H3K36me2
HMconsensus~H2BK12ac
HMconsensus~H4K12ac
HMconsensus~H4K20me1
HMconsensus~H2BK15ac
HMconsensus~H3.3
HMconsensus~H3F3A
HMconsensus~H3K4me1
HMconsensus~H2BK120ub
N=4629
N=4514
N=2742
N=4528
N=5057
N=2002
N=606
N=2182
=1341
=2001
0
100
200
300
−log10(FDR)
−1
0
1
2
3
log2(Odds Ratio)

Supplementary figure 4. Detailed results of SeSAMe analysis for histone modifications for the different DMR groups, as indicated. For each DMR group the first graph shows the significance of the association with the specific histone modification adjusted for false discovery rate (−log10(FDR)). The second also includes details of the number of overlapping DMRs overlapping with the specific histone modification.

Supplementary Figure 5

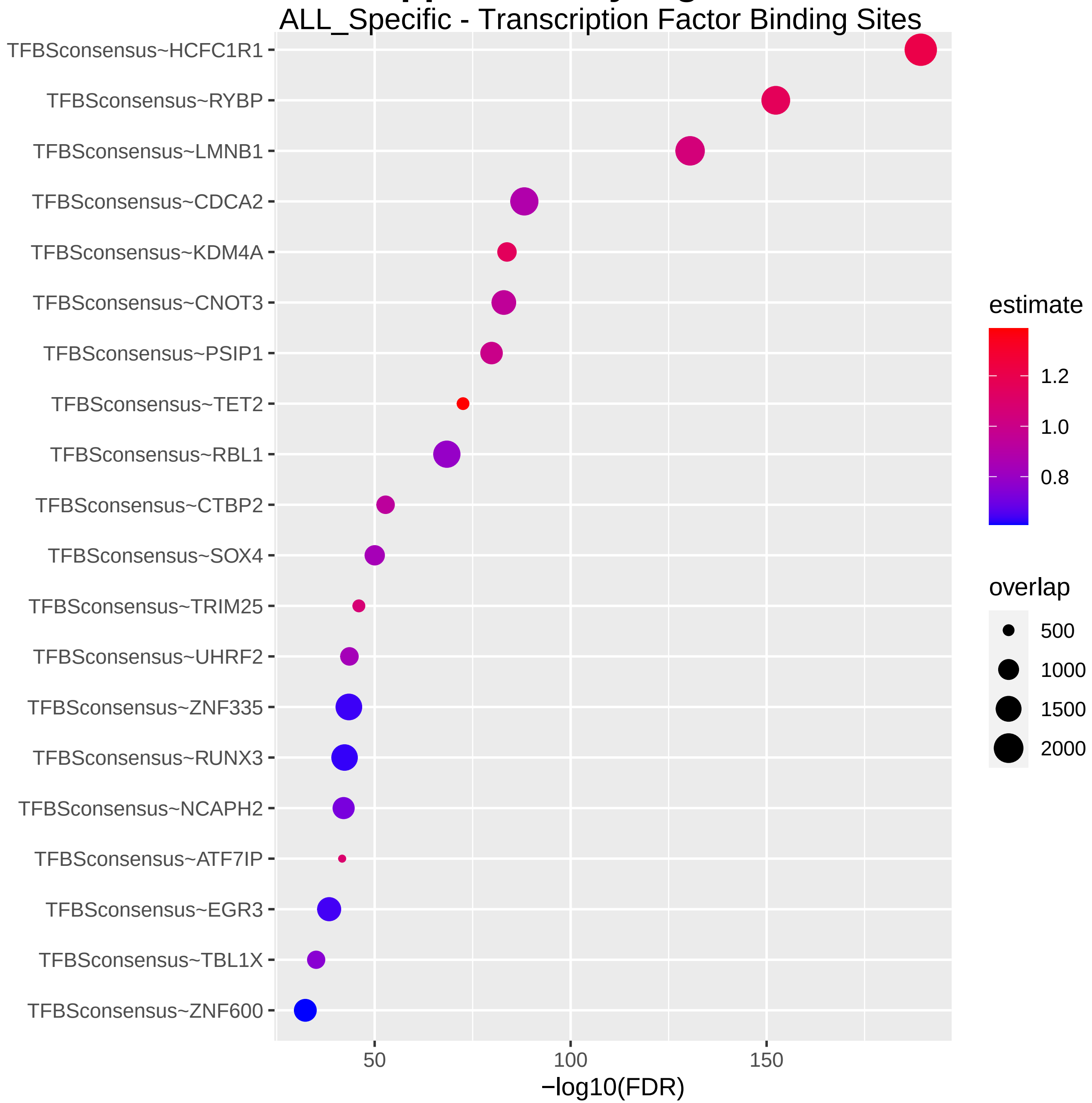

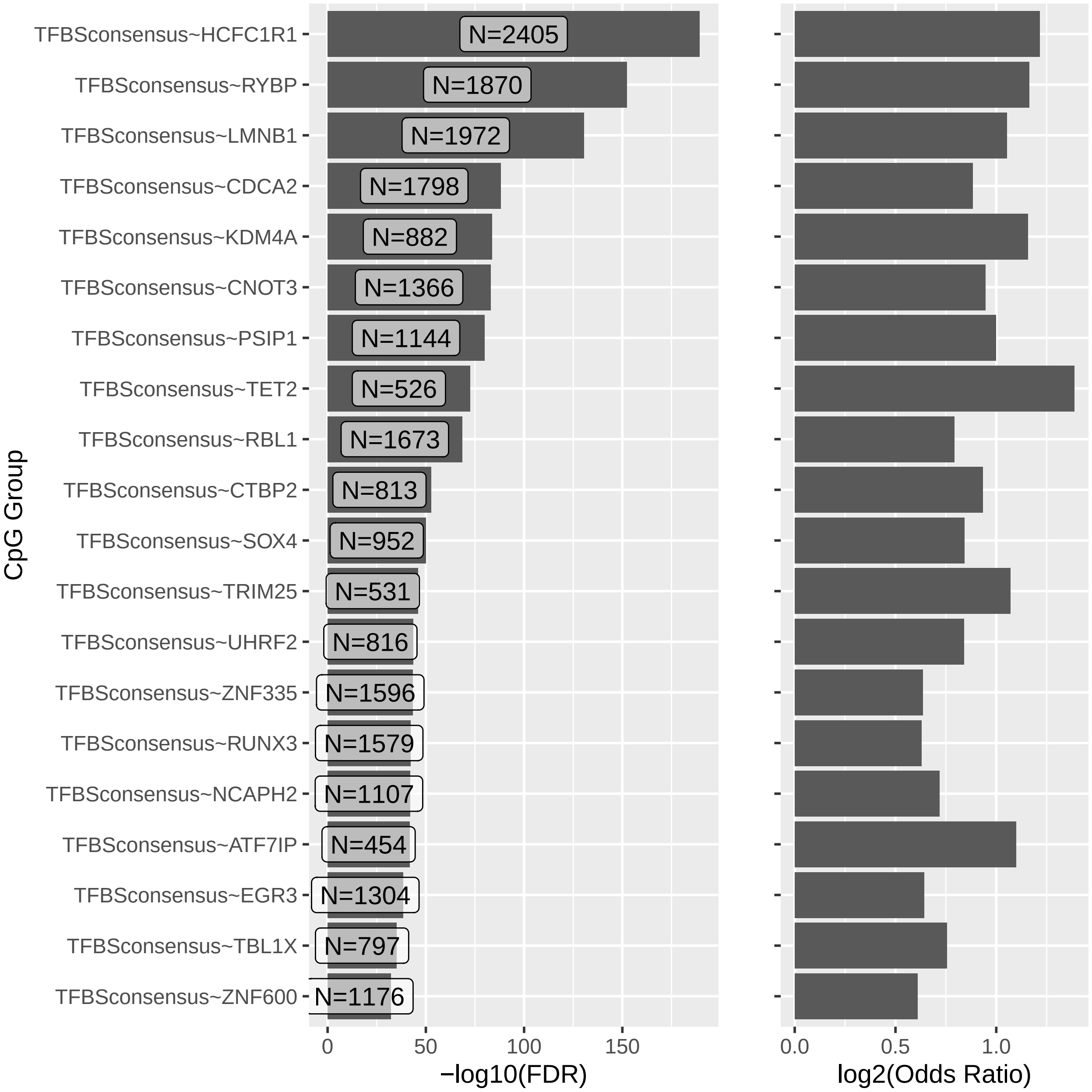

CpG Group
TFBSconsensus~HCFC1R1
TFBSconsensus~RYBP
TFBSconsensus~LMNB1
TFBSconsensus~CDCA2
TFBSconsensus~KDM4A
TFBSconsensus~CNOT3
TFBSconsensus~PSIP1
TFBSconsensus~TET2
TFBSconsensus~RBL1
TFBSconsensus~CTBP2
TFBSconsensus~SOX4
TFBSconsensus~TRIM25
TFBSconsensus~UHRF2
TFBSconsensus~ZNF335
TFBSconsensus~RUNX3
TFBSconsensus~NCAPH2
TFBSconsensus~ATF7IP
TFBSconsensus~EGR3
TFBSconsensus~TBL1X
TFBSconsensus~ZNF600
N=2405
N=1870
N=1972
N=1798
N=882
N=1366
N=1144
N=526
N=1673
N=813
N=952
N=531
N=816
N=1596
N=1579
N=1107
N=454
N=1304
N=797
N=1176
0
50
100
150
−log10(FDR)
0.0
0.5
1.0
log2(Odds Ratio)

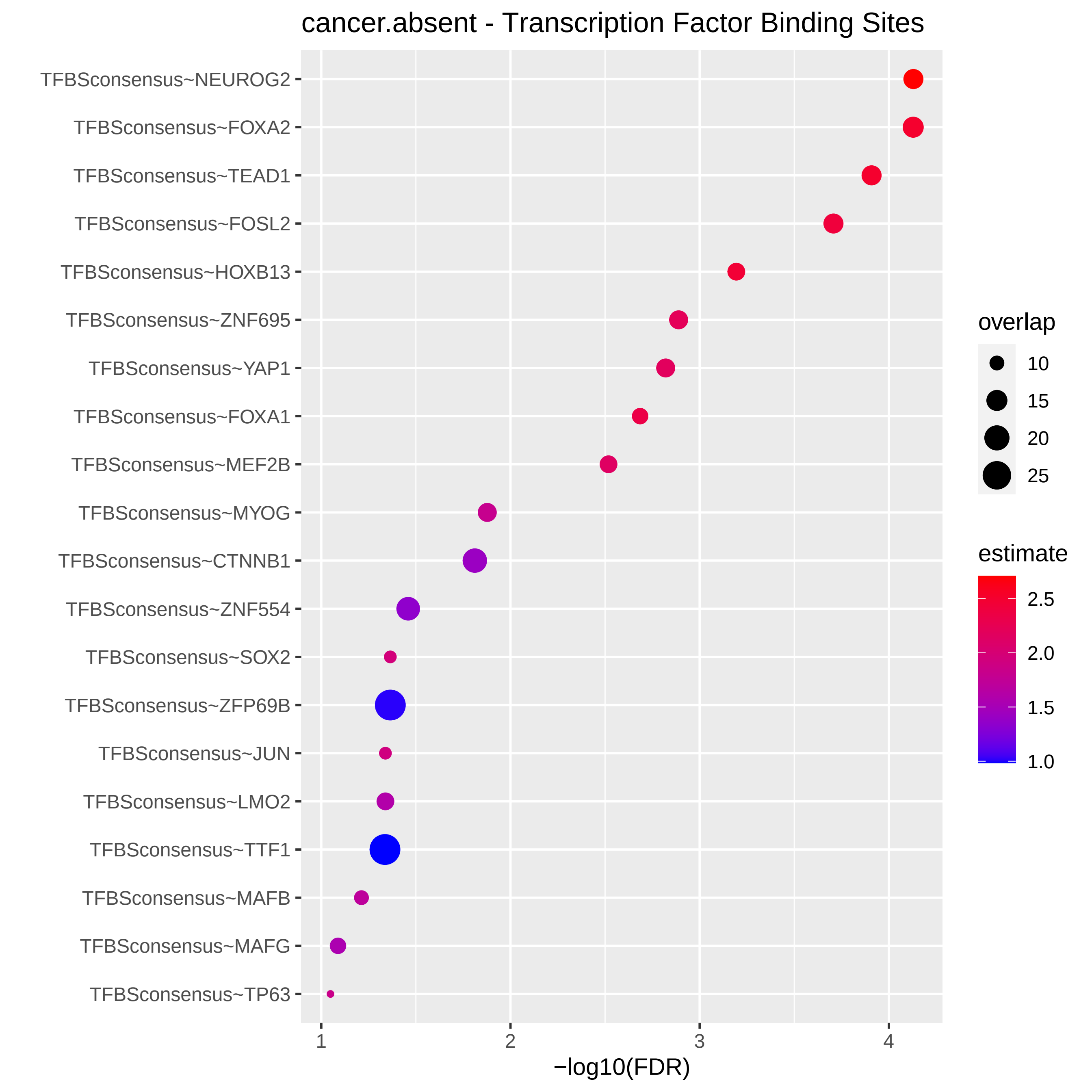

cancer.absent - Transcription Factor Binding Sites
TFBSconsensus~NEUROG2
TFBSconsensus~FOXA2
TFBSconsensus~TEAD1
TFBSconsensus~FOSL2
TFBSconsensus~HOXB13
TFBSconsensus~ZNF695
TFBSconsensus~YAP1
TFBSconsensus~FOXA1
TFBSconsensus~MEF2B
TFBSconsensus~MYOG
TFBSconsensus~CTNNB1
TFBSconsensus~ZNF554
TFBSconsensus~SOX2
TFBSconsensus~ZFP69B
TFBSconsensus~JUN
TFBSconsensus~LMO2
TFBSconsensus~TTF1
TFBSconsensus~MAFB
TFBSconsensus~MAFG
TFBSconsensus~TP63
1
2
3
4
−log10(FDR)
overlap
10
15
20
25
estimate
2.5
2.0
1.5
1.0

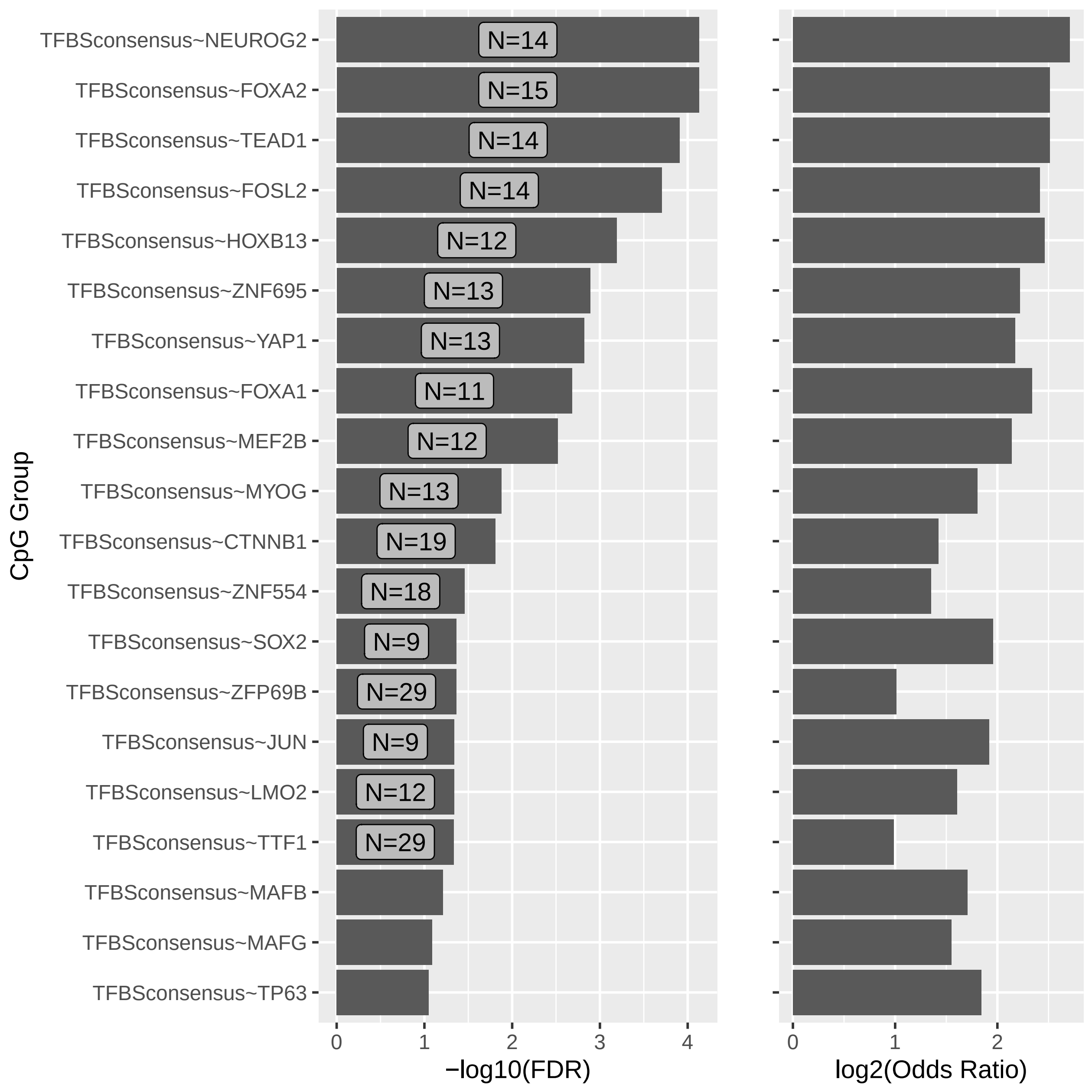

CpG Group
TFBSconsensus~NEUROG2
TFBSconsensus~FOXA2
TFBSconsensus~TEAD1
TFBSconsensus~FOSL2
TFBSconsensus~HOXB13
TFBSconsensus~ZNF695
TFBSconsensus~YAP1
TFBSconsensus~FOXA1
TFBSconsensus~MEF2B
TFBSconsensus~MYOG
TFBSconsensus~CTNNB1
TFBSconsensus~ZNF554
TFBSconsensus~SOX2
TFBSconsensus~ZFP69B
TFBSconsensus~JUN
TFBSconsensus~LMO2
TFBSconsensus~TTF1
TFBSconsensus~MAFB
TFBSconsensus~MAFG
TFBSconsensus~TP63
N=14
N=15
N=14
N=14
N=12
N=13
N=13
N=11
N=12
N=13
N=19
N=18
N=9
N=29
N=9
N=12
N=29
0
1
2
3
4
−log10(FDR)
0
1
2
log2(Odds Ratio)

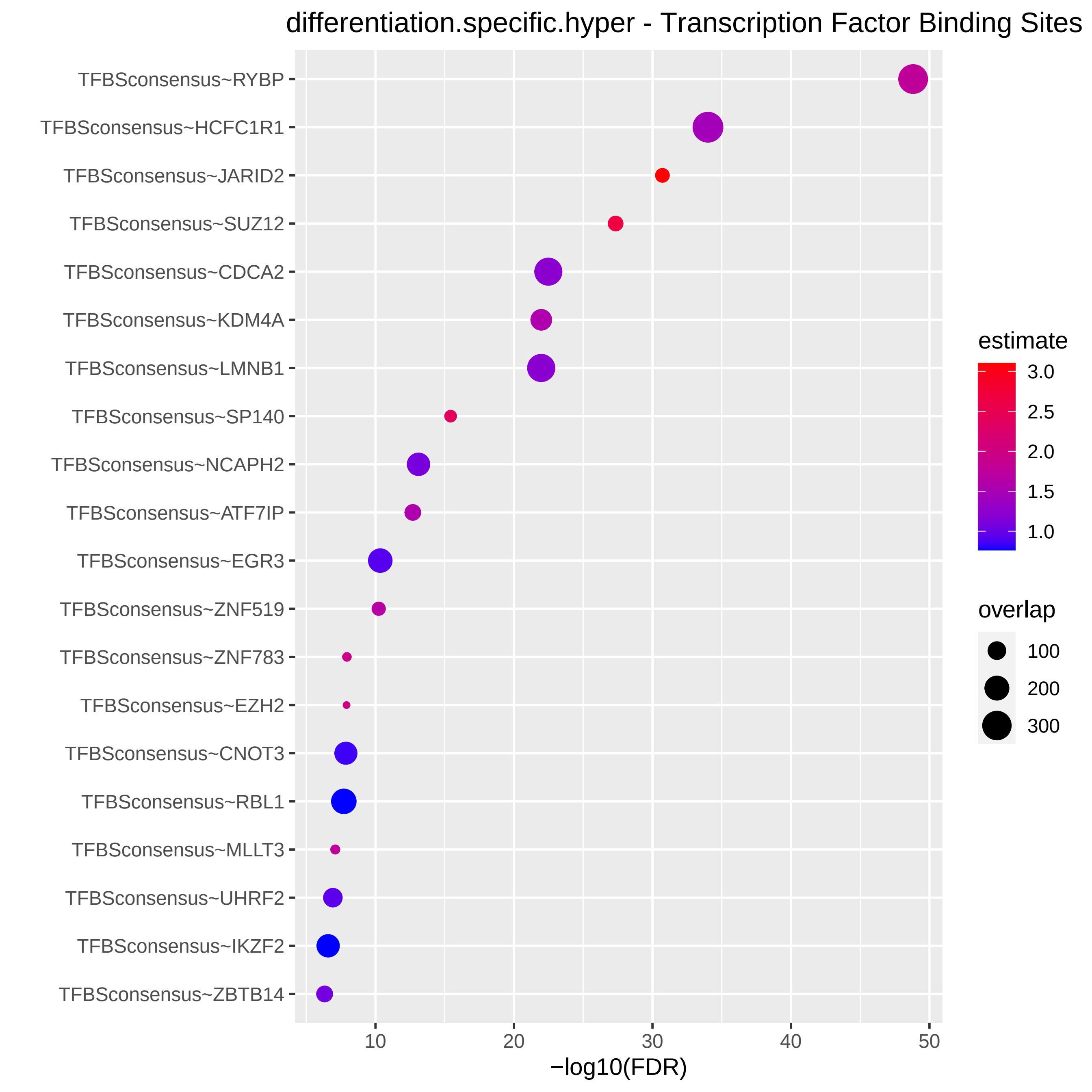

differentiation.specific.hyper - Transcription Factor Binding Sites
TFBSconsensus~RYBP
TFBSconsensus~HCFC1R1
TFBSconsensus~JARID2
TFBSconsensus~SUZ12
TFBSconsensus~CDCA2
TFBSconsensus~KDM4A
TFBSconsensus~LMNB1
TFBSconsensus~SP140
TFBSconsensus~NCAPH2
TFBSconsensus~ATF7IP
TFBSconsensus~EGR3
TFBSconsensus~ZNF519
TFBSconsensus~ZNF783
TFBSconsensus~EZH2
TFBSconsensus~CNOT3
TFBSconsensus~RBL1
TFBSconsensus~MLLT3
TFBSconsensus~UHRF2
TFBSconsensus~IKZF2
TFBSconsensus~ZBTB14
10
20
30
40
50
−log10(FDR)
estimate
3.0
2.5
2.0
1.5
1.0
overlap
100
200
300

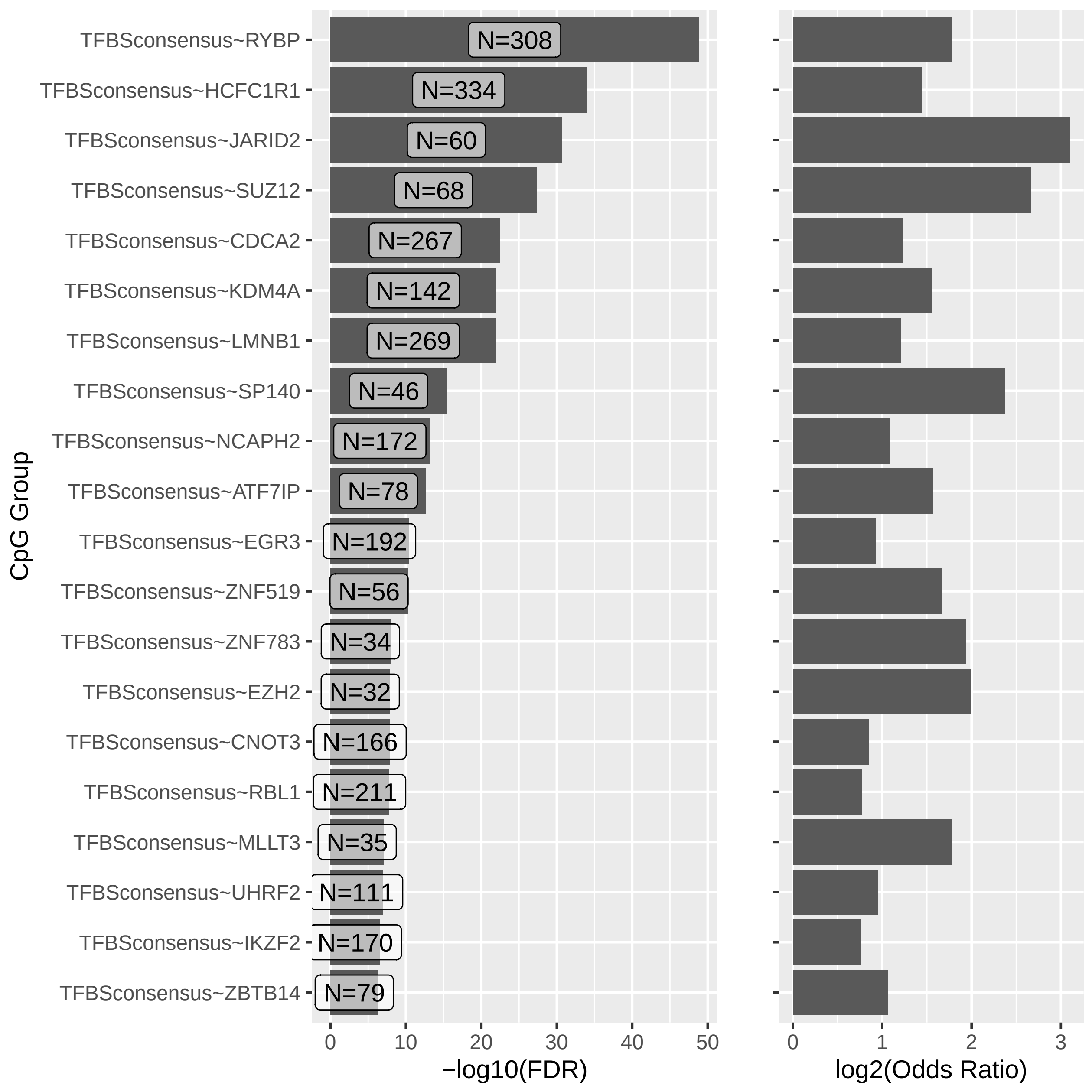

CpG Group
TFBSconsensus~RYBP
TFBSconsensus~HCFC1R1
TFBSconsensus~JARID2
TFBSconsensus~SUZ12
TFBSconsensus~CDCA2
TFBSconsensus~KDM4A
TFBSconsensus~LMNB1
TFBSconsensus~SP140
TFBSconsensus~NCAPH2
TFBSconsensus~ATF7IP
TFBSconsensus~EGR3
TFBSconsensus~ZNF519
TFBSconsensus~ZNF783
TFBSconsensus~EZH2
TFBSconsensus~CNOT3
TFBSconsensus~RBL1
TFBSconsensus~MLLT3
TFBSconsensus~UHRF2
TFBSconsensus~IKZF2
TFBSconsensus~ZBTB14
N=308
N=334
N=60
N=68
N=267
N=142
N=269
N=46
N=172
N=78
N=192
N=56
N=34
N=32
N=166
N=211
N=35
N=111
N=170
N=79
0
10
20
30
40
50
−log10(FDR)
0
1
2
3
log2(Odds Ratio)

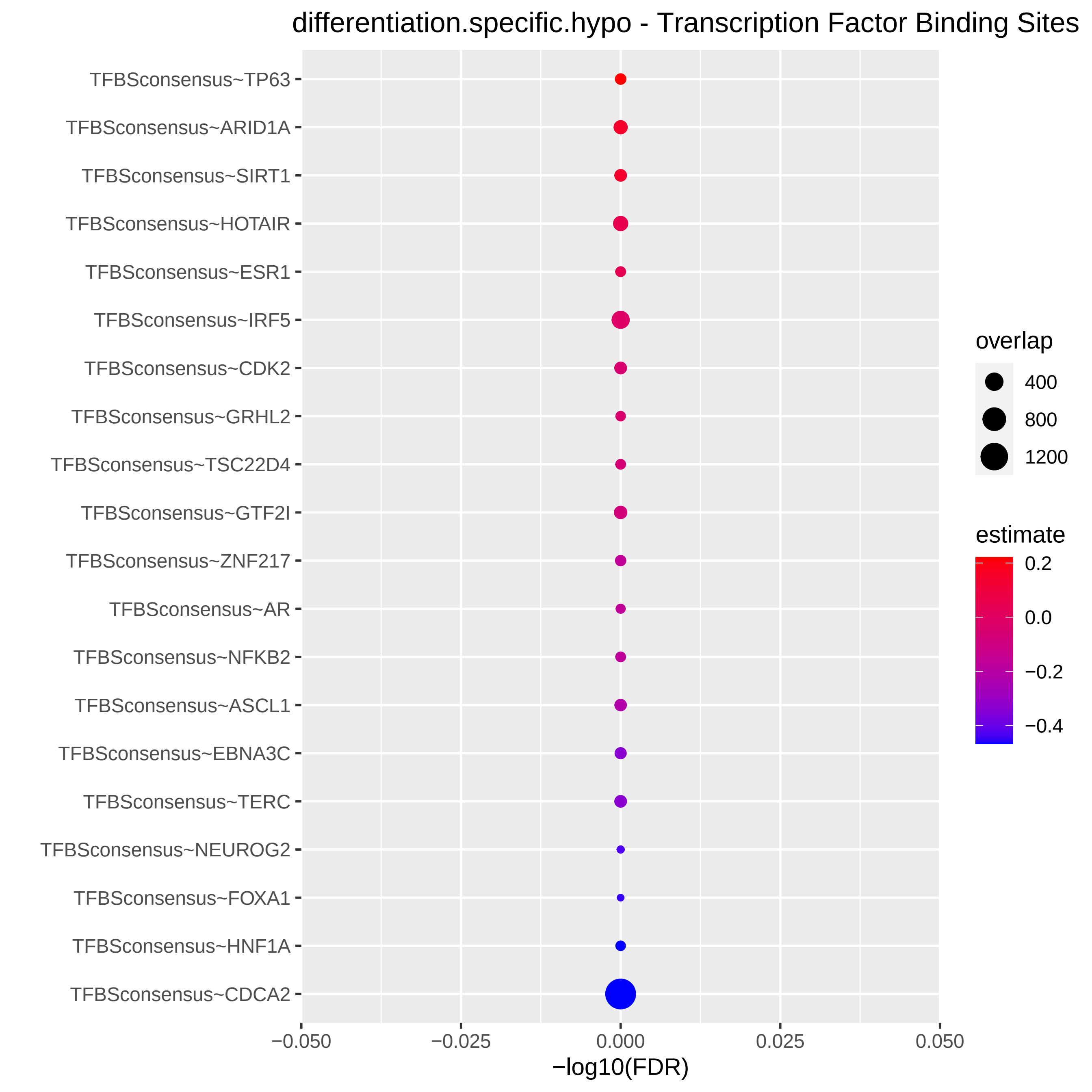

differentiation.specific.hypo - Transcription Factor Binding Sites
TFBSconsensus~TP63
TFBSconsensus~ARID1A
TFBSconsensus~SIRT1
TFBSconsensus~HOTAIR
TFBSconsensus~ESR1
TFBSconsensus~IRF5
TFBSconsensus~CDK2
TFBSconsensus~GRHL2
TFBSconsensus~TSC22D4
TFBSconsensus~GTF2I
TFBSconsensus~ZNF217
TFBSconsensus~AR
TFBSconsensus~NFKB2
TFBSconsensus~ASCL1
TFBSconsensus~EBNA3C
TFBSconsensus~TERC
TFBSconsensus~NEUROG2
TFBSconsensus~FOXA1
TFBSconsensus~HNF1A
TFBSconsensus~CDCA2
−0.050
−0.025
0.000
0.025
0.050
−log10(FDR)
overlap
400
800
1200
estimate
0.2
0.0
−0.2
−0.4

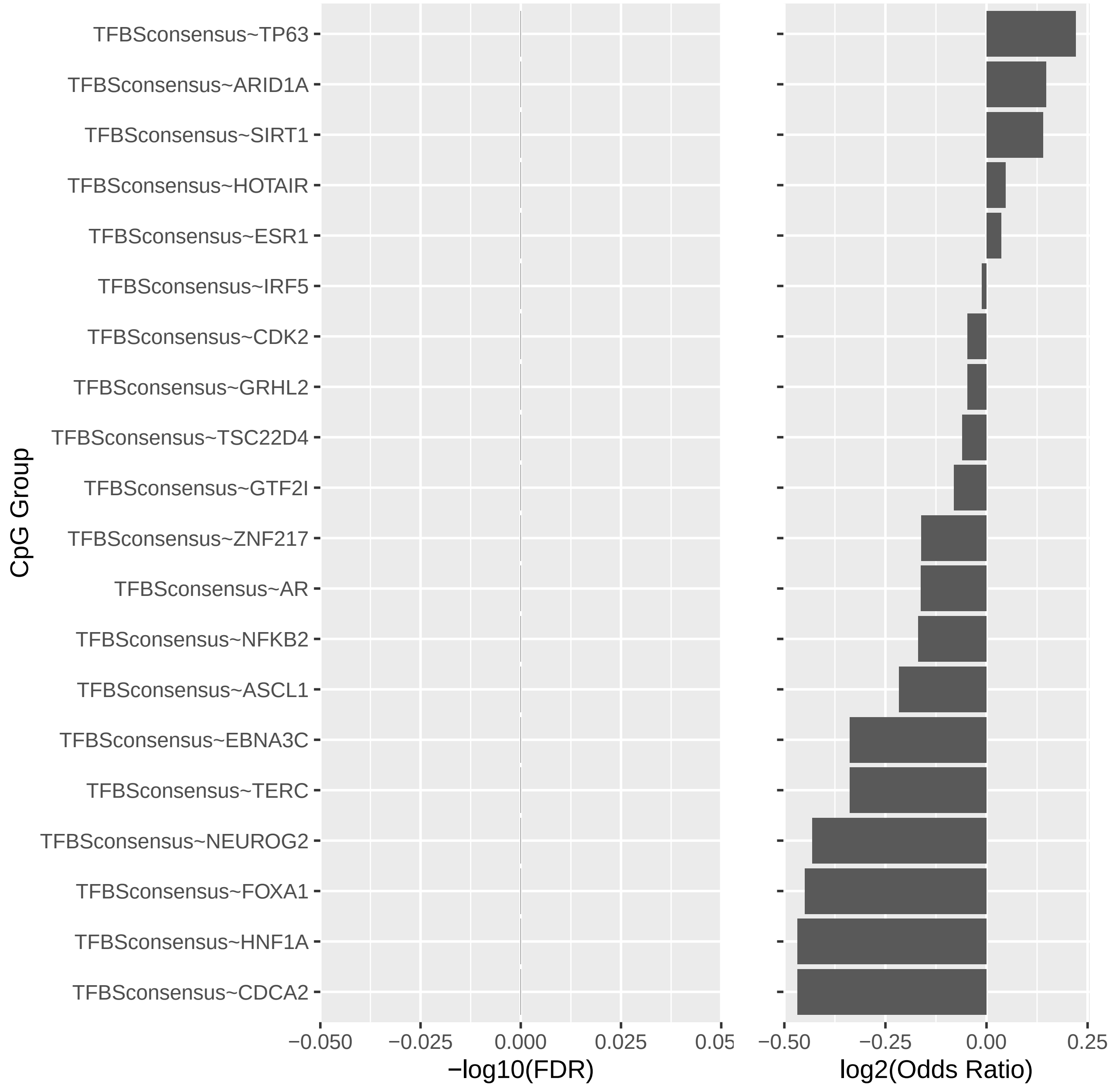

TFBSconsensus~TP63
TFBSconsensus~ARID1A
TFBSconsensus~SIRT1
TFBSconsensus~HOTAIR
TFBSconsensus~ESR1
TFBSconsensus~IRF5
TFBSconsensus~CDK2
TFBSconsensus~GRHL2
TFBSconsensus~TSC22D4
TFBSconsensus~GTF2I
TFBSconsensus~ZNF217
TFBSconsensus~AR
TFBSconsensus~NFKB2
TFBSconsensus~ASCL1
TFBSconsensus~EBNA3C
TFBSconsensus~TERC
TFBSconsensus~NEUROG2
TFBSconsensus~FOXA1
TFBSconsensus~HNF1A
TFBSconsensus~CDCA2
CpG Group
−0.050 −0.025 0.000 0.025 0.05
−log10(FDR)
−0.50 −0.25 0.00 0.25
log2(Odds Ratio)

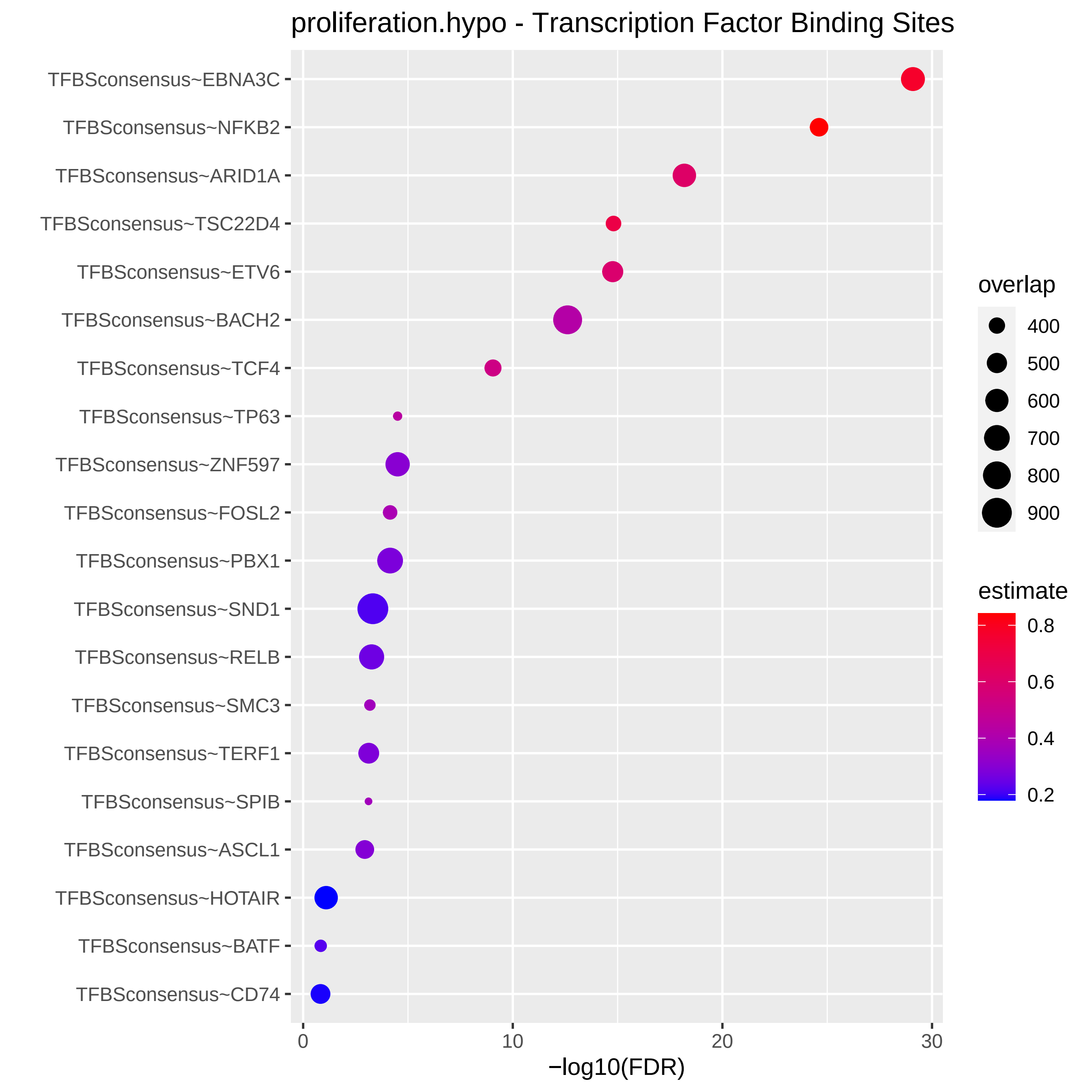

proliferation.hypo - Transcription Factor Binding Sites
TFBSconsensus~EBNA3C
TFBSconsensus~NFKB2
TFBSconsensus~ARID1A
TFBSconsensus~TSC22D4
TFBSconsensus~ETV6
TFBSconsensus~BACH2
TFBSconsensus~TCF4
TFBSconsensus~TP63
TFBSconsensus~ZNF597
TFBSconsensus~FOSL2
TFBSconsensus~PBX1
TFBSconsensus~SND1
TFBSconsensus~RELB
TFBSconsensus~SMC3
TFBSconsensus~TERF1
TFBSconsensus~SPIB
TFBSconsensus~ASCL1
TFBSconsensus~HOTAIR
TFBSconsensus~BATF
TFBSconsensus~CD74
0
10
20
30
−log10(FDR)
overlap
400
500
600
700
800
900
estimate
0.8
0.6
0.4
0.2

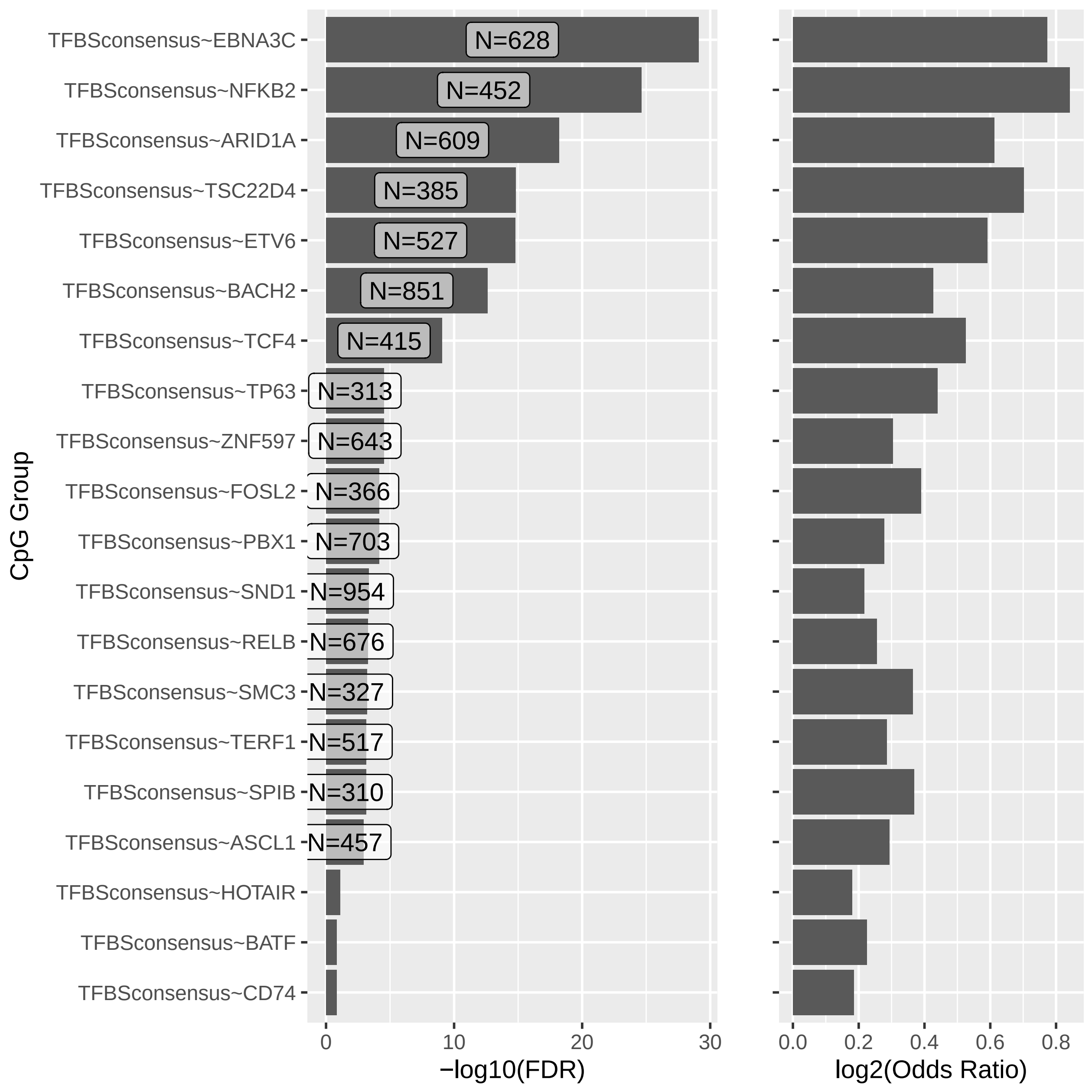
CpG Group
TFBSconsensus~EBNA3C
TFBSconsensus~NFKB2
TFBSconsensus~ARID1A
TFBSconsensus~TSC22D4
TFBSconsensus~ETV6
TFBSconsensus~BACH2
TFBSconsensus~TCF4
TFBSconsensus~TP63
TFBSconsensus~ZNF597
TFBSconsensus~FOSL2
TFBSconsensus~PBX1
TFBSconsensus~SND1
TFBSconsensus~RELB
TFBSconsensus~SMC3
TFBSconsensus~TERF1
TFBSconsensus~SPIB
TFBSconsensus~ASCL1
TFBSconsensus~HOTAIR
TFBSconsensus~BATF
TFBSconsensus~CD74
N=628
N=452
N=609
N=385
N=527
N=851
N=415
N=313
N=643
N=366
N=703
N=954
N=676
N=327
N=517
N=310
N=457
0
10
20
30
−log10(FDR)
0.0
0.2
0.4
0.6
0.8
log2(Odds Ratio)

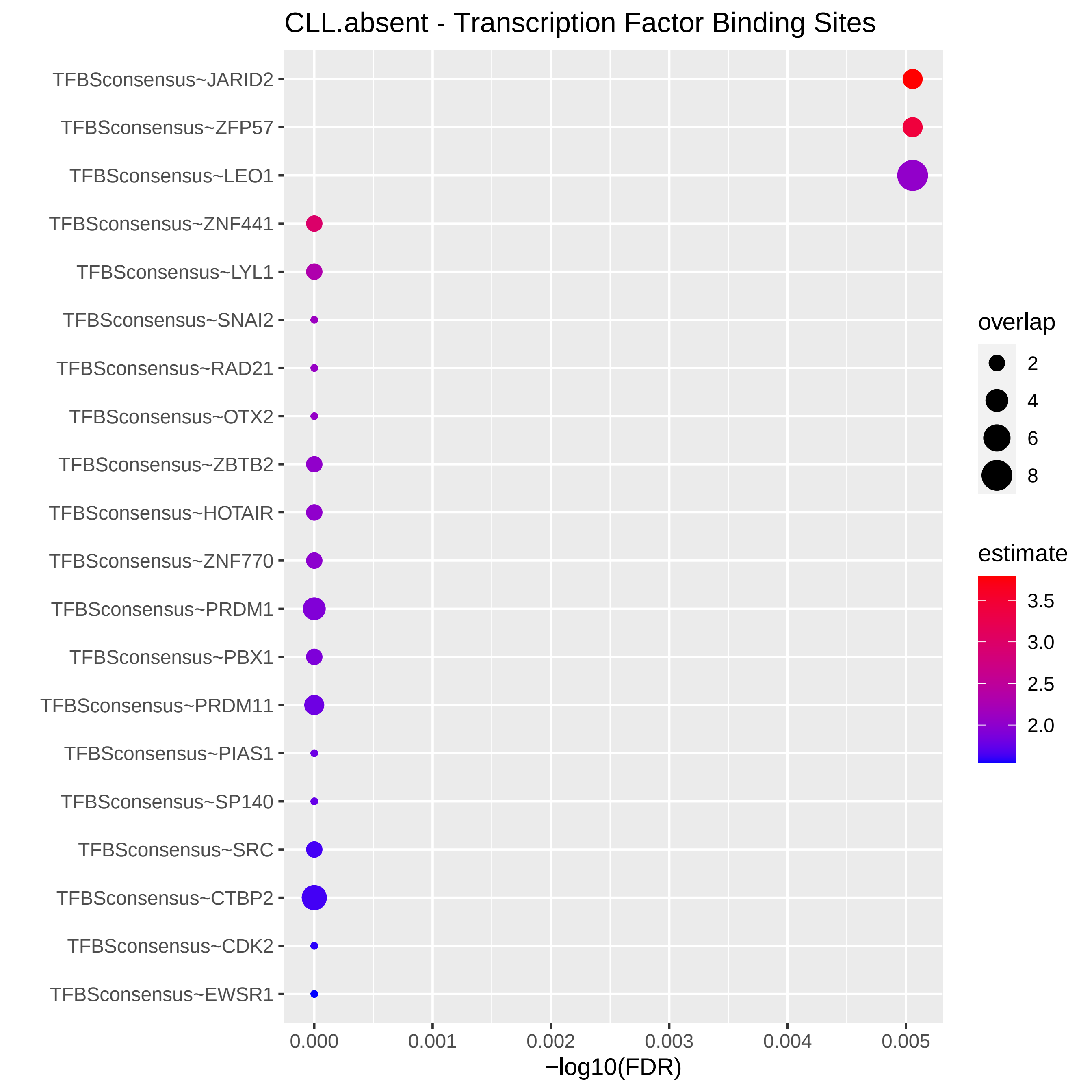

CLL.absent - Transcription Factor Binding Sites
TFBSconsensus~JARID2
TFBSconsensus~ZFP57
TFBSconsensus~LEO1
TFBSconsensus~ZNF441
TFBSconsensus~LYL1
TFBSconsensus~SNAI2
TFBSconsensus~RAD21
TFBSconsensus~OTX2
TFBSconsensus~ZBTB2
TFBSconsensus~HOTAIR
TFBSconsensus~ZNF770
TFBSconsensus~PRDM1
TFBSconsensus~PBX1
TFBSconsensus~PRDM11
TFBSconsensus~PIAS1
TFBSconsensus~SP140
TFBSconsensus~SRC
TFBSconsensus~CTBP2
TFBSconsensus~CDK2
TFBSconsensus~EWSR1
0.000
0.001
0.002
0.003
0.004
0.005
−log10(FDR)
overlap
2
4
6
8
estimate
3.5
3.0
2.5
2.0

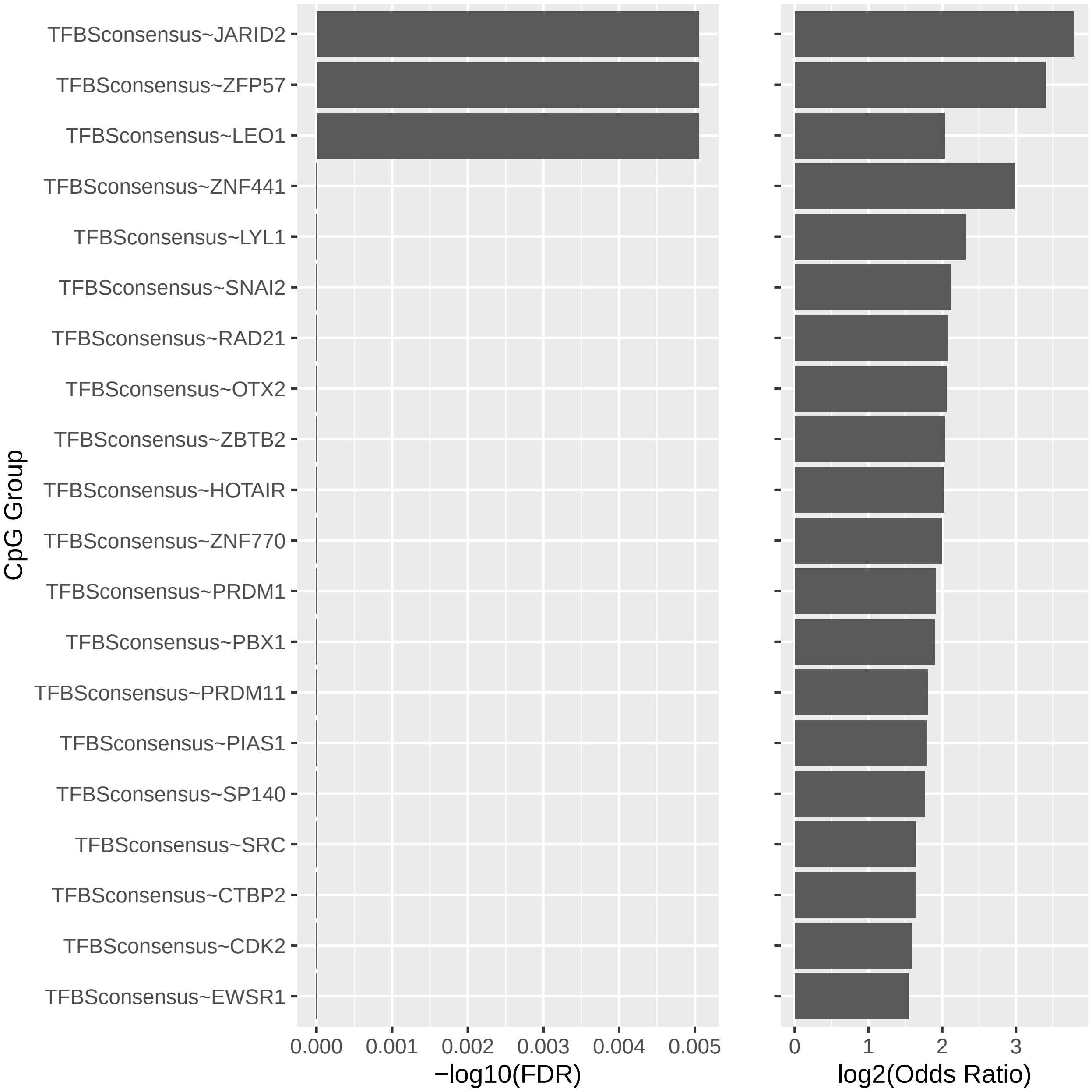

CpG Group
TFBSconsensus~JARID2
TFBSconsensus~ZFP57
TFBSconsensus~LEO1
TFBSconsensus~ZNF441
TFBSconsensus~LYL1
TFBSconsensus~SNAI2
TFBSconsensus~RAD21
TFBSconsensus~OTX2
TFBSconsensus~ZBTB2
TFBSconsensus~HOTAIR
TFBSconsensus~ZNF770
TFBSconsensus~PRDM1
TFBSconsensus~PBX1
TFBSconsensus~PRDM11
TFBSconsensus~PIAS1
TFBSconsensus~SP140
TFBSconsensus~SRC
TFBSconsensus~CTBP2
TFBSconsensus~CDK2
TFBSconsensus~EWSR1
0.000
0.001
0.002
0.003
0.004
0.005
−log10(FDR)
0
1
2
3
log2(Odds Ratio)

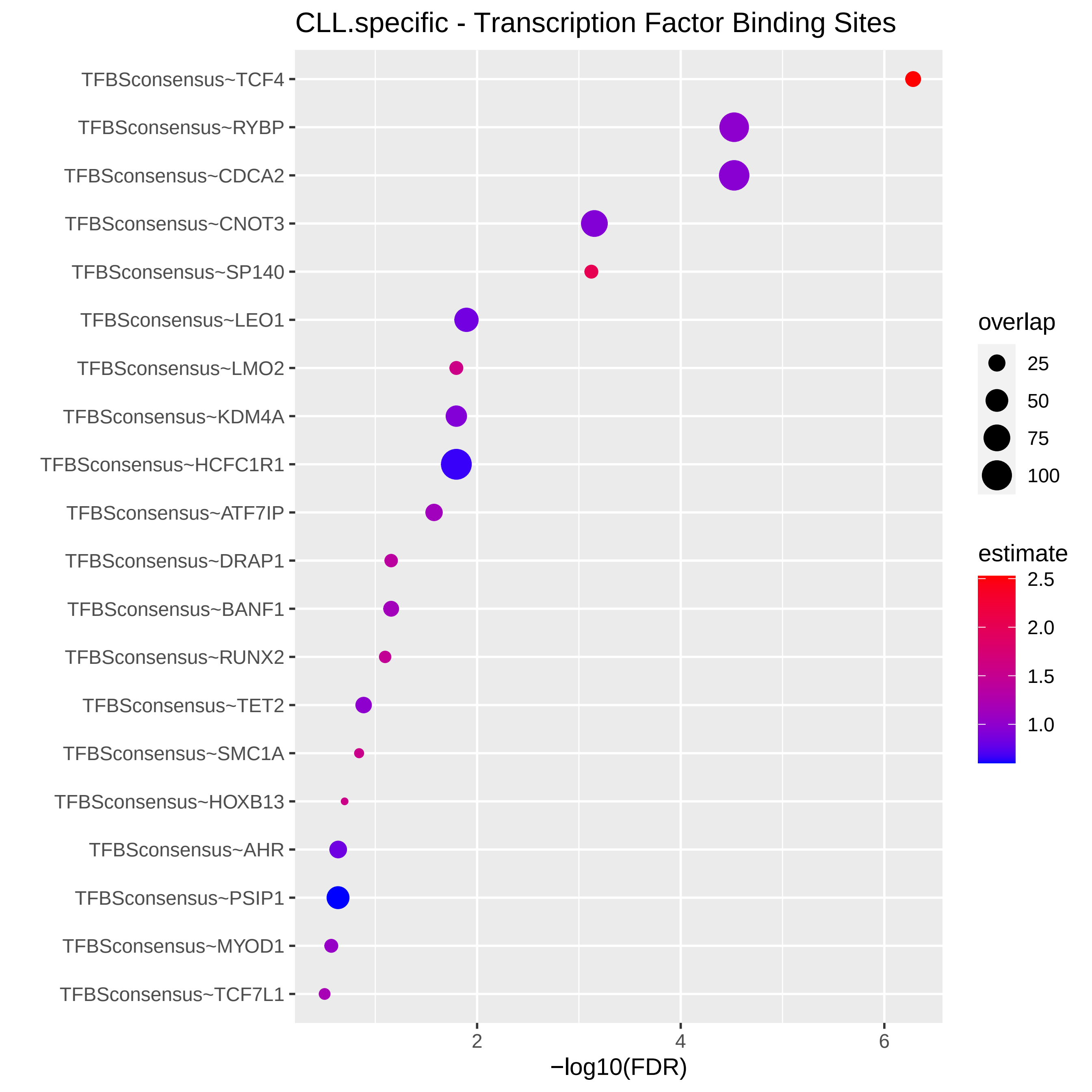
CLL.specific - Transcription Factor Binding Sites
TFBSconsensus~TCF4
TFBSconsensus~RYBP
TFBSconsensus~CDCA2
TFBSconsensus~CNOT3
TFBSconsensus~SP140
TFBSconsensus~LEO1
TFBSconsensus~LMO2
TFBSconsensus~KDM4A
TFBSconsensus~HCFC1R1
TFBSconsensus~ATF7IP
TFBSconsensus~DRAP1
TFBSconsensus~BANF1
TFBSconsensus~RUNX2
TFBSconsensus~TET2
TFBSconsensus~SMC1A
TFBSconsensus~HOXB13
TFBSconsensus~AHR
TFBSconsensus~PSIP1
TFBSconsensus~MYOD1
TFBSconsensus~TCF7L1
2
4
6
−log10(FDR)
overlap
25
50
75
100
estimate
2.5
2.0
1.5
1.0

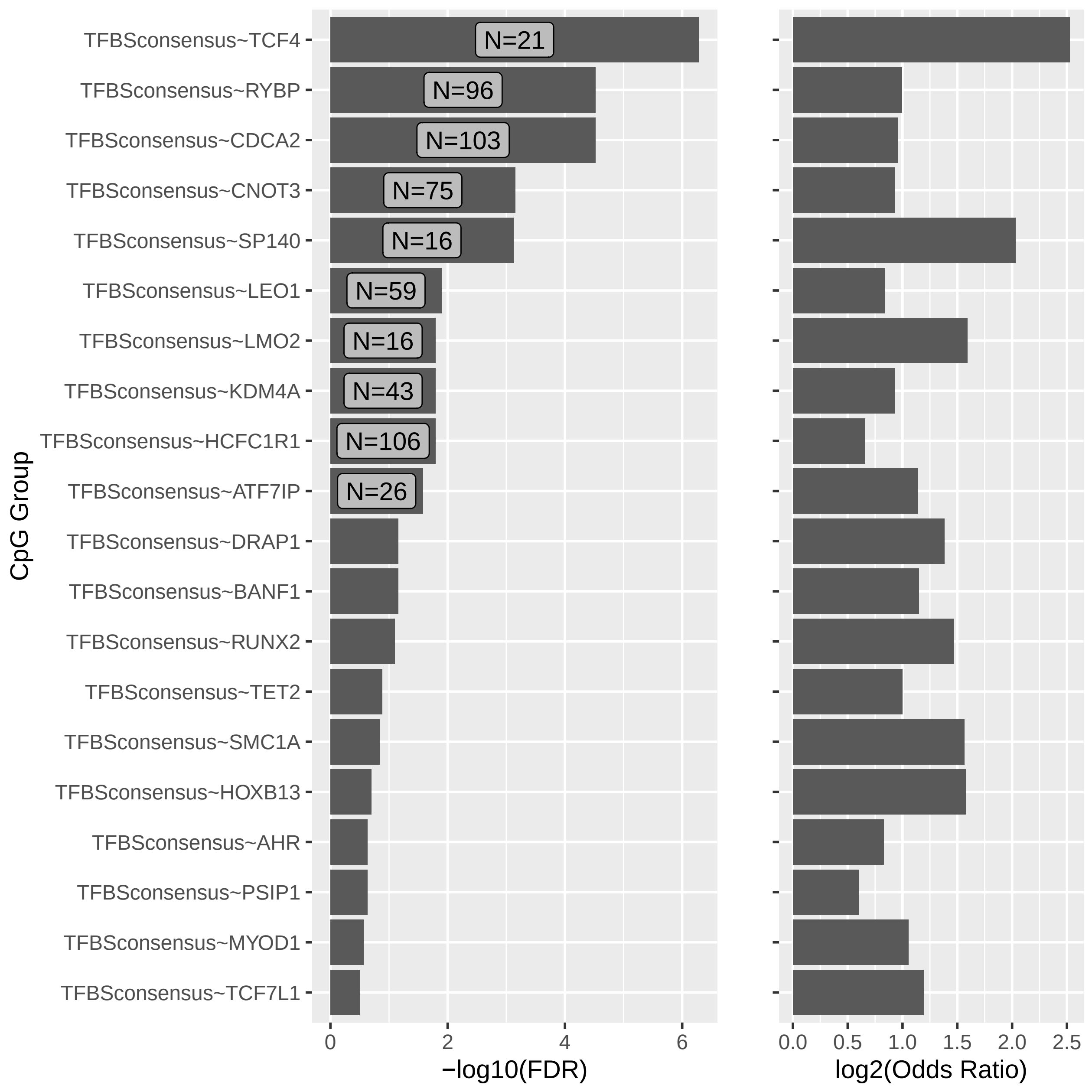
CpG Group
TFBSconsensus~TCF4
TFBSconsensus~RYBP
TFBSconsensus~CDCA2
TFBSconsensus~CNOT3
TFBSconsensus~SP140
TFBSconsensus~LEO1
TFBSconsensus~LMO2
TFBSconsensus~KDM4A
TFBSconsensus~HCFC1R1
TFBSconsensus~ATF7IP
TFBSconsensus~DRAP1
TFBSconsensus~BANF1
TFBSconsensus~RUNX2
TFBSconsensus~TET2
TFBSconsensus~SMC1A
TFBSconsensus~HOXB13
TFBSconsensus~AHR
TFBSconsensus~PSIP1
TFBSconsensus~MYOD1
TFBSconsensus~TCF7L1
N=21
N=96
N=103
N=75
N=16
N=59
N=16
N=43
N=106
N=26
0
2
4
6
−log10(FDR)
0.0
0.5
1.0
1.5
2.0
2.5
log2(Odds Ratio)

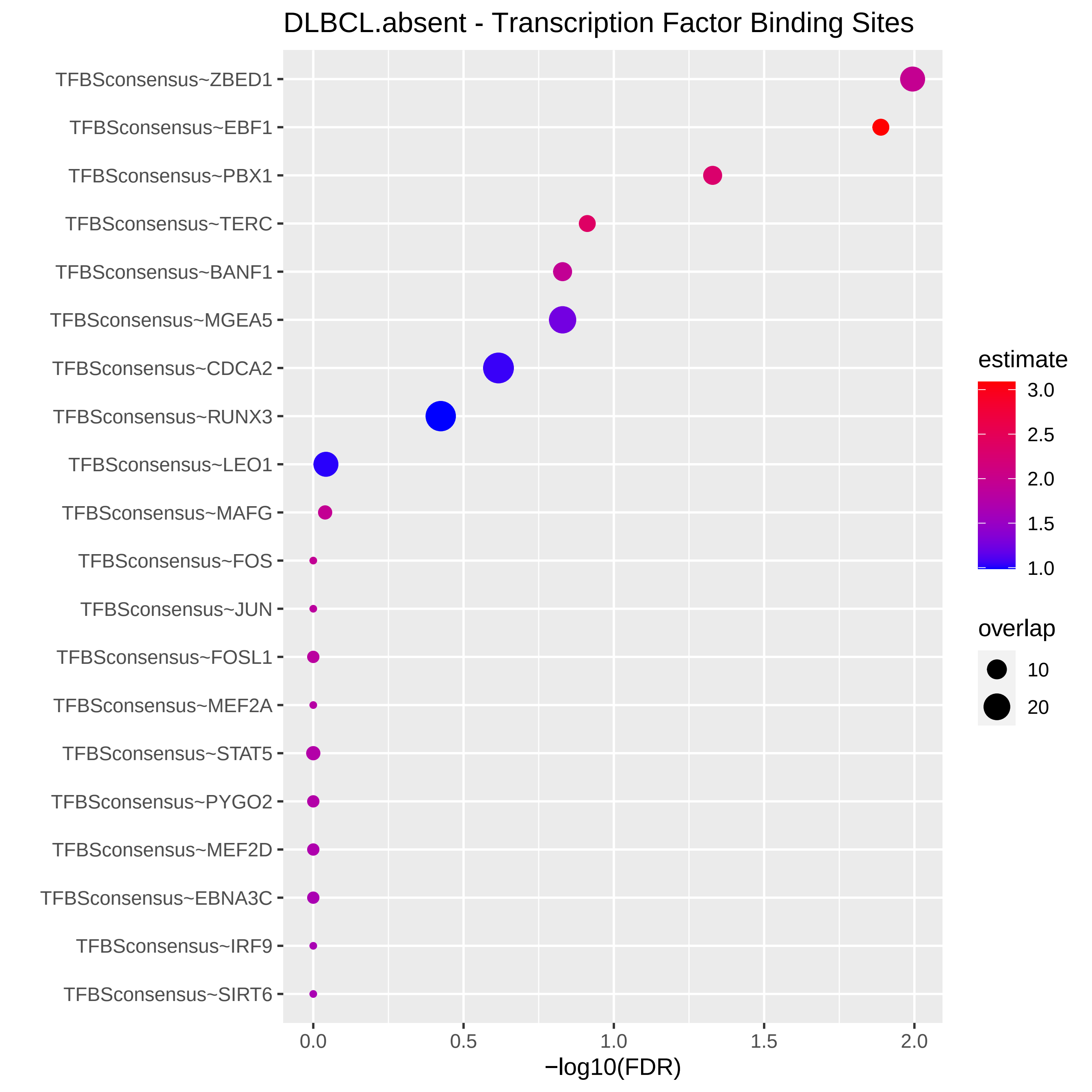
DLBCL.absent - Transcription Factor Binding Sites
TFBSconsensus~ZBED1
TFBSconsensus~EBF1
TFBSconsensus~PBX1
TFBSconsensus~TERC
TFBSconsensus~BANF1
TFBSconsensus~MGEA5
TFBSconsensus~CDCA2
TFBSconsensus~RUNX3
TFBSconsensus~LEO1
TFBSconsensus~MAFG
TFBSconsensus~FOS
TFBSconsensus~JUN
TFBSconsensus~FOSL1
TFBSconsensus~MEF2A
TFBSconsensus~STAT5
TFBSconsensus~PYGO2
TFBSconsensus~MEF2D
TFBSconsensus~EBNA3C
TFBSconsensus~IRF9
TFBSconsensus~SIRT6
0.0
0.5
1.0
1.5
2.0
−log10(FDR)
estimate
3.0
2.5
2.0
1.5
1.0
overlap
10
20

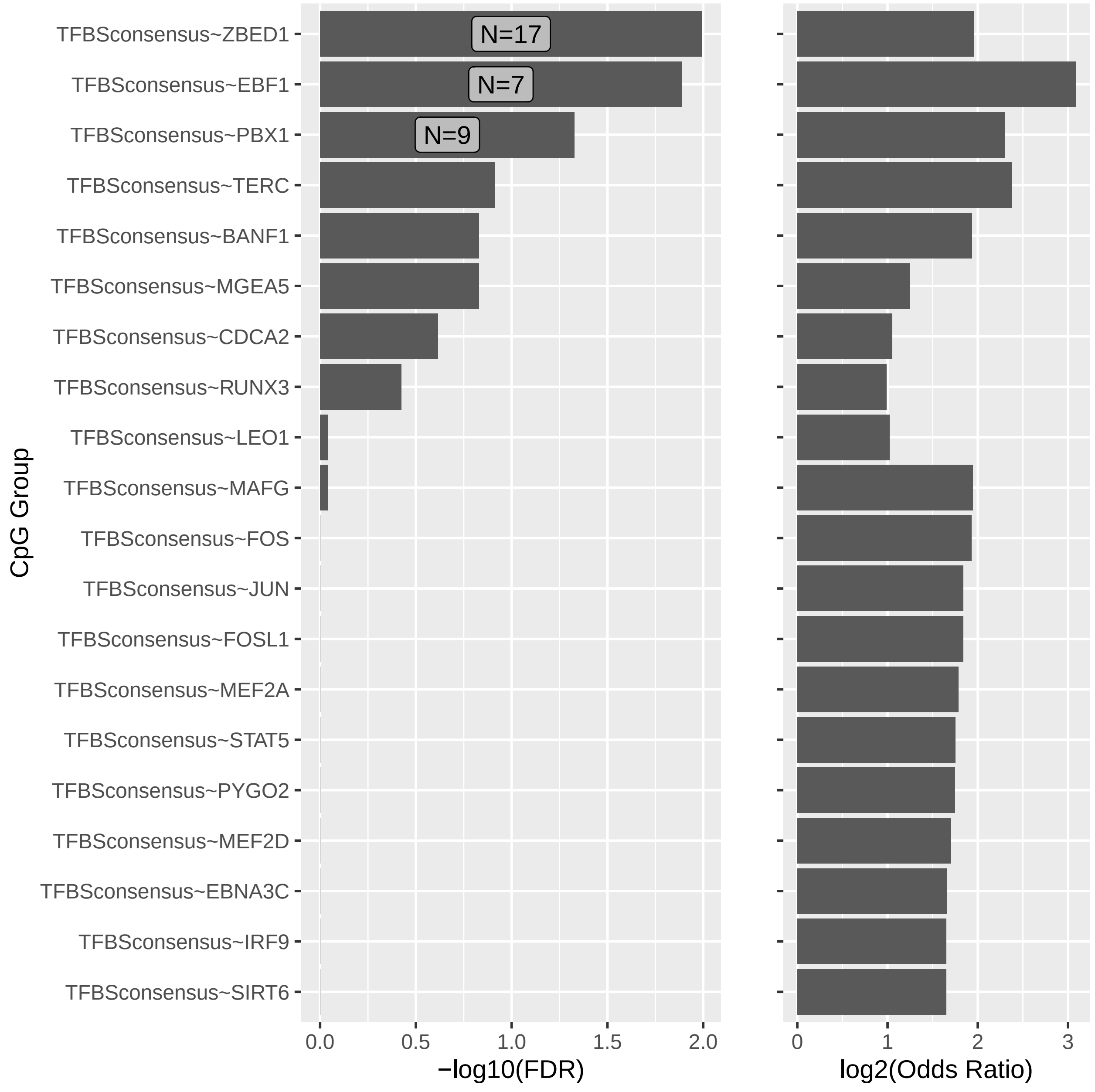

CpG Group
TFBSconsensus~ZBED1
TFBSconsensus~EBF1
TFBSconsensus~PBX1
TFBSconsensus~TERC
TFBSconsensus~BANF1
TFBSconsensus~MGEA5
TFBSconsensus~CDCA2
TFBSconsensus~RUNX3
TFBSconsensus~LEO1
TFBSconsensus~MAFG
TFBSconsensus~FOS
TFBSconsensus~JUN
TFBSconsensus~FOSL1
TFBSconsensus~MEF2A
TFBSconsensus~STAT5
TFBSconsensus~PYGO2
TFBSconsensus~MEF2D
TFBSconsensus~EBNA3C
TFBSconsensus~IRF9
TFBSconsensus~SIRT6
N=17
N=7
N=9
0.0
0.5
1.0
1.5
2.0
−log10(FDR)
0
1
2
3
log2(Odds Ratio)

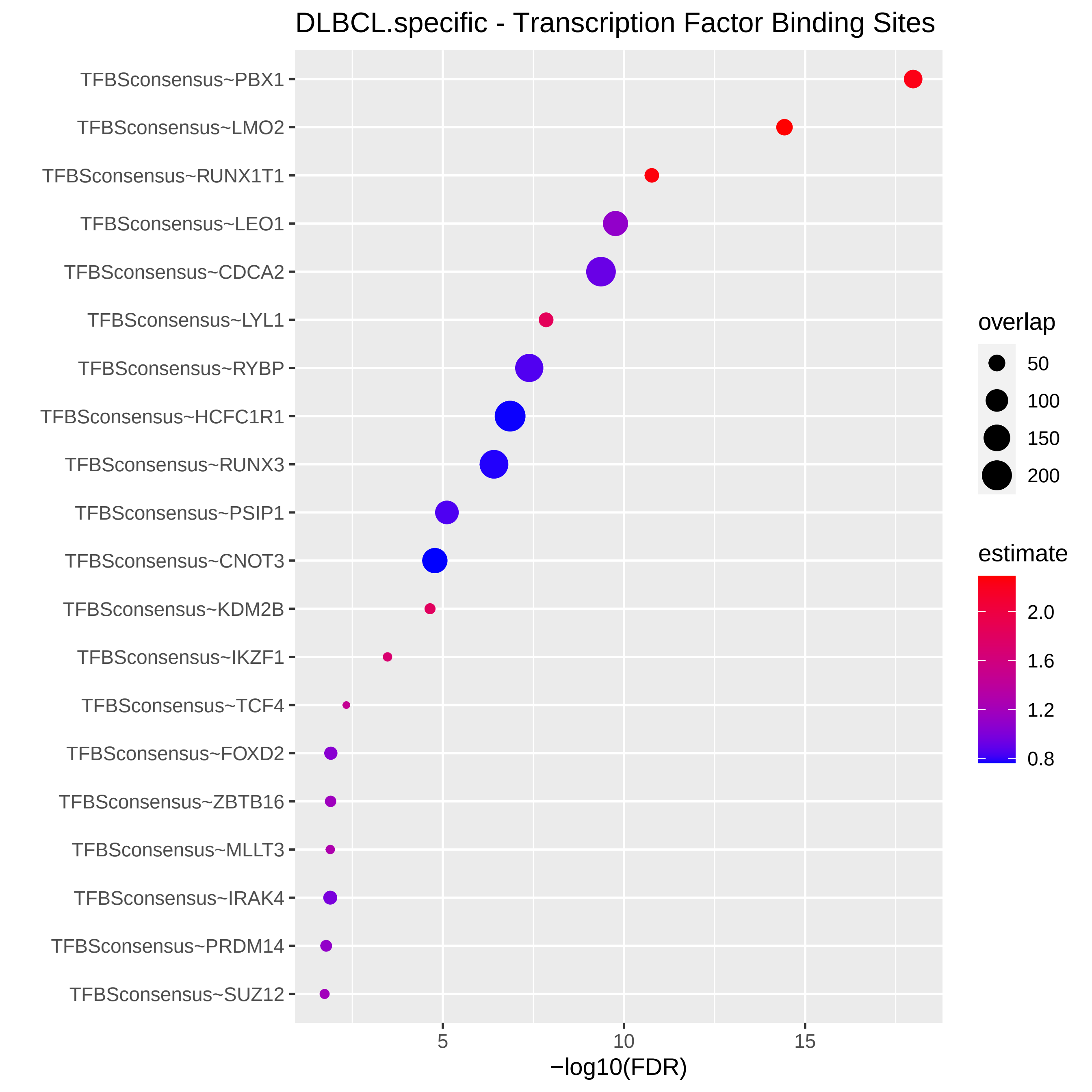
DLBCL.specific - Transcription Factor Binding Sites
TFBSconsensus~PBX1
TFBSconsensus~LMO2
TFBSconsensus~RUNX1T1
TFBSconsensus~LEO1
TFBSconsensus~CDCA2
TFBSconsensus~LYL1
TFBSconsensus~RYBP
TFBSconsensus~HCFC1R1
TFBSconsensus~RUNX3
TFBSconsensus~PSIP1
TFBSconsensus~CNOT3
TFBSconsensus~KDM2B
TFBSconsensus~IKZF1
TFBSconsensus~TCF4
TFBSconsensus~FOXD2
TFBSconsensus~ZBTB16
TFBSconsensus~MLLT3
TFBSconsensus~IRAK4
TFBSconsensus~PRDM14
TFBSconsensus~SUZ12
5
10
15
−log10(FDR)
overlap
50
100
150
200
estimate
2.0
1.6
1.2
0.8

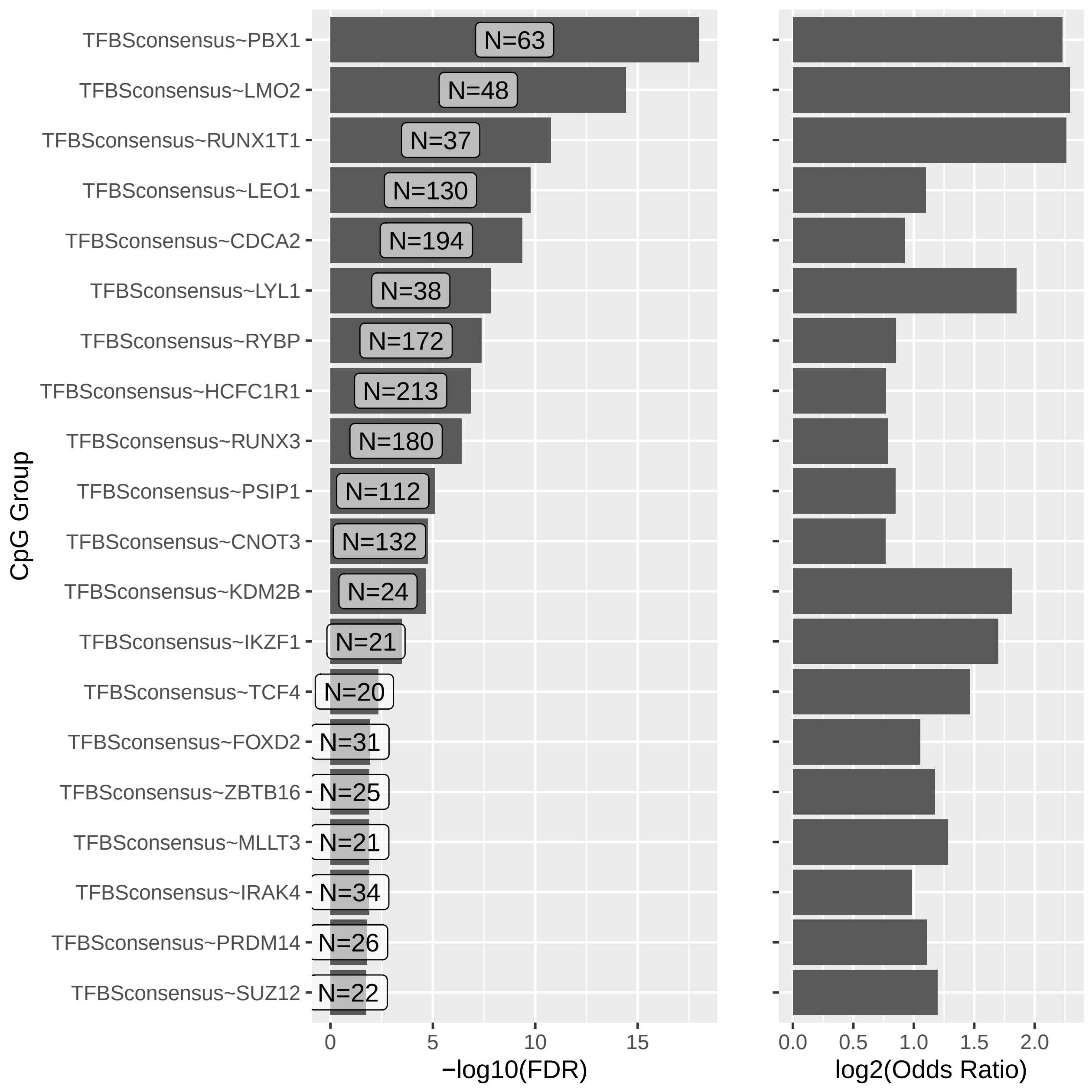

CpG Group
TFBSconsensus~PBX1
TFBSconsensus~LMO2
TFBSconsensus~RUNX1T1
TFBSconsensus~LEO1
TFBSconsensus~CDCA2
TFBSconsensus~LYL1
TFBSconsensus~RYBP
TFBSconsensus~HCFC1R1
TFBSconsensus~RUNX3
TFBSconsensus~PSIP1
TFBSconsensus~CNOT3
TFBSconsensus~KDM2B
TFBSconsensus~IKZF1
TFBSconsensus~TCF4
TFBSconsensus~FOXD2
TFBSconsensus~ZBTB16
TFBSconsensus~MLLT3
TFBSconsensus~IRAK4
TFBSconsensus~PRDM14
TFBSconsensus~SUZ12
N=63
N=48
N=37
N=130
N=194
N=38
N=172
N=213
N=180
N=112
N=132
N=24
N=21
N=20
N=31
N=25
N=21
N=34
N=26
N=22
0
5
10
15
−log10(FDR)
0.0
0.5
1.0
1.5
2.0
log2(Odds Ratio)

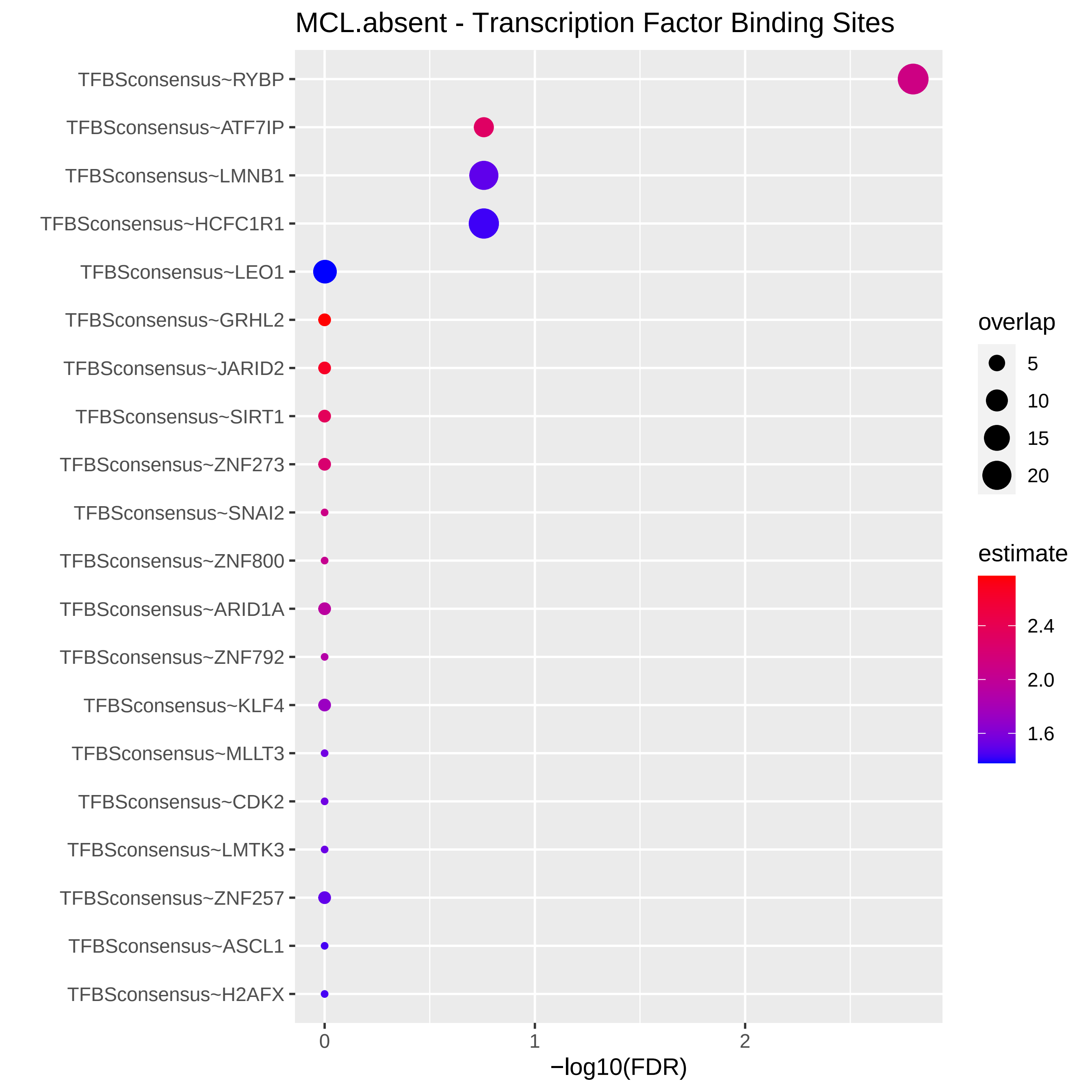
MCL.absent - Transcription Factor Binding Sites
TFBSconsensus~RYBP
TFBSconsensus~ATF7IP
TFBSconsensus~LMNB1
TFBSconsensus~HCFC1R1
TFBSconsensus~LEO1
TFBSconsensus~GRHL2
TFBSconsensus~JARID2
TFBSconsensus~SIRT1
TFBSconsensus~ZNF273
TFBSconsensus~SNAI2
TFBSconsensus~ZNF800
TFBSconsensus~ARID1A
TFBSconsensus~ZNF792
TFBSconsensus~KLF4
TFBSconsensus~MLLT3
TFBSconsensus~CDK2
TFBSconsensus~LMTK3
TFBSconsensus~ZNF257
TFBSconsensus~ASCL1
TFBSconsensus~H2AFX
0
1
2
−log10(FDR)
overlap
5
10
15
20
estimate
2.4
2.0
1.6

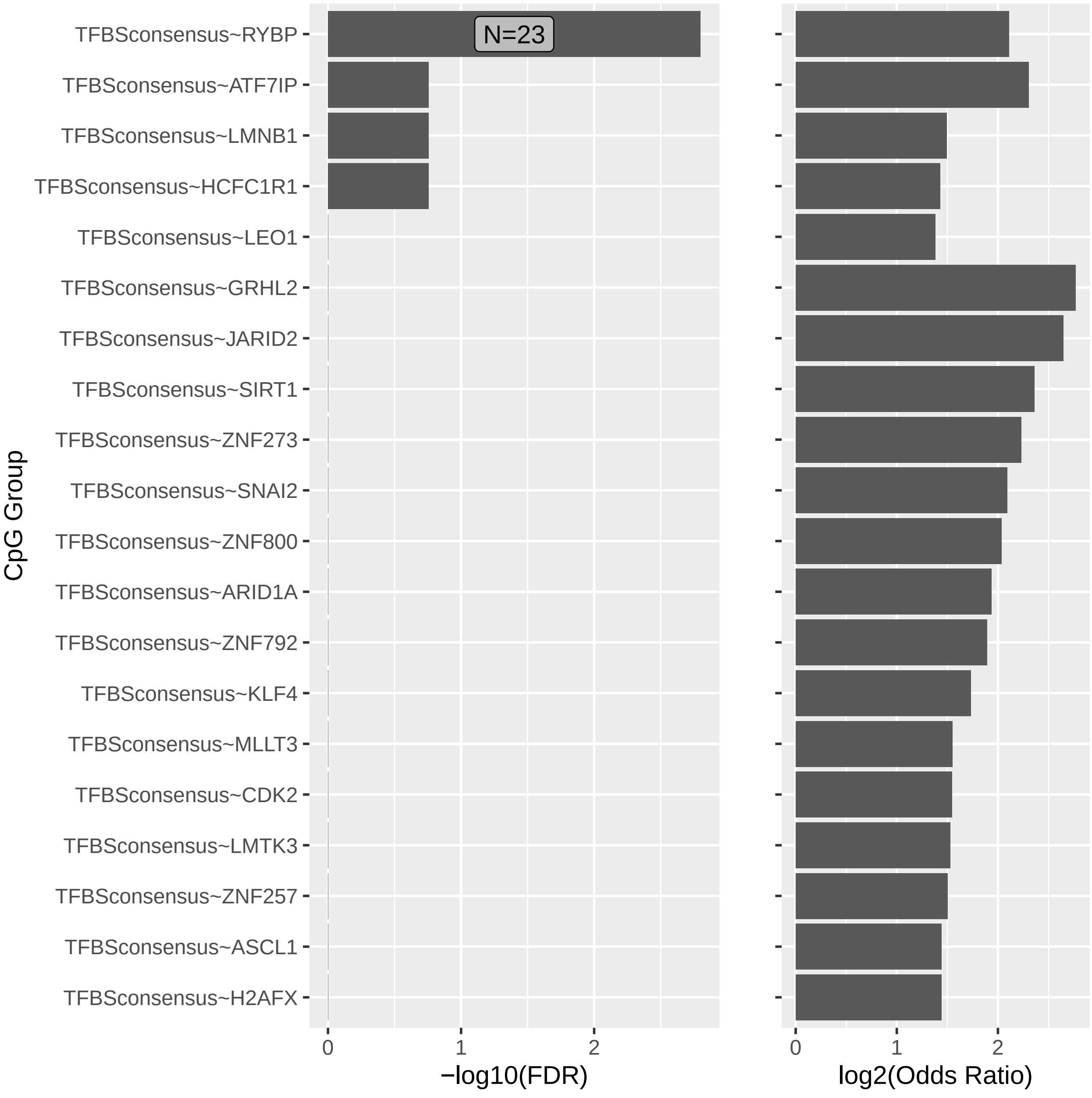

N=23
TFBSconsensus~RYBP
TFBSconsensus~ATF7IP
TFBSconsensus~LMNB1
TFBSconsensus~HCFC1R1
TFBSconsensus~LEO1
TFBSconsensus~GRHL2
TFBSconsensus~JARID2
TFBSconsensus~SIRT1
TFBSconsensus~ZNF273
TFBSconsensus~SNAI2
TFBSconsensus~ZNF800
TFBSconsensus~ARID1A
TFBSconsensus~ZNF792
TFBSconsensus~KLF4
TFBSconsensus~MLLT3
TFBSconsensus~CDK2
TFBSconsensus~LMTK3
TFBSconsensus~ZNF257
TFBSconsensus~ASCL1
TFBSconsensus~H2AFX
CpG Group
0
1
2
−log10(FDR)
0
1
2
log2(Odds Ratio)

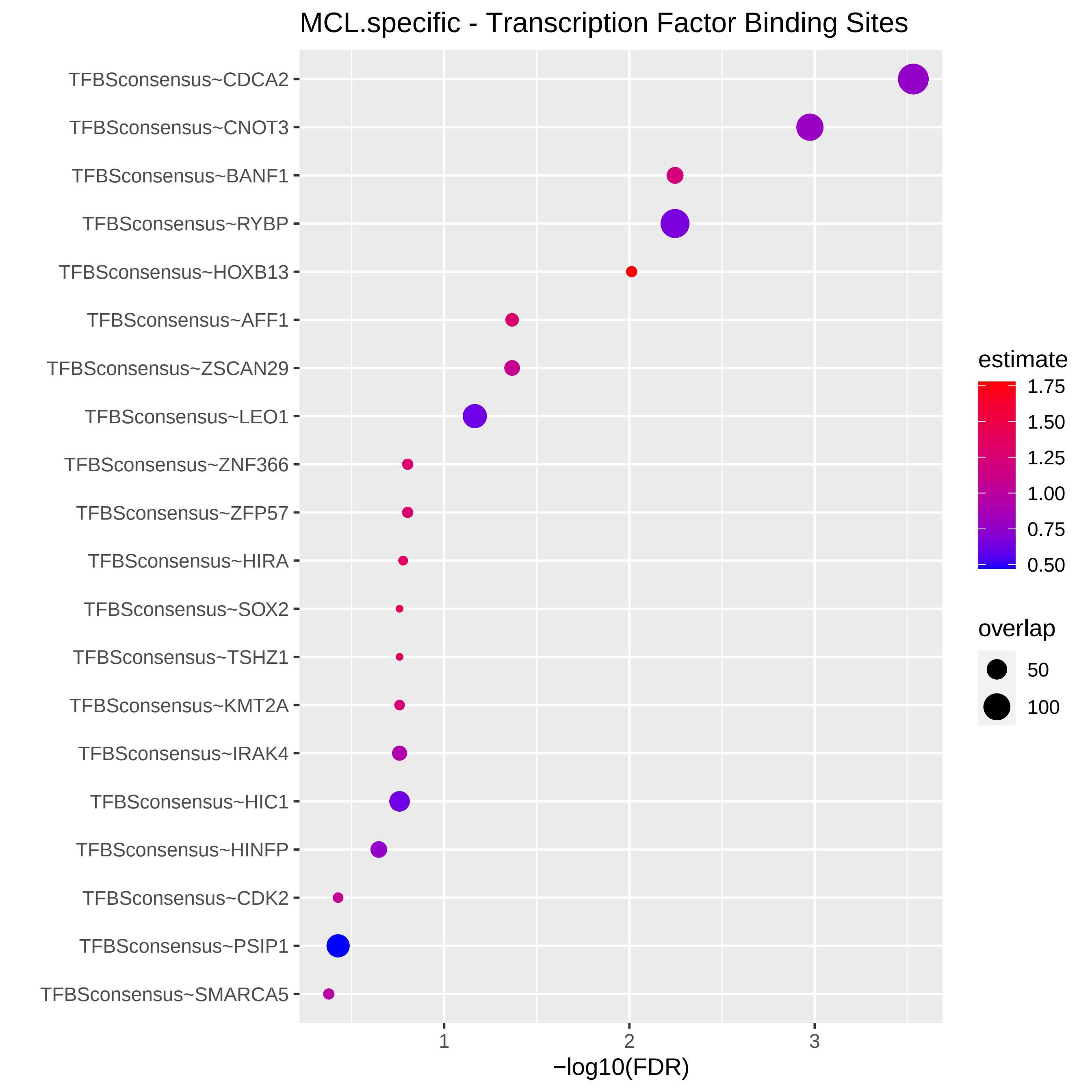

MCL.specific - Transcription Factor Binding Sites
TFBSconsensus~CDCA2
TFBSconsensus~CNOT3
TFBSconsensus~BANF1
TFBSconsensus~RYBP
TFBSconsensus~HOXB13
TFBSconsensus~AFF1
TFBSconsensus~ZSCAN29
TFBSconsensus~LEO1
TFBSconsensus~ZNF366
TFBSconsensus~ZFP57
TFBSconsensus~HIRA
TFBSconsensus~SOX2
TFBSconsensus~TSHZ1
TFBSconsensus~KMT2A
TFBSconsensus~IRAK4
TFBSconsensus~HIC1
TFBSconsensus~HINFP
TFBSconsensus~CDK2
TFBSconsensus~PSIP1
TFBSconsensus~SMARCA5
1
2
3
−log10(FDR)
estimate
1.75
1.50
1.25
1.00
0.75
0.50
overlap
50
100

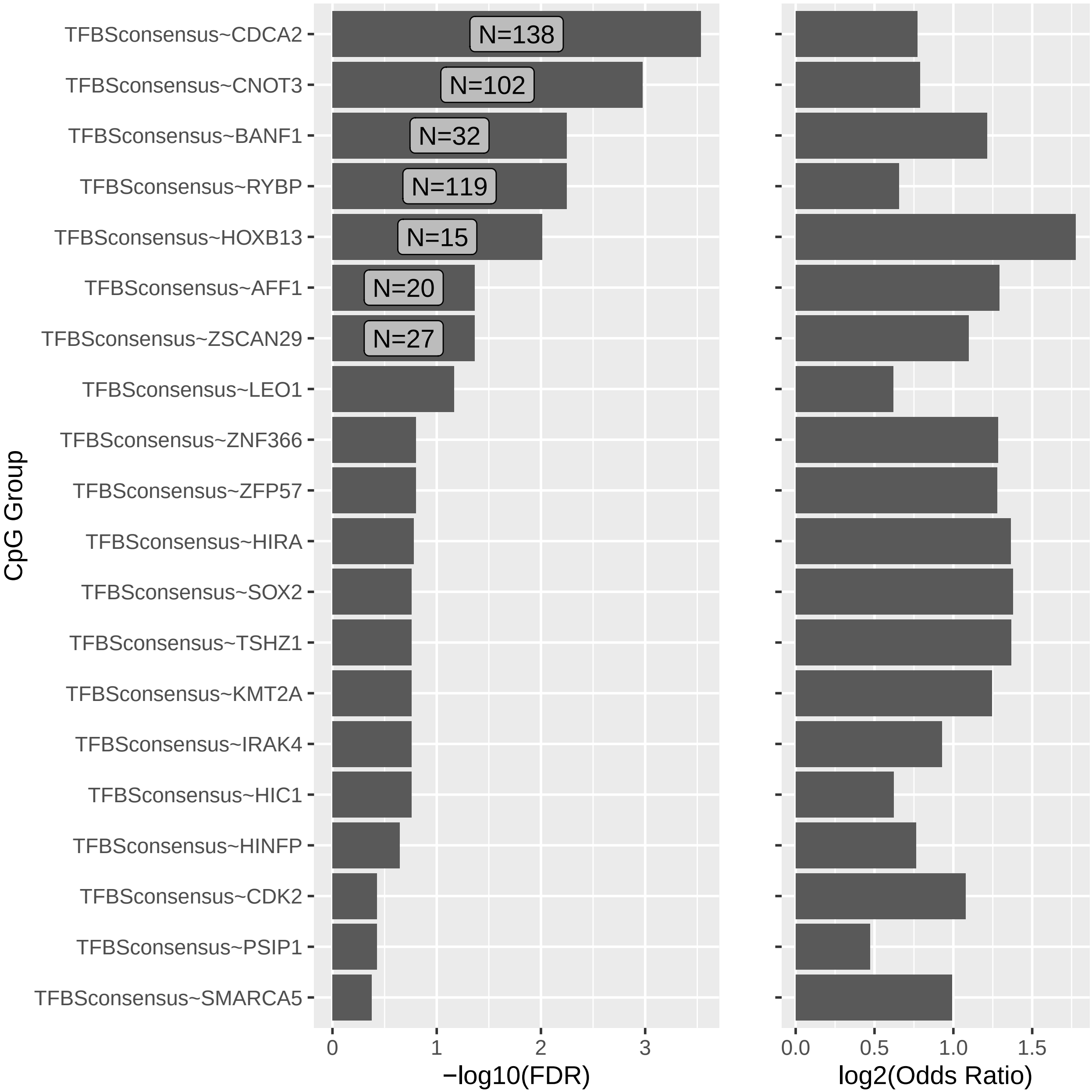

TFBSconsensus~CDCA2
TFBSconsensus~CNOT3
TFBSconsensus~BANF1
TFBSconsensus~RYBP
TFBSconsensus~HOXB13
TFBSconsensus~AFF1
TFBSconsensus~ZSCAN29
TFBSconsensus~LEO1
TFBSconsensus~ZNF366
TFBSconsensus~ZFP57
TFBSconsensus~HIRA
TFBSconsensus~SOX2
TFBSconsensus~TSHZ1
TFBSconsensus~KMT2A
TFBSconsensus~IRAK4
TFBSconsensus~HIC1
TFBSconsensus~HINFP
TFBSconsensus~CDK2
TFBSconsensus~PSIP1
TFBSconsensus~SMARCA5
N=138
N=102
N=32
N=119
N=15
N=20
N=27
CpG Group
0
1
2
3
−log10(FDR)
0.0
0.5
1.0
1.5
log2(Odds Ratio)

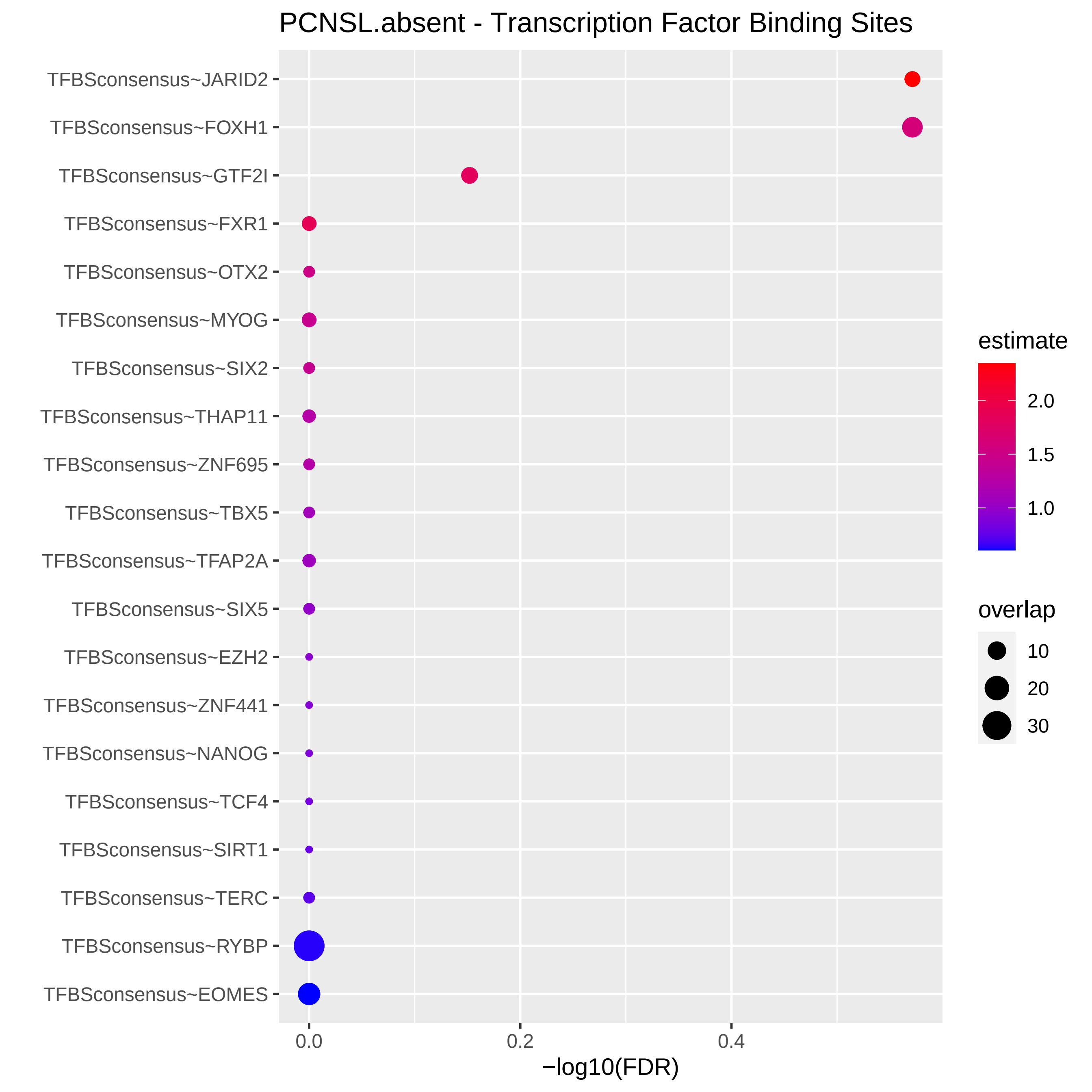
PCNSL.absent - Transcription Factor Binding Sites
TFBSconsensus~JARID2
TFBSconsensus~FOXH1
TFBSconsensus~GTF2I
TFBSconsensus~FXR1
TFBSconsensus~OTX2
TFBSconsensus~MYOG
TFBSconsensus~SIX2
TFBSconsensus~THAP11
TFBSconsensus~ZNF695
TFBSconsensus~TBX5
TFBSconsensus~TFAP2A
TFBSconsensus~SIX5
TFBSconsensus~EZH2
TFBSconsensus~ZNF441
TFBSconsensus~NANOG
TFBSconsensus~TCF4
TFBSconsensus~SIRT1
TFBSconsensus~TERC
TFBSconsensus~RYBP
TFBSconsensus~EOMES
0.0
0.2
0.4
−log10(FDR)
estimate
2.0
1.5
1.0
overlap
10
20
30

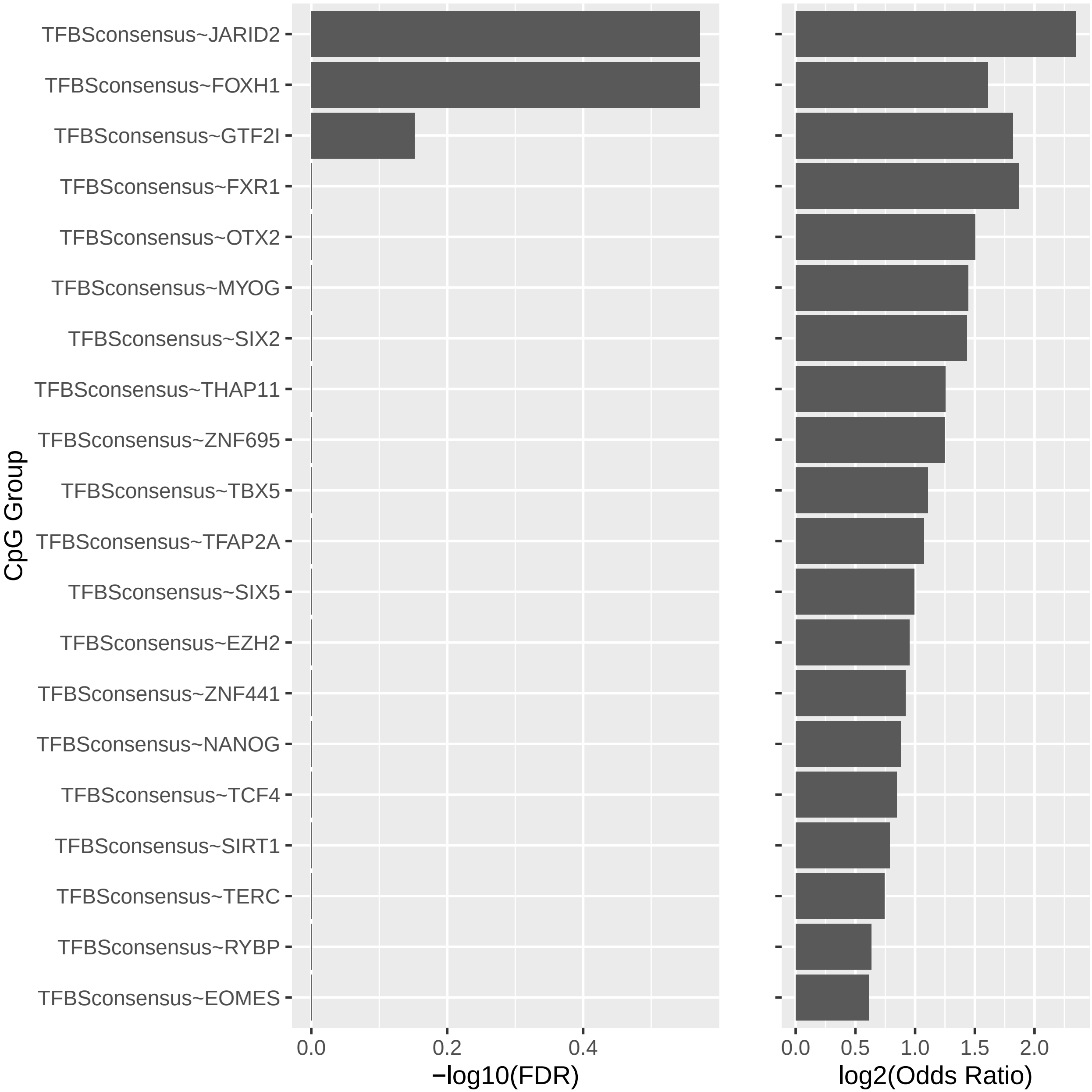

CpG Group
TFBSconsensus~JARID2
TFBSconsensus~FOXH1
TFBSconsensus~GTF2I
TFBSconsensus~FXR1
TFBSconsensus~OTX2
TFBSconsensus~MYOG
TFBSconsensus~SIX2
TFBSconsensus~THAP11
TFBSconsensus~ZNF695
TFBSconsensus~TBX5
TFBSconsensus~TFAP2A
TFBSconsensus~SIX5
TFBSconsensus~EZH2
TFBSconsensus~ZNF441
TFBSconsensus~NANOG
TFBSconsensus~TCF4
TFBSconsensus~SIRT1
TFBSconsensus~TERC
TFBSconsensus~RYBP
TFBSconsensus~EOMES
0.0
0.2
0.4
−log10(FDR)
0.0
0.5
1.0
1.5
2.0
log2(Odds Ratio)

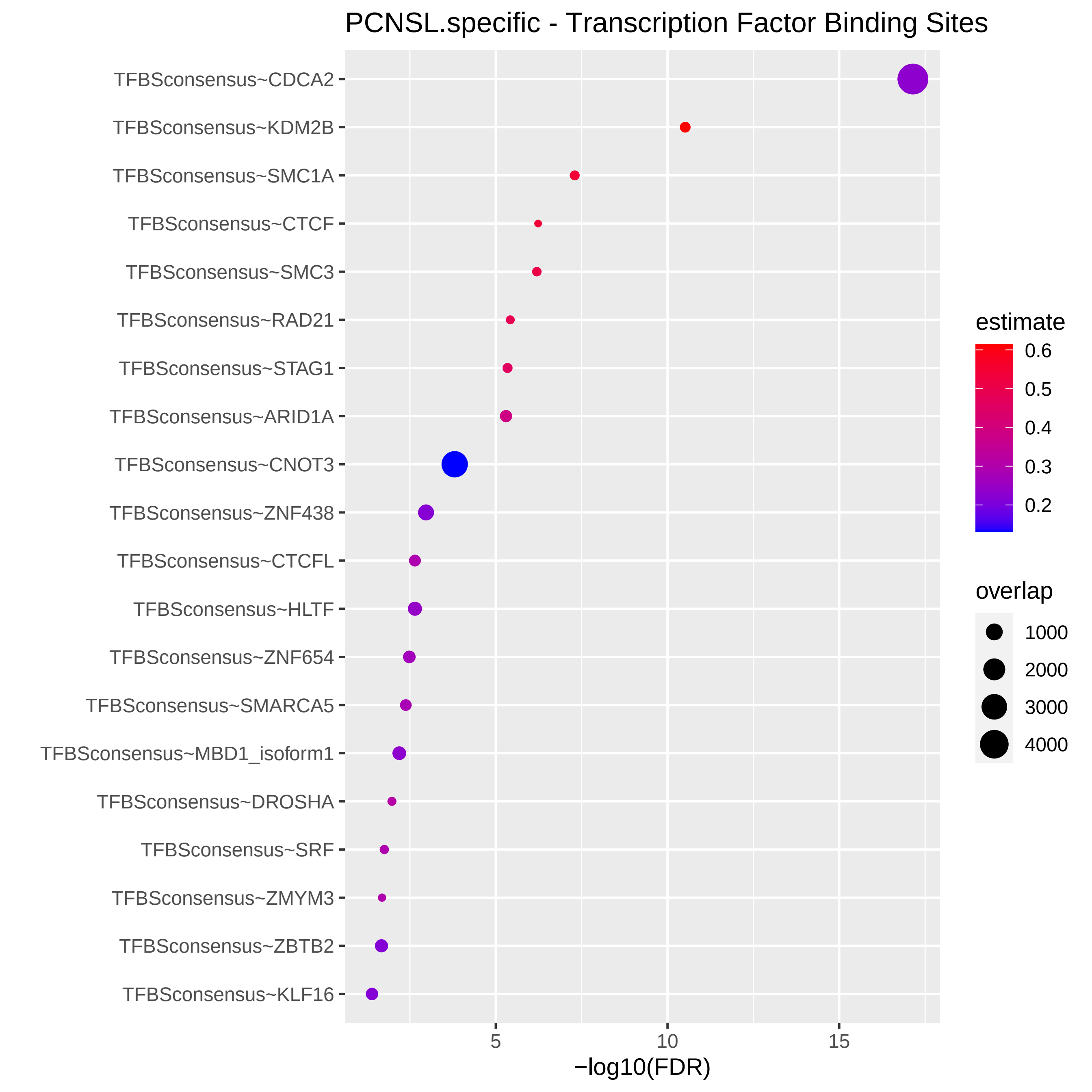

PCNSL.specific - Transcription Factor Binding Sites
TFBSconsensus~CDCA2
TFBSconsensus~KDM2B
TFBSconsensus~SMC1A
TFBSconsensus~CTCF
TFBSconsensus~SMC3
TFBSconsensus~RAD21
TFBSconsensus~STAG1
TFBSconsensus~ARID1A
TFBSconsensus~CNOT3
TFBSconsensus~ZNF438
TFBSconsensus~CTCFL
TFBSconsensus~HLTF
TFBSconsensus~ZNF654
TFBSconsensus~SMARCA5
TFBSconsensus~MBD1_isoform1
TFBSconsensus~DROSHA
TFBSconsensus~SRF
TFBSconsensus~ZMYM3
TFBSconsensus~ZBTB2
TFBSconsensus~KLF16
5
10
15
−log10(FDR)
estimate
0.6
0.5
0.4
0.3
0.2
overlap
1000
2000
3000
4000

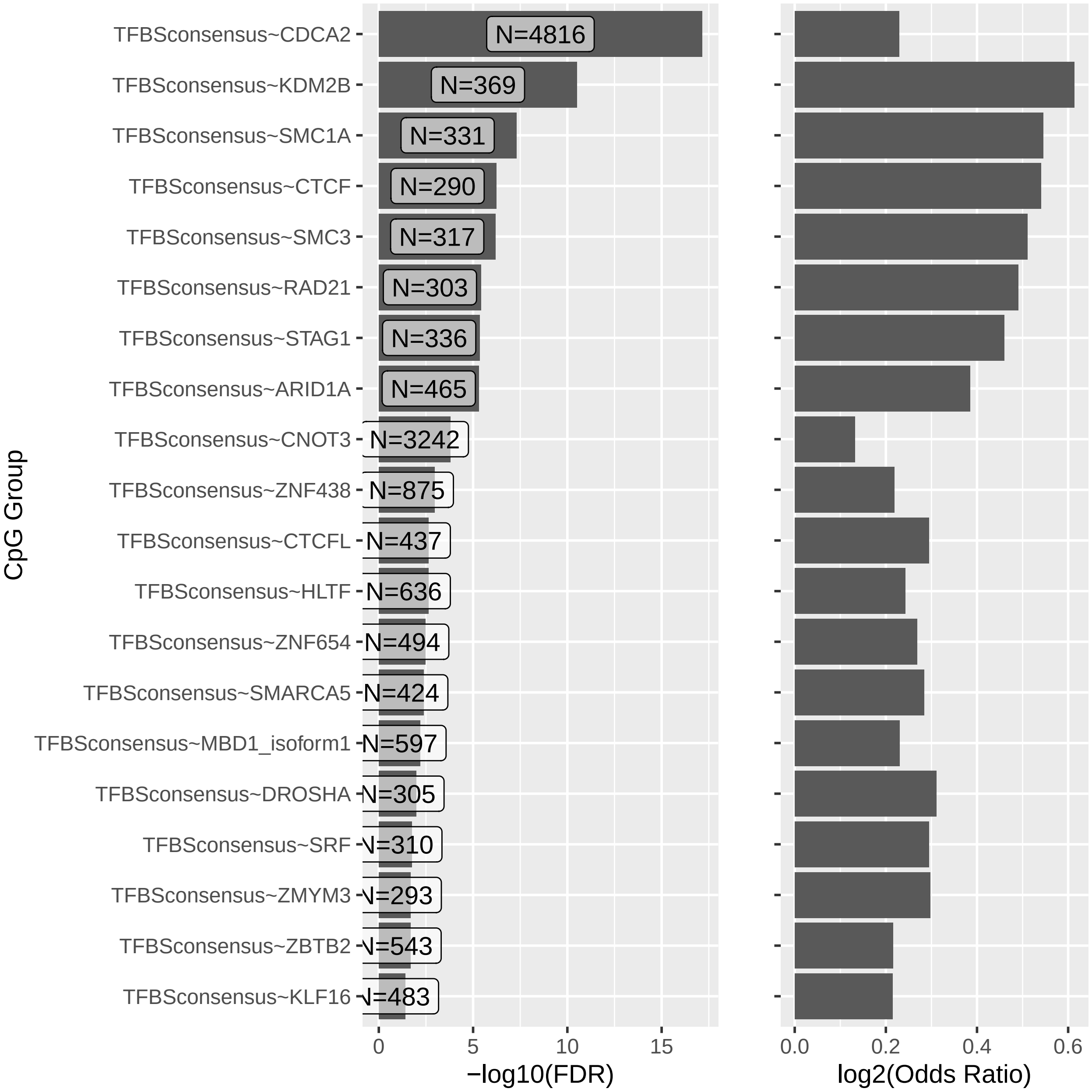

TFBSconsensus~CDCA2
TFBSconsensus~KDM2B
TFBSconsensus~SMC1A
TFBSconsensus~CTCF
TFBSconsensus~SMC3
TFBSconsensus~RAD21
TFBSconsensus~STAG1
TFBSconsensus~ARID1A
TFBSconsensus~CNOT3
TFBSconsensus~ZNF438
TFBSconsensus~CTCFL
TFBSconsensus~HLTF
TFBSconsensus~ZNF654
TFBSconsensus~SMARCA5
TFBSconsensus~MBD1_isoform1
TFBSconsensus~DROSHA
TFBSconsensus~SRF
TFBSconsensus~ZMYM3
TFBSconsensus~ZBTB2
TFBSconsensus~KLF16
CpG Group
N=4816
N=369
N=331
N=290
N=317
N=303
N=336
N=465
N=3242
N=875
N=437
N=636
N=494
N=424
N=597
N=305
0
5
10
15
−log10(FDR)
0.0
0.2
0.4
0.6
log2(Odds Ratio)

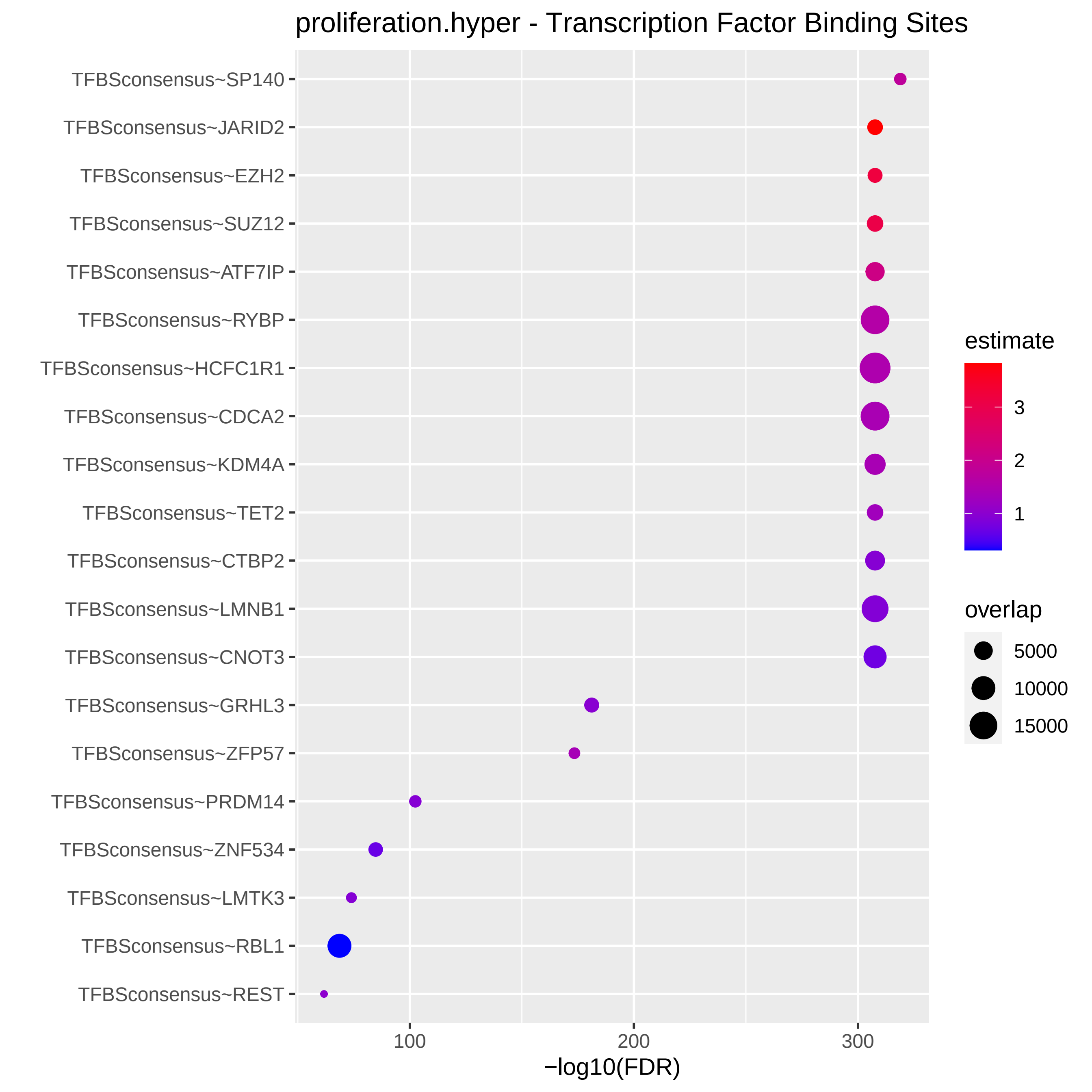

proliferation.hyper - Transcription Factor Binding Sites
TFBSconsensus~SP140
TFBSconsensus~JARID2
TFBSconsensus~EZH2
TFBSconsensus~SUZ12
TFBSconsensus~ATF7IP
TFBSconsensus~RYBP
TFBSconsensus~HCFC1R1
TFBSconsensus~CDCA2
TFBSconsensus~KDM4A
TFBSconsensus~TET2
TFBSconsensus~CTBP2
TFBSconsensus~LMNB1
TFBSconsensus~CNOT3
TFBSconsensus~GRHL3
TFBSconsensus~ZFP57
TFBSconsensus~PRDM14
TFBSconsensus~ZNF534
TFBSconsensus~LMTK3
TFBSconsensus~RBL1
TFBSconsensus~REST
100
200
300
−log10(FDR)
estimate
3
2
1
overlap
5000
10000
15000

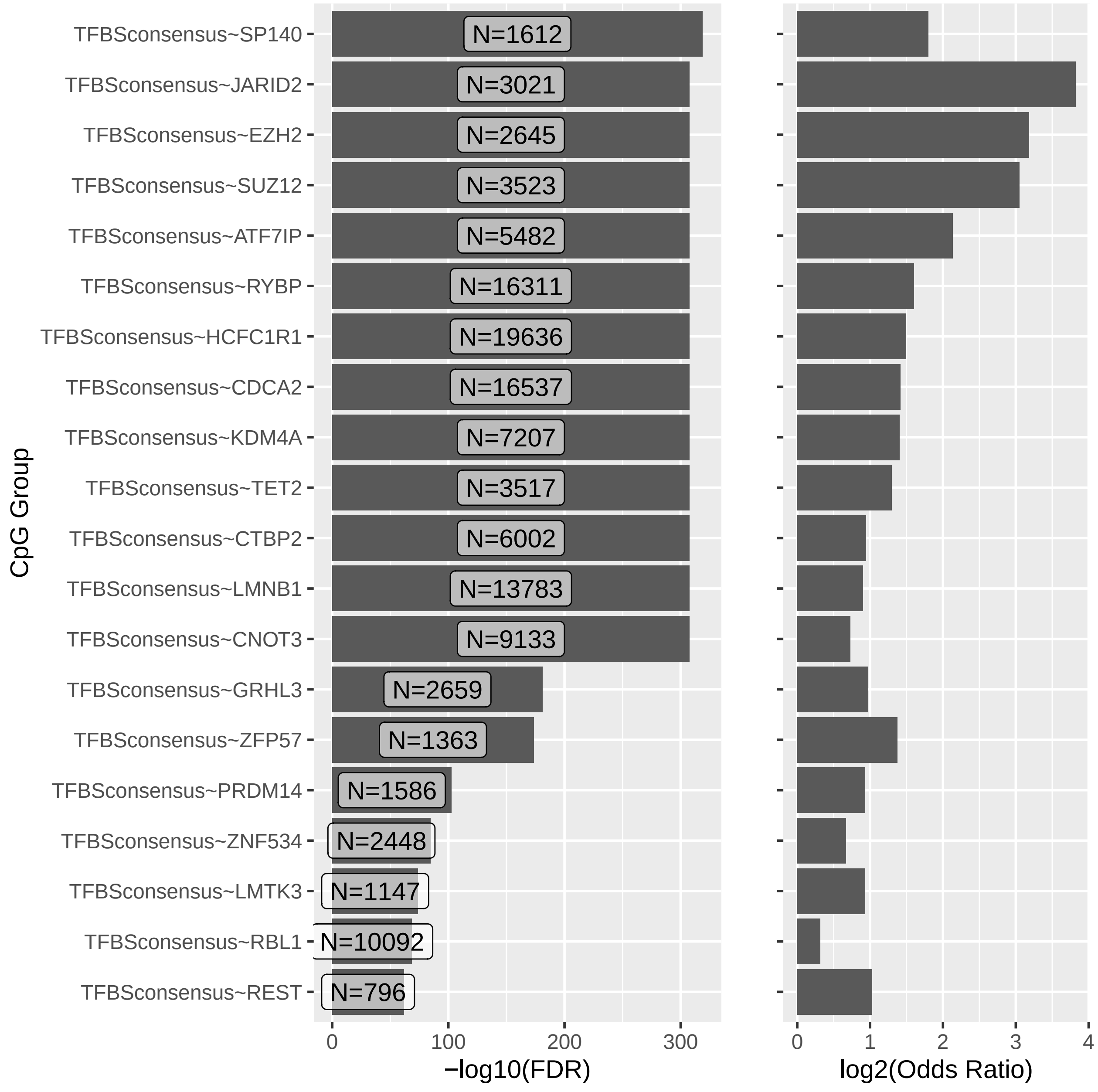

CpG Group
TFBSconsensus~SP140
TFBSconsensus~JARID2
TFBSconsensus~EZH2
TFBSconsensus~SUZ12
TFBSconsensus~ATF7IP
TFBSconsensus~RYBP
TFBSconsensus~HCFC1R1
TFBSconsensus~CDCA2
TFBSconsensus~KDM4A
TFBSconsensus~TET2
TFBSconsensus~CTBP2
TFBSconsensus~LMNB1
TFBSconsensus~CNOT3
TFBSconsensus~GRHL3
TFBSconsensus~ZFP57
TFBSconsensus~PRDM14
TFBSconsensus~ZNF534
TFBSconsensus~LMTK3
TFBSconsensus~RBL1
TFBSconsensus~REST
N=1612
N=3021
N=2645
N=3523
N=5482
N=16311
N=19636
N=16537
N=7207
N=3517
N=6002
N=13783
N=9133
N=2659
N=1363
N=1586
N=2448
N=1147
N=10092
N=796
0
100
200
300
−log10(FDR)
0
1
2
3
4
log2(Odds Ratio)

Supplementary figure 5. Detailed results of SeSAMe analysis for transcription factor binding sites for the different DMR groups, as indicated. For each DMR group the first graph shows the significance of the association with the specific transcription factor binding site adjusted for false discovery rate (−log10(FDR)). The second also includes details of the number of overlapping DMRs overlapping with the specific transcription factor binding site.

Supplementary Figure 6

SLC22A15

Methylation

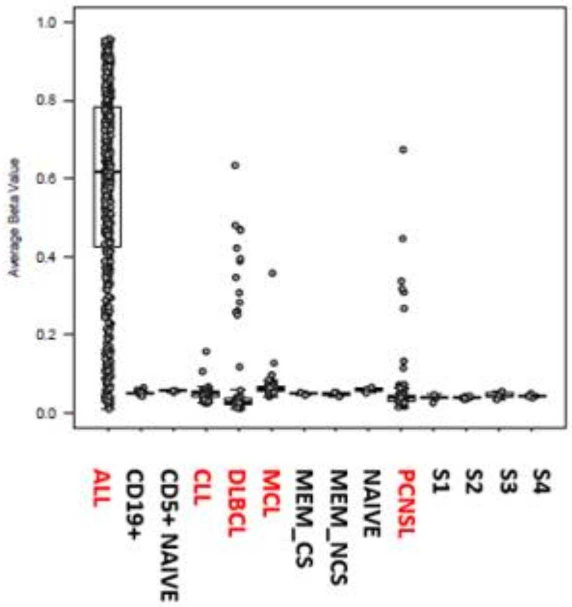

Expression

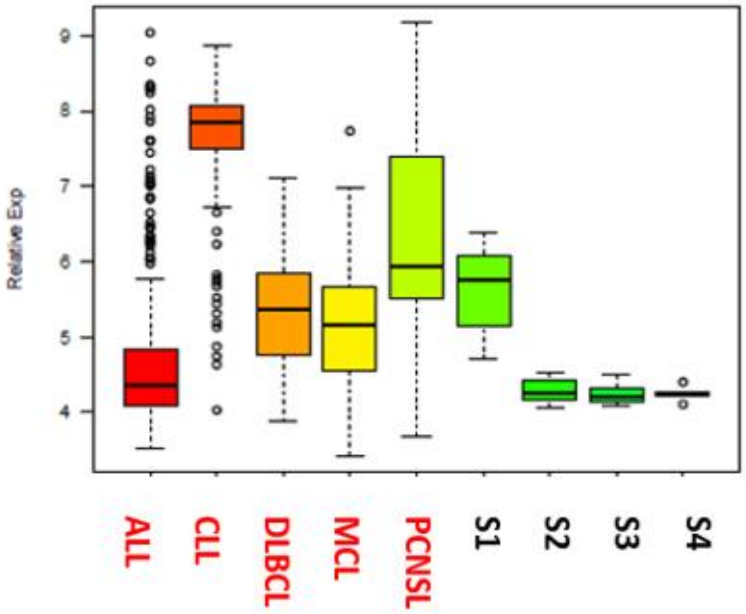

THEM4

Methylation

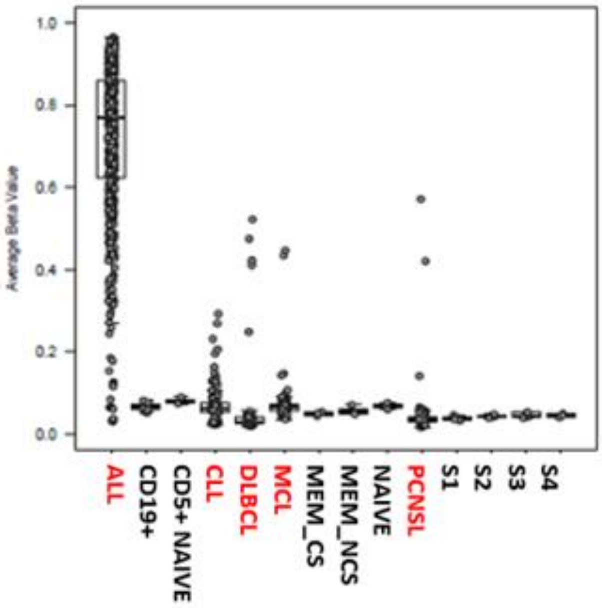

Expression

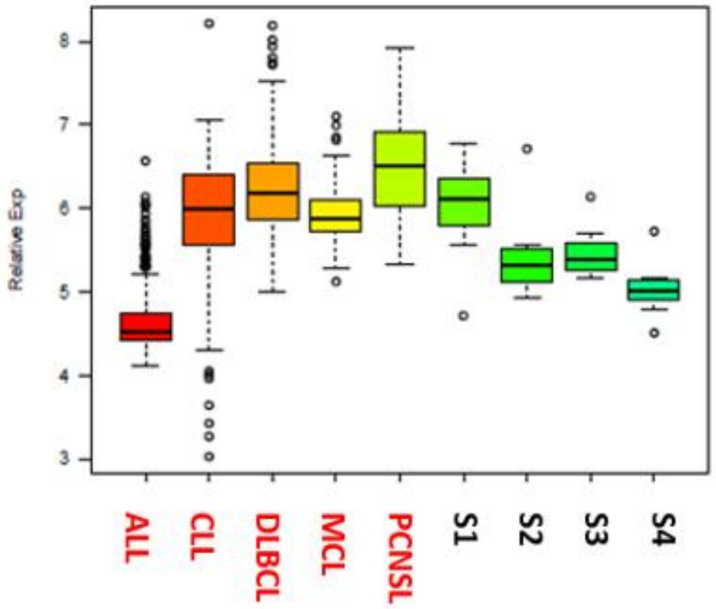

TTC12

Methylation

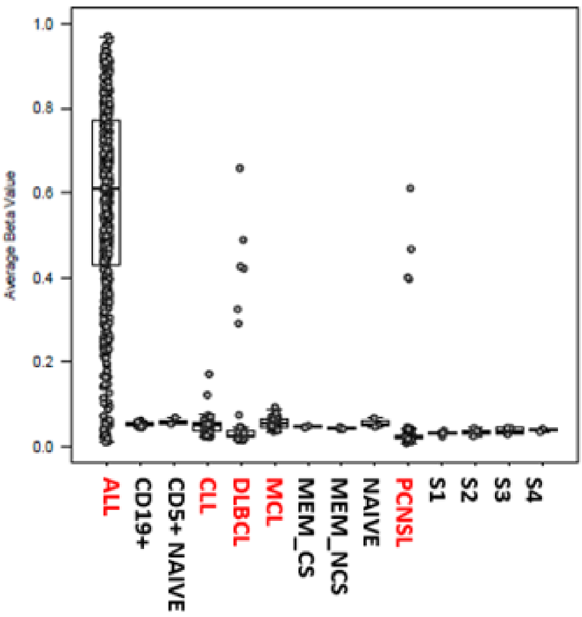

Expression

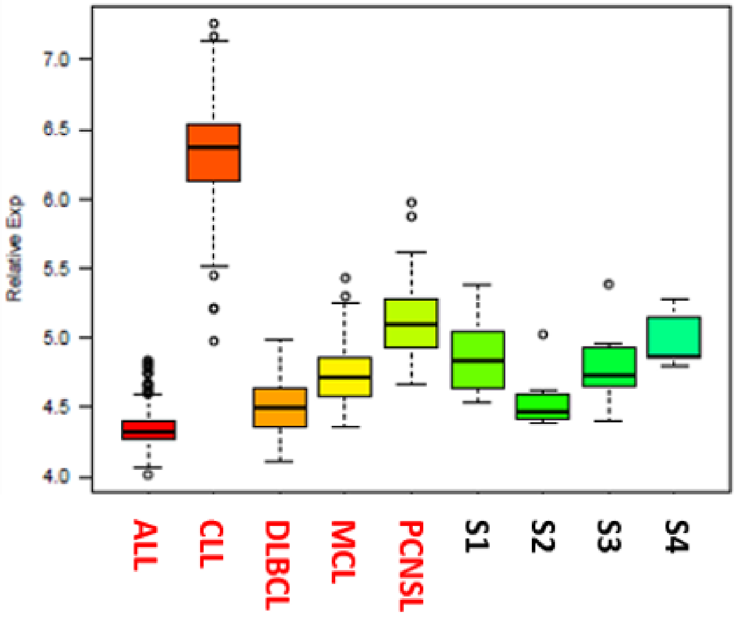

MAP9

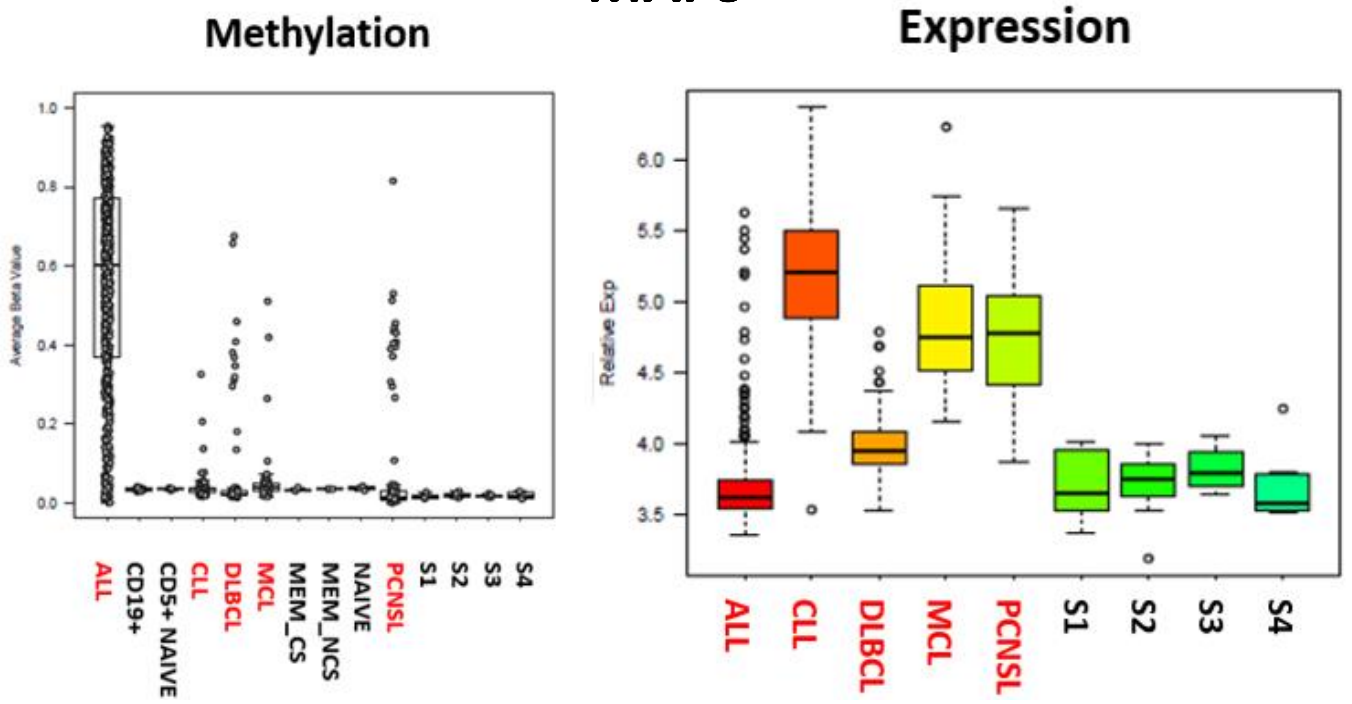

DTD1

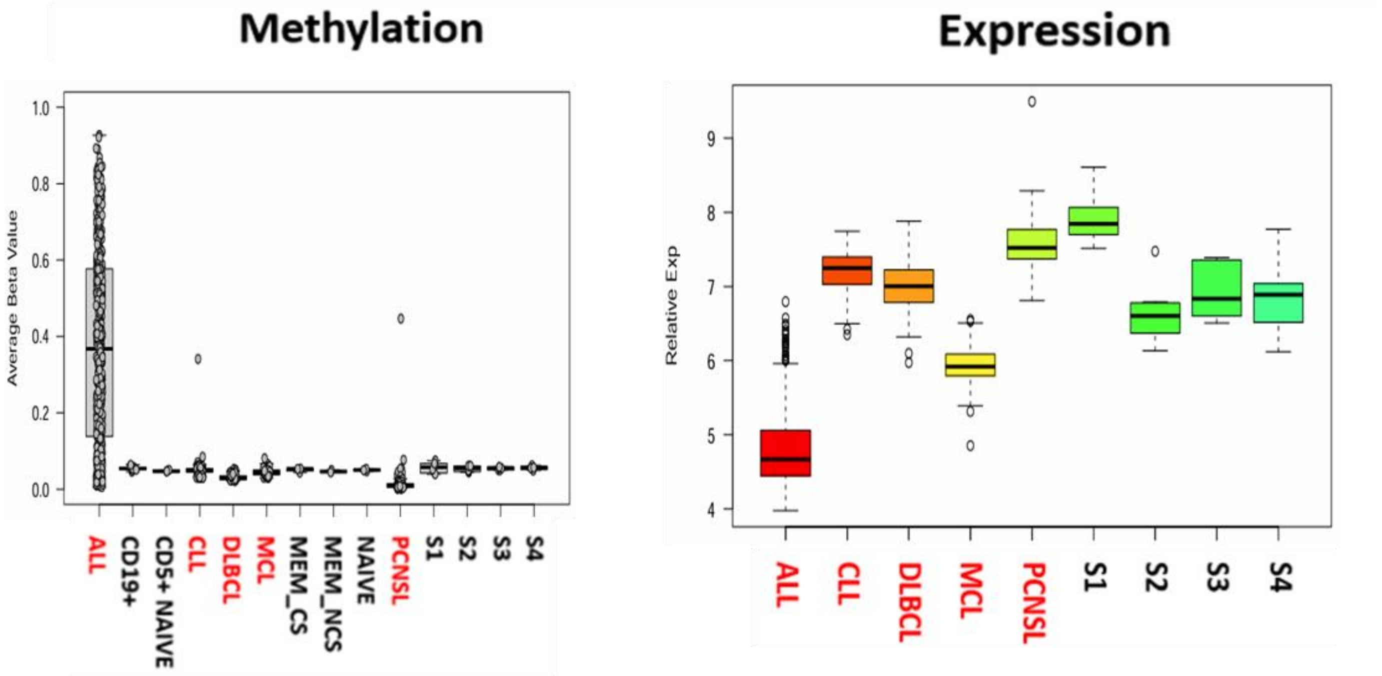

BMP2

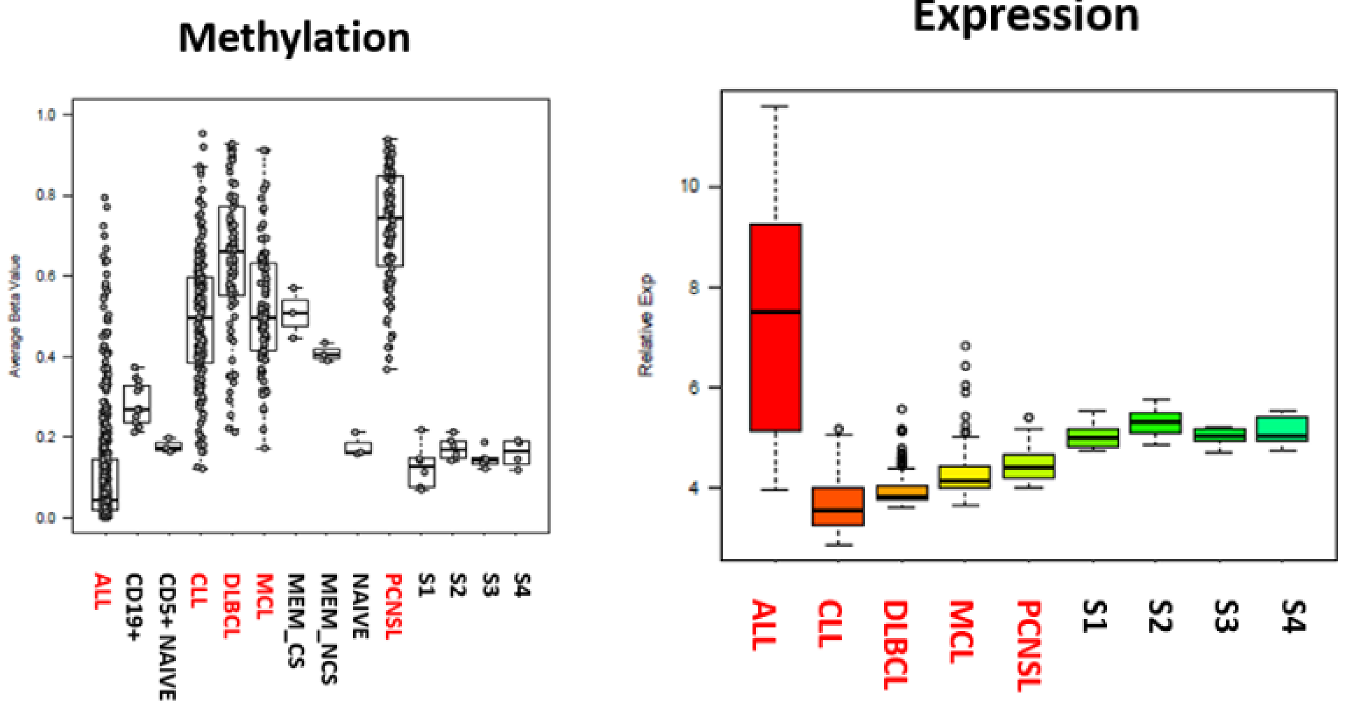

CTGF

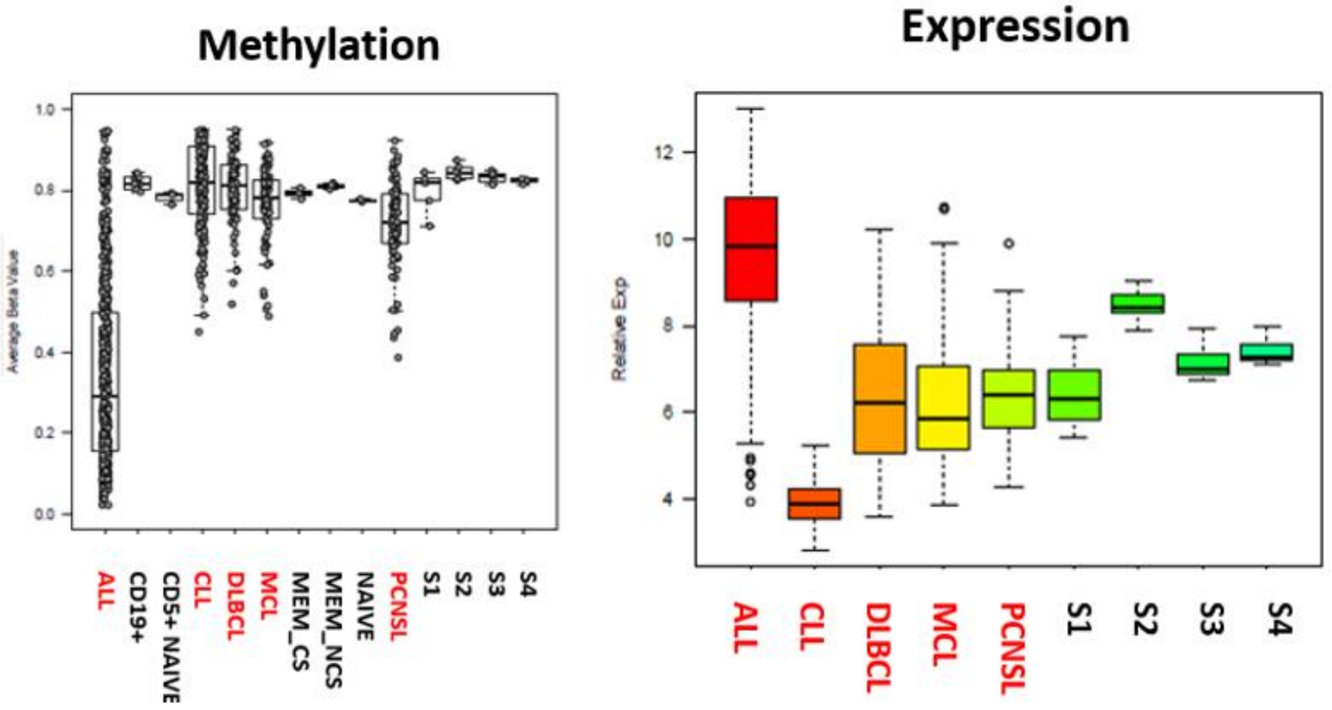

NPY

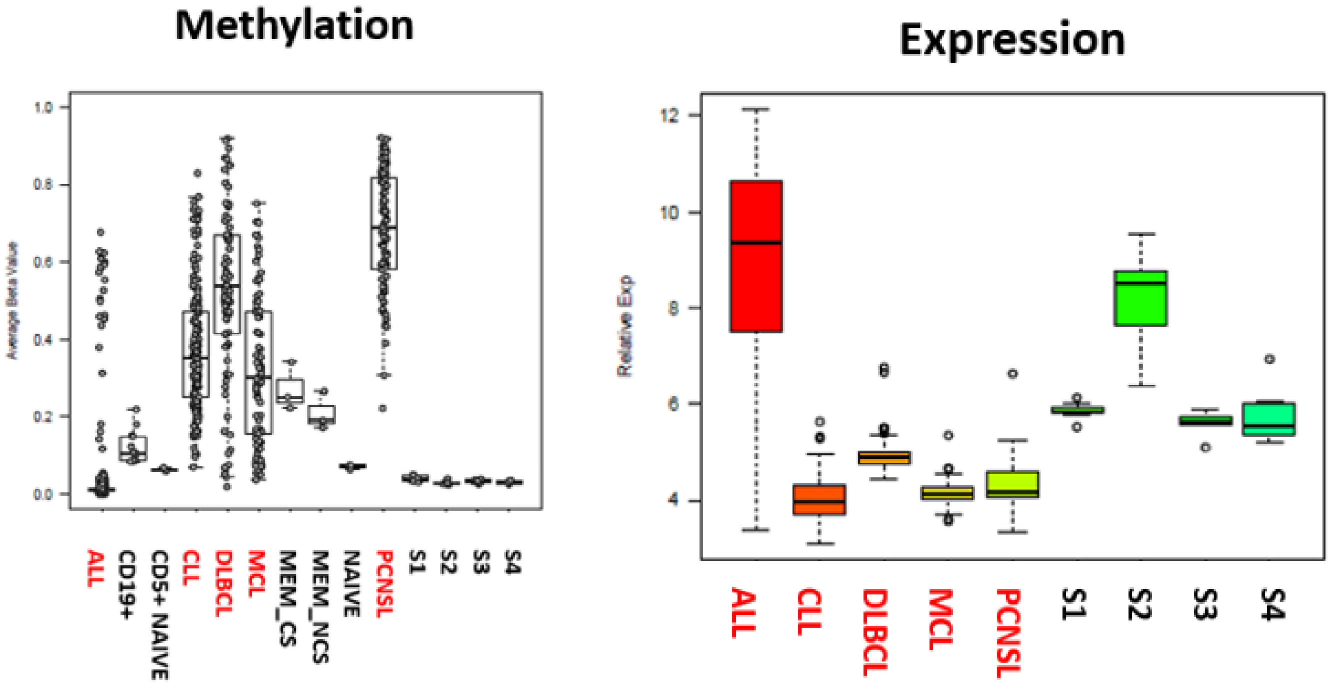

ZNF423

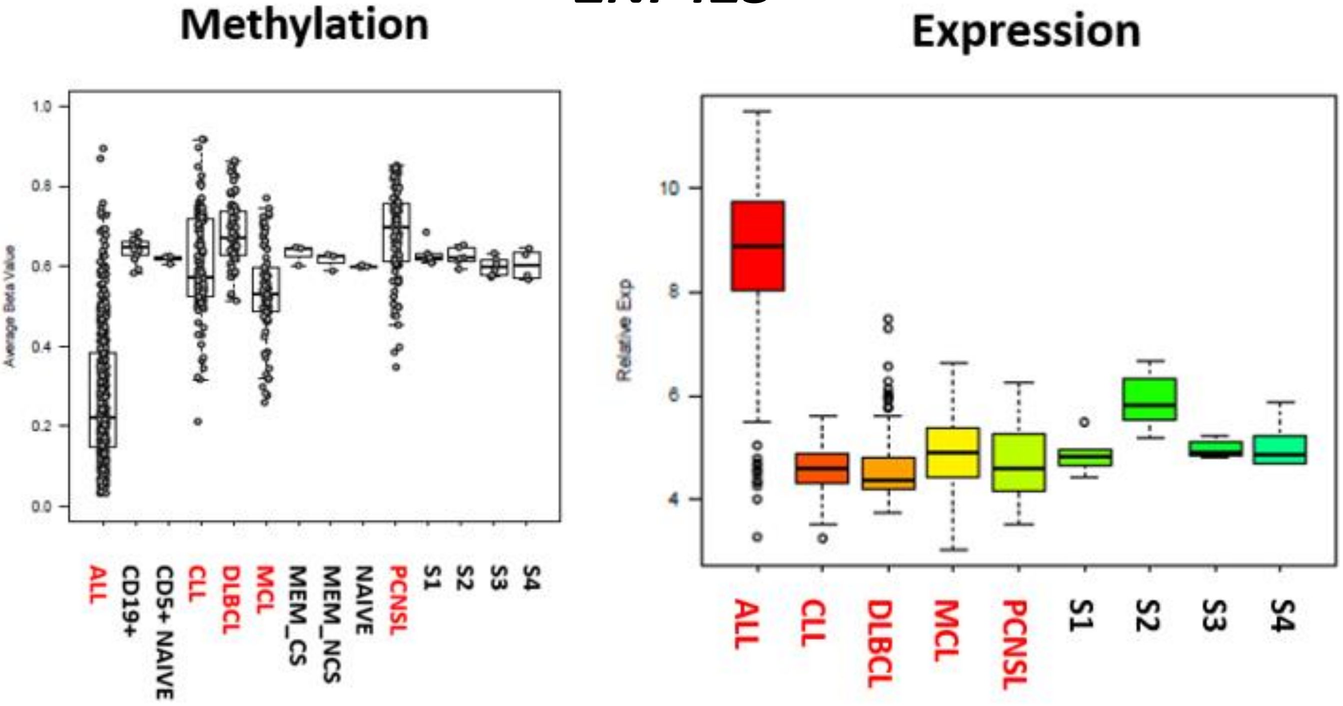

SMIM3

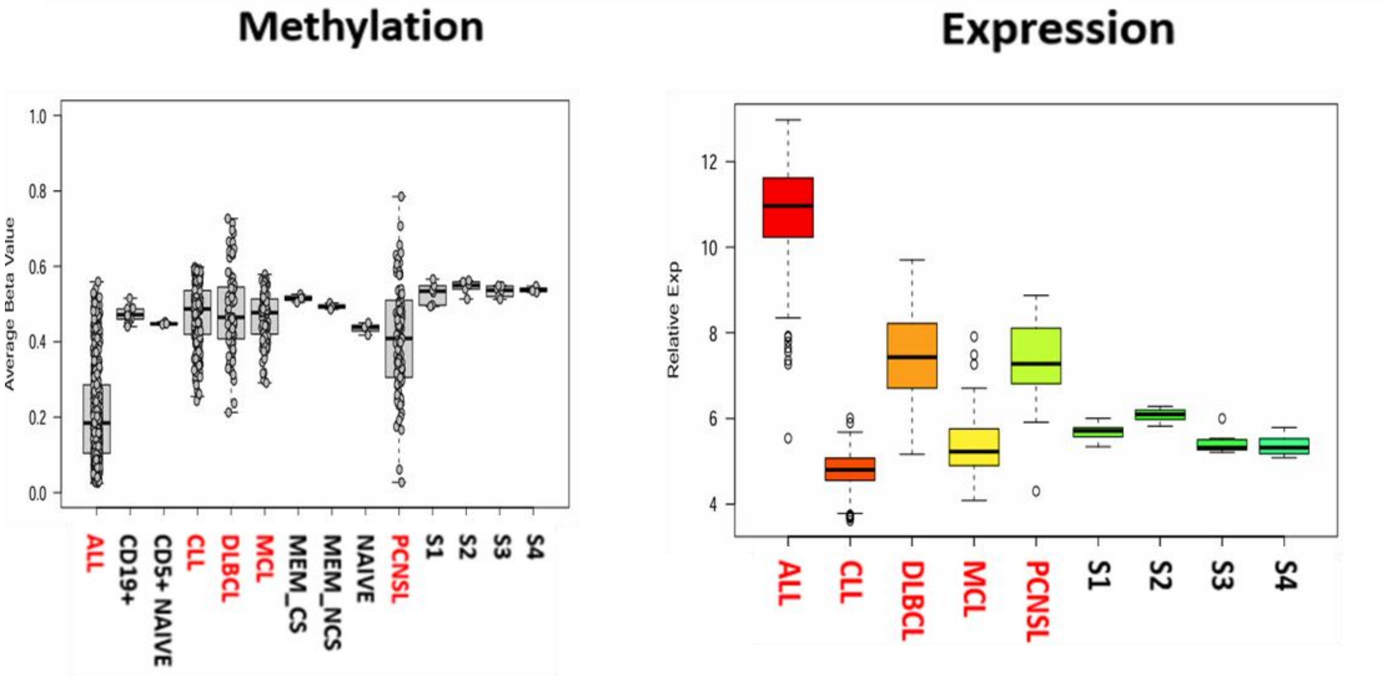

CMTM2

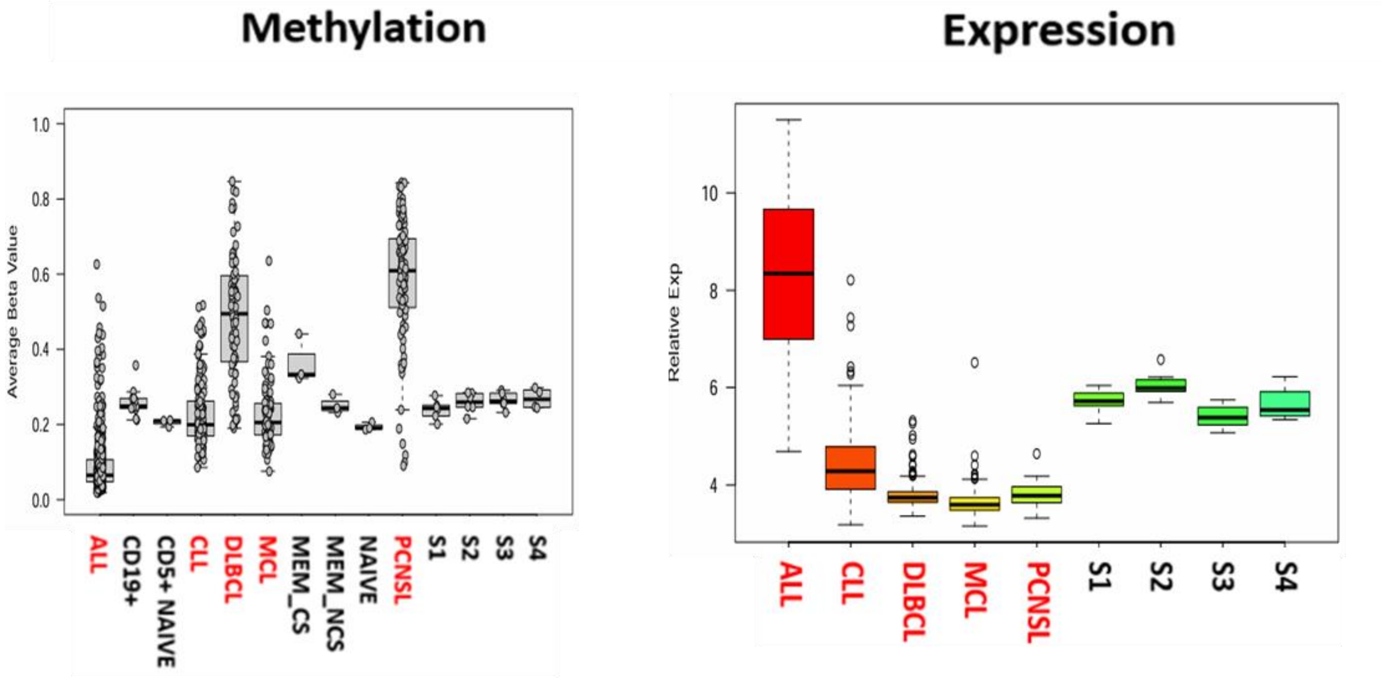

SNX18

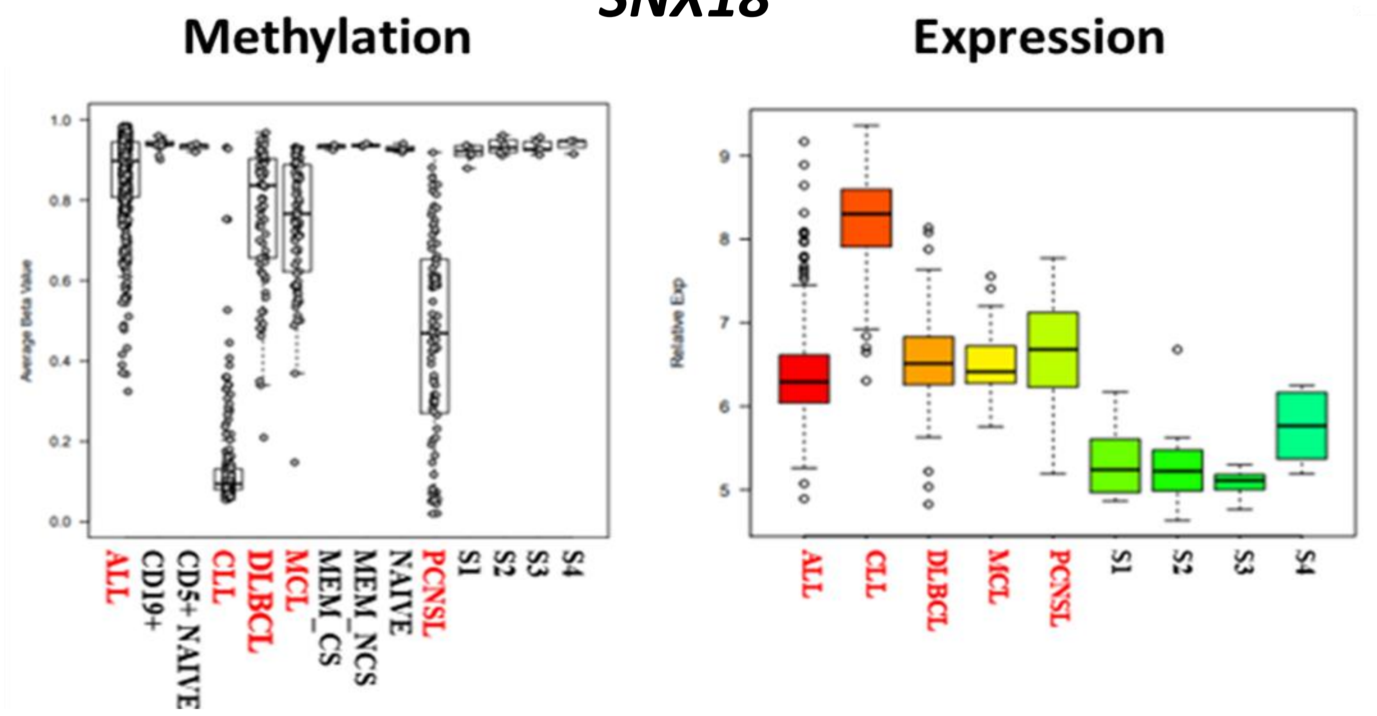

SOX11

Methylation

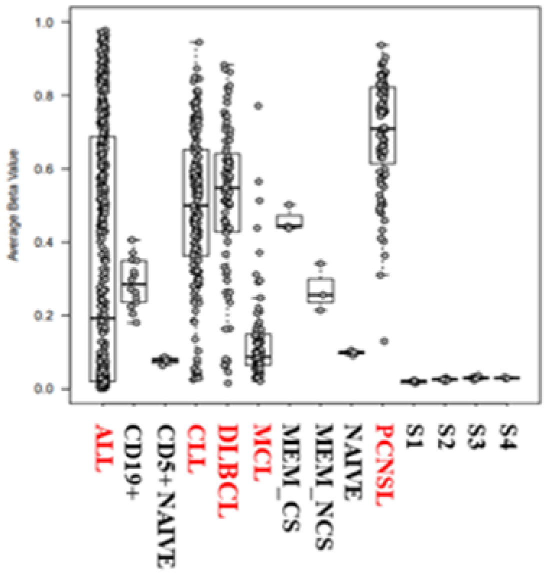

Expression

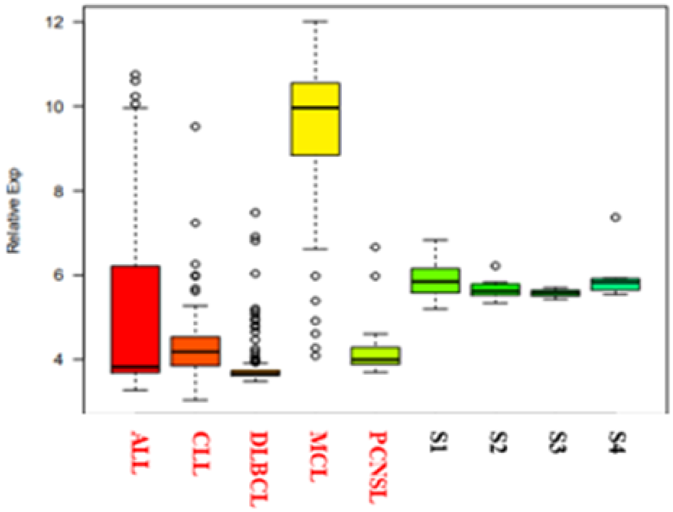

PAPPA

Methylation

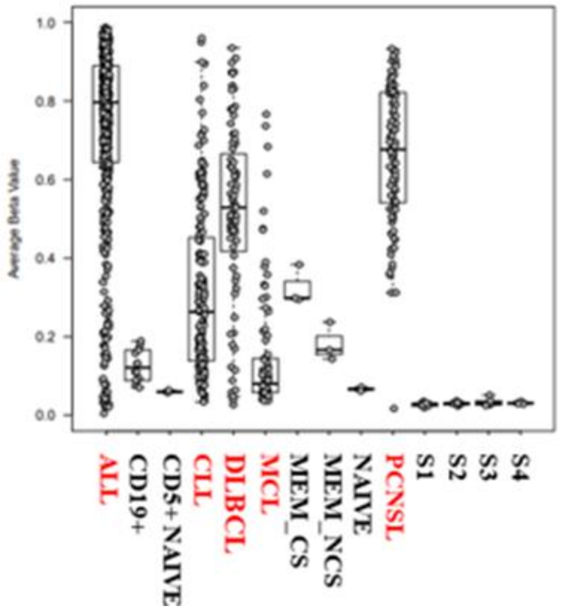

Expression

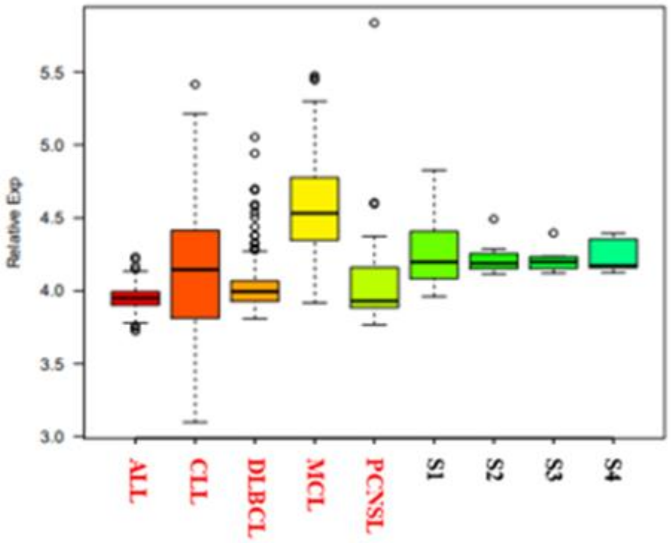

MFHAS1

Methylation

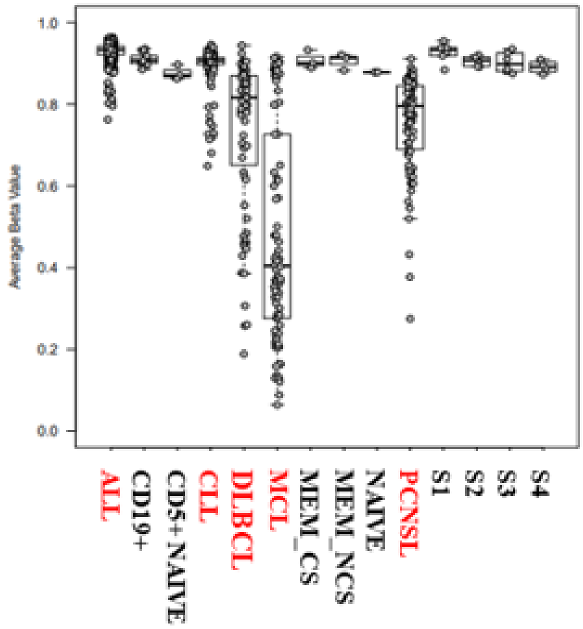

Expression

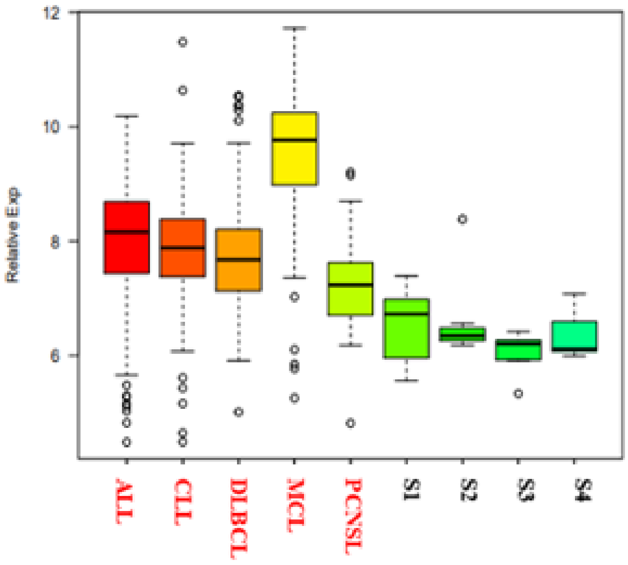

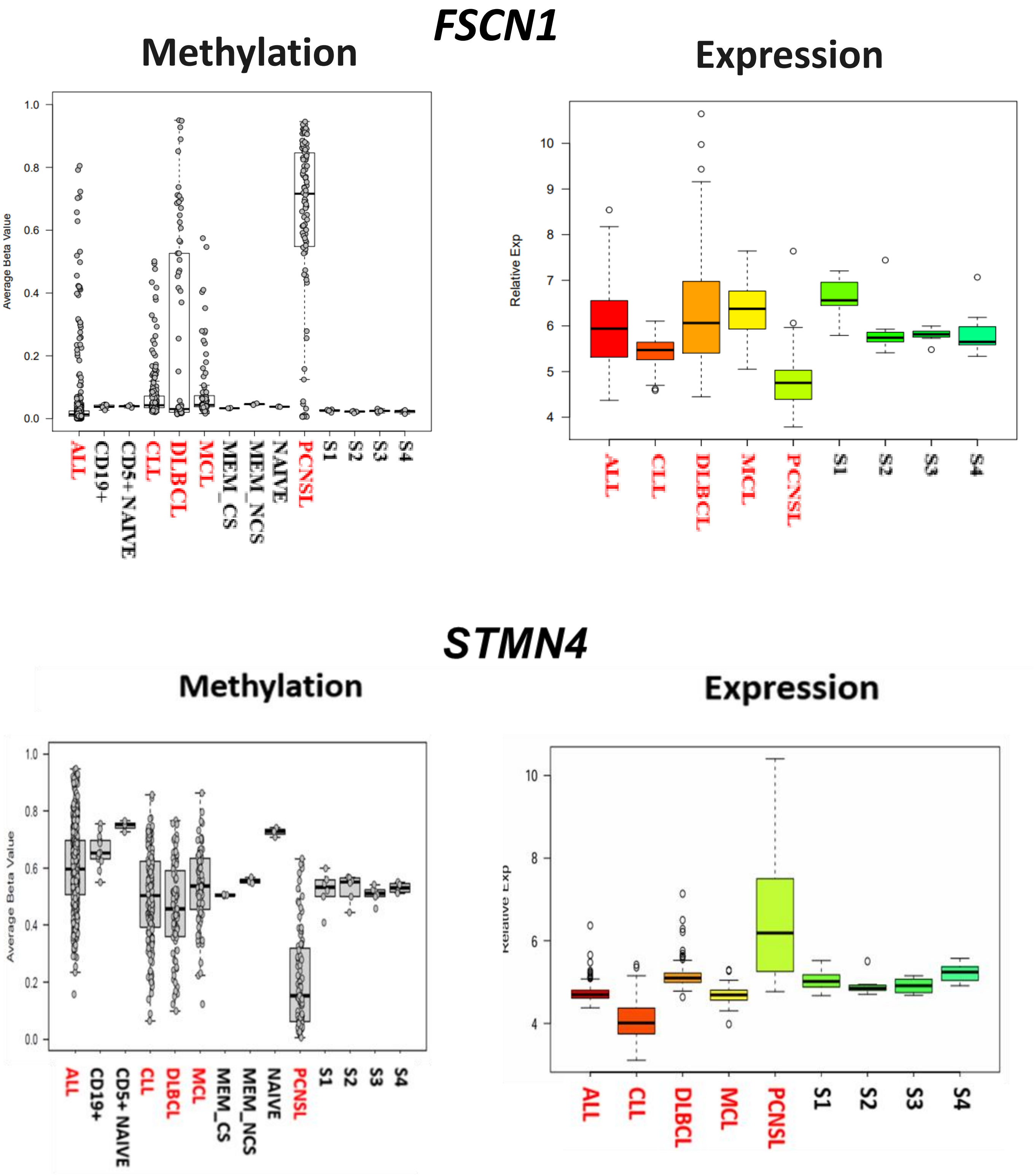

Supplementary Figure 6 – Methylation and expression patterns of candidates identified by Methylation Mapping of disease specific analysis. Methylation (left-hand graph) and expression (right-hand graph) patterns for the seventeen loci identified disease specific methylation and inverse, disease specific expression patterns. Gene names are indicated above each set of methylation/expression graphs. Malignant diseases are labelled in red, while normal cell populations are labelled in black.

Supplementary Figure 7

A

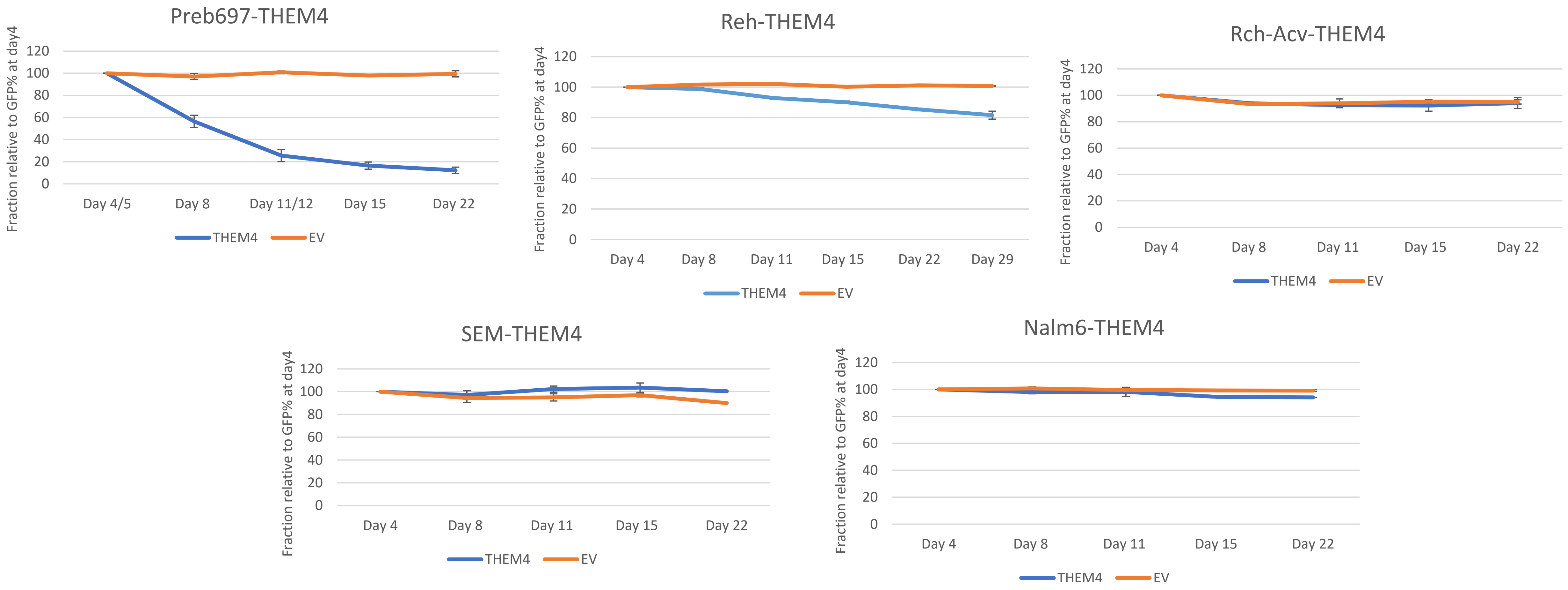

B

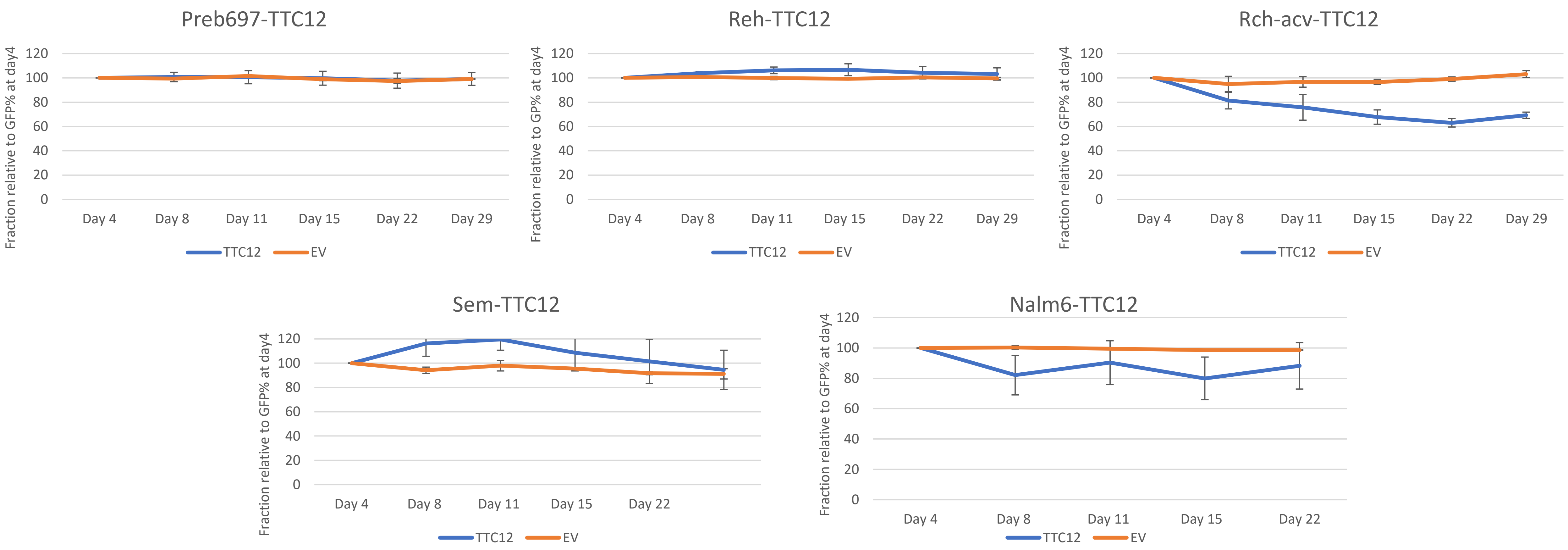

Supplementary figure 7 – Transduction of ALL cell lines with lentiviral vectors expressing tumour suppressor candidates. A) Five ALL cell lines were transduced with THEM4 expressing lentiviral construct (or an empty vector control). While PreB697 cells exhibited a rapid decrease in THEM4 expressing cells, suggesting a strong negative selection, rapid loss of THEM4 expressing cells was not seen in any of the other four cell lines. B) Five ALL cell lines were transduced with TTC12 expressing lentiviral construct (or an empty vector control). Rch-acv and NALM-6 exhibit a slight fall in the fraction of TTC12 expressing cells. However, loss occurs at a low rate and the other three cell lines do not exhibit loss of TTC12 expressing cells.
